# Structural and antigenic characterization of novel and diverse *Henipavirus* glycoproteins

**DOI:** 10.1101/2024.12.11.627382

**Published:** 2024-12-11

**Authors:** Aaron J. May, Muralikrishna Lella, Jared Lindenberger, Alex Berkman, Moumita Dutta, Maggie Barr, Rob Parks, Amanda Newman, Xiao Huang, Ujjwal Kumar, Kijun Song, Victor Ilevbare, Salam Sammour, Chan Soo Park, Radha Devkota Adhikari, Priyanka Devkota, Katarzyna Janowska, Yanshun Liu, Garrett Scapellato, Taylor N. Spence, Katayoun Mansouri, Robert J Edwards, Barton F. Haynes, Priyamvada Acharya

**Affiliations:** 1 Duke University, Duke Human Vaccine Institute, Durham NC 27710, USA; 2 Duke University, Department of Biochemistry, Durham NC 27710, USA; 3 Duke University, Department of Medicine, Durham, NC 27710, USA; 4 Duke University, Department of Integrative Immunology, Durham NC 27710, USA; 5 Duke University, Department of Surgery, Durham NC 27710, USA; $ Lead contact

## Abstract

*Henipaviruses* (HNVs), a genus within the *Paramyxoviridae* family, includes the highly virulent Nipah and Hendra viruses that cause yearly reoccurring outbreaks of deadly disease. Recent discoveries of several new *Henipavirus* species, including the zoonotic Langya virus, have revealed much higher antigenic diversity than currently characterized. Here, to explore the limits of structural and antigenic variation in HNVs, we construct an expanded, antigenically diverse panel of HNV fusion (F) and attachment (G) glycoproteins from 56 unique HNV strains that better reflects global HNV diversity. We expressed and purified the F ectodomains and the G head domains, characterized their biochemical, biophysical and structural properties. We performed immunization experiments in mice leading to the elicitation of antibodies reactive to multiple HNV F proteins. Cryo-EM structures of diverse F proteins elucidate molecular determinants of differential pre-fusion state metastability and higher order contacts. A crystal structure of the Gamak virus G head domain revealed an additional domain added to the conserved 6-bladed, β-propeller fold. Taken together, these studies expand the known structural and antigenic limits of the Henipavirus genus, reveal new cross-reactive epitopes within the HNV genus and provide foundational data needed for the development of broadly reactive countermeasures.

## Introduction

Henipaviruses (HNVs) are a genus of ssRNA viruses that includes the highly virulent Nipah virus (NiV) responsible for causing yearly reoccurring outbreaks of deadly disease. The *Henipavirus* genus is a part of the *Paramyxoviridae* family, and therefore related to other notable human pathogens such as the parainfluenza viruses, measles, and mumps (1). The combined possibility within the family for both rapid transmission, as with measles, and high lethality, as with NiV, further highlights pandemic risk and necessitates urgent research to establish preparedness that will enable a rapid and effective response to a future emergent threat. For HNVs specifically, their identification in diverse animal reservoirs in different geographical locations, their high risk of zoonotic transmission, and current lack of approved vaccines or therapies to treat HNV infection in humans highlight the high risk and pandemic potential of this genus.

HNVs have been studied since the discovery of Hendra (HeV) and Nipah viruses in 1994 and 1998, respectively (2–4). Additional members of the genus have been indentified since then, but it was not until 2022 with the discovery of Langya virus (LayV) that another species with pathogenicity in humans was confirmed (5). Like HeV and NiV, LayV was of zoonotic origin. However, unlike HeV, NiV, and most of the other known HNVs at the time, LayV was found to have likely originated from a shrew reservoir, rather than from fruit bats. Several concurrent studies identified more shrew-borne HNVs over a wide geographic range, indicating the existence of a largely uncharacterized clade within the Henipavirus genus (6–8). Among these studies are some untargeted surveillance efforts, revealing new HNV sequences among dozens of other sequences across multiple viral families (9).

From the perspective of vaccine or therapeutic countermeasure development for HNV infection, the most important HNV proteins to study are the attachment (G) and fusion (F) surface glycoproteins. These are the two sole surface-exposed proteins in HNV virions, and therefore the only targets for neutralizing antibodies (10). Together, G and F facilitate viral entry into host cells, with G responsible for receptor binding and F mediating membrane fusion. During this process, both the G and F proteins undergo important conformational changes, including the destabilization of the prefusion conformation of F by G. The specifics of these conformational steps, including how G destabilizes the pre-fusion F conformation or “triggers” its pre-fusion to post-fusion transition remain elusive. Given dramatic structural differences observed among the Paramyxoviridae attachment proteins, there may be no universal model that can describe receptor-mediated F protein triggering. Although a class I fusion protein (11), the HNV-F protein, along with all *Paramyxoviridae* F proteins, differs from the canonical class I fusion mechanism in the separation of the attachment mechanism into a separate protein (12). Whereas other viral fusion proteins proceed through the fusion conversion process by repositioning or removal of attachment subunits, Henipavirus F proteins can retain inherent metastability through F protein architecture alone.

In addition to mechanistic questions about HNVs, the antigenicity of these proteins and the possibility of cross-reactivity across the genus remains largely unexplored. While there are several known monoclonal antibodies (mAbs) that are able to neutralize both NiV and HeV (13–17), so far none of these have been found to have any reactivity with LayV (18, 19). Of the few mAbs known to bind to LayV, some have been noted to be cross-reactive with Mojiang virus (MojV), but not to NiV or HeV (19). Whether there are any broadly reactive anti-HNV antibodies or where the limits of cross-reactivity are within the genus considering newly-discovered species remains unknown. In order to address these genus-wide mechanistic and antigenic unknowns, we assembled a curated set of HNV G and F sequences that samples the diversity of the genus as broadly as possible in order to purify and characterize these proteins, biochemically, biophysically, and structurally.

### Identification and Classification of Henipavirus Species

To begin categorizing this diverse set of Henipavirus strains, we identified as many unique Henipavirus G and F proteins as could be found, assessed the phylogeny of these sequences, and developed a universal naming scheme. The NCBI BLAST tool (20) was employed to search for all available unique sequences related to the input sequence, using either the G or F amino acid sequence of either Hendra Virus (NC_001906.3, (21)) or Nipah virus, (NP_112027.1, (22)). In addition to utilizing BLAST, we carried out an extensive literature search to include other sequences that may not have been discoverable through BLAST, such as Gamak virus (6). After identifying related sequences, we narrowed the list to include only sequences from mostly complete genomes, rather than partial DNA sequences of only G or F proteins. Almost all the identified strains had genome lengths close to the canonical 18.2 kbp for Henipaviruses (23), with some species being slightly longer. No sequence in our panel had a deposited genome shorter than 11.2 kbp. The several sequences that were shorter than the rest were either Nipah-Malaysia or Hendra strains that were not recently discovered and have been well-characterized in the literature.

**Figure 1:**
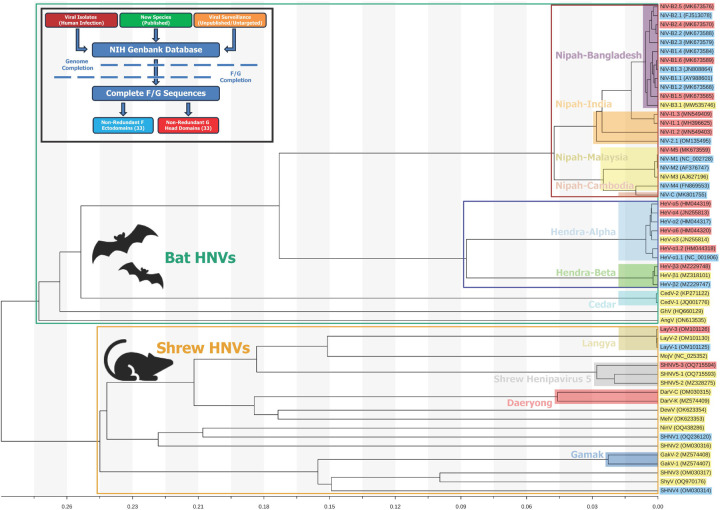
Selection and Classification of Henipavirus Species. **Upper Left:** A flowchart that describes the origin of sequences used and the filtering mechanisms, Genome and F/G sequence completion, used to assemble a list of non-redundant F and G proteins. **Tree:** A phylogenetic tree based on full-genome nucleotide sequences of the selected strains. Horizontal branch lengths are to scale to represent sequence divergence. Hollow boxes are drawn to represent broad groupings within the tree: Bat Henipaviruses, green, Shrew Henipaviruses, orange, Nipah viruses, red, Hendra viruses, dark blue. Solid highlight boxes are drawn to represent specific species or subspecies groupings: Nipah-Bangladesh, purple, Nipah-India, orange, Nipah-Malaysia, yellow, Nipah-Cambodia, salmon, Hendra-alpha, blue, Hendra-Beta, green, Cedar virus, cyan, Langya virus, tan, Shrew Henipavirus 5, grey, Daeryong virus, red, Gamak virus, dark blue. The tips of each branch are labelled with the strain names and associated gen bank accession number. Each strain is colored based on which type of Henipavirus protein was purified based on the strain: F protein only, blue, G protein only, red, both, yellow.

Next, the Clustal Omega tool (24) was used to align the G and F amino acid sequences. As our goal was to purify soluble ectodomain constructs, we truncated the sequences, using trends in the sequence alignments to decide on exact truncation points between species. Henipavirus F proteins were truncated at the C-terminal end of the ectodomain, at the site equivalent to Nipah virus residue 488 to be consistent with previous purifications of F ectodomains (18). For the G proteins, the boundaries of domains do not align as consistently across species as they do with the overall less variable F protein. For ectodomains, the N-terminal portion of the sequence up to the end of the transmembrane domain was removed, with the position set at the analogous site to Nipah virus residue 71. For G Head domains, the site analogous to Nipah virus residue 177 was selected as a suitable start point based on sequence analysis and available structural data for Nipah and Langya G proteins (13, 25). When comparing the truncated sequences, it was found that the amino acid sequences of several strains differed from other panel members only in their signal sequence, transmembrane domains, cytosolic domains, or other regions not included in the final constructs. Therefore, these redundant sequences were eliminated from the panel, and the remaining sequences have been assembled into a phylogenetic tree based on complete genomes (Figure 1).

To begin categorizing these sequences based on their phylogeny, we used the previous handling of Nipah virus naming in the literature. Previously, strains have been named based on the location the sequence was reported, specifically, Bangladesh (NiV-B) and Malaysia (NiV-M). Our tree supports the existence of potentially two further groupings, that we have also named based on location, India (NiV-I) and Cambodia (NiV-C) (Figure 1). With the Bangladesh strains (Figure 1), the phylogeny revealed the grouping of strains into two clades, prompting us to denote the two groupings as NiV-B1 and NiV-B2, respectively. From here, unique entries were designated an additional number to further classify the variant (i.e. NiV-B2.1, NiV-B2.2, NiV-B3.1, etc.). An identical scheme was applied for the India strains (Figure 1) which previously have been referred to as members of the Bangladesh clade (26). Malaysia strains were classified by number as well. Entry MK801755 was identified during surveillance in Cambodia in 2003, where it was isolated from *Pteropus lylei* fruit bats (27). Appearing distinct from most other Malaysian strains in our tree, it was assigned as a new Cambodia clade (NiV-C) (Figure 1).

Previous studies have established two clades for Hendra virus (28), in which the more recently discovered clade is referred to as HeV-g2. However, due to the use of G as an abbreviation for the attachment protein, we sought to adopt a different naming scheme to avoid confusion. Unlike Nipah, where genomic differences align with country borders, all Hendra virus discoveries have been in Australia. Therefore, the two distinct clades have been named Alpha (α) and Beta (β), with each member of the clade given a number. The other Henipaviruses that have emerged from a fruit bat reservoir (Figure 1) have all previously been named in the literature. These include Cedar Virus (CedV, (29)), Ghana Virus (GhV, (30)), and Angavokely Virus (AngV, (31)). Our panel includes two unique Cedar virus attachment proteins; therefore, these strains were numbered 1 and 2. There are only singular strains of Ghana and Angavokely viruses identified, however some evidence indicated that the originally deposited sequence for Ghana virus F protein may have included an inaccurate N-terminal end of the protein and a corrected sequence was suggested (32). While our phylogenetic tree only includes the one strain reported, our panel of purified proteins included both F protein sequences.

Among Henipaviruses found in shrews, several already have established names in the literature, including Mojiang Virus (MojV), Langya Virus (LayV), Gamak Virus (GakV), Denwin Virus (DewV), Melian Virus (MelV), and Ninorex Virus (NinV) (5–7, 33). For those with more than one strain reported, specifically Langya Virus (LayV) and Gamak Virus (GakV), each strain was designated with a number. However, many of the sequences were given repetitive names based on their shared collection site. To simplify these names, we began using the name “Shrew Henipavirus (SHNV)” with a number, thus establishing SHNV1, SHNV2, SHNV3, SHNV4, and SHNV5. For SHNV5 specifically, there were 3 distinct strains that the phylogenetic tree indicated were closely related enough to be considered the same species (Figure 1). As a result, we gave an additional number to each strain. Interestingly, these sequences were not all from the same collection event or species, indicating that SHNV5 is found in several Chinese shrew populations. Similarly, while Lee et al,. 2020 (6) previously reported the discovery of Daeryong Virus in Korea, our phylogenetic analysis revealed that Jingmen Crocidura shantungensis virus 2 (OM030315), detected in China, should be considered the same species as Daeryong Virus. Following the naming procedure used for Nipah virus, the strains were given the names Daeryong virus-Korea (DarV-K) and Daeryong virus-China (DarV-C). Again, this demonstrates that several of these newly discovered shrew-borne Henipaviruses are geographically well-distributed. In the case of Crocidura tanakae Henipavirus (OQ970176), we renamed the virus Shiyan Virus (ShyV) given the identification of the virus in Shiyan, China. A full listing of the panel, including original deposition names, accession codes, and sequence ranges used is available as Supplemental Table 1.

### Preparation of diverse Henipavirus F ectodomains

**Figure 2:**
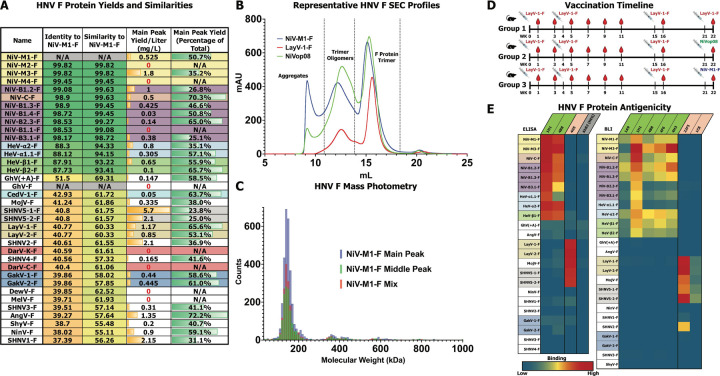
Characterization of Henipavirus Fusion Proteins. **(A)** Table of key statistics for each member of the F protein panel. Percent identities and similarities of the full amino acid sequence compared to NiV-M1-F are listed. GhV-F similarity values were excluded due to the likely inaccurate N-terminal sequence in the deposited sequence. Main peak yield is based on the final calculated protein yield after SEC. Main peak percentage of total is based on SEC area under the curve of the main peak as a fraction of all collected fractions. Color scheme for HNV species continued from Figure 1. **(B)** Representative SEC profiles of HNV-F Proteins with peaks annotated. Both the trimer oligomer peak, sometimes called the “middle peak,” and the main trimer peaks are collected separately. NiVop08 is a pre-fusion stabilized construct (35) not part of the panel but purified for a vaccination study and for comparison to wild-type proteins. **(C)** Mass photometry analysis of NiV-M1-F SEC components. Each individual measurement is assigned a calculated molecular weight by the instrument software, and individual results are binned in 10kDa bin sizes. The graph reports the count of events for each molecular weight bin with the main, middle, and mixed peak data overlayed. **(D)** Graphical timeline of a vaccination study using HNV-F antigens with mice. Each group was vaccinated with LayV-1-F and boosted with LayV-1-F or NiV antigens as marked. Blood draws and antigen injections occurred at the indicated weeks after the start of the study. **(E)** Heatmap of antibody binding to HNV-F proteins, on a spectrum of blue for low binding to red for high binding. The left-side graph is based on ELISA data with immobilized F proteins, antibody analytes, and anti-Human Fab-conjugated HRP as a reporter. The values for each combination are based on the log of the area under the curve for the plotted fluorescence over increasing concentrations. The right-side graph is based on Bio-layer interferometry (BLI) with immobilized F proteins and direct measurement of analyte to the BLI sensors, either mouse fabs or mouse IgG (22F5 and 1C8). The values for each combination are based on the maximum wavelength shift measured during the association phase of the BLI experiment. HNV-F antigens colored as in (A), antibody analytes colored as follows: green, known anti-NiV-F binding; salmon, known anti-LayV-F binding; gray, anti-influenza HA negative control antibody.

To prepare a panel of HNV F proteins we expressed constructs that encoded the ectodomains spanning residues 1 to the equivalent of 488 in NiV. Following the end of the ectodomain, we added a foldon trimerization domain (34) and a C-terminal Twin Strep tag (IBA Lifesciences). The proteins were expressed through transient transfection in HEK 293 cells and purified using their C-terminal Strep affinity tags followed by size exclusion chromatography (SEC). The yield of F proteins was variable, with some constructs expressing over 2 mg/L and others less than 0.1 mg/L (Figure 2A). For eight F proteins, the yields were too low to perform biochemical characterization. We found that yields could vary dramatically between strains that only differed by a small number of amino acids. For example, NiV-B1.1-F and NiV-B1.2-F differ by only three residues in the ectodomain, but while NiV-B1.2-F produced ~1mg/L, NiV-B1.1-F had no yield in both of two purification attempts. The sequence identity relative to NiV-M1-F is relatively high for both NiV and HeV strains, with all NiV sequences over 98% and HeV strains over 97% (Figure 2A). However, the sequence identity drops dramatically outside this grouping, with most other species at or below 40% sequence identity to NiV-M1-F. Sequence similarity also follows this drop outside the NiV and HeV clade, albeit with the percentages much higher, with no sequence dropping below 55% similar. When considering the phylogeny of HNV-F proteins on a basis of amino acid sequence, the tree remains very similar to the overall genetic-based tree with several small differences. Specifically, the distinction between NiV-C-F and NiV-M strains is more apparent, the F proteins of ShyV, SHNV3 appear to be very closely related, and AngV-F appears to be distinct from all other HNV strains, rather than part of the bat clade as seen through full-genome phylogeny (Supplemental Figure 1, Top).

HNV-F ectodomains typically elute from SEC with three peaks, one that elutes immediately after the column void volume, a very broad “middle peak,” and the main peak that corresponds to the molecular weight of an F trimer eluting last (Figure 2B, Supplemental Figure 2), with the ratio of the peaks to one another differing from strain to strain (Figure 2B). Furthermore, the middle peak of some strains can sometimes be seen to have a shoulder, indicating there could be multiple sized components eluting within this peak. The SDS-PAGE profile of the middle peak components is similar to that of the main trimer peak, with a major band observed at a position identical to where the band corresponding to the main trimer peak appears and some minor higher molecular weight bands observed sometimes, indicating that the presence of the middle peak is not caused by any kind of covalent linkage, nor any differential glycosylation significant enough to change the apparent molecular weight by several hundred kDa (Supplemental Figure 3). This conclusion is supported by mass photometry analysis of the middle and main peaks. Both peaks individually or mixed together generated an essentially identical molecular weight profile, with each being dominated by the expected molecular weight of the trimer, and both showing a very small presence of species twice to three times the weight of the trimer (Figure 2C). Furthermore, negative stain electron microscopy (NSEM) analysis of each middle or main peak reveals largely comparable sets of two-dimensional class averages. The pre- and postfusion shapes typically seen previously (18) can be seen throughout the panel for both middle and main peak components (Supplemental Figure 4). Thus, the middle peak appears to be indicative of transient non-covalent association of the F ectodomain trimers.

### Antigenicity of HNV F ectodomains

We next sought to characterize the antigenicity of the F ectodomain panel. Given the novelty of recently identified HNV species and limited availability of antibody sequences with known reactivity to LayV and other newly categorized members of our panel, we sought to elicit F-directed antibodies from vaccination of mice with several HNV-F antigens (Figure 2D). One group was given four injections of the LayV-1-F ectodomain, and for two others, the fourth injection of LayV-1-F was replaced with NiV, either the wild-type NiV-M1-F, or NiVop08, a pre-fusion stabilized NiV construct (35). By boosting with NiV constructs, we sought to elicit broadly reactive antibodies. We identified two murine IgG1 antibodies, named 1C8 and 22F5, both from group 2, with reactivity to LayV-1-F.

Either through ELISA or BLI, we assessed the binding of these newly identified antibodies, as well as previously identified anti-NiV or anti-LayV antibodies, to our panel of HNV-F ectodomains (19, 36). 1H1 and 4B8, previously identified as anti-NiV-F antibodies, displayed broad reactivity to all NiV and HeV strains, but extremely limited reactivity to all other species. 4G5, previously identified as an anti-LayV-F and MojV-F antibody, strongly bound not only to LayV-F and MojV-F, but also to SHNV5–1 and SHNV5-2-F, thus indicating that antibodies targeting the 4G5 epitope will be broader than was previously known. (Figure 2E, Supplemental Figure 5–6). A panel of mouse anti-NiV-F Fabs broadly reacted to nearly all NiV and HeV antigens, as expected. The two mouse antbodies identified in our vaccination study demonstrated strong reactivity not only to LayV-F proteins, but also to MojV-F and both SHNV5 strains as well (Figure 2E, Supplemental Figure 7). Furthermore, 22F5, which generated stronger binding signals than 1C8, bound to SHNV2-F as well. We assessed the binding of 22F5 to LayV-1-F ectodomain constructs using Surface Plasmon Resonance (SPR) (Supplemental Figure 7). 22F5 bound to LayV-1-F wild type ectodomain that consists of a mix of pre- and post-fusion F proteins, to heat treated LayV-1-F that consists of only post-fusion F, and pre-fusion stabilized constructs of the LayV-1-F ectodomain, thus confirming that the 22F5 epitope is accessible in both the pre- and post-fusion conformations of the F protein. We performed competition assays with 4G5 antibody, which is the only LayV F directed antibody known currently. It was previously shown that the 4G5 antibody cross-reacts with LayV and MojV F proteins and binds a stalk epitope that is accessible in both the pre- and post-fusion conformations of the F protein (19). We showed that 22F5 bound LayV-F in the pre- and post-fusion conformation with low nM affinity. We also showed that 22F5 binds LayV-F, MojV-F, SHNV5-1-F, SHNV5-2-F and SHNV2-F, and does not compete with 4G5. These results indicate that 22F5 targets a novel epitope on the HNV F proteins and has greater breadth than any previously identified antibody targeting shrew HNVs.

### Differential Scanning Fluorimetry of HNV F ectodomains

**Figure 3:**
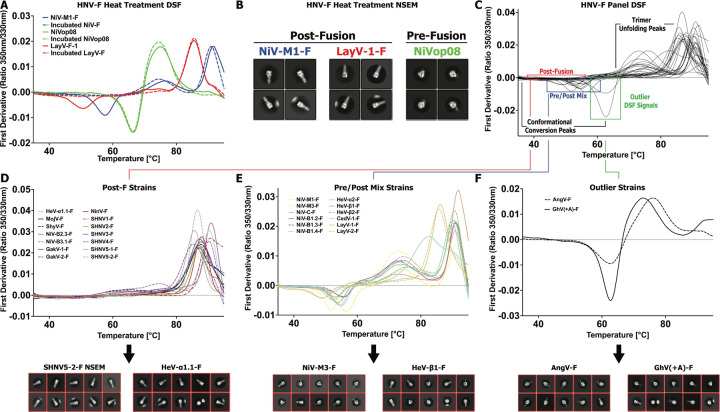
Thermostability of Henipavirus F Proteins. **(A)** DSF profiles of a set of HNV proteins showing the first derivative of 350/330nm emission ratio as a function of temperature. Dotted lines of the same color represent the DSF profile of the same proteins after first being incubated at 60°C for 90 minutes. **(B)** Two-dimensional NSEM class averages of a portion of the heat-treated samples from A. **(C)** DSF profiles of diverse HNV-F species. Sections of the profile are noted indicating the conformational steps occurring at those temperatures, with highlighting of peaks of interest. **(D)** A subset of DSF profiles from C, containing only those species that do not demonstrate any noticeable conformational conversion peak. Below are representative two-dimensional NSEM class averages for select members of this subset. **(E)** A subset of DSF profiles from C, containing only those species that demonstrate some presence of the standard conformational conversion signal. Below are representative two-dimensional NSEM class averages for select members of this subset. **(F)** A subset of DSF profiles from C, containing only those species that demonstrate a profile notably different from the typical HNV-F species. Below are representative two-dimensional NSEM class averages for the two members of this subset.

To assess the stability of the purified F ectodomains, we performed Differential Scanning Fluorimetry (DSF) using a label-free method that measures changes in intrinsic fluorescence as the proteins unfolded. We first performed DSF on two wild-type proteins, NiV-M1-F and LayV-1-F, and one prefusion-stabilized NiV-F construct, NiVop08 (35). The DSF profiles were consistent with our previous reports (18). With each peak in a DSF profile indicating a conformational event, for the unstabilized F ectodomains we observed one transition between 45–65°C and one or more transitions at higher temperature, up to over 90°C (Figure 3A). LayV-1-F showed two transitions with a negative peak at 50°C and a positive peak at 86°C. NiV-M1-F showed three transitions with a negative peak 56°C, and positive peaks at 76°C, and 92°C. NiVop08 showed two transitions, a negative peak at 66°C and a positive peak at 76°C. We next measured the the DSF profiles of the same set of proteins after pre-incubation at 60°C for 90 minutes, based on previous observations that heat treatment could force conformational conversion in *Paramyxoviridae* F proteins (37). Overlaying all DSF profiles revealed that the lower-temperature negative peak present in all three proteins initially was eliminated in only the two wild-type proteins after heat incubation (Figure 3A). Negative Stain Electron Microscopy (NSEM) confirmed that the wild type proteins have been fully converted to the postfusion conformation, as revealed by the characteristic elongated six-helix bundle present in the 2D class averages (Figure 3B). The NiVop08 prefusion stabilized construct retained the more compact shape characteristic of the prefusion state (Figure 3B). Together, these results indicate that the negative DSF peak present around 45–60°C is indicative of a pre- to post-fusion conformational change taking place.

We next assessed all purified F proteins for which there was sufficient material purified, typically under 5µg, with DSF. We observed a wide range of conversion peak intensities and temperatures (Figure 3C–F). While all proteins generated a large positive peak at high temperature indicative of a denaturation event, not all proteins produced a detectable conversion peak. 14 proteins had essentially no detectable signal prior to the high temperature peak (Figure 3D). Two-dimensional class averages from NSEM analysis revealed consistent and universal postfusion conformation for these proteins, confirming DSF results. Though not exclusively, most of the proteins with no conversion peak are part of the shrew-borne clade of the Henipaviruses, and inversely, all of the shrew clade proteins have no conversion peak, except for Langya virus strains. Many of the shrew clade F proteins could not be assessed due to insufficient purification yield, and these results may suggest it could be due to inherent instability. Among the F proteins that generated a conversion peak, there were shifts in temperature and intensity (Figure 3E). These changes did not appear related to species differences, with NiV-M1-F and NiV-M3-F, separated only by one amino acid mutation, being right- and left-shifted compared to the average, respectively. Both Langya virus strains were the most left-shifted of the set. Two-dimensional NSEM classes show a mix of pre- and postfusion conformations in this set, indicating that the presence of this negative peak indicates only some amount of prefusion conformation present, rather than presence of a uniform set of prefusion proteins. Between the conversion peak and the final high temperature peak is typically a broad, positive peak, present with differing intensities and shapes. Further investigation would be required to determine if this peak is either part of any natural conformational event or part of the denaturation process.

Two proteins of the panel, AngV-F and the sequence-corrected version of GhV-F that restores the signal peptide, GhV(+A)-F, display DSF profiles that are outliers to the rest of the panel (Figure 3F). Interestingly, both proteins display a profile that seems closer to NiVop08, with their high temperature positive peak significantly left-shifted. However, these are wild-type proteins that can be assumed to be capable of undergoing normal conformational conversion, barring unexpected and dramatic mechanistic differences. Two-dimensional NSEM classes for these two proteins show the presence of largely, but not exclusively, prefusion conformations.

Since DSF is reliant on primarily Tryptophan residues to generate signal, it is possible that differences in DSF profile could arise from differing number and position of Tryptophans, however, the F ectodomain has only one Tryptophan that is almost universally conserved, with only CedV-1-F mutated at this spot (Supplemental Figure 8). Furthermore, AngV-F contains one additional tryptophan unique to AngV-F. All F ectodomain constructs additionally contain one tryptophan in the foldon tag used to assist with trimerization and two in the twin-strep tag used for purification. In general, sequence variability is likely not a significant factor in DSF analysis of F proteins.

### Cryo-EM structures of diverse Henipavirus F proteins

**Figure 4.**
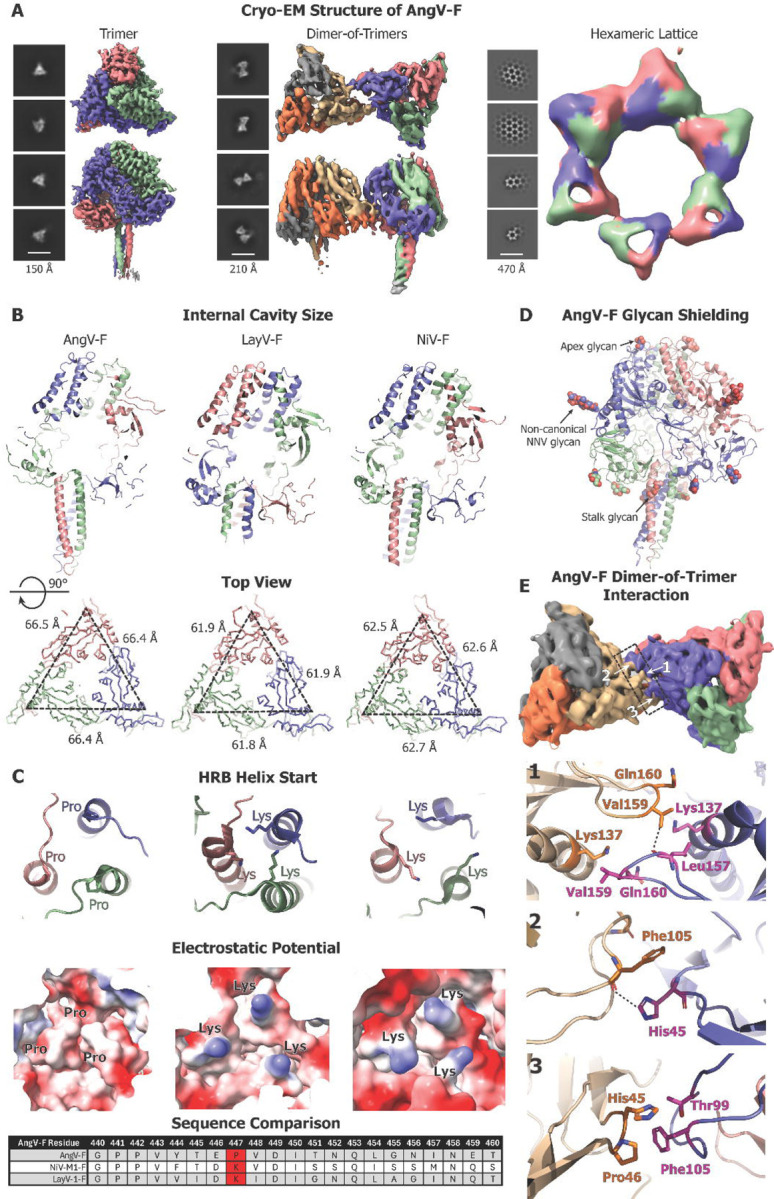
Cryo-EM structures of Diverse Henipavirus F Proteins. **(A)** Cryo-EM structure of the AngV-F ectodomain. Three different particle populations in the AngV-F and their representative 2D classes. Cryo-EM reconstruction of top and side view shown for AngV F trimer (left), dimer-of-trimers (middle) and hexameric lattice (right). **(B)** Comparison of the AngV-F, LayV-F and NiV-F proteins. Top. A slice through the F-protein visualizing the central cavity and the placement of the stalk region within the F-protein. Bottom. A top-down view showing measurements. **(C)** Zoom-in view of the stalk region featuring amino acid present at the start of helix bundle (top) and their varied electrostatic potentials (bottom), with sequence alignment at the local region between each of the three species below. The HRB start residue is marked red in the sequence alignment. **(D)** AngV-F cryo-EM structure colored by protomer with the glycans shown as spheres and colored by element. Glycans at the different regions (Apex, non-canonical NNV, stalk) are marked with arrows. **(E)** The dimer-of-trimer interaction interface is shown (top), along with their molecular-level interactions (bottom).

To understand the structure of an HNV-F protein that has divergent properties from the currently characterized F proteins, we determined the structure of the AngV-F protein by cryo-EM single particle analysis (Figure 4, Supplemental Figure 9–11, Supplemental Table 2). The cryo-EM dataset revealed three distinct particle populations (Figure 4A). The most populated class was of the isolated F ectodomain trimer, followed by a dimer-of-trimers population, and finally a population where the F proteins formed a hexameric array. The isolated AngV-F trimer was refined to a resolution of 4.0 Å and used for detailed examination of the F ectodomain structure and for comparing to the previously known structures of other F proteins. In cryo-EM structures of the HNV-F proteins the stalk region is typically poorly defined (14, 18, 36, 38, 39). Our AngV-F cryo-EM structure revealed well-defined density for the stalk, allowing atomic-level modeling.

The AngV F ectodomain exhibited a large central cavity compared to NiV-F and LayV-F proteins. This expanded cavity is the result of both the stalk region not inserting as far into the cavity as in other HNV-F proteins and the domains surrounding the cavity being spaced farther apart (Figure 4B). In the previously determined HNV-F structures, the helical stalk region begins with a lysine at its N-terminal end. This trio of lysines forms the bottom of the internal cavity. In AngV-F, we observe a proline at the beginning of the stalk helix, also known as the HRB helix (Figure 4C). The surface electrostatics of this region is strikingly different at this position for the AngV-F compared to the NiV-F and LayV-F structures. The proximity of the lysines in LayV-F and NiV-F and their electrostatic repulsion could be a source of metastability of the pre-fusion form of the F protein, as charge repulsion may force apart the HRB helix during conformational conversion. The absence of this lysine trio and replacement with a proline cap at the N terminus of the HRB helix may lend greater stability to the pre-fusion form of the AngV-F protein. Notably, all species in our panel have either a lysine or arginine at this site, with only AngV-F substituting the proline (Supplemental Sequence Alignment F).

Several glycans could be resolved (N61, N93, N353, N434 and N458), including ones analogous to those previously defined in HNVF structures (38, 39). The glycans shield known sites of vulnerability in *Paramyxoviridae* F proteins, including at the apex (N61), near the fusion peptide (N93 in Domain III), and on the stalk domain (N458). Interestingly, the glycan proximal to the fusion peptide utilizes a non-canonical NNV sequence (Figure 4D). The MojV-F and SHNV2-F proteins also have NNV sequences at this site that may be similarly glycosylated. We also observed glycans present in Domain I (N353) and Domain II (N434).

Interactions between the AngV-F trimers were mediated through intermolecular contacts along the DI and DIII domains previously described (10). These include hydrophobic interactions in DIII between L157 and V159 on neighboring ectodomains, both positioned on a loop previously identified as an important antigenic site (10, 36) (Figure 4E). Furthermore, there are charge interactions involving H45 from DI and T99 and F105 of DIII (Figure 4E). Similar oligomeric states between pre-fusion F protein molecules (trimer, dimer-of-trimers, trimer-of-trimers) were also found in the cryo-EM dataset of a Hendra virus F protein (HeV-α2-F) (Supplemental Figure 11). AngV-F dimers resemble “mirror-like” protein dimers, with each exterior interaction surface of a protomer available for dimerization. These dimers, in turn, interact with protomers from three other trimers, forming hexameric structures. Each trimer is aligned within the hexameric assembly, and in turn, hexameric ring units interact in a consistent plane, distinct from an NiV-F crystal structure in which the lattice was not within one plane (38).

Taken together, our cryo-EM structures reveal substantial diversity in the pre-fusion F protein while maintaining an overall conserved architecture. They also reveal multimerized pre-fusion F proteins adding to the growing body of literature that suggest a role for the multimeric assemblies in the context of the virion and suggest a distinct mode of F protein multimerization leading to the formation of hexameric lattices for the AngV-F protein.

### Purification and characterization of diverse Henipavirus G head domains

**Figure 5:**
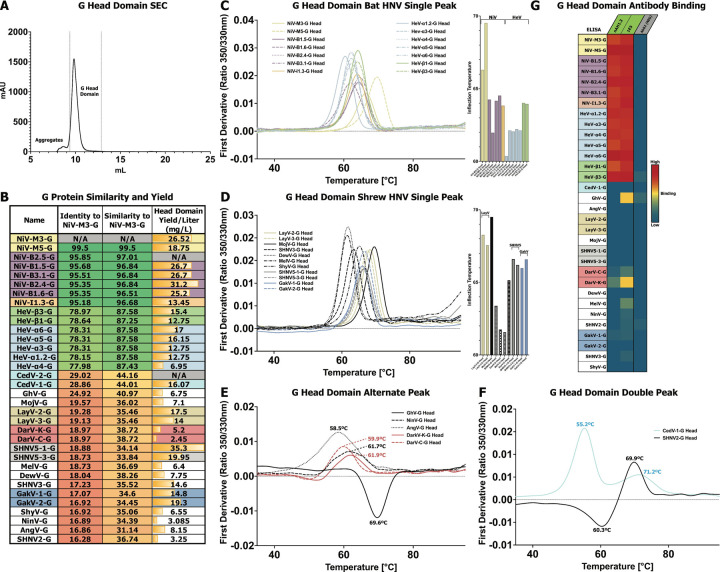
Characterization of Henipavirus G Proteins. **(A)** Representative SEC elution profile for G head domains with collected fractions designated. **(B)** Table of key statistics for each member of the G protein panel. Percent identities and similarities of the full amino acid sequence compared to NiV-M3-G are listed. Yields for species are marked gray and N/A for species that had a unique G ectodomain but not a unique head domain and therefore were not purified as part of this panel. Main peak yield is based on the final calculated protein yield after SEC. Color scheme for HNV species continued from Figure 1. **(C-F)** DSF analysis of G head domains, reporting the first derivative of 350/330nm ratio as a function of temperature. The panel’s results are split based on their phylogeny and general DSF profile: C, bat-clade proteins with a single DSF peak; D, shrew-clade proteins with a single DSF peak; E, proteins with alternative peak shapes (broad or negative); F, proteins with double peaks. In (C-D), a side plot of inflection temperatures is shown for each species in the associated graph. In (E-F), inflection temperatures are drawn directly on the plot. **(G)** Heatmap of antibody binding to G head domains, on a spectrum of blue for low binding to red for high binding. The graph is based on ELISA data with immobilized G head domains, antibody analytes, and anti-Human Fab-conjugated HRP as a reporter. The values for each combination are based on the log of the area under the curve for the plotted fluorescence over increasing concentrations. G head domain antigens colored as in (A), antibody analytes colored as follows: green, known anti-NiV-G binding; salmon, known anti-LayV-G binding; gray, anti-influenza HA negative control antibody.

To analyze our panel of HNV-G proteins, we began with a focus on the isolated head domain, which is responsible for receptor binding and is an important antigenic site. To allow for the previously described head domain sequences to be secreted in a mammalian expression system, we added an artificial secretion signal (40), and appended a C-terminal 8x His tag. The G head domains were transiently transfected and affinity purified with cobalt resin affinity chromatography, followed by SEC. The typical SEC profile for head domain proteins reflects the simple nature of their single-domain fold. All proteins eluted with a consistent single main peak (Figure 5A), typically with a small, higher molecular shoulder or peak that is co-eluted aggregates. Yields for the head domains ranged from 2.5 to 35.3 mg/L (Figure 5B), with the majority above 10 mg/L.

Compared to F proteins, HNV-G proteins demonstrate much greater variability within the genus. Sequence identity values are only ~78% between NiV and HeV, and drop to below 20% for shrew-clade G proteins, compared to NiV (Figure 5B). The sequence similarity scores are also rather low, with most shrew-clade strains around 35% similarity to NiV (Figure 5B). When considering amino acid-based phylogeny, the relationships between strains remains similar. One notable difference is that ShyV-G and SHNV3-G, as with their F counterparts, appear more closely related than their full genome would suggest (Supplemental Figure 1, Bottom). This limited similarity indicates that there could be greater antigenic, structrual, and even mechanistic differences between different HNV-G proteins than among F proteins.

We used DSF to assess the thermostability of the G head domains. Most proteins generated a profile with a single peak between 60–70°C. Among bat-clade strains demonstrating single-peak profiles, there were some small shifts in the inflection temperature that was largely consistent within species groupings (Figure 5C). The NiV-M, B, and I clades generally had higher inflection temperatures than HeV strains (Figure 5C). Among HeV strains, α had notably lower inflection temperatures than almost all other strains. Shrew-clade strains demonstrating single-peak profiles also had a diversity of inflection temperatures that tracked with species groupings (Figure 5D). The closely-related LayV and MojV showed the highest inflection temperatures for their G head domains, with the DewV and MelV G head domains having much lower inflection temperatures. However, several species generated profiles distinct from the most common result. Specifically, several species generated a rather broad and left-shifted peak, and another, GhV-G, inverting the main peak, instead generating a negative peak (Figure 5E). Two species, CedV-1-G, and SHNV2-G generated profiles with multiple peaks (Figure 5F). Unlike the relatively high conservation of tryptophan positions in F proteins, G proteins display a much higher level of sequence diversity (Supplemental Figure 8). Indeed, there are significant differences in tryptophan positions in species that generated outlier profiles, CedV, GhV, AngV, and SHNV2 especially.

We used ELISA to assess the antigenicity of our G head domain panel with two previously characterized anti-NiV-G antibodies, nAH1.3 and 1E5 (41, 42). These two antibodies demonstrated strong reactivity to all NiV and HeV constructs in the panel, but very limited reactivity outside this set. 1E5 demonstrated marginal reactivity to several species, specifically GhV, both DarV species, and MelV. Interestingly, nAH1.3 also demonstrated limited reactivity to DarV-K-G only (Figure 5G, Supplemental Figure 12).

## Structure of Gamak virus attachment protein head domain

**Figure 6:**
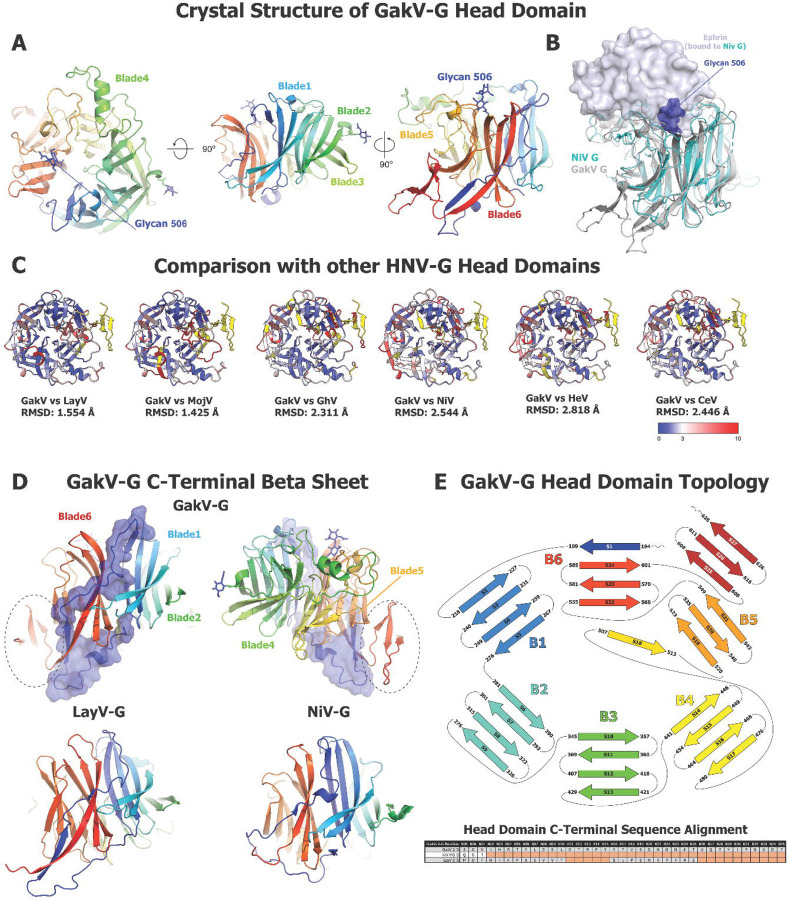
Crystal structure of GakV G Protein Head Domain. **(A)** Three views of the GakV-G head domain colored in rainbow. **(B)** Superposition of GakV-G head domain on Ephrin-bound NiV-G head domain (PDB: 2VSM, (43)). GakV-G is colored gray, NiV-G cyan and Ephrin light blue. Ephrin is shown as a transparent surface. The GakV-G glycan 506 is shown as blue spheres. **(C)** Overlay of GakV-G head domain on the G head domains from other HNV species. PDB Codes: LayV-G, 8K80 (Unpublished); MojV-G, 5NOP (60); GhV-G, 4UF7 (61); NiV-G, 3D11 (62); HeV-G, 6VY4 (15); CedV-G, 6P72 (63). **D.** Top. Two views of the GakV-G head domain colored in rainbow with the N terminal Asp to His218 shown as a transparent surface. The additional minidomain defined in the GakV-2-G head structure is highlighted within a dashed oval. Bottom. G head domains of LayV and NiV. **E.** Topology diagram of GakV-G protein with sequence alignment of select HNV species below.

Henipavirus G proteins are tetramers (dimer of dimers), with each monomer, in the ectodomain, composed of a helical stalk domain, a neck domain involved in oligomerization and control of quaternary structure, and a 6-bladed, β-propeller head domain where the receptor-binding site is found. A crystal structure of the GakV-2-G protein head domain determined at 1.4 Å resolution (Figure 6A, Supplemental Table 3) showed the expected 6-bladed β-propeller architecture that is well-conserved among HNV-G proteins (Figure 6A). A well-resolved glycan was observed in the region that overlays with the Ephrin (EFN) receptor binding surface in the NiV-G head domain (Figure 6B). While NiV, HeV, GhV and CedV bind the Ephrin family of receptors at a common receptor binding surface, the receptors for many Henipaviruses, including LayV, MojV, and GakV remain unknown. Though EFNB2/3 engages the same face of the head domain in NiV and HeV sialic acid does in other Paramyxoviruses (43, 44), the measles virus receptor, CD150, binds at the side surface of the head domain, making major contacts along blades 5 and 6 (45). The glycan present in the GakV-2-G head domain structure would likely hinder binding of a protein receptor in the EFNB2/3 site, suggesting that GakV either uses a smaller receptor, binds along a side face, as in measles virus, or adopts a different, yet to be discovered mode altogether. The Ephrin binding site is an immunogenic site in NiV/HeV and the target of neutralizing antibodies. An addition of a glycan in the GakV-2-G head domain would pose a steric barrier to the elicitation of antibodies targeting this site, suggesting that the GakV-2-G protein will have an antigenic profile distinct from the Ephrin-binding HNV-G proteins.

Though the GakV-2-G head domain structure was overall similar to that of other head domains (Figure 6C), some differences were noted. The N-terminus of the head domain appeared well ordered and packed against the 6-blade β-propeller core. Residues 194–199 threaded between blades 5 and 6, contributing a β-strand to blade 6 as it continued to lead into blade 1. This stretch of residues also contacted blades 2, 4, and 5 thus forming a connection between different secondary structures within the β-propeller. Interestingly, a novel minidomain was observed at the C-terminus of the head domain, spanning residues 606 to 628, that formed a three-stranded β-sheet stacked against blade 5 of the β-propeller (Figure 6D). This additional compact and ordered domain was distinct from the previosuly known structures of *Henipavirus* G protein head domains.

Taken together, the structure of the GakV G head domain illustrates the structural diversity that can be accommodated within the conserved β-propeller fold to impact receptor tropism and antigenicity.

## Discussion

Over the past few years, there has been a rapid expansion of identified species and strains in the *Henipavirus* genus. Given the history of zoonotic transmission with these viruses, charcterization of these novel species is an important first step for pandemic readiness. Here we characterize a diversified panel of HNV F and G proteins to construct a panel that better reflects the global diversity of HNVs. Up until recently, many of the strains in our expanded panel of Henipaviruses were totally uncharacterized and their relationships to other Henipaviruses unknown. Now, we have been able to identify two new species groupings containing strains that were identified independently. Given that the two strains of Daeryong virus were from sample collection in two different countries (Korea and China), this confirms that just as with Nipah, there is a likely a broad geographic range of these newly-identified, shrew-borne Henipaviruses.

Purifying and characterizing the F protein ectodomains of these strains has revealed that despite low sequence identity, HNV-F proteins share many architectural and biophysical similarities, a trend we previously noted by comparing Langya virus to Niaph virus (18), and one that is apparent with our species panel. One of the most notable similarities was the essentially universal presence of some level of oligomerization between F trimers. This was apparent from the SEC profiles, but yet the presence of trimer multimers did not seem to correlate with other parameters measured, such as purification yield, metastability, or phylogeny. It appears instead that the formation of these complexes is transient and dependant on the concentration and environment of the F protein, as suggested by the almost exclusive presence of trimer molecular weights detected by mass photometry, where sample concentration is much lower than during size-exclusion chromatography and structural analysis. Regardless, the ubiquity of this type of interaction indicates that oligomerization of F proteins likely is a conserved trait within Henipaviruses.

In the case of Angavokely virus, the formation of a hexamer lattice of F protein trimers, was observed in the cryo-EM datasets. The existence of a hexamer-of-trimers arrangment has been noted previously with NiV (38), though the biological relevance was limited by the use of crosslinking to stabilize the arrangement. Our structure demonstrates the ability of AngV-F to multimerize in this way without any such assistance, and, critically, without the involvement of the transmembrane domain, viral envelope, cytoplasmic domain, or viral matrix protein. The hexameric lattice is formed purely through ectodomain interactions, validating the characterizations of inter-trimer interactions previously reported for NiV (46), yet distinct in the implicated residues, with the AngV-F interaction involving DI and DIII, while the NiV-F interaction is dominated by DII residues.

Previous studies have defined the lattice of *Paramyxoviridae* and *Pneumoviridae* F proteins, with various arrangements being determined. In one case, hexameric lattices have been observed, as with a whole-virion tomography structure of parainfluenza virus 3 (37). In others, such as with measles virus and respiratory syncytial virus, alternate arrangements of fusion and attachment proteins have been observed (47). Our results with AngV-F suggest that HNV-F proteins may adopt the hexameric arrangement, though structural studies of pseudoviruses and whole virions would help confirm this finding. Furthermore, it cannot be ruled out that the formation of a hexameric F lattice may comprise one stage of HNV-F functionality, as the arrangment could shift during some type of maturation event or during G-mediated triggering.

While several anti-NiV and anti-HeV neutralizing antibodies have been well-characterized, the antigenicity of novel HNV strains had not yet been explored. By assessing the binding of a panel of both previously identified anti-HNV antibodies and newly discovered antibodies elicited through vaccination, we can now better describe the antigenic landscape of HNV proteins. As expected, we saw little ability of anti-NiV/HeV antibodies to bind to non-NiV/HeV proteins, with the exception of 1E5, which demonstrated some level of reactivity to select strains outside this group. Most interestingly, we found clear antigenic overlap between the F proteins of LayV, MojV, SHNV5, and to a lesser extent, GakV. Both a previously reported anti-MojV-F antibody, 4G5, and our vaccine-elicited antibodies, 22F5 especially, bound to these strains. When considering our phylogenetic analysis, both by full genome and amino acid sequence, it can be seen this cross-reactivity spans distantly related strains. SHNV5 appears to be more distantly related from LayV than HeV is from NiV. GakV is even more distant phylogenetically, appearing to be roughly as distant from LayV as CedV is from NiV.

With a group of viruses prone to such sequence diversity, one major concern in the event of a possible widespread outbreak of disease in humans is that mutations would occur so quickly that the immunity conferred from prior infection or immunization would not be able to track with newer strains. This phenomenon can be observed with yearly outbreaks of new influenza strains or with the progression of the SARS-CoV-2 pandemic, where new strains emerged and quickly dominated the pool of circulating virus as they evaded prior immunity. A key focus of efforts to address this possibility have long been designing immunogens that can elicit antibodies with broad coverage of the currently exisitng, and hopefully, future diversity of strains. Being able to draw antigenic boundaries within the *Henipavirus* genus is a foundation for similar efforts, and here we see that while the prospect of cross-reactivity over the entire genus remains elusive, there is already demonstrated cross-reactivity among a large portion of the shrew-borne clade. Of course, neutralizing potency is an important concern in this effort, and it will be important to assess 22F5’s potency as we continue to identify antibodies against novel Henipaviruses.

In addition to antigenic characterization, our biophysical characterizations of HNV-F proteins now gives us a tool with which we can assess their metastability. The pre- and postfusion conformations of HNV-F proteins are very visually distinct, allowing for relatively simple assignment of conformational states in a structural dataset. However, the process of obtaining structural data for such a large panel can be expensive and time-consuming. Therefore, we worked to develop our DSF-based assay to be able to indicate the conformational state without relying on structure determination. In an intrinsic protein fluorescence DSF experiment, emission wavelength of key residues change based on their chemical environment, which changes during refolding or unfolding processes. For F proteins, both the conformational steps associated with pre- to postfusion conformational conversion and denaturation and dissociation of the trimer would be likely to be detected by DSF. However, without any additonal information, changes in DSF signal could not clearly be assigned to a particular conformational event. By combining the concept of heat treatment, previously used to force conversion with PIV3 (37), with DSF, we were able to force conversion on command. By seeing elimination of early negative peaks only for wild-type proteins, we were able to confirm that this peak corresponds to conformational conversion. By applying this technique to the entire panel, we are able to link differences in sequence or structure to more definitive conclusions about metastability. In the case of AngV-F, the striking differences between its structure and those of other HNV-F proteins line up with the differences observed in its DSF profile. Clearly, AngV-F has greater pre-fusion state stability than other HNV-F proteins. The existence of a wider internal cavity may play a role in this, but more likely, the altered electrostatics and the introduction of a proline residue at the beginning of the stalk domain, also referred to as the HRB region, are responsible for this change. During conformational conversion in most HNV-F proteins, it is likely that the electropositive cluster works to disperse the helix bundle, serving to lower the energetic barrier to conformational conversion. Instead, in AngV-F, the absence of such electrostatic repulsion at this site along with the presence of a proline at the start of the HRB helix leads to a more stable prefusion HRB arrangement, which is likely why the AngV-F stalk domain is so well resolved in our cryo-EM structure.

DSF analysis of G head domains also reveals a diversity of themostability patterns. Although the G head domains constitute a single domain 6-stranded ß-propellar fold and are not known to have any major internal conformational rearrangements as part of the receptor-binding mechanism, some studies have indicated that receptor binding by HNV-G proteins could effect subtle changes in protein flexibility that don’t manifest as large-scale conformational events (48). The differences in thermostability could potentially correlate with mechanistic differences between strains, however, more structural data detailing the structural changes that take place after receptor binding in G proteins will be needed to confirm this. From our crystal structure of the Gamak virus head domain, we can observe other likely sources of mechanistic differences between species. Specifically, the presence of a glycan in the face of the beta propellor analagous to the binding site for the Ephrin receptor in NiV-G indicates that it is unlikely a proteinaceous receptor similar to EFNB2/B3 that binds at this side in GakV. Furthermore, the altered arrangement of the N- and C-terminal end of the head domain could suggest a different pattern for quaternary interactions in GakV-G ectodomain tetramers, different types of conformational changes within GakV-G after receptor binding, or it may even serve as a possible receptor binding site for an as of yet undiscovered receptor.

Ultimately, this broad characterization of HNV proteins provides a set of valuable biophysical and biochemical data for many species and strains that, to this point, had only been known as a sequence in a database. As these novel species are studied in more depth, we hope that our analysis can be used to provide context for the comparison of other key aspects of *Henipavirus* biochemistry, structure, and immunology.

## Methods

### Phylogenetic Analysis

Candidate sequences were identified by using the NCBI Protein BLAST tool (Ref). For F proteins, each of NP_112026.1, NP_047111.2, and *UUV47205.1* were used as input sequences. For G proteins, both of NP_112027.1and NP_047112.2 were input. A distance matrix was generated for all selected sequences by using the Clustal Omega tool (24). The final tree was built as a UPGMA based on the distance matrix using the Tree Viewer software (49).

### Protein Production

For HNV proteins, human codon-optimized DNA sequences were synthesized by GeneImmune Biotechnology and cloned into a pαH plasmid vector. Fusion protein ectodomain sequences (Supplemental Sequence Alignment F) were followed at the C-terminal end by a foldon trimerization domain, HRV3C protease site, 8x his tag, and a twin-strep tag (IBA Lifesciences) Attachment protein head domains (Supplemental Sequence Alignment G) had an artificial secretion signal, secrecon (40) added to the N-terminal end and an 8x his tag added to the C-terminal end. HNV protein plasmids were transiently transfected into HEK 293 cell lines, either Expi293 or 293F (Supplemental Table 1). The cells were allowed to incubate at 37°C, 8% CO_2_ and the supernatant was harvested 5 days post-transfection.

Fusion proteins were purified by first sequestering free biotin by applying BioLock blocking solution (IBA) and then using the Strep-Tactin affinity chromatography resin (IBA). Eluted protein was then further purified using a Superose 6 Increase 10/300 GL size-exclusion column (Cytiva) with PBS buffer at pH 8. The elution from SEC was spin concentrated with 100kDa filters and flash frozen with liquid nitrogen.

Attachment protein head domains were purified first by sequestering EDTA by adding 2mg of Cobalt Chloride per 1L of supernatant. TALON Cobalt Resin (Takara) was used for purification, with 20mM HEPES and 150mM NaCl at pH 8 as a buffer, adding 50mM Imidazole for elution. Proteins were further purified with a Superdex 75 Increase 10/300 GL SEC column (Cytiva), again using the HEPES-NaCl buffer. The elution from SEC was spin concentrated with 10kDa filters and flash frozen with liquid nitrogen.

Antibody human codon-optimized DNA sequences were synthesized by GeneImmune Biotechnology and cloned into pVRC8400 plasmid vectors. Heavy chains were designed to begin with the beginning of the antibody sequence and continue through the human IgG1 Fc region. Light chains included both the VL and CL domains. Depending on the sequence source, some antibody sequences either utilized mouse or human VL and VH domains. Mouse anti-NiV-F fabs were designed to end after the mouse CH1 domain and had an 8x his tag added to the C-terminal end, as described previously (36).

Plasmids encoding the heavy and light chains of the antibodies IgG 1E5, nAH1.3, 4G5, and DH851.3 were co-transfected into Expi293 cells at a 1:1 ratio, using the ExpiFectamine 293 Transfection Kit (Thermo Fisher Scientific, Cat. No. A14525), according to the manufacturer’s instructions. Following a six-day incubation period post-transfection, the cells were harvested, and the culture supernatant was clarified by filtration through a 0.2 µm PES membrane filter to remove cellular debris. IgG purification was subsequently carried out using Protein A affinity chromatography. The filtered supernatant was passed through the Protein A affinity column (Pierce^™^ Recombinant Protein A Agarose Cat No. 20334), and the bound IgG was eluted using IgG elution buffer by adjusting pH via Tris Base. Eluted fractions were pooled and concentrated with Centricon-70 30 kDA filter (Millipore Sigma) followed by size-exclusion chromatography using a HiLoad 16/600 Superdex 200pg column (Cytiva), pre-equilibrated with 1× phosphate-buffered saline (PBS, pH 7.4) containing 0.02% sodium azide to prevent microbial contamination. The purified IgG was collected, concentrated, and analyzed for purity and integrity by SDS-PAGE.

Anti-NiV-F fabs were purified using the same Cobalt resin-based procedure as described above for head domains.

To generate Fab fragments for 22F5 mouse IgG and 1C8 mouse IgG, 2 mg of each IgGs were passed through Zeba Spin Desalting Columns (ThermoFisher), then washed with digestion buffer containing 35 mg cysteine. IgGs were incubated with 0.250 ml of the 50% slurry of Immobilized Papain resin (ThermoFisher) followed by incubation with digestion buffer containing 35 mg cysteine at 37 °C overnight in microcentrifuge tubes. The digested Fabs were separated from the Immobilized Papain by centrifuging at 5000 x g for 1 minute, followed by washing with digestion buffer. After digestion, the mixture was subjected to protein A affinity chromatography (ThermoFisher) then monitored by SDS-PAGE to confirm Fab generation. To further purify the Fab fragments, Size-Exclusion Chromatography (SEC) was used for purification of the Fab fragments using Superose 6 Increase 10/300 column GL (Cytiva). The sample was loaded onto the column at a volume of 0.5 mL and the elution was carried out with PBS at a flow rate of 0.5 mL/min. Fractions were collected, yielding a final concentration of 3.2 mg/mL and 4.2 mg/ml for 22F5 Fab and 1C8 Fab, respectively. The purified Fab was stored at −80°C.

### DSF

Samples were analyzed using a Prometheus Panta (Nanotemper Technologies). All samples were diluted to 0.125mg/mL using PBS, pH 8. Roughly 10µL was added to glass capillaries in triplicate for measurements. The instrument was programmed to measure 350nm and 330nm fluorescence from 35.0–95.0°C using a 6.0°C/min temperature gradient. All curves reported represent averages from the triplicate readings.

### BLI

Bio-layer interferometry (BLI) was employed to assess the binding between fusion proteins and murine monoclonal antibodies (mAbs) or fragment antigen-binding regions (Fabs). The analysis was performed using an Octet RH16 (Octet RED384) system (Sartorius) at 25°C with kinetics buffer (Sartorius) as the running buffer. HNV-F proteins with the twin-strep tag at 6 µg/mL were captured onto Streptavidin (SA) Biosensors (Sartorius) for 300s, followed by a 60s wash in kinetics buffer. The biosensors were then exposed to 20 µg/mL mAbs or 50 µg/mL Fabs for 300s to allow for association with the fusion protein. Dissociation in kinetics buffer was monitored for an additional 300s. Sensor data was analyzed using Octet Analysis Studio (Sartorius), with reference biosensor and zero-analyte control sensorgrams subtracted from the raw data to obtain the curves corresponding to specific binding events.

### SPR

Binding and kinetic assays were perfomed by Surface Plasmon Resonance (SPR) on a T-200 Biacore system (Cytiva) at 25°C. All the binding and kinetic experiments were carried out in the HBS-EP+ (10 mM HEPES, pH 7.4, 150 mM NaCl, 3 mM EDTA and 0.05% surfactant P-20) running buffer. For the binding assay, anti-Fc mouse SPR chip were used to capture the 200nM of 22F5 IgG at 10ul/min flow rate and 50nM of LayV-1-F ectodomains as analyte were run at 30ul/min flow rate and association and dissociation were monitor at 120 seconds. In the competitive assay, 200nM of 4G5 IgG was captured on anti-Fc human SPR chip at 10ul/min and analyte as 22F5 Fab with LayV-1-F ectodomain complex in 5:1 ratio was flowed at 30ul/min flow rate for 60 seconds. SPR SA chip was used to capture the 50nM of the LayV-1-F ectodomains at 5ul/min flow rate by biotin tag for kinetics analysis. 22F5 Fab 50nM with two-fold dilution (50nM, 25nM, 12.5nM, 6.25nM and 3.12nM) as analyte was flowed with 50ul/min flow rate and association and dissociation time were analyzed at 60 and 240 seconds, respectively. For the reference curves, we kept the same parameter in reference flow cells without protein capture. The sensorgrams were blank corrected and analyzed in the Biacore T-200 evaluation software.

### Negative-Stain Electron Microscopy

Frozen samples from −80 °C were thawed at room temperature for 5 minutes. Samples were then diluted to 40 µg/mL with 0.02 g/dL Ruthenium Red in HBS (20 mM HEPES, 150 mM NaCl pH 7.4) buffer containing 8 mM glutaraldehyde. After 5-minute incubation, glutaraldehyde was quenched by adding sufficient 1M Tris stock, pH 7.4, to give 80 mM final Tris concentration and incubated for 5 minutes. Quenched sample was applied to a glow-discharged carbon-coated EM grid for 10–12 seconds, blotted, consecutively rinsed with 2 drops of 1/20X HBS, and stained with 2 g/dL uranyl formate for 1 minute, blotted and air-dried. Grids were examined on a Philips EM420 electron microscope operating at 120 kV and nominal magnification of 49,000x, and images were collected on a 76 Mpix CCD camera at 2.4 Å/pixel. Images were analyzed by 2D class averages using standard protocols with Relion 3.0 (50).

### Mass Photometry

A TwoMP (Refeyn) mass photometer was used for analysis. Calibrated system with a three-point curve consisting of β-amylase, Apoferritin, and Thyroglobulin. Samples were diluted to 40µM in PBS. To each well, 10µL of PBS was added to focus the system before adding 10µL of sample and mixing for a final concentration of 20µM. Recorded data for 60s, with the machine counting individual adsorption events. The TwoMP processing software (Refeyn) was used to assign molecular weights to individual events.

### Methods for Mouse Study #738

Immunization in VRC01ucadKI, hTdT HOM; HOM; het (SE13) mice

VRC01ucadKI, hTdT HOM; HOM; het (SE13) were intramuscularly (i.m.) immunized with LayV-F_WT (25 mcg/animal) and NiVop8 (25 mcg/animal) adjuvanted with GLA-SE (IDRI EM-082, 5 mcg/animal) at weeks 0, 4, 15 and 21. Serum titers were monitored by ELISA as described below. Mice with high-binding antibody titers were selected for the subsequent spleen cell fusion and B-cell sorting experiments.

### Hybridoma cell line generation and monoclonal antibody production

Mice were boosted with the indicated priming antigen 3 days prior to fusion. Spleen cells were harvested and fused with NS0 murine myeloma cells using PEG1500 to generate hybridomas. After 2 weeks, supernatant of hybridoma clones were collected and screened by binding ELISA as described below. Hybridomas that secreted LayV-F reactive antibodies were cloned by limiting dilution until the phenotypes of all limiting dilution wells were identical. IgG mAbs were purified by protein G purification.

### Indirect binding ELISA method

ELISA assays were utilized for the purposes of measuring mouse serum antibody titers, screening for antigen specific hybridoma clones and characterizing the binding of monoclonal antibodies. 384 well ELISA plates (Costar #3700) were coated with 2mcg/ml streptavidin (Thermo Fisher Scientific Inc. Cat. No. S-888) or protein antigen in 0.1M sodium bicarbonate overnight at 4°C. Plates were washed with PBS/0.1% Tween-20 and blocked for one hour with assay diluent (PBS containing 4%(w/v) whey protein/15% Normal Goat Serum/0.5% Tween-20/0.05% Sodium Azide). Streptavidin coated plates were washed and followed by 10μl Twin-Strep-tag^®^ (IBA Lifesciences GmbH) protein at 2mcg/ml in assay diluent for one hour. All plates were then washed and samples were added in 10μl volumes depending on sample type as follows. Mouse serum was diluted 1 to 30 and titrated three-fold. Hybridoma supernatants were added undiluted as a single well. Monoclonal Fabs and IgG were added starting at 100mcg/ml in three-fold titration. Plates were washed and 10µl goat anti-mouse IgG-HRP secondary antibody diluted 1:10,000 (Southern Biotech #1030–05) (for serum and hybridoma supernatants), goat anti-human IgG Fab-HRP diluted 1:15,000 (Jackson Immunoresearch, 109-035-097 ) or goat anti-Mouse IgG Fab-HRP diluted 1:10,000 (Southern Biotech 1015–05) (for monoclonal Fabs and IgG) in assay diluent without azide was incubated for 1 hour, washed again and detected with 20µl SureBlue Reserve (Seracare 5120–0081) for 15 minutes. Reaction was stopped with the addition of 20µl HCL stop solution. Plates were read at 450nm.

### Cryo-EM grid Preparation and Imaging

To prepare grids, UltaFoil R1.2/1.3 (Cu, 300-mesh; Electron Microscopy Sciences, PA) grids were glow discharged for 15 seconds at 15 mA using a PELCO easiGlow Cleaning System (Ted Pella Inc). The final concentration of purified AngV-F protein was maintained as 1.5 mg/ml in PBS. 0.005 % (w/v) of n-dodecyl β-D-maltoside (DDM) was added to the protein samples before applying to the grid to prevent the protein form adhering to the air-water interface. 3.0 μL of the protein sample was applied to the grid and incubated for 30 seconds at >95% humidity. Excess protein was blotted away for 2.5 seconds before being plunge frozen into liquid ethane using Leica EM GP2 plunge freezer (Leica Microsystems). Cryo-grids were imaged using an FEI Titan Krios (Thermo Scientific) equipped with K3 camera (Gatan) at 81k magnification, operated at 300kV. Approximately 24,000 micrographs were collected at nominal defocused range between −2.8 to −1.7 μM with dose range of 63–65 e/A^2^.

#### Cryo-EM Data Processing:

Cryo-EM image quality was monitored on-the-fly during data collection using automated processing routines. Data processing was carried out using cryoSPARC (51, 52). Micrographs were curated through contrast transfer function (CTF) where greater than >30 Å were discarded. Automated blob picker was used to assign the particle position. Different box sizes are utilized to accommodate the varying dimensions of specific molecular assemblies. For instance, monomer particles are extracted using a box size of 320 pixels, while dimer particles require a larger box size of 512 pixels. For even larger molecular assemblies, such as hexamer lattices, a box size of 1024 pixels was employed. Following particle extraction, multiple rounds of 2D classification was performed to remove junk. *ab-initio* 3D reconstruction was used to create 3D reconstructions and poor-quality particles were discarded after through heterogenous refinement. Final resulting volumes were subjected to non-uniform refinement to build a high-resolution 3D reconstruction. Phenix (53), Coot (54), Pymol, and ChimeraX (55) were used for model building and refinement.

### X-ray crystallography.

Crystals were grown via the vapor diffusion method at room temperature in siting drop well format. GakV-2-G head domain protein crystals were formed by mixing 0.20 μL of protein at 15 mg/mL with 0.20 μL of well solution containing 0.2 M magnesium sulfate heptahydrate, 20% w/v PEG3350 at pH 7.1. Crystals appeared within one week.

Cryoprotection of the crystals was done by mixing glycerol to a final concentration of 50% with the well solution. Crystals were moved to the drop containing the well solution and glycerol and soaked for one minute then flash-cooled in liquid nitrogen and data were collected at the NSLS-II, Beamline 19-ID at 100 K and a wavelength of 1 Å. All data were indexed and integrated using iMosflm (56) and scaled using AIMLESS.

Phaser (57) in the PHENIX suite was used to perform molecular replacement using a model generated from the AlphaFold3 prediction server (58) using the GakV-2-G head domain sequence. Phenix.refine (59) was used for data refinement, and manual refinement was done in Coot (54).

## Supplementary Material

Supplement 1

Supplement 2

Supplement 3

Supplement 4

Supplement 5

Supplement 6
