# Structural and antigenic characterization of novel and diverse Henipavirus glycoproteins

**DOI:** 10.1101/2024.12.11.627382

**Published:** 2025-04-10

**Authors:** Aaron J May, Muralikrishna Lella, Jared Lindenberger, Alex Berkman, Ujjwal Kumar, Moumita Dutta, Maggie Barr, Rob Parks, Xiaozhi Lu, Madison Berry, Amanda Newman, Xiao Huang, Kijun Song, Victor Ilevbare, Salam Sammour, Chan Soo Park, Radha Devkota Adhikari, Priyanka Devkota, Katarzyna Janowska, Yanshun Liu, Garrett Scapellato, Taylor N Spence, Katayoun Mansouri, Kevin Wiehe, Robert J Edwards, Kevin O'Neil Saunders, Barton F Haynes, Priyamvada Acharya

## Abstract

Henipaviruses, a genus within the Paramyxoviridae family, include the highly virulent Nipah and Hendra viruses that cause reoccurring outbreaks of deadly disease. Recent discoveries of several new Paramyxoviridae species, including the zoonotic Langya virus, have revealed much higher antigenic diversity than currently characterized and prompted the reorganization of these viruses into the Henipavirus and Parahenipavirus genera. Here, to explore the limits of structural and antigenic variation in both genera, collectively referred to here as HNVs, we constructed an expanded, antigenically diverse panel of HNV fusion and attachment glycoproteins from 56 unique HNV strains that better reflects global HNV diversity. We expressed and purified the fusion protein ectodomains and the attachment protein head domains and characterized their biochemical, biophysical and structural properties. We performed immunization experiments in mice leading to the elicitation of antibodies reactive to multiple HNV fusion proteins. Cryo-electron microscopy structures of diverse fusion proteins elucidated molecular determinants of differential pre-fusion state metastability and higher order contacts. A crystal structure of the Gamak virus attachment head domain revealed an additional domain added to the conserved 6-bladed, β-propeller fold. Taken together, these studies expand the known structural and antigenic limits of the HNVs, reveal new cross-reactive epitopes within both genera and provide foundational data for the development of broadly reactive countermeasures.

## Full Text Availability

The license terms selected by the author(s) for this preprint version do not permit archiving in PMC. The full text is available from the preprint server.

