# Supplementary material for: Structural and Antigenic Characterization of Novel and Diverse Henipavirus Glycoproteins
Source: bioRxiv. 2025 Feb 25:2024.12.11.627382. Originally published 2024 Dec 11. Preprint. [Version 3] doi: 10.1101/2024.12.11.627382 (PMC11661166; doi:10.1101/2024.12.11.627382)
Supplement: Supplementary file 1 [file media-1.pdf]

Supplemental Material For:

Structural and Antigenic Characterization of Novel and Diverse *Henipavirus* Glycoproteins

Authors:

Aaron J. May[1,2,*], Muralikrishna Lella[1,#], Jared Lindenberger[1,#], Alex Berkman[1,#], Ujjwal Kumar[1], Moumita Dutta[1], Maggie Barr[1], Rob Parks[1], Amanda Newman[1], Xiao Huang[1], Kijun Song[1,2], Victor Ilevbare[1], Salam Sammour[1], Chan Soo Park[1], Radha Devkota Adhikari[1], Priyanka Devkota[1], Katarzyna Janowska[1], Yanshun Liu[1], Garrett Scapellato[1], Taylor N. Spence[1], Katayoun Mansouri[1], Robert J Edwards[1,3], Kevin O. Saunders[1,4,5], Barton F. Haynes[1,3,4], Priyamvada Acharya[1,2,5,*,$]

Affiliations:

[1] Duke University, Duke Human Vaccine Institute, Durham NC 27710, USA

[2] Duke University, Department of Biochemistry, Durham NC 27710, USA

[3] Duke University, Department of Medicine, Durham, NC 27710, USA

[4] Duke University, Department of Integrative Immunology, Durham NC 27710, USA

[5] Duke University, Department of Surgery, Durham NC 27710, USA

[$]Lead contact

#These authors contributed equally

[*]Correspondence: aaron.may@duke.edu (A.M.), priyamvada.acharya@duke.edu (P.A.)

This PDF File Includes:

Supplemental Figures 1-14

Supplemental Tables 2-3

Supplemental Data 1-2

Note: Supplemental Table 1 provided as a separate excel file.

Supporting Material Contents

Content **Page(s)**

Supplemental Figure 1: Amino Acid-based Phylogeny of HNV Species	3
Supplemental Figure 2: Size Exclusion Chromatography (SEC) Profiles of HNV-F Ectodomains	4-7
Supplemental Figure 3: SDS-PAGE Profiles of HNV-F Ectodomains	8
Supplemental Figure 4: NSEM Analysis of HNV-F Ectodomains	9-12
Supplemental Figure 5: Measurement of Antibody Binding to HNV-F Proteins using Biolayer Interferometry (BLI)	13-14
Supplemental Figure 7: Cryo-EM Data Processing Workflow for LayV-1-F Ectodomain and 22F5 Fab Complex	15
Supplemental Figure 8: SPR Analysis of 22F5 Binding to LayV-1-F Ectodomain	16
Supplemental Figure 9: DSF-relevant Residue Variability	17
Supplemental Figure 10: Workflow of Particle Extraction and 2D Classification	18
Supplemental Figure 11: Cryo-EM Data Processing and Refinement Outline of Monomer Pre-fusion AngV-F Protein	19
Supplemental Figure 12: Oligomeric States of AngV-F and HeV-α2-F	20
Supplemental Figure 13: AngV-F Glycan Distribution and Differences Compared to other HNV-F Proteins	21
Supplemental Figure 14: Measurement of Antibody Binding to HNV-G Dead Domains using ELISA	22
Supplemental Table 2. Cryo-Em Data Collection and Refinement Statistics	23
Supplemental Table 3. Data Collection and Refinement Statistics (Molecular Replacement)	24
Supplemental Data 1: Sequence Alignment of HNV-F Proteins	25-28
Supplemental Data 2: Sequence Alignment of HNV-G Proteins	29-33

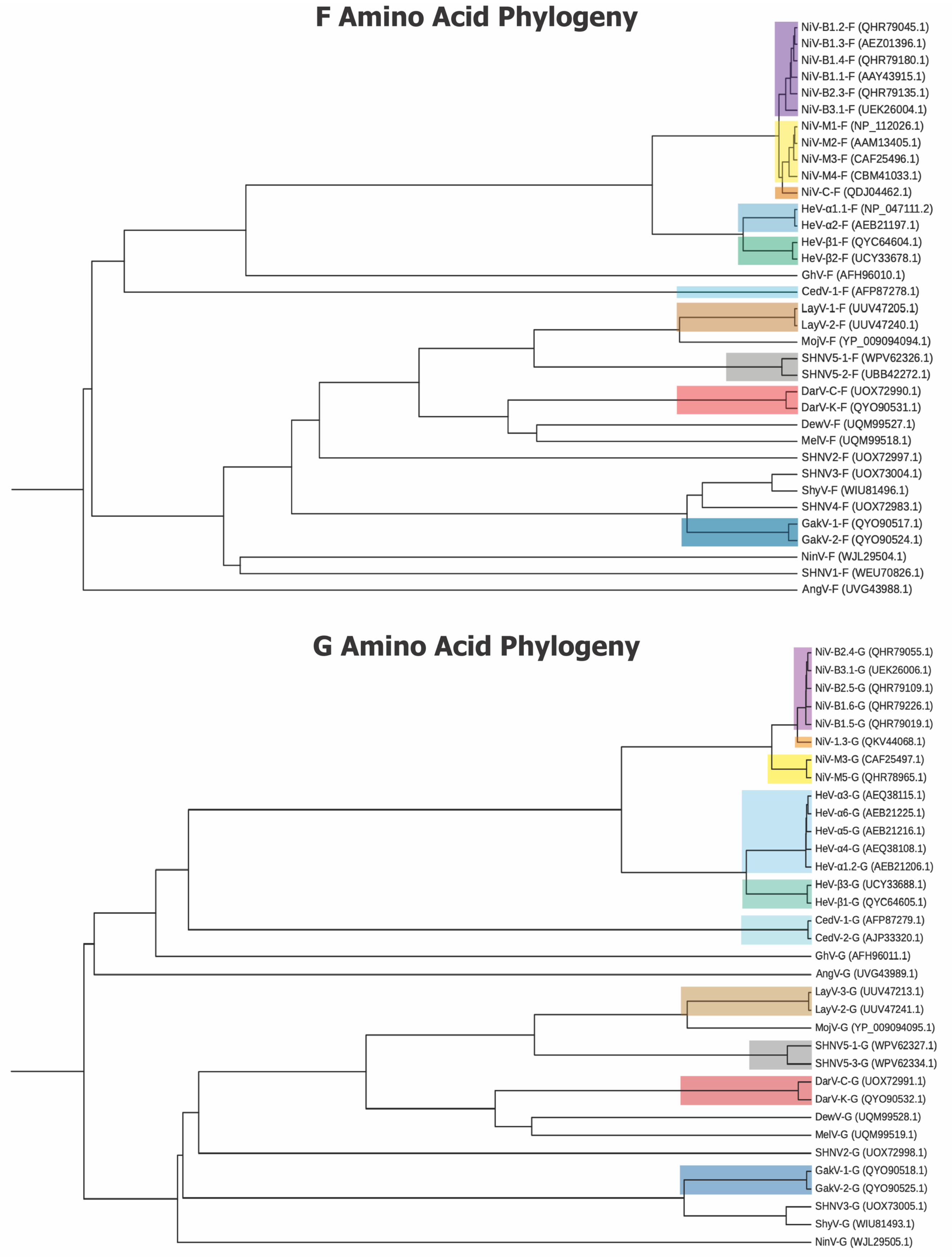

Supplemental Figure 1: Amino Acid-based Phylogeny of HNV Species. Phylogenetic trees constructed using either F or G amino acid sequences. Genbank amino acid accession codes listed next to each tip. Color scheme is continued from Figure 1.

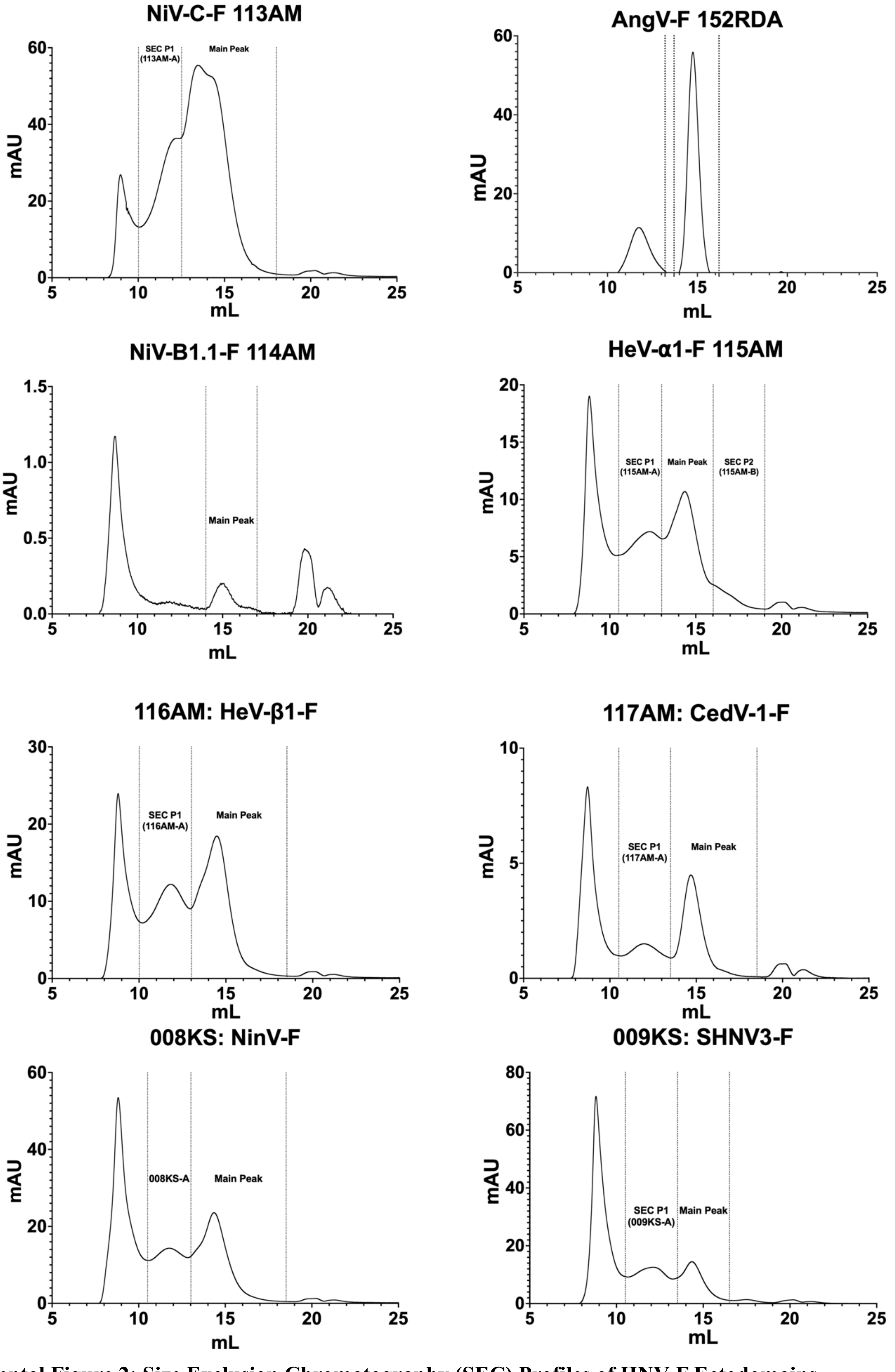

Supplemental Figure 2: Size Exclusion Chromatography (SEC) Profiles of HNV-F Ectodomains.

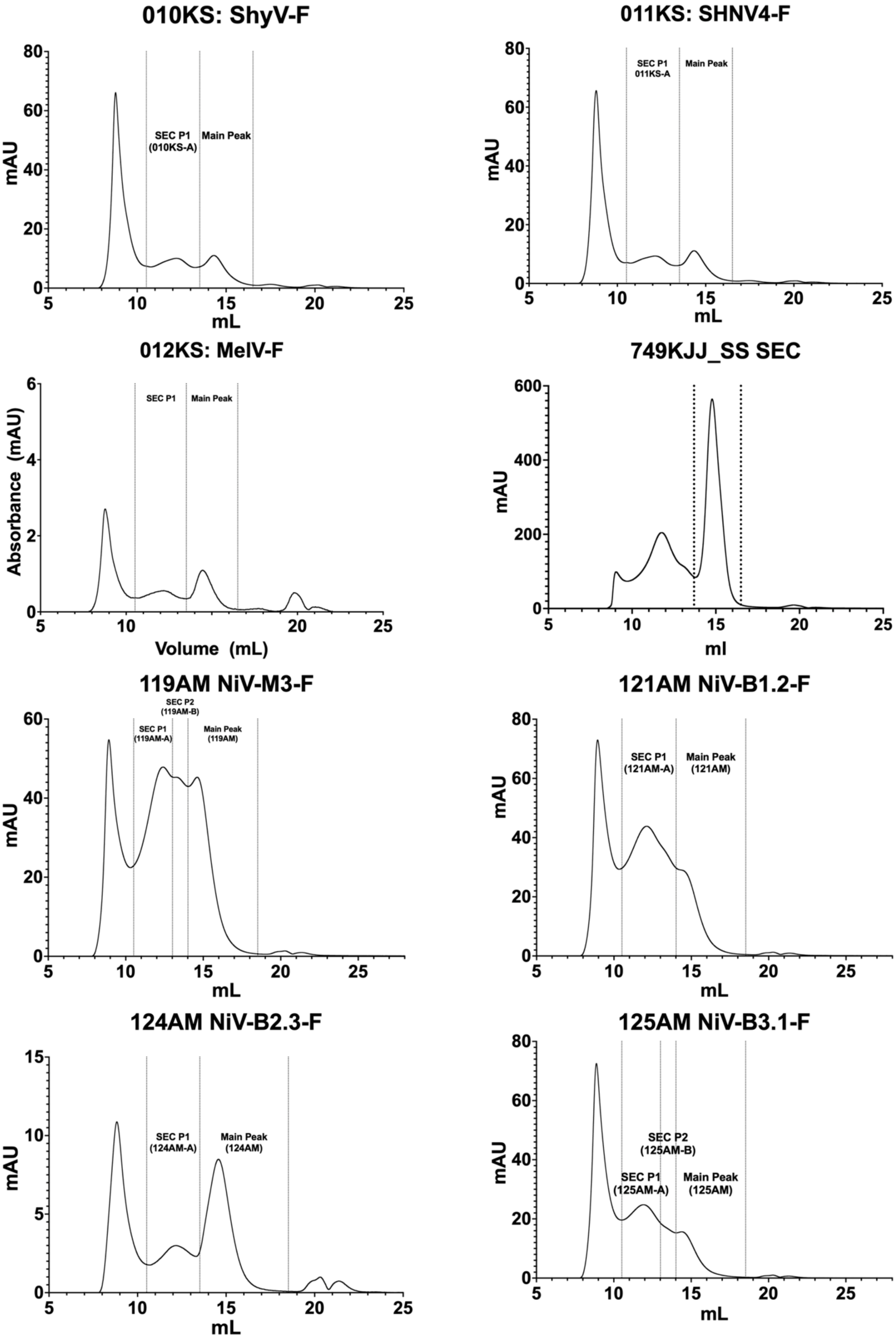

Supplemental Figure 2 (continued): Size Exclusion Chromatography (SEC) profiles of HNV F ectodomains.

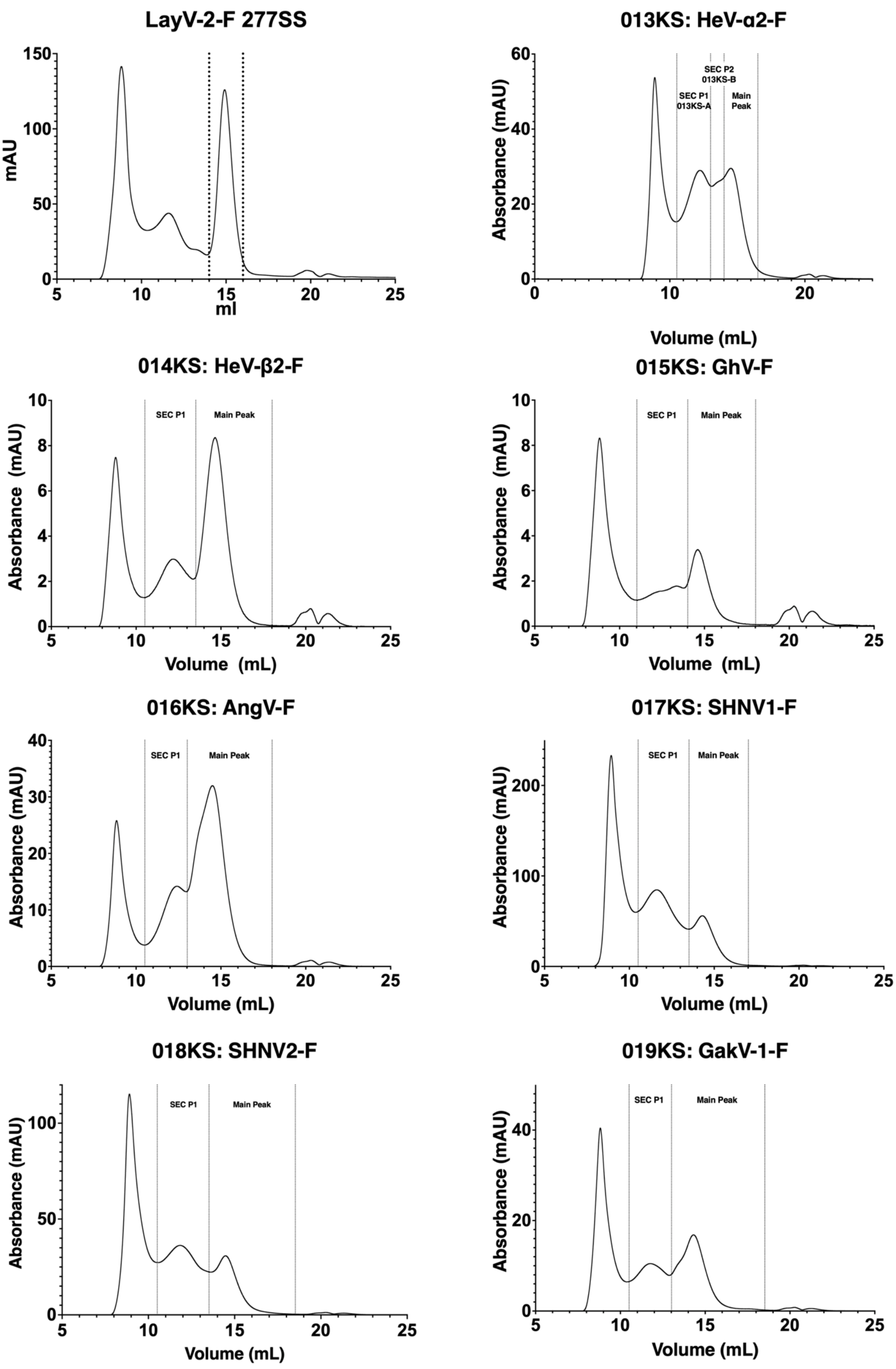

Supplemental Figure 2 (continued): Size Exclusion Chromatography (SEC) Profiles of HNV-F Ectodomains.

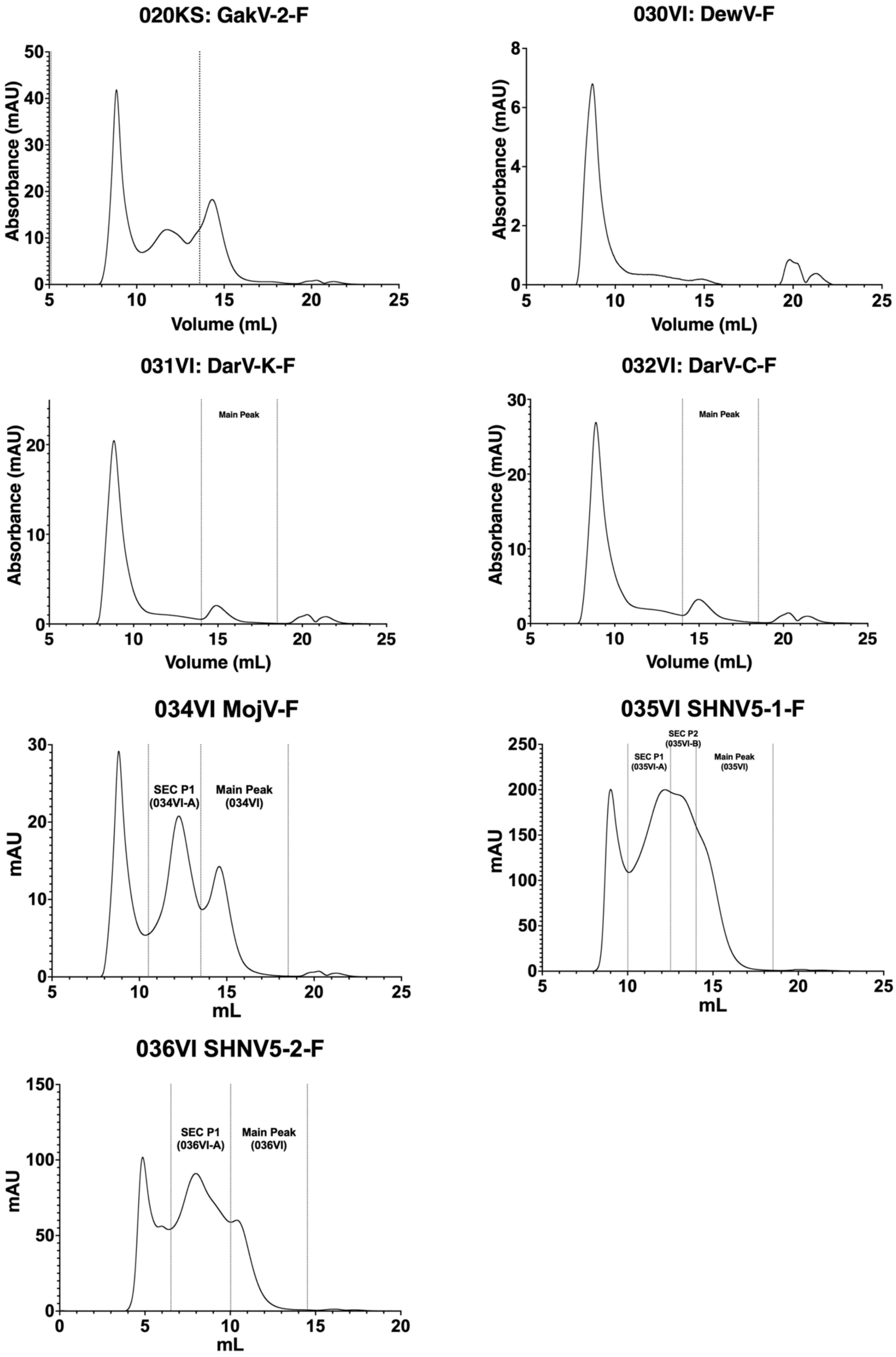

Supplemental Figure 2 (continued): Size Exclusion Chromatography (SEC) Profiles of HNV-F Ectodomains.

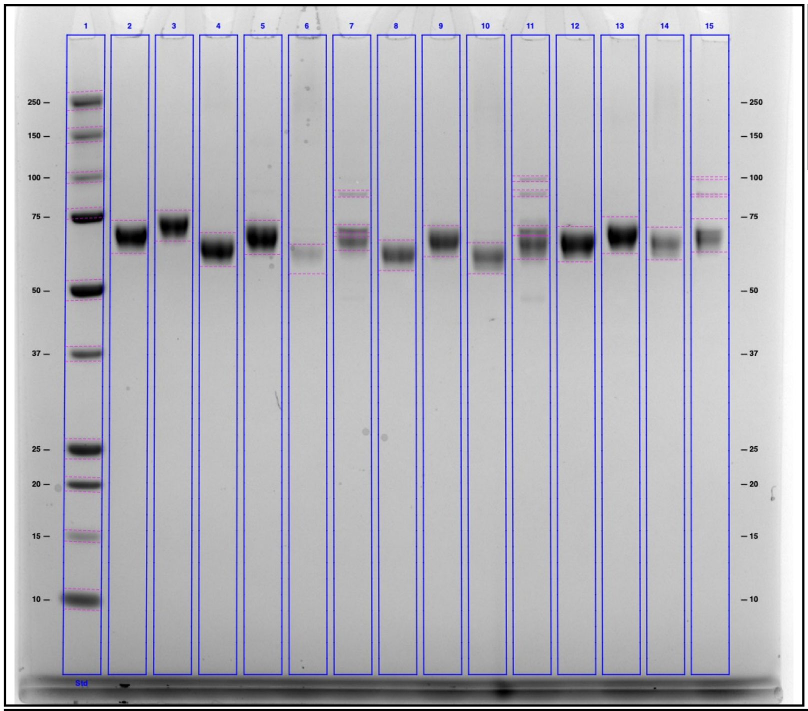

Protocol: 15 µL of each sample was loaded both reduced and non-reduced. The sample was boiled @ 95˚C for 3 min.
DTT: 1M DTT
Protein Marker: Precision Plus Protein Unstained Standards
Sample Buffer: 4X Laemmli Sample buffer
Gel: NuPAGE 4-12% BT 1.0, 15-well combs

Lane	Sample Name	Amount Loaded (µg)	Volume Loaded (µL)	Reduced (R/NR)	Molecular Weight (kDa)
1	Ladder	N/A	4	N/A	N/A
2	117AM: CedV-1-F	1	15	NR	67.3
3	117AM: CedV-1-F	1	15	R	71.5
4	123AM: NiV-B1.4-F	0.68	15	NR	62.4
5	123AM: NiV-B1.4-F	0.68	15	R	66.8
6	123AM-A: NiV-B1.4-F SEC P1	1	15	NR	61.8
7	123AM-A: NiV-B1.4-F SEC P1	1	15	R	88.9, 69.5, 65.8
8	124AM: NiV-B2.3-F	1	15	NR	60.8
9	124AM: NiV-B2.3-F	1	15	R	65.5
10	124AM-A: NiV-B2.3-F SEC P1	0.81	15	NR	60.3
11	124AM-A: NiV-B2.3-F SEC P1	0.81	15	R	99.2, 88.9, 68.9, 64.7
12	014KS: HeV-β2-F	1	15	NR	64.7
13	014KS: HeV-β2-F	1	15	R	67.5
14	014KS-A: HeV-β2-F SEC P1	1	15	NR	64.9
15	014KS-A: HeV-β2-F SEC P1	1	15	R	98.9, 88.3, 67.1

Notes: NR: 3.75 µL Dye, Protein, PBS
R: 3.75 µL dye, 4.5 µL DTT , Protein, PBS

Supplemental Figure 3: SDS-PAGE Profiles of HNV-F Ectodomains. Representative example of an SDS-PAGE profile shown for HNV F ectodomains. Lane 2 and 3: CedV-1-F (NR= Non-reduced; R= Reduced). Lanes 4 and 5: Main (trimer) peak of NiV-B1.4-F. Lanes 6 and 7: Aggregate of trimers (Middle peak; P1) of NiV-B1.4-F. Lanes 8-11: NiV-B2.3-F, same order of samples as for NiV-B1.4-F. Lanes 12-15: Hev-ß2-F, same order of samples as for NiV-B1.4-F.

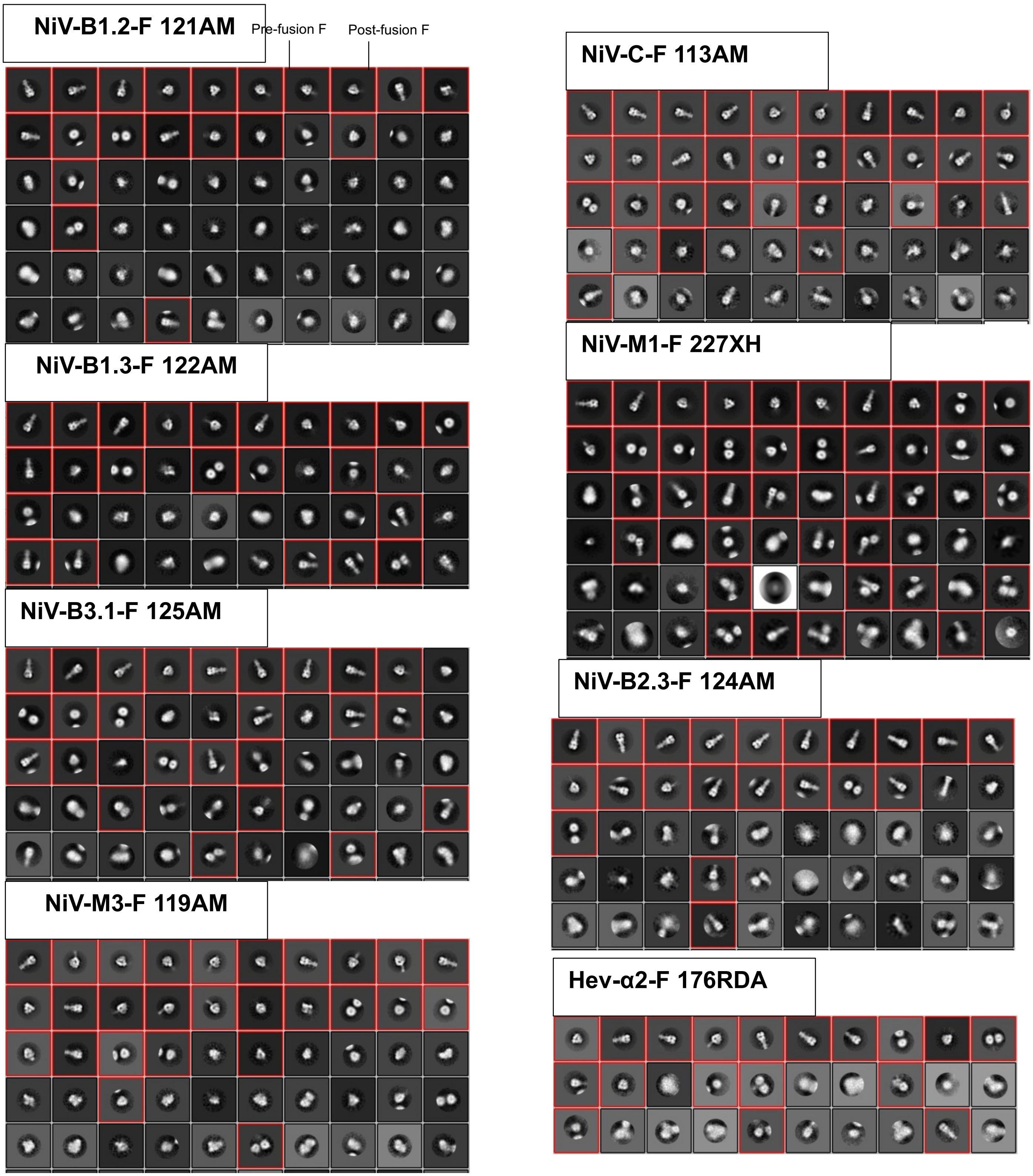

Supplemental Figure 4: NSEM Analysis of HNV-F Ectodomains. Representative 2D classes are shown. Classes containing the pre-fusion or post-fusion F protein can be identified by their shapes (as shown for NiV-B1.2-F as representative examples). The classes that correspond to the F proteins are identified by their typical shape and distinguishable from junk classes.

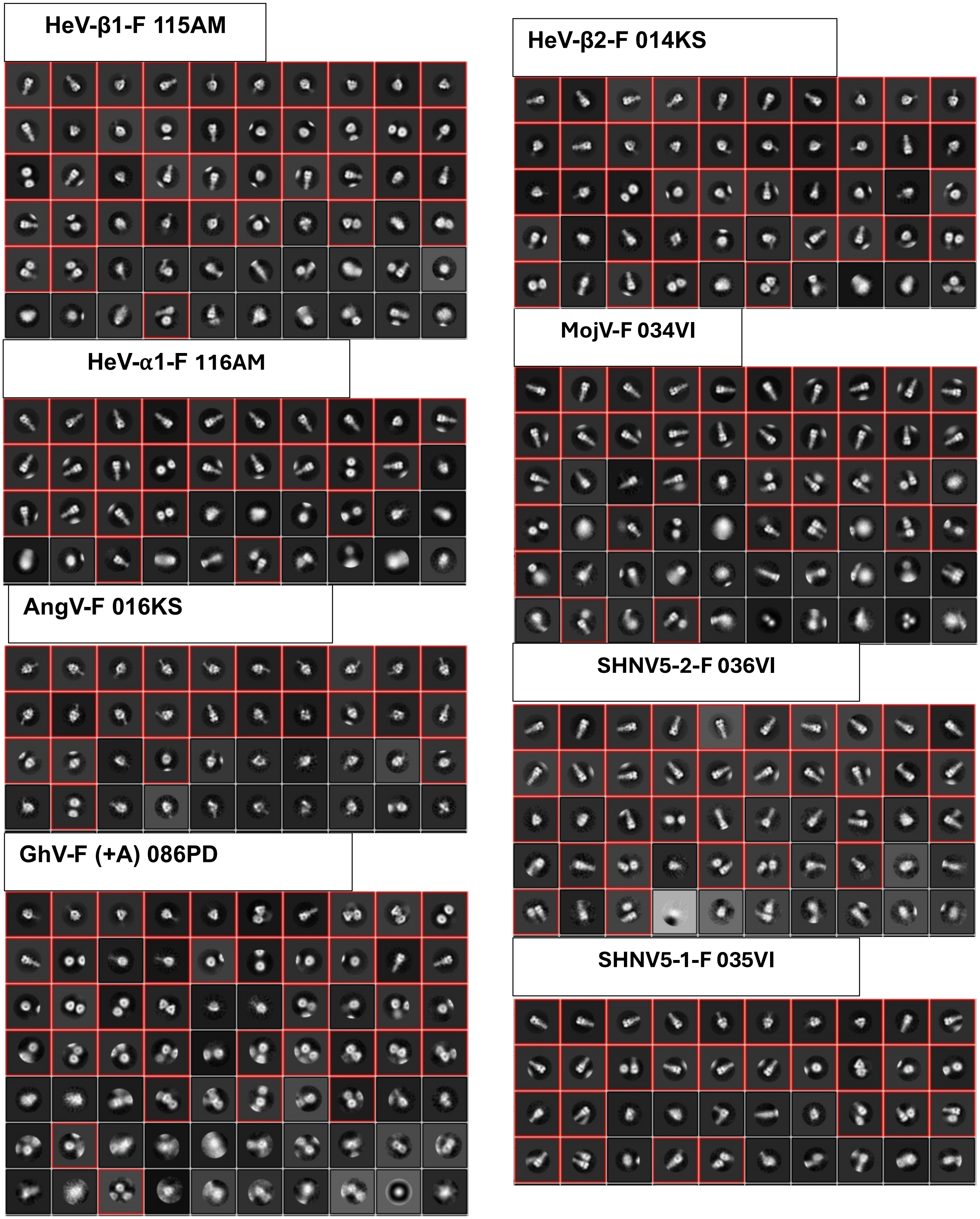

Supplemental Figure 4 (continued): NSEM Analysis of HNV-F Ectodomains. Representative 2D classes are shown.

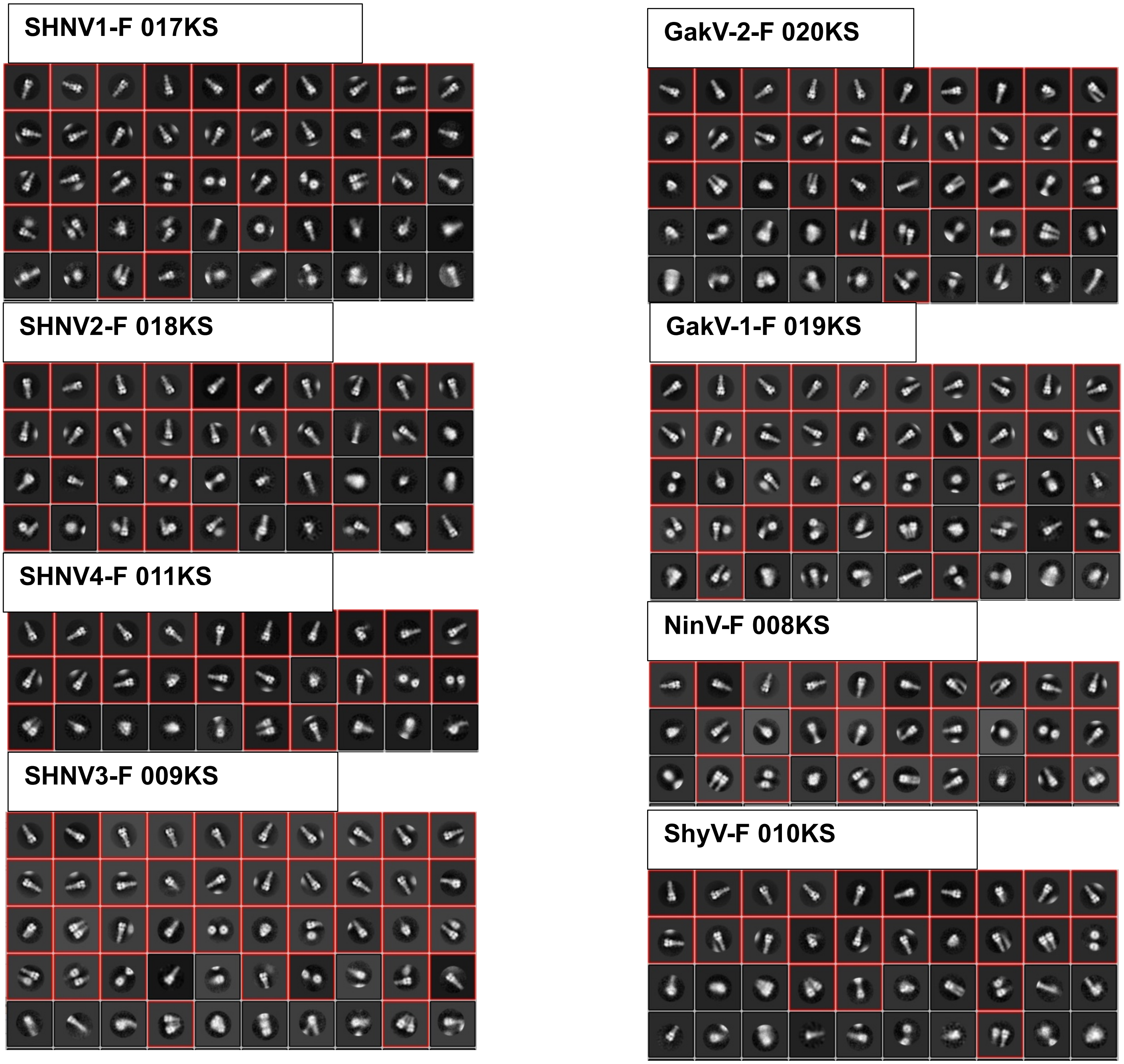

Supplemental Figure 4 (continued): NSEM Analysis of HNV-F Ectodomains. Representative 2D classes are shown.

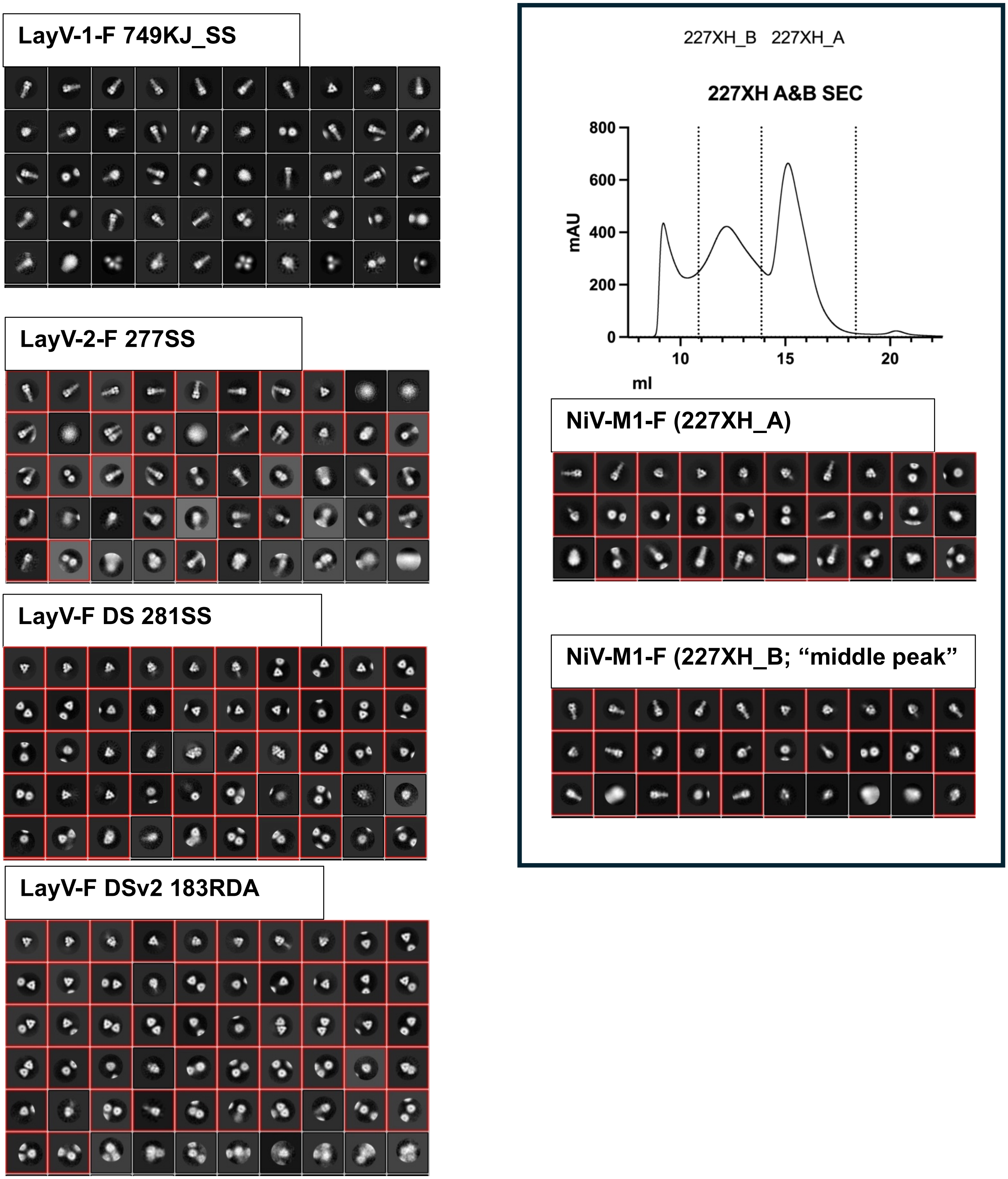

Supplemental Figure 4 (continued): NSEM Analysis of HNV-F Ectodomains. Representative 2D classes are shown. Right panel shows SEC profile of a NiV-M1-F ectodomain preparation with panels below showing representative NSEM 2D classes from the two peaks indicated by vertical dotted lines in the SEC profile.

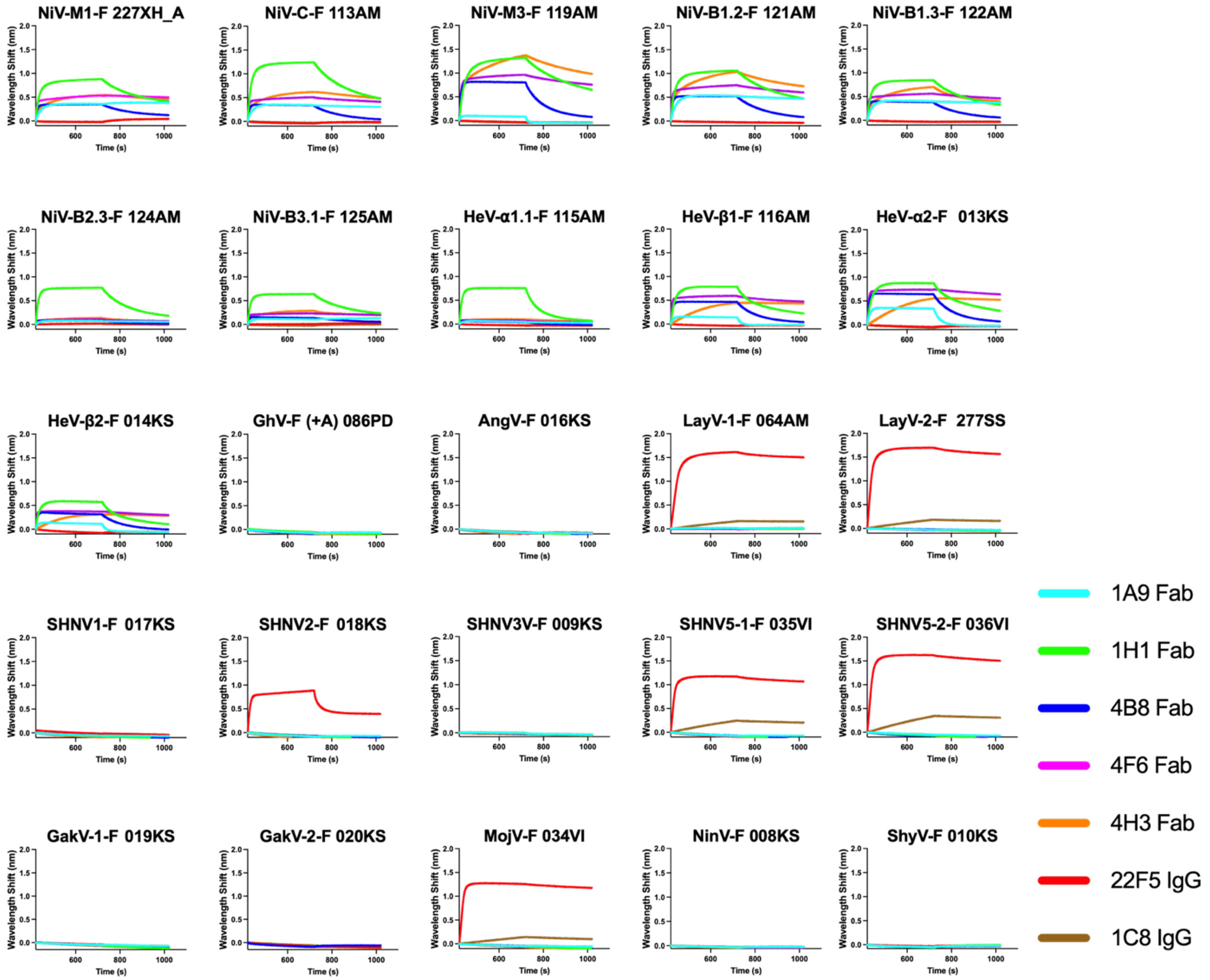

Supplemental Figure 5: Measurement of Antibody Binding to HNV-F Proteins using Biolayer Interferometry (BLI). The F proteins were captured on a BLI sensor tips via their C terminal TwinStrep tags. Binding to Fabs (1A9, 1H1, 4B8, 4F6, 4H3) or the vaccine elicited antibodies (22F5, 1C8) were measured. For each graph the virus from which the F ectodomain is derived is indicated at the top along with the lot number of the F ectodomain preparation.

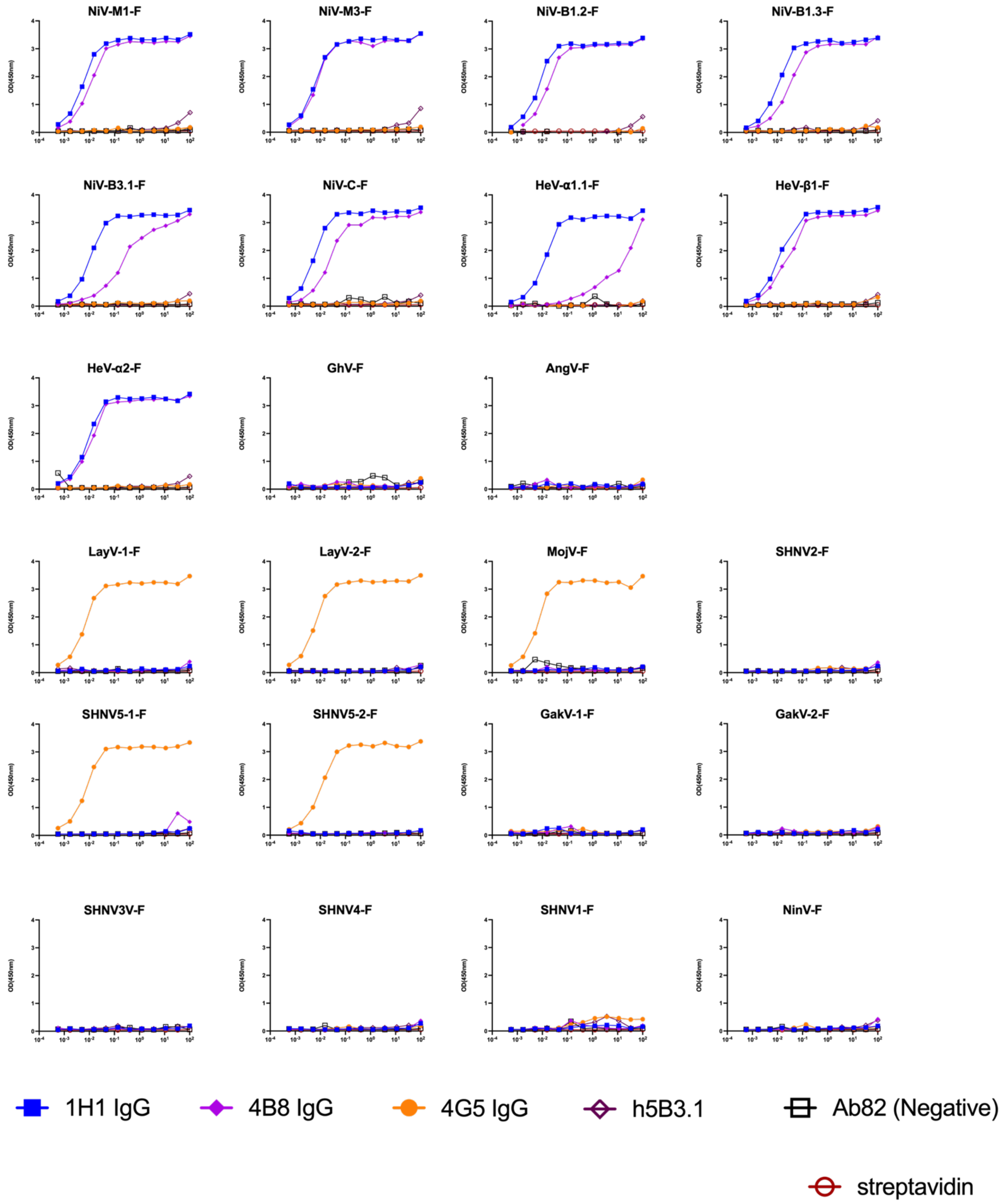

Supplemental Figure 6: Measurement of Antibody Binding to HNV-F Proteins using ELISA.

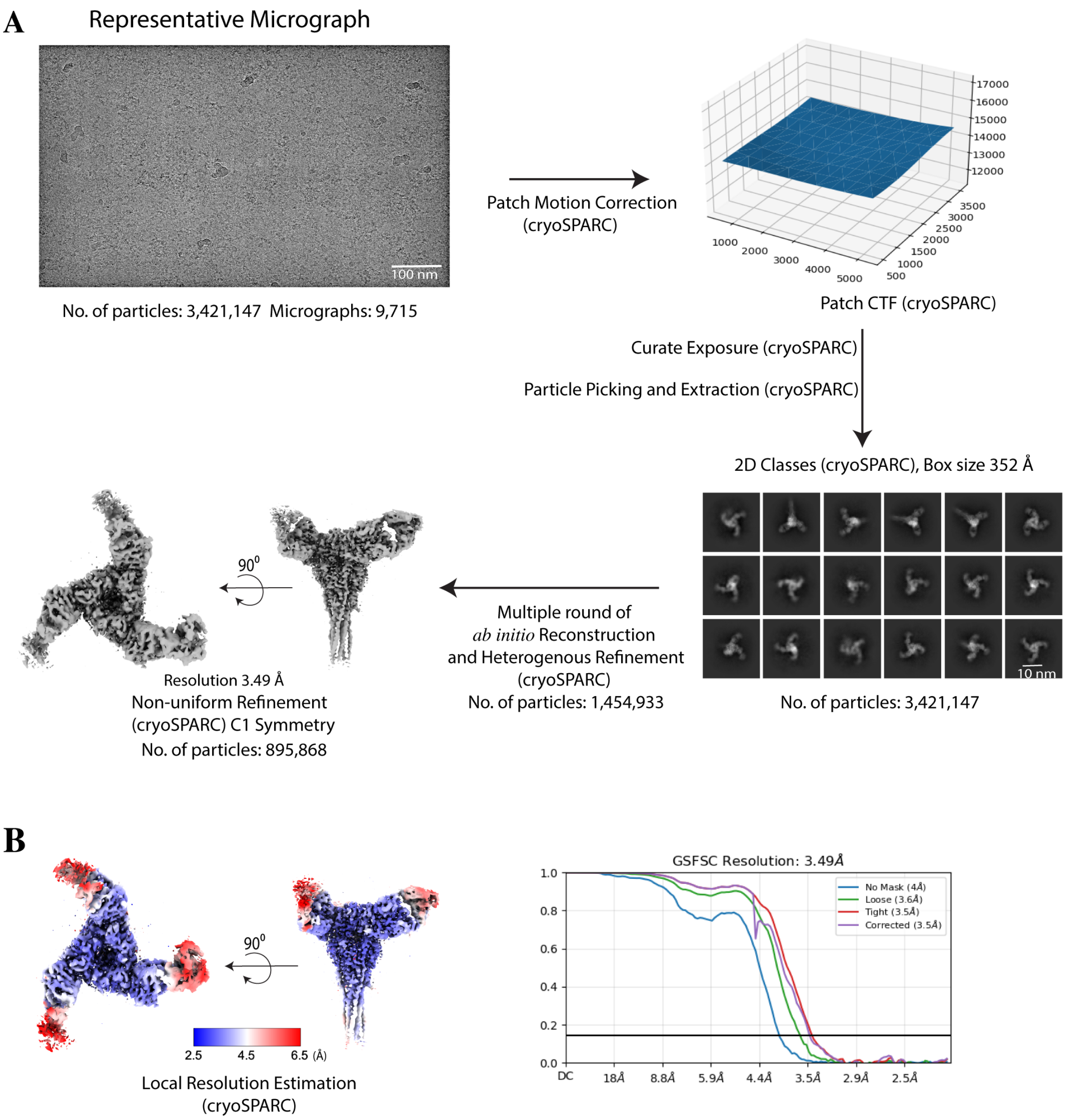

Supplemental Figure 7: Cryo-EM Data Processing Workflow for LayV-1-F Ectodomain and 22F5 Fab Complex. A. Representative cryo-EM micrograph of the LayV-1-F with 22F5 Fab complex after motion correction within cryoSPARC. Patch CTF estimation was performed by exposure curation, particle picking (Blob Picker) and particle extraction. Multiple rounds of 2D classification were performed. A clean stack of particles was used for *ab initio* 3D reconstruction to generate the volume map. To refine further and improve the volume map, selected particles were processed multiple times through heterogeneous refinement and followed by final high resolution 3D reconstruction via non-uniform refinement. Side and top views of final refined map are shown. **B**. Local resolutions were estimated and represented in the color spectrum which indicates high resolution as blue and low resolution as red. The complex was refined using C1 symmetry. Fourier Shell Correlation (FSC) curve from the gold standard refinement of the final map is shown.

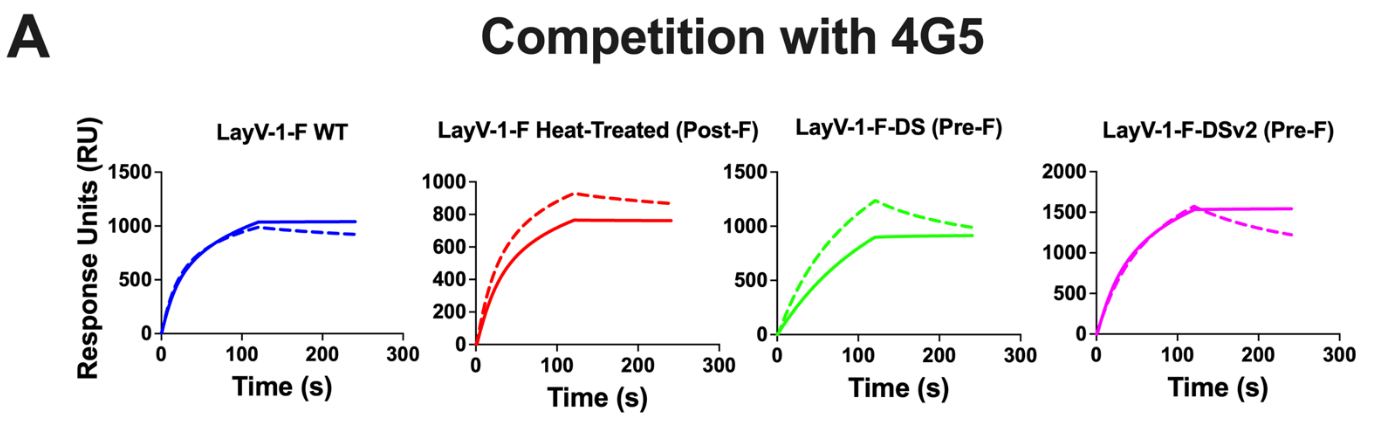

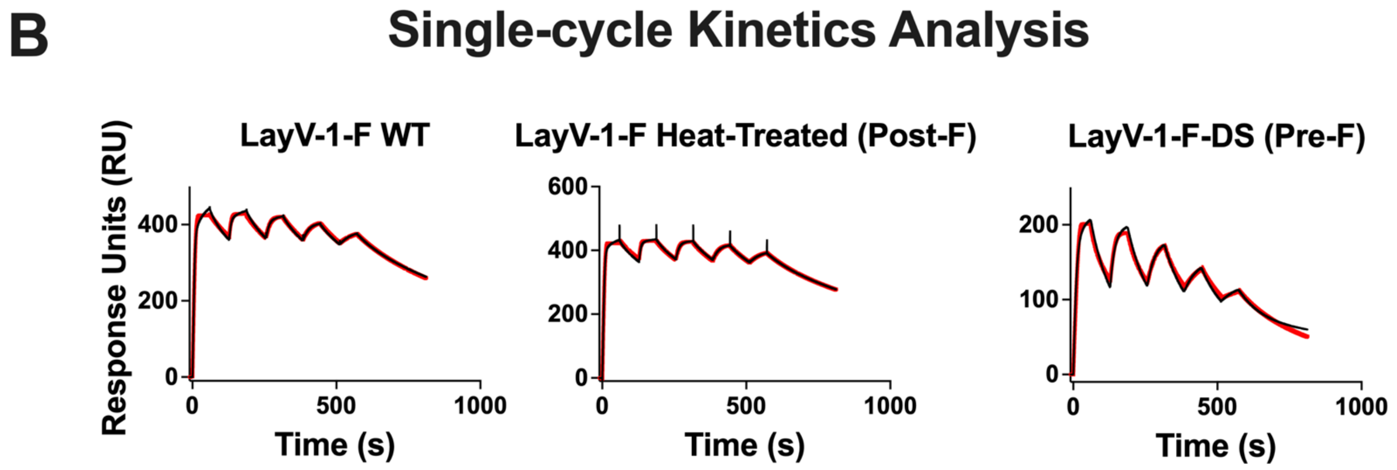

	LayV-1-F WT	LayV-1-F Heat-Treated (Post-F)	LayV-F-DS (Post-F)
ka (1/Ms)	7.90E+06	1.10E+07	7.74E+06
kd (1/s)	0.002806	0.002879	0.01923
KD (M)	3.55E-10	2.62E-10	2.49E-09

Supplemental Figure 8: SPR Analysis of 22F5 Binding to LayV-1-F Ectodomain. A. Competition assay of 22F5 vs 4G5, with 4G5 IgG captured on sensor chip and LayV-F ectodomains injected as analyte either with (dashed lines) or without (solid lines) 4G5 Fab. **B.** Measurement of affinity and kinetics of binding of 22F5 Fab to LayV-F ectodomains. The black lines in the graphs are the blank subtracted sensorgrams and the red lines are the fit of the data to a 1:1 Langmuir binding model. The table below shows the affinity and kinetic parameters obtained from the measurements.

F Protein W and Y Variability

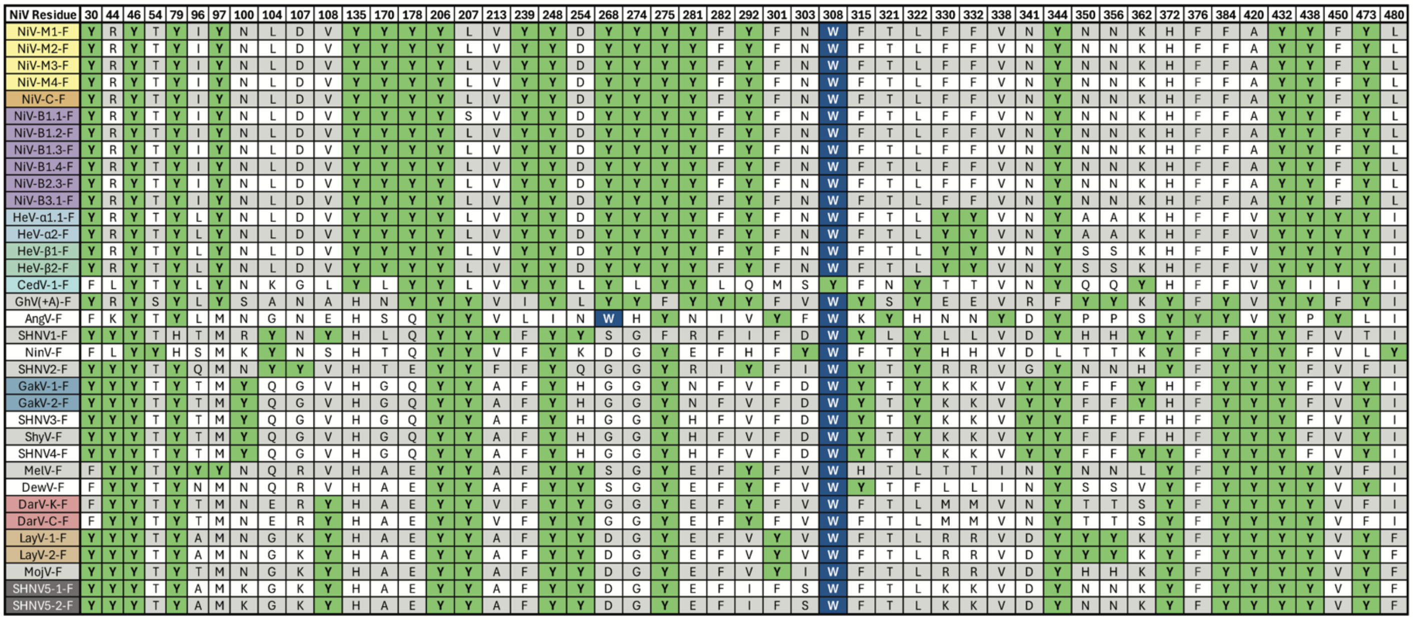

G Head Domain W and Y Variability

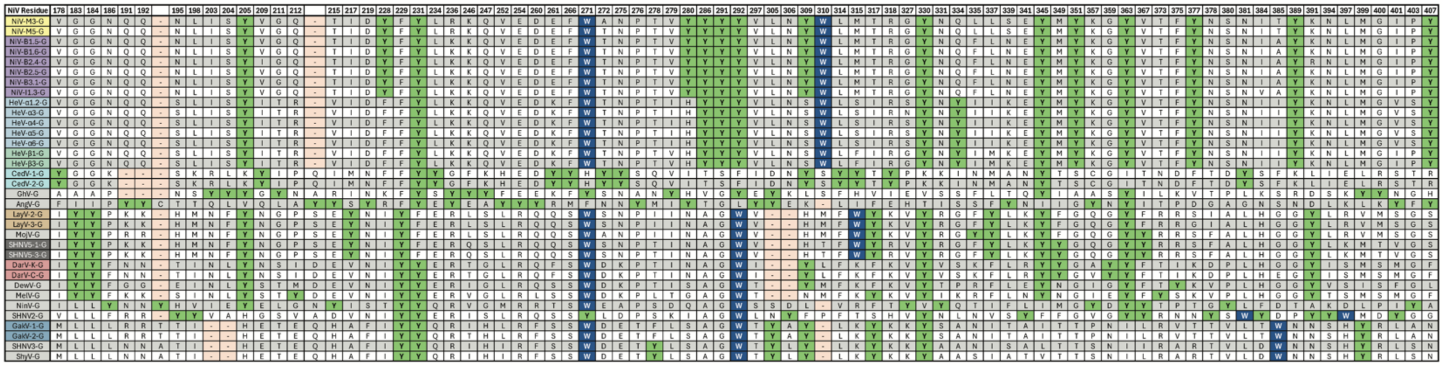

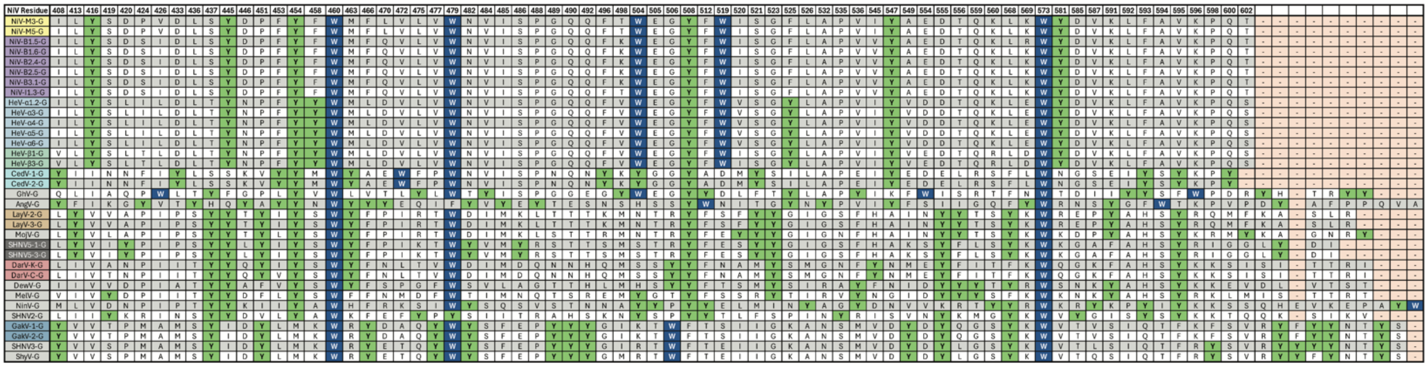

Supplemental Figure 9: DSF-relevant Residue Variability. Sequence alignment and highlighting of DSF-relevant tryptophan, blue, and tyrosine, green, residues. Gaps in the alignment are colored salmon. Species color scheme continued from Figure 1.

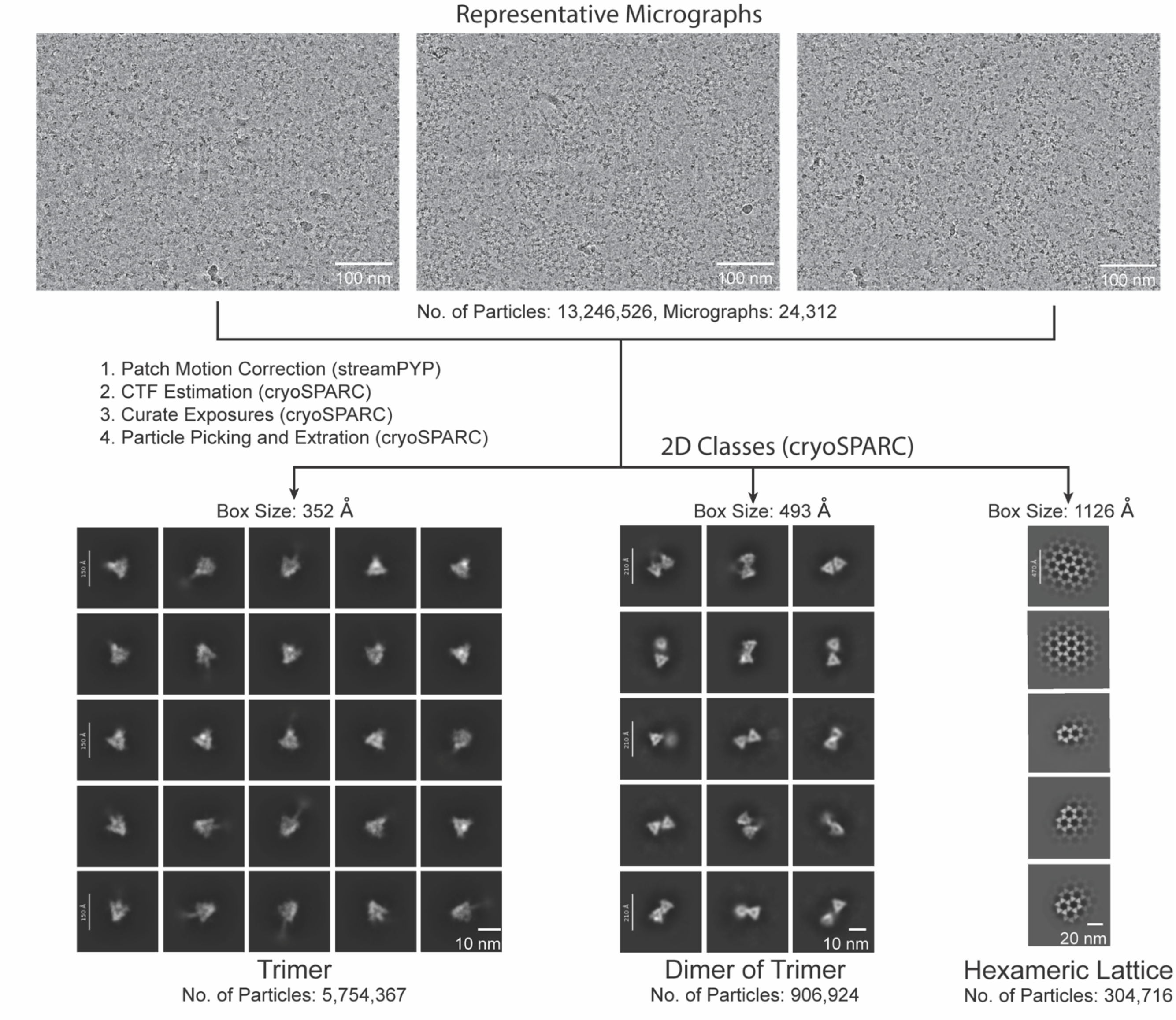

Supplemental Figure 10: Workflow of Particle Extraction and 2D Classification. Representative cryo-EM micrographs of the AngV-F protein after motion correction by nextPYP. Particles were picked and extracted after curating micrographs through CTF estimation. Different box sizes are utilized to accommodate the varying dimensions of specific molecular assemblies. Trimer particles are extracted using a box size of 320 pixels, while dimer of trimer particles require a larger box size of 512 pixels. For even larger molecular assemblies, such as hexameric lattices, a box size of 1024 pixels is employed. These extracted particles are then subjected to 2D classification to group and refine them based on their structural features, ensuring accurate downstream analysis (Supplemental Figures 11-12).

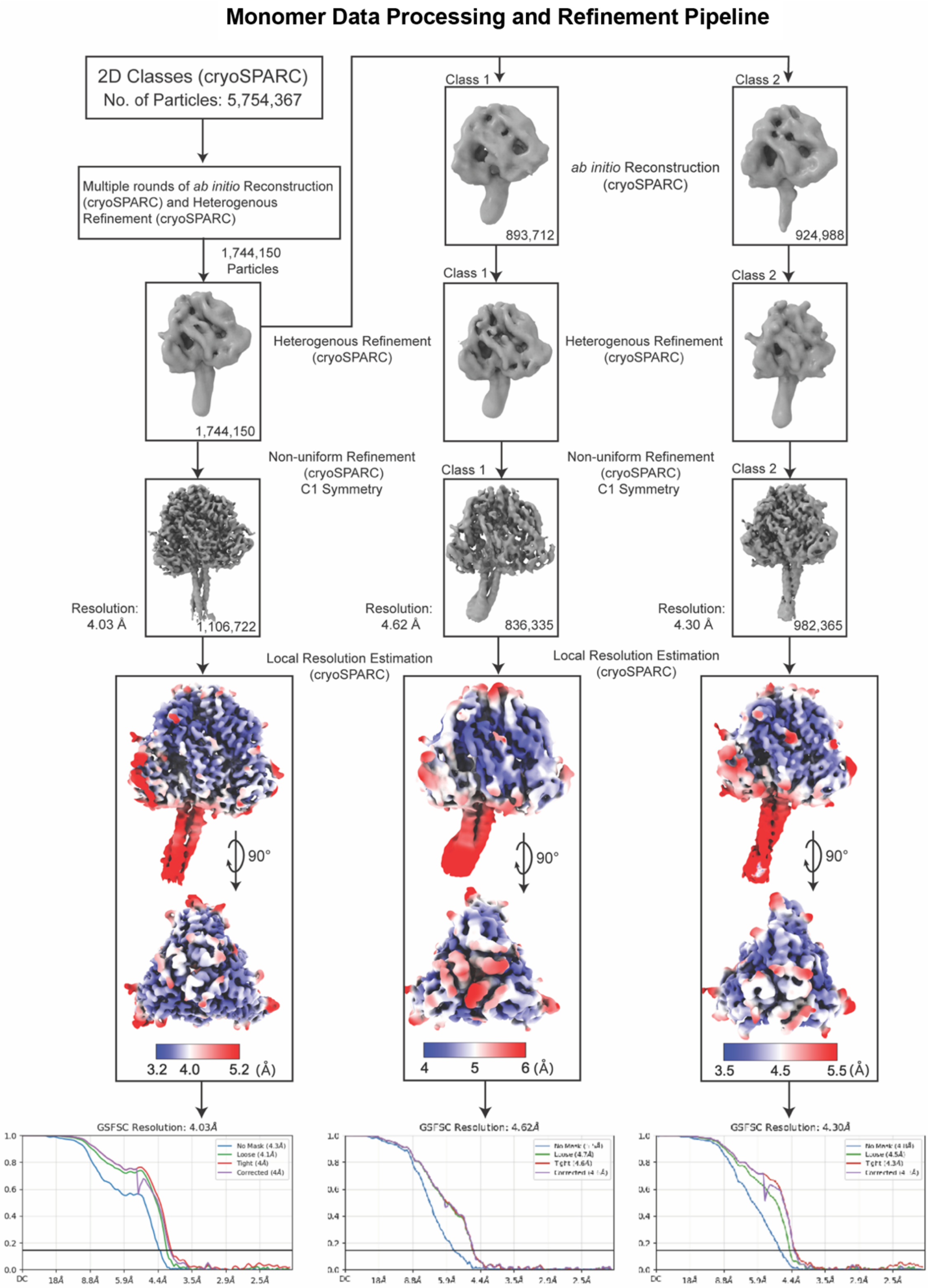

Supplemental Figure 11: Cryo-EM Data Processing and Refinement Outline of Monomer Pre-fusion AngV-F Protein. Representative 2D classes (Supplemental Figure 10) were selected for the *ab-intio* 3D reconstruction, with number of particles and software programs used in each step documented. Poor-quality particles were discarded after through heterogenous refinement and resulting volumes were processed using non-uniform refinement to obtain a high-resolution 3D reconstruction. Local resolutions were estimated and plotted on the side and top views of the AngV-F protein. Resolution values are shown as a blue to red spectrum for high-resolution regions (~3-4 Å) blue, and low-resolution regions (~5–6 Å) red. Structures were refined using C1 symmetry, with gold-standard Fourier shell correlation curves displayed at the bottom.

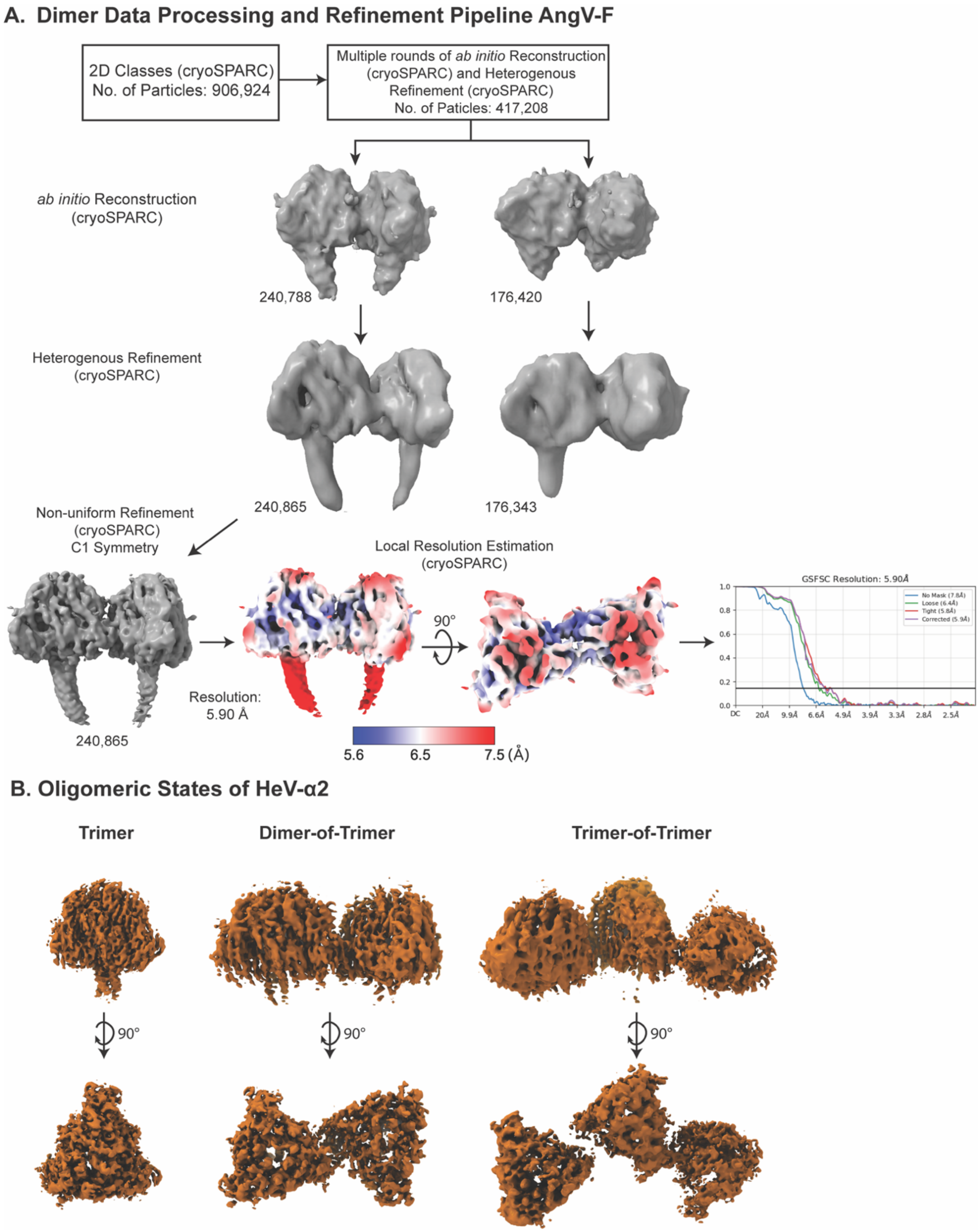

Supplemental Figure 12: Oligomeric States of AngV-F and HeV-α2-F. A. Cryo-EM Data Processing and Refinement Outline of Dimer pre-fusion AngV-F protein. Representative 2D classes (Supplemental Figure 10) were selected for the *ab-intio* 3D reconstruction, with number of particles and software programs used in each step documented. Poor-quality particles were discarded after heterogenous refinement and resulting volume was processed with non-uniform refinement to build a high-resolution 3D reconstruction. Local resolutions were estimated and plotted on the side and top views of the AngV-F Dimer. Resolution values are shown as a blue to red spectrum for high-resolution regions (~5-6 Å) blue, and low-resolution regions (~7.5 Å) red. Structures were refined using C1 symmetry, with gold-standard Fourier shell correlation curves displayed at the bottom. **B.** Cryo-EM HeV-α2-F ectodomain. The non-uniform refinement reconstruction (cryosparc) of trimer (left), dimer-of-trimers (middle) and trimer-of-trimers (right) are shown here.

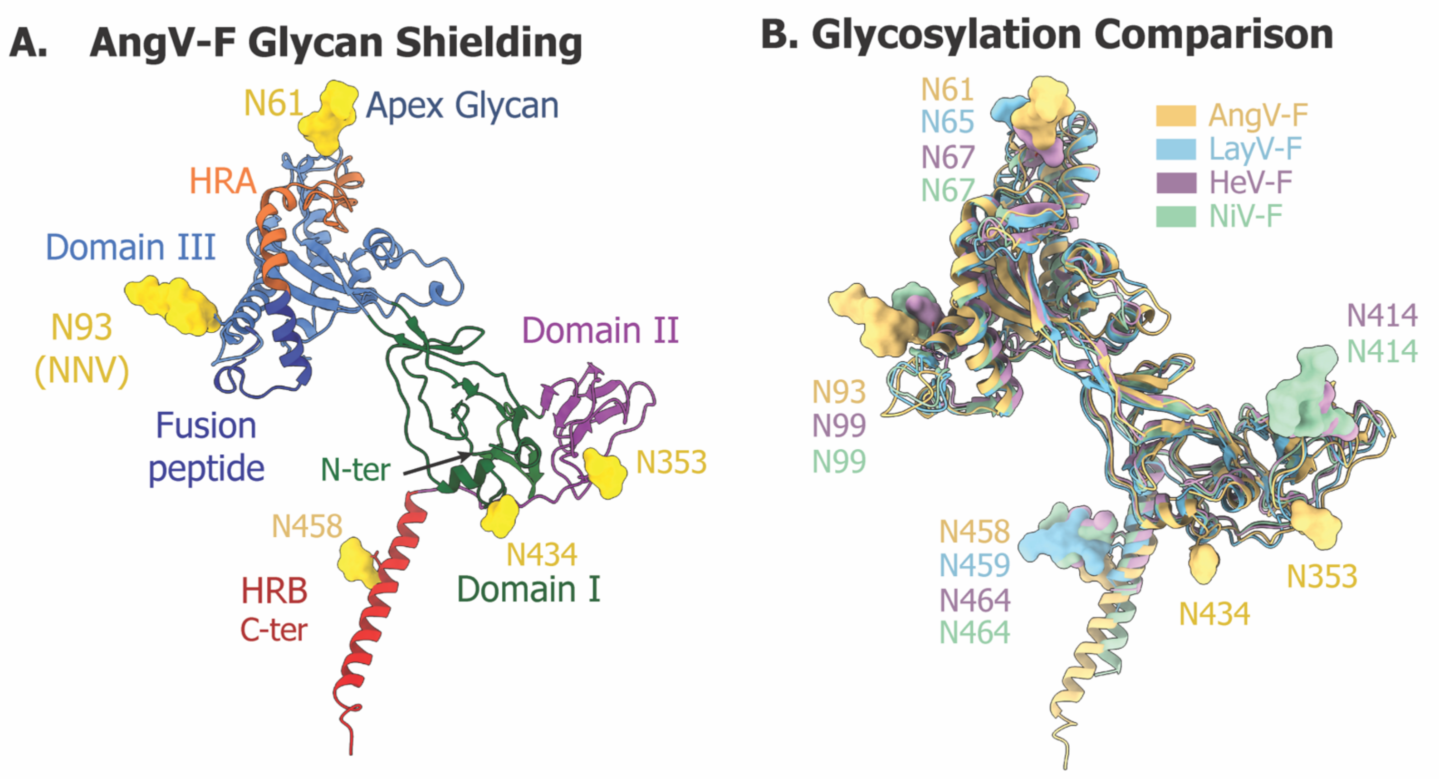

Supplemental Figure 13: AngV-F Glycan Distribution and Differences Compared to other HNV-F Proteins. **A.** Cartoon representation of an AngV-F protomer in the prefusion state, colored and annotated to highlight respective domains by functionality and glycans (yellow). **B.** Comparison of glycans among the F-proteins: AngV-F (yellow, from this study), LayV-F (blue, PDB: 8FMX), HeV-F (violet, PDB: 7KI6), and NiV-F (green, PDB: 5EVM).

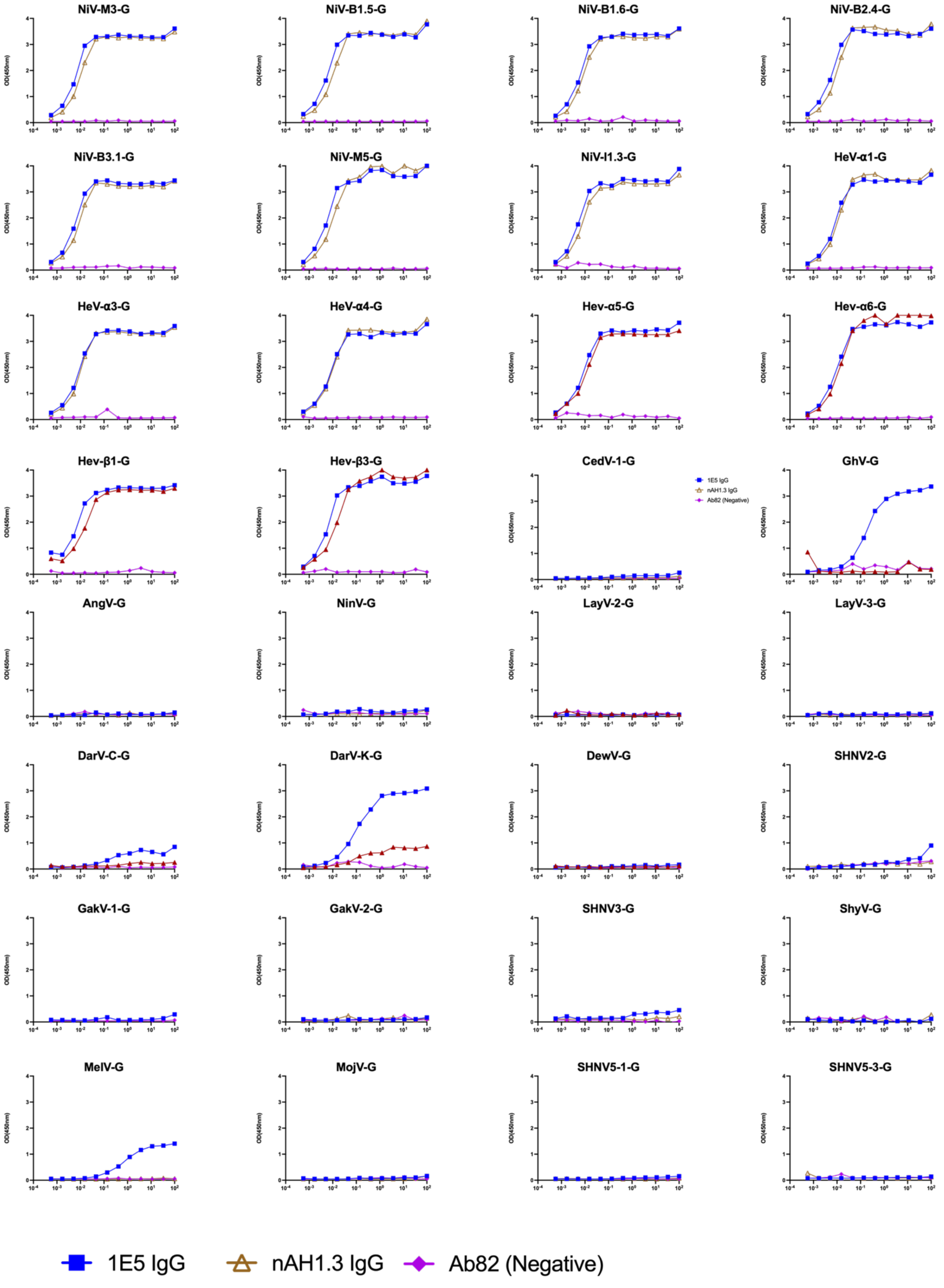

Supplemental Figure 14: Measurement of Antibody Binding to HNV-G Dead Domains using ELISA.

Supplemental Table 2. Cryo-Em Data Collection and Refinement Statistics.

	**AngV-F Trimer**	**AngV-F Dimer-of-Trimer**	**LayV-F with 22F5**
**PDB ID**	9MNH	9MQN	N/A
**EMDB ID**	EMD-48423	EMD-48535	EMD-48715
**Data collection and processing**			
Microscope	FEI Titan Krios	FEI Titan Krios	FEI Titan Krios
Detector	Gatan K3	Gatan K3	Gatan K3
Magnification	81000	81000	81000
Voltage (kv)	300	300	300
Electron exposure (e-/å^2)	65	65	59.6
DEFOCUS RANGE (μm)	-2.8 to -1.5	-2.8 to -1.5	-2.8 to -1.2
Pixel size (å)	1.1	1.1	1.1
Reconstruction software	cryoSPARC	cryoSPARC	cryoSPARC
Symmetry imposed	C1	C1	C1
Initial particle images (no.)	5,754,367	906,924	3,421,147
Final particle images (no.)	1,106,722	240,865	895,868
Map resolution (å)	4.03	5.9	3.49
Fsc threshold	0.143	0.143	0.143
**Model composition**			
Nonhydrogen atoms	10875	21278	
Protein residues	491	916	
Carbohydrates	30	48	
**Validation**			
Molprobity score	1.63	1.78	
Clashscore	3.73	6.05	
Poor rotamers (%)	0	0	
**Ramachandran plot**			
Favored regions (%)	92.45	93.09	
Allowed (%)	7.41	6.65	
Disallowed regions (%)	0.15	0.26	

Supplemental Table 3. Data Collection and Refinement Statistics (Molecular Replacement)

	**GakV-2-F Head Domain**
**PDB ID**	9EHU
**Data collection**	
Space group	P 31 2 1
Cell dimensions	
A, *b*, *c* (Å)	85.48, 85.48, 162.62
α, β, γ (°)	90, 90, 120
Resolution (Å)	67.38 – 1.42
R_{merge}	0.167 (7.383)
I / σI	10.4 (0.5)
Completeness (%)	97.5 (91.6)
Redundancy	16.8 (18.7)
**Refinement**	
Resolution (Å)	67.38 – 1.42
No. Unique reflections	128347 (3998)
R_{work} / r_{free}	0.2102/0.2271
No. Atoms	3756
Protein	3462
Water	224
Ligand	70
B-factors	
Protein	27.36
Water	30.74
Ligand	36.31
R.M.S. Deviations	
Bond lengths (Å)	0.0006
Bond angles (°)	0.86
Ramachandran	
Favored (%)	97.59
Allowed (%)	2.19
Outlier (%)	0.22

Supplemental Data 1: Sequence Alignment of HNV-F Proteins

Reference sequence (1): NiV-M1
Identities normalised by aligned length.
Colored by: identity

```
               cov    pid   1 [       .         .         .         .         :         .         .        . 80
 1 NiV-M1    100.0% 100.0%     ------------MVVI-----LDKRCYCNL-LILILMISECSVGILHYEKLSKIGLVKGVTRKYKIKSNPLTKDIVIKMI
 2 NiV-M2    100.0%  99.8%     ------------MVVI-----LDKRCYCNL-LILILMISECSVGILHYEKLSKIGLVKGVTRKYKIKSNPLTKDIVIKMI
 3 NiV-M3    100.0%  99.8%     ------------MVVI-----LDKRCYCNL-LILILMISECSVGILHYEKLSKIGLVKGVTRKYKIKSNPLTKDIVIKMI
 4 NiV-M4    100.0%  99.5%     ------------MVVI-----LDKRCYSNL-LILILMISECSVGILHYEKLSKIGLVKGVTRKYKIKSNPLTKDIVIKMI
 5 NiV-C     100.0%  98.9%     ------------MVVV-----LDKRCYSNL-LMLILMISECSVGILHYEKLSKIGLVKGITRKYKIKSNPLTKDIVIKMI
 6 NiV-B1.1  100.0%  98.5%     ------------MAVI-----LNKRYYSNL-LILILMISECSVGILHYEKLSKIGLVKGITRKYKIKSNPLTKDIVIKMI
 7 NiV-B1.2  100.0%  99.1%     ------------MAVI-----LNKRYYSNL-LILILMISECSVGILHYEKLSKIGLVKGITRKYKIKSNPLTKDIVIKMI
 8 NiV-B1.3  100.0%  98.9%     ------------MAVI-----LNKRYYSNL-LILILMISECSVGILHYEKLSKIGLVKGITRKYKIKSNPLTKDIVIKMI
 9 NiV-B1.4  100.0%  98.7%     ------------MAVI-----LNKRYYSNL-LILILMISECSVGILHYEKLSKIGLVKGITRKYKIKSNPLTKDIVIKMI
10 NiV-B2.3  100.0%  98.5%     ------------MAVI-----LNKRYYSNL-LILILMISECSVGILHYEKLSKVGLVKGITRKYKIKSNPLTKDIVIKMI
11 NiV-B3.1  100.0%  98.2%     ------------MAVI-----LNMRYYSNL-LILILMISECSVGILHYEKLSKIGLVKGITRKYKIKSNPLTKDIVIKMI
12 HeV-α1.1   99.8%  88.1%    ------------MAT------QEVRLKCLLCGIIVLVLSLEGLGILHYEKLSKIGLVKGITRKYKIKSNPLTKDIVIKMI
13 HeV-α2     99.8%  88.3%    ------------MAT------QEVRLKCLLCGIIVLVLSLEGLGILHYEKLSKIGLVKGITRKYKIKSNPLTKDIVIKMI
14 HeV-β1    100.0%  87.9%    ------------MATQ-----RVKVSYLIC-GIVISALSLEGLGILHYEKLSKIGLVKGVTRKYKIKSNPLTKDIVIKMI
15 HeV-β2    100.0%  87.7%    ------------MATQ-----GVKVSYLIC-GIVISALSLEGLGILHYEKLSKIGLVKGVTRKYKIKSNPLTKDIVIKMI
16 CedV-1     99.6%  42.9%     ------------MS-------NKRTTVLIIISYTLFYLNNAAIVGFDFDKLNKIGVVQGRVLNYKIKGDPMTKDLVLKFI
17 GhV(+A)    99.6%  51.5%     ------------MV-------INGNIITIILLLILILKTQMSEGAIHYETLSKIGLIKGITREYKVKGTPSSKDIVIKLI
18 AngV       98.0%  39.3%     ------------MGFR-----KNLRLLGIF-WLIILS------DTVDFERLASIGGIKGHSKLYKIKGHPTTKDIVIKLV
19 SHNV1      98.4%  37.7%     ---------------------MNYTIVGIM-IITFVHESQC----INYEQLASIGVIKGHTYNYKIRGPPNTKLMVVKLI
20 NinV       99.8%  38.0%     --MSENSHWYNIIYII-----MKSRCSRLIGLIIVLTALMSAVVGIDFEGLSSIGVVKGRTLNYKIRGNSAYKILVIKLT
21 SHNV2      99.1%  40.6%     ------------MSLKFRQSTNNRTNIRLILIIVLIKHMLC----IHYENLSSVGIIKGNTYNYKIKGDPSTKMLVVKLI
22 GakV-1     99.6%  39.9%     -------------MEL-----IKLFNILYL-VGYSGLFFTDVNAGLDYEGLSSIGVIKGPSYNYKIRGTPSTKLLVIKLI
23 GakV-2     99.6%  39.9%     -------------MEL-----IKLFYILYL-VGYSGLFFTDVNAGLDYEGLSSIGVIKGPSYNYKIRGTPSTKLLVIKLI
24 SHNV3      99.6%  39.6%     -------------MEL-----IKNYCLIYL-IFYSSTLIRPLQAGLDYEALASIGVIKGPSYNYKIRGTPSTKLLVIKLI
25 ShyV       99.8%  38.7%     MKLNNTRRDNKINKQHTTMACIKNCCLVYL-ILYSSTLIIPLEAGLDYESLASIGVIKGPSYNYKIRGTPSTKLLVIKLI
26 SHNV4      99.6%  40.6%     -------------MGL-----TKSTILVYL-LVYYHAHIIAINAGLDYEGLASIGVVKGPSYNYKIRGTPTTKLLVIKLI
27 MelV       99.1%  39.7%     ----------MAKFVF-----LKTMCIGLL-----IIISDRVDSSMDFHSLSKIGIIKGKTYNYKIRGEPNTKLMVIKLI
28 DewV       99.1%  40.1%     ---------------------MARLQVVIL-YLYLLVASDVVKASLDFDNMSKIGIIKGNTYNYKIRGEPTTKLMVVKLI
29 DarV-K     99.6%  40.6%     ------------MSS------SNKIRITVI-IINILISNYLIHCSMDFVELSRVGIIKGSTYNYKIRGEPSTKLMVVKLI
30 DarV-C     99.6%  40.4%     ------------MSS------SNKIRITVI-IINILISNYLINCSMDFVELSRVGIIKGSTYNYKIRGEPSTKLMVVKLV
31 LayV-1     98.9%  40.8%     -------------MAF-----LKSAIICYL-LFYPHI----VKSSLHYDSLSKVGIIKGLTYNYKIKGSPSTKLMVVKLI
32 LayV-2     98.9%  40.8%     -------------MAF-----LKSAIICYL-LFYPHI----VKSSLHYDSLSKVGIIKGLTYNYKIKGSPSTKLMVVKLI
33 MojV       99.5%  41.2%     ------------MA-------LNKNMFSSLFLGYLLVYATTVQSSIHYDSLSKVGVIKGLTYNYKIKGSPSTKLMVVKLI
34 SHNV5-1    99.8%  40.8%     ------------MINMD----SKRLKVKVVFPIYLIIYIQMSRASLDYDQLSKIGIVKGPSYNYKIKGSPSTKLMVVKLV
35 SHNV5-2    99.8%  40.8%     ------------MINME----SKRLKVKVVFSIYFIIYIQMSRASLDYDQLSKIGIVKGPSYNYKIKGSPSTKLMVVKLV

               cov    pid  81         .         1         .         .         .         :        . 160
 1 NiV-M1    100.0% 100.0%     PNV------SNMSQCTGSVMENYKTRLNGILTPIKGALEIYKNNTHDLVGDVRLAGVIMAGVAIGIATAAQITAGVALYE
 2 NiV-M2    100.0%  99.8%     PNV------SNMSQCTGSVMENYKTRLNGILTPIKGALEIYKNNTHDLVGDVRLAGVIMAGVAIGIATAAQITAGVALYE
 3 NiV-M3    100.0%  99.8%     PNV------SNMSQCTGSVMENYKTRLNGILTPIKGALEIYKNNTHDLVGDVRLAGVIMAGVAIGIATAAQITAGVALYE
 4 NiV-M4    100.0%  99.5%     ANV------SNMSQCTGSVMENYKTRLNGILTPIKGALEIYKNNTHDLVGDVRLAGVIMAGVAIGIATAAQITAGVALYE
 5 NiV-C     100.0%  98.9%     PNV------SNMSQCTGSVMENYKTRLNGILTPIKGALEIYKNNTHDLVGDVRLAGVIMAGVAIGIATAAQITAGVALYE
 6 NiV-B1.1  100.0%  98.5%     PNV------SNMSQCTGSVMENYKTRLNGILTPIKGALEIYKNNTHDLVGDVRLAGVIMAGVAIGIATAAQITAGVALYE
 7 NiV-B1.2  100.0%  99.1%     PNV------SNMSQCTGSVMENYKTRLNGILTPIKGALEIYKNNTHDLVGDVRLAGVIMAGVAIGIATAAQITAGVALYE
 8 NiV-B1.3  100.0%  98.9%     PNV------SNMSQCTGSVMENYKTRLNGILTPIKGALEIYKNNTHDLVGDVRLAGVIMAGVAIGIATAAQITAGVALYE
 9 NiV-B1.4  100.0%  98.7%     PNV------SNMSQCTGSVMENYKTRLNGILTPIKGALEIYKNNTHDLVGDVRLAGVIMAGVAIGIATAAQITAGVALYE
10 NiV-B2.3  100.0%  98.5%     PNV------SNMSQCTGSVMENYKTRLNGILTPIKGALEIYKNNTHDLVGDVRLAGVIMAGVAIGIATAAQITAGVALYE
11 NiV-B3.1  100.0%  98.2%     PNV------SNMSQCTGSVMENYKTRLNGILMPIKGALEIYKNNTHDLVGDVRLAGVIMAGVAIGIATAAQITAGVALYE
12 HeV-α1.1   99.8%  88.1%    PNV------SNVSKCTGTVMENYKSRLTGILSPIKGAIELYNNNTHDLVGDVKLAGVVMAGIAIGIATAAQITAGVALYE
13 HeV-α2     99.8%  88.3%    PNV------SNVSKCTGTVMENYKSRLTGILSPIKGAIELYNNNTHDLVGDVKLAGVVMAGIAIGIATAAQITAGVALYE
14 HeV-β1    100.0%  87.9%    PNV------SNVSKCTGTVMENYKNRLTGILSPIKGAIELYNNNTHDLIGDVKLAGVVMAGIAIGIATAAQITAGVALYE
15 HeV-β2    100.0%  87.7%    PNV------SNVSKCTGTVMENYKNRLTGILSPIRGAIELYNNNTHDLIGDVKLAGVVMAGIAIGIATAAQITAGVALYE
16 CedV-1     99.6%  42.9%     PNI------VNITECVREPLSRYNETVRRLLLPIHNMLGLYLNNTNAKMTGLMIAGVIMGGIAIGIATAAQITAGFALYE
17 GhV(+A)    99.6%  51.5%     PNV------TGLNKCTNISMENYKEQLDKILIPINNIIELYANSTKSAPGNARFAGVIIAGVALGVAAAAQITAGIALHE
18 AngV       98.0%  39.3%     PNL------NNLTVCSEMSIDGYLNLINAVIIPISQSLELMRNNVKDGTPNENIFGAIVAGAALGIATAAQVTSVVALHK
19 SHNV1      98.4%  37.7%     PNINIDNLGGGLSNCSSKQMESHKELVEKVLSPVAQALETMRNRVTDYSGNYRFVGAVMAGAALGVATAATVTAGIALHQ
20 NinV       99.8%  38.0%     PTIRSTSGAVNFENCTKDQISAHKVLVRNVLTPVKDALTSMKSKVTDYTPNSRLFGAIVAGAALVTATAATITAGVALHQ
21 SHNV2      99.1%  40.6%     PNI------DGLGNCTDKQMKDYKTLVKSTLQPVKDSLAQMLNNVETYNGYVKFFGAVMAGAALGVATAATVTAGIALHQ
22 GakV-1     99.6%  39.9%     PNV------ESIDNCTQKQMSDYRALVKNVLTPVSESLSTMLNYIEQQSNGVRLIGAVLAGAALGVATGAAITAGIALHK
23 GakV-2     99.6%  39.9%     PNV------ESIDNCTQKQMSDYRALVKNVLTPVSESLSTMLNYIEQQSNGVRLIGAVLAGAALGVATGAAITAGIALHK
24 SHNV3      99.6%  39.6%     PNV------GSLDNCTQKQMSDYRALVKNVLTPVSESLKTMLNYIEQQSNGVRLIGAVLAGAALGVATGAAITAGIALHK
25 ShyV       99.8%  38.7%     PNV------GSLDNCTQKQMADYKALVKNVLTPVSESLKTMLNYIEQQSNGVRLIGAVLAGAALGVATGAAITAGIALHK
26 SHNV4      99.6%  40.6%     PNV------GSLDNCTQKQMADYKSLVKNVLTPVSDALSTMLNYIEQQSNGVRLIGAVLAGAALGVATGAAITAGIALHK
27 MelV       99.1%  39.7%     PNI------DVVENCSTTQVNNYKKLVRNVLTPVRIISRYYVKNVIEQNNRVRLFGAIMAGAALGVATAATVTAGIALHR
28 DewV       99.1%  40.1%     PNI------DVVENCSATQLANYKKLVTNVLTPVKLSLDNMLKNVQDQNNRVRLFGAIMAGAALGVATAATVTAGIALHR
29 DarV-K     99.6%  40.6%     PNV------GDVANCSATQVSNYKKLVKNVLSPVSNALNTMLENVQTENNRYRLFGAIMAGAALGVATAATVTAGIALHR
30 DarV-C     99.6%  40.4%     PNV------GDVANCSTTQVSNYKKLVKNVLSPVSNALNTMLENVQTENNRYRLFGAIMAGAALGVATAATVTAGIALHR
31 LayV-1     98.9%  40.8%     PNI------DGVRNCTQKQFDEYKNLVKNVLEPVKIALNAMLDNVKSGNNKYRFAGAIMAGVALGVATAATVTAGIALHR
32 LayV-2     98.9%  40.8%     PNI------DRVRNCTQKQFDEYKNLVKNVLEPVKLALNAMLDNVKSGNNKYRFAGAIMAGVALGVATAATVTAGIALHR
33 MojV       99.5%  41.2%     PNI------DSVKNCTQKQYDEYKNLVRKALEPVKMAIDTMLNNVKSGNNKYRFAGAIMAGVALGVATAATVTAGIALHR
34 SHNV5-1    99.8%  40.8%     PNI------KEVANCTHKQVENYKTLVRNVLDPVKMSLQAMLEKVKSGNNKYRFAGAIMAGVALGVATAATVTAGIALHR
35 SHNV5-2    99.8%  40.8%     PNI------KEVANCTHKQIENYKTLVRNVLDPVKMSLQAMLEKVKSGNNKYRFAGAIMAGVALGVATAATVTAGIALHR
```

		cov	pid	161 . . . 2 240
1	NiV-M1	100.0%	100.0%	AMKNADNINKLKSSIESTNEAVVKLQETAEKTVYVLTALQDYINTNLVPTIDKISCKQTELSLDLALSKYLSDLLFVFGP
2	NiV-M2	100.0%	99.8%	AMKNADNINKLKSSIESTNEAVVKLQETAEKTVYVLTALQDYINTNLVPTIDKISCKQTELSLDLALSKYLSDLLFVFGP
3	NiV-M3	100.0%	99.8%	AMKNADNINKLKSSIESTNEAVVKLQETAEKTVYVLTALQDYINTNLVPTIDKISCKQTELSLDLALSKYLSDLLFVFGP
4	NiV-M4	100.0%	99.5%	AMKNADNINKLKSSIESTNEAVVKLQETAEKTVYVLTALQDYINTNLVPTIDKISCKQTELSLDLALSKYLSDLLFVFGP
5	NiV-C	100.0%	98.9%	AMKNADNINKLKSSIESTNEAVIKLQETAEKTVYVLTALQDYINTNLVPTIDKISCKQTELSLDLALSKYLSDLLFVFGP
6	NiV-B1.1	100.0%	98.5%	AMKNADNINKLKSSIESTNEAVVKLQETAEKTVYVLTALQDYINTNLVPTIDKISCKQTELSLDLALSKYSSDLLFVFGP
7	NiV-B1.2	100.0%	99.1%	AMKNADNINKLKSSIESTNEAVVKLQETAEKTVYVLTALQDYINTNLVPTIDKISCKQTELSLDLALSKYLSDLLFVFGP
8	NiV-B1.3	100.0%	98.9%	AMKNADNINKLKSSIESTNEAVVKLQETAEKTVYVLTALQDYINTNLVPTIDKISCKQTELSLDLALSKYLSDLLFVFGP
9	NiV-B1.4	100.0%	98.7%	AMKNADNINKLKSSIESTNEAVVKLQETAEKTVYVLTALQDYINTNLVPTIDKISCKQTELSLDLALSKYLSDLLFVFGP
10	NiV-B2.3	100.0%	98.5%	AMKNADNINKLKSSIESTNEAVVKLQETAEKTVYVLTALQDYINTNLVPTIDKISCKQTELSLDLALSKYLSDLLFVFGP
11	NiV-B3.1	100.0%	98.2%	AMKNADNINKLKSSIESTNEAVVKLQETAEKTVYVLTALQDYINTNLVPTIDKISCKQMELSLDLALSKYLSDLLFVFGP
12	HeV-α1.1	99.8%	88.1%	AMKNADNINKLKSSIESTNEAVVKLQETAEKTVYVLTALQDYINTNLVPTIDQISCKQTELALDLALSKYLSDLLFVFGP
13	HeV-α2	99.8%	88.3%	AMKNADNINKLKSSIESTNEAVVKLQETAEKTVYVLTALQDYINTNLVPTIDQISCKQTELALDLALSKYLSDLLFVFGP
14	HeV-β1	100.0%	87.9%	AMKNADNINKLKSSIESTNEAVVKLQETAEKTVYVLTALQDYINTNLVPTIDQISCKQTELALDLALSKYLSDLLFVFGP
15	HeV-β2	100.0%	87.7%	AMKNADNINKLKSSIESTNEAVVKLQETAEKTVYVLTALQDYINTNLVPTIDQISCKQTELALDLALSKYLSDLLFVFGP
16	CedV-1	99.6%	42.9%	AKKNTENIQKLTDSIMKTQDSIDKLTDSVGTSILILNKLQTYINNQLVPNLELLSCRQNKIEFDLMLTKYLVDLMTVIGP
17	GhV(+A)	99.6%	51.5%	ARQNAERINLLKDSISATNNAVAELQEATGGIVNVITGMQDYINTNLVPQIDKLQCSQIKTALDISLSQYYSEILTVFGP
18	AngV	98.0%	39.3%	SNQNAAKINSLRDSITKTNQAVEQLSLGVQETVSVLMGLQDQINTNLVPKINILSCKQLSNTLNIMLLQYYSQILTVFGP
19	SHNV1	98.4%	37.7%	SNQNAKAIDQMKEAIRTTNKAVQELTLSTRQTLLVIDSLQNQINTQIVPAMNRLSCEVLGLTVGIQLTQYYSEILTYFGP
20	NinV	99.8%	38.0%	SNQNAQAIANMKDAISKTNQAVSELKQGTQKLATVVDSLQNQINSQIIPVLNQLGCQTTGLTIGLYLTRYYSEILTVFGP
21	SHNV2	99.1%	40.6%	SNQNAKDIANMKDAIMKTNQAITTLSSASQKMLTVIDSIRGEINQQIIPVLNQLSCETVGLNLGIKLTQYYSQISTFFGP
22	GakV-1	99.6%	39.9%	SNQNAQAIAQLKDAIKNTNQAVQTLKMANQELLGVVDSLRGQINTQIIPVLNKLSCDTVGLTLGIKLTQYYSEILTAFGP
23	GakV-2	99.6%	39.9%	SNQNAQAIAQLKDAIKNTNQAVQTLKMANQELLGVVDSLRGQINTQIIPVLNKLSCDTVGLTLGIKLTQYYSEILTAFGP
24	SHNV3	99.6%	39.6%	SNQNAQAIAQLKDSIKNTNMAVQTLKLANQEILGVVDSLRGQINTQIIPVLNQLSCDTVGLTLGIKLTQYYSEILTAFGP
25	ShyV	99.8%	38.7%	SNQNAQAIAQLKDSIKNTNMAVQTLKLANQEILGVVDSLRGQINTQIIPVLNQLSCDTVGLTLGIKLTQYYSEILTAFGP
26	SHNV4	99.6%	40.6%	SNQNAQAIAQLKDAIKNTNQAVQTLKLANQEILGVVDSLRGQINTQIIPVINQLSCDTIGLTLGIKLTQYYSEILTAFGP
27	MelV	99.1%	39.7%	SNENARNIALMKEAIKNTNQAVTKLQLAGQQTLAVIDNIRGEINNQIIPVINKLTCENIGLNVGIKLTQYYSEVLTAFGP
28	DewV	99.1%	40.1%	SNENAKNIAKLKNAIQNTNLAITKLQMAGQQTLAVIDNIRGEINNQIIPVLNQLSCETVGLNVGIKLTQYYSEVLTAFGP
29	DarV-K	99.6%	40.6%	SNENARNIAAMKNAIQNTNQAITKLQMAGQQTLAVIDNIRGEINNQIIPLLNKLSCETVGLNVGIKLTQYYSEILTVFGP
30	DarV-C	99.6%	40.4%	SNENARNIAAMKNAIQNTNQAITKLQMAGQQTLAVIDNIRGEINNQIIPLLNKLSCETVGLNVGIKLTQYYSEILTVFGP
31	LayV-1	98.9%	40.8%	SNENAQAIANMKNAIQNTNEAVKQLQLANKQTLAVIDTIRGEINNNIIPVINQLSCDTIGLSVGIKLTQYYSEILTAFGP
32	LayV-2	98.9%	40.8%	SNENAQAIANMKNAIQNTNEAVKQLQLANKQTLAVIDTIRGEINNNIIPVINQLSCDTIGLSVGIKLTQYYSEILTAFGP
33	MojV	99.5%	41.2%	SNENAQAIANMKSAIQNTNEAVKQLQLANKQTLAVIDTIRGEINNNIIPVINQLSCDTIGLSVGIRLTQYYSEIITAFGP
34	SHNV5-1	99.8%	40.8%	SSENAQAIARIKGAIQNTNEAVKQLQLANKQMLAVIDTIRGEINENIIPIMNELSCETIGLNVGIKLTQYYSEIITAFGP
35	SHNV5-2	99.8%	40.8%	SSENAQAIARIKGAIQNTNEAVKQLQLGNKQMLAVIDTIRGEINENIIPIMNELSCETIGLNVGIKLTQYYSEIITAFGP

		cov	pid	241 : . . . . 3 . . 320
1	NiV-M1	100.0%	100.0%	NLQDPVSNSMTIQAISQAFGGNYETLLRTLGYATEDFDDLLESDSITGQIIYVDLSSYYIIVRVYFPILTEIQQAYIQEL
2	NiV-M2	100.0%	99.8%	NLQDPVSNSMTIQAISQAFGGNYETLLRTLGYATEDFDDLLESDSITGQIIYVDLSSYYIIVRVYFPILTEIQQAYIQEL
3	NiV-M3	100.0%	99.8%	NLQDPVSNSMTIQAISQAFGGNYETLLRTLGYAIEDFDDLLESDSITGQIIYVDLSSYYIIVRVYFPILTEIQQAYIQEL
4	NiV-M4	100.0%	99.5%	NLQDPVSNSMTIQAISQAFGGNYETLLRTLGYATEDFDDLLESDSITGQIIYVDLSSYYIIVRVYFPILTEIQQAYIQEL
5	NiV-C	100.0%	98.9%	NLQDPVSNSMTIQAISQAFGGNYETLLRTLGYATEDFDDLLESDSITGQIIYVDLSSYYIIVRVYFPILTEIQQAYIQEL
6	NiV-B1.1	100.0%	98.5%	NLQDPVSNSMTIQAISQAFGGNYETLLRTLGYATEGFDDLLESDSITGQIIYVDLSGYYIIVRVYFPILTEIQQAYIQEL
7	NiV-B1.2	100.0%	99.1%	NLQDPVSNSMTIQAISQAFGGNYETLLRTLGYATEDFDDLLESDSITGQIIYVDLSSYYIIVRVYFPILTEIQQAYIQEL
8	NiV-B1.3	100.0%	98.9%	NLQDPVSNSMTIQAISQAFGGNYETLLRTLGYATEDFDDLLESDSITGQIIYVDLSGYYIIVRVYFPILTEIQQAYIQEL
9	NiV-B1.4	100.0%	98.7%	NLQDPVSNSMTIQAISQAFGGNYETLLRTLGYATEDFDDLLESDSITGQIIYVDLSGYYIIVRVYFPILTEIQQAYIQEL
10	NiV-B2.3	100.0%	98.5%	NLQDPVSNSMTIQAISQAFGGNYETLLRTLGYTTEDFDDLLESDSITGQIIYVDLSGYYIIVRVYFPILTEIQQAYIQEL
11	NiV-B3.1	100.0%	98.2%	NLQDPVSNSMTIQAISQAFGGNYETLLRTLGYATEDFDDLLESDSITGQIIYVDLSGYYIIVRVYFPILTEIQQAYIQEL
12	HeV-α1.1	99.8%	88.1%	NLQDPVSNSMTIQAISQAFGGNYETLLRTLGYATEDFDDLLESDSIAGQIVYVDLSSYYIIVRVYFPILTEIQQAYVQEL
13	HeV-α2	99.8%	88.3%	NLQDPVSNSMTIQAISQAFGGNYETLLRTLGYATEDFDDLLESDSITGQIVYVDLSSYYIIVRVYFPILTEIQQAYVQEL
14	HeV-β1	100.0%	87.9%	NLQDPVSNSMTIQAISQAFGGNYETLLRTLGYATEDFDDLLESDSITGQIVYVDLSSYYIIVRVYFPILTEIQQAYVQEL
15	HeV-β2	100.0%	87.7%	NLQDPVSNSMTIQAISQAFGGNYETLLRTLGYATEDFDDLLESDSITGQIVYVDLSSYYIIVRVYFPILTEIQQAYVQEL
16	CedV-1	99.6%	42.9%	NINNPVNKDMTIQSLSLLFDGNYDIMMSELGYTPQDFLDLIESKSITGQIIYVDMENLYVVIRTYLPTLIEVPDAQIYEF
17	GhV(+A)	99.6%	51.5%	NLQNPVTTSMSIQAISQSFGGNIDLLLNLLGYTANDLLDLLESKSITGQITYINLEHYFMVIRVYYPIMTTISNAYVQEL
18	AngV	98.0%	39.3%	NLRDPATVPVSIQALSQLFEGNLELLTSSLGISSTDFNDLLTGKLITGSIIWADTQGHYLILRVNIPDLIEVPGAVIQEF
19	SHNV1	98.4%	37.7%	ALQDPIDSTLTIQAISHAFGGNFDILMKTMGYTVGDLYDVLKGDLITGKIISVNPKEGFIALEVRFPTLTQVNNAIVQEL
20	NinV	99.8%	38.0%	AIQDPVNSALTIQAISKAFEGNFDKLMSAMGYSVSDLKDVLESDLVRGKIIDVDPDVGYMALQIEFPQITPVTGAHIQEL
21	SHNV2	99.1%	40.6%	ALQNPVQSILTIQAISHAFGGNFNELMTVMGYTGSDFQDIIQGNLITGSVIGVDPEVGYIALEIRIPSLTVVPNAYVQEL
22	GakV-1	99.6%	39.9%	AIQDPVNSKLTIQAISGAFSGNFDEMMKVMGYSGSDLHDILQGDLITGNIIGVDPDVGYIALEINFPTLTEIPNAVIQEL
23	GakV-2	99.6%	39.9%	AIQDPVNSKLTIQAISGAFSGNFDEMMKVMGYSGSDLHDILQGDLITGNIIGVDPDVGYIALEINFPTLTEIPNAVIQEL
24	SHNV3	99.6%	39.6%	AIQDPVNSKLTIQAISGAFSGNFDEMMKVMGYTGSDLHDILQGDLITGNIIGVDPDVGYIALEIHFPTLTEIPNAVIQEL
25	ShyV	99.8%	38.7%	AIQDPVNSKLTIQAISGAFSGNFDEMMKVMGYTGSDLHDILQGDLITGNIIGVDPDVGYIALEIHFPTLTEIPNAVIQEL
26	SHNV4	99.6%	40.6%	AIQDPVNSKLTIQAISGAFSGNFDEMMKVMGYTGSDLHDILQGDLITGNIIGVDPDIGYIALEIHFPTLTEIPNAVIQEL
27	MelV	99.1%	39.7%	AIQDPVNARITIQAISKVFNNNFDELLNVMGYSTQDLYEVLHGGLIRGNVISADPEVGYLALEIEFPNLSVVPNAYIQEI
28	DewV	99.1%	40.1%	AIQDPVNSKITIQAISKAFNNNFDELLKVMGYTSQDLYEILHGDLITGNVISADPEAGYIALEIEFPNLSTVPNAYVQEL
29	DarV-K	99.6%	40.6%	AIQDPINSKITIQAISKAFGGNFDELLKVMGYTSQDLYEILHGGLITGNIIGVDPDTGYIALEIEFPNLSIVQNAYIQEL
30	DarV-C	99.6%	40.4%	AIQDPINSKITIQAISKAFGGNFDELLNVMGYTSQDLYEILHGGLITGNIIGVDPDTGYIALEIEFPNLSIVQNAYIQEL
31	LayV-1	98.9%	40.8%	ALQNPVNTRITIQAISSVFNRNFDELLKIMGYTSGDLYEILHSGLIRGNIIDVDVEAGYIALEIEFPNLTLVPNAVVQEL
32	LayV-2	98.9%	40.8%	ALQNPVNTRITIQAISSVFNRNFDELLKIMGYTSGDLYEILHSGLIRGNIIDVDVEAGYIALEIEFPNLTLVPNAVVQEL
33	MojV	99.5%	41.2%	ALQNPVNTRITIQAISSVFNGNFDELLKIMGYTSGDLYEILHSELIRGNIIDVDVDAGYIALEIEFPNLTLVPNAVVQEL
34	SHNV5-1	99.8%	40.8%	ALQNPIGSKITIQAISSAFNGNFDELLKSMGYTSNDLYEVLQSGLIRGNIIDVDPEVGYIALEIEFPNLTLVPNAIIQEL
35	SHNV5-2	99.8%	40.8%	ALQNPIGSKITIQAISSAFNGNFDELLKSMGYTSNDLYEVLQSGLIRGNIIDVDPEAGYIALEIEFPNLTLVPNAIIQEL

		cov	pid	321 . . : 4 400
1	NiV-M1	100.0%	100.0%	LPVSFNNDNSEWISIVPNFILVRNTLISNIEIGFCLITKRSVICNQDYATPMTNNMRECLTGSTEKCPRELVVSSHVPRF
2	NiV-M2	100.0%	99.8%	LPVSFNNDNSEWISIVPNFILVRNTLISNIEIGFCLITKRSVICNQDYATPTTNNMRECLTGSTEKCPRELVVSSHVPRF
3	NiV-M3	100.0%	99.8%	LPVSFNNDNSEWISIVPNFILVRNTLISNIEIGFCLITKRSVICNQDYATPMTNNMRECLTGSTEKCPRELVVSSHVPRF
4	NiV-M4	100.0%	99.5%	LPVSFNNDNSEWISIVPNFILVRNTLISNIEIGFCLITKRSVICNQDYATPMTNNMRECLTGSTEKCPRELVVSSHVPRF
5	NiV-C	100.0%	98.9%	LPVSFNNDNSEWISIVPNFILVRNTLISNIEIGFCLITKRSVICNQDYATPMTNNMRECLTGSTEKCPRELVVSSHVPRF

```
 6 NiV-B1.1  100.0%  98.5%    LPVSFNNDNSEWISIVPNFILVRNTLISNIEIGFCLITKRSVICNQDYATPMTNNMRECLTGSTEKCPRELVVSSHVPRF
 7 NiV-B1.2  100.0%  99.1%    LPVSFNNDNSEWISIVPNFILVRNTLISNIEIGFCLITKRSVICNQDYATPMTNNMRECLTGSTEKCPRELVVSSHVPRF
 8 NiV-B1.3  100.0%  98.9%    LPVSFNNDNSEWISIVPNFILVRNTLISNIEIGFCLITKRSVICNQDYATPMTNNMRECLTGSTEKCPRELVVSSHVPRF
 9 NiV-B1.4  100.0%  98.7%    LPVSFNNDNSEWISIVPNFILVRNTLISNIEIGFCLITKRSVICNQDYATPMTNNMRECLTGSTEKCPRELVVSSHVPRF
10 NiV-B2.3  100.0%  98.5%    LPVSFNNDNSEWISIVPNFILVRNTLISNIEIGFCLITKRSVICNQDYATPMTNNMRECLTGSTEKCPRELVVSSHVPRF
11 NiV-B3.1  100.0%  98.2%    LPVSFNNDNSEWISIVPNFILVRNTLISNIEIGFCLITKRSVICNQDYATPMTNNMRECLTGSTEKCPRELVVSSHVPRF
12 HeV-α1.1   99.8%  88.1%    LPVSFNNDNSEWISIVPNFVLIRNTLISNIEVKYCLITKKSVICNQDYATPMTASVRECLTGSTDKCPRELVVSSHVPRF
13 HeV-α2     99.8%  88.3%    LPVSFNNDNSEWISIVPNFVLIRNTLISNIEVKYCLITKKSVICNQDYATPMTASVRECLTGSTDKCPRELVVSSHVPRF
14 HeV-β1    100.0%  87.9%    LPVSFNNDNSEWISIVPNFVLIRNTLISNIEVKYCLTTKKSVICNQDYATPMTSTVRECLTGSTDKCPRELVVSSHVPRF
15 HeV-β2    100.0%  87.7%    LPVSFNNDNSEWISIVPNFVLIRNTLISNIEVKYCLTTKKSVICNQDYATPMTSTVRECLTGSTDKCPRELVVSSHVPRF
16 CedV-1     99.6%  42.9%    NKITMSSNGGEYLSTIPNFILIRGNYMSNIDVATCYMTKASVICNQDYSLPMSQNLRSCYQGETEYCPVEAVIASHSPRF
17 GhV(+A)    99.6%  51.5%    IKISFNVDGSEWVSLVPSYILIRNSYLSNIDISECLITKNSVICRHDFAMPMSYTLKECLTGDTEKCPREAVVTSYVPRF
18 AngV       98.0%  39.3%    IKIGYNFGGSLWMPTIPNKIIIRGYHLSLIDSTNCIITDNSYVCDRDYSLPMNPILRECFEGNTSSCGREMVLNSYIPLY
19 SHNV1      98.4%  37.7%    MPISFNDKGDEWISTVPRYVLERVLYLSNIDISLCSVGETSVVCDNDYASPMSHQLRECLQTNTSYCPRERVLASYVPKF
20 NinV       99.8%  38.0%    LPISFNYKGDEWITILPSFVLNRMTYLSNIDIAHCLVTETSVICDNDLAMPMSTQMRDCLLGNTSKCSREQVITSYVPRF
21 SHNV2      99.1%  40.6%    LPVSFNIDGDEWMTIVPNYVLTRTTYLSNIDINRCLITDKSVICGNDYATPMSNQLINCLNGNTQHCAREAVVTSYVPKF
22 GakV-1     99.6%  39.9%    MPISFNDKGDEWMALVPRYVLLRTTYISNIDISKCLLTERSVICYNDYATPMSFDIIRCLTGNLTYCPREQIMASHVPKF
23 GakV-2     99.6%  39.9%    MPISFNDKGDEWMALVPRYVLLRTTYISNIDISKCLLTERSVICYNDYATPMSFDIIRCLTGNLTYCPREQIMASHVPKF
24 SHNV3      99.6%  39.6%    MPISFNDKGDEWMALVPRYVLLRTTYVSNIDISKCLLTERSVICYNDYATPMSFDVLRCLTGNLTFCPREQIIASHVPKF
25 ShyV       99.8%  38.7%    MPISFNDKGDEWMALVPRYVLLRTTYVSNIDISKCLLTERSVICYNDYATPMSFDVLRCLTGNLTFCPREQIIASHVPKF
26 SHNV4      99.6%  40.6%    MPISFNDKGDEWMTLVPRYVLLRTTYVSNIDISKCLITERSVICYNDYATPMSFDVIRCLTGNLTYCPREQIIASYVPRF
27 MelV       99.1%  39.7%    MPISFNVDGDEWVTVVPRHTLIRTTLLSNIDITTCSIIESSIICNNDYALPMSNELINCLQGSTDLCAREKVISNYVPKF
28 DewV       99.1%  40.1%    MPISFNVDGDEWVTLVPRYVLIRTTFLSNIDISLCSIMETSIVCNNDYALPMSSELINCLQGDTGVCAREKVISSYVPKF
29 DarV-K     99.6%  40.6%    MPISFNVDGDEWVTLAPRFVLIRTTLLSNIDTSMCTIIDSSVICNNDYALPMSTELINCLQGMTESCAREKVISSYVPKF
30 DarV-C     99.6%  40.4%    MPISFNVDGDEWVTLAPRFVLTRTTLLSNIDTSMCTIIDSSVICNNDYALPMSTELINCLQGMTESCAREKVISSYVPKF
31 LayV-1     98.9%  40.8%    MPISYNVDGDEWVTLVPRFVLTRTTLLSNIDTSRCTVTESSVICDNDYALPMSYELIGCLQGDTSKCAREKVVSSYVPRF
32 LayV-2     98.9%  40.8%    MPISYNVDGDEWVTLVPRFVLTRTTLLSNIDTSRCTVTESSVICDNDYALPMSYELIGCLQGDTSKCAREKVVSSYVPRF
33 MojV       99.5%  41.2%    MPISYNIDGDEWVTLVPRFVLTRTTLLSNIDTSRCTITDSSVICDNDYALPMSHELIGCLQGDTSKCAREKVVSSYVPKF
34 SHNV5-1    99.8%  40.8%    MPISFNSEGDEWVTLMPRFILTRTTLLSNIDITKCTVTDRSVICDNDYALPMSNQLIECLRGDTMKCTREKVMSSYIPKF
35 SHNV5-2    99.8%  40.8%    MPISFNSEGDEWVTLMPRFILTRTTLLSNIDITKCTVTDRSVICDNDYALPMSNQLIECLRGDTMKCTREKVMSSYIPKF

                cov    pid 401         .         .         .         .         :         .         .         . 480
 1 NiV-M1    100.0% 100.0%    ALSNGVLFANCISVTCQCQTTGRAISQSGEQTLLMIDNTTCPTAVLGNVIISLGKYLGSVNYNSEGIAIGPPVFTDKVDI
 2 NiV-M2    100.0%  99.8%    ALSNGVLFANCISVTCQCQTTGRAISQSGEQTLLMIDNTTCPTAVLGNVIISLGKYLGSVNYNSEGIAIGPPVFTDKVDI
 3 NiV-M3    100.0%  99.8%    ALSNGVLFANCISVTCQCQTTGRAISQSGEQTLLMIDNTTCPTAVLGNVIISLGKYLGSVNYNSEGIAIGPPVFTDKVDI
 4 NiV-M4    100.0%  99.5%    ALSNGVLFANCISVTCQCQTTGRAISQSGEQTLLMIDNTTCPTAVLGNVIISLGKYLGSVNYNSEGIAIGPPVFTDKVDI
 5 NiV-C     100.0%  98.9%    ALSNGVLFANCISVTCQCQTTGRAISQSGEQTLLMIDNTTCPTAVLGNVIISLGKYLGSVNYNSEGIAVGPPVFTDKVDI
 6 NiV-B1.1  100.0%  98.5%    ALSNGVLFANCISVTCQCQTTGRAISQSGEQTLLMIDNTTCPTAVLGNVIISLGKYLGSVNYNSEGIAIGPPVFTDKVDI
 7 NiV-B1.2  100.0%  99.1%    ALSNGVLFANCISVTCQCQTTGRAISQSGEQTLLMIDNTTCPTAVLGNVIISLGKYLGSVNYNSEGIAIGPPVFTDKVDI
 8 NiV-B1.3  100.0%  98.9%    ALSNGVLFANCISVTCQCQTTGRAISQSGEQTLLMIDNTTCPTAVLGNVIISLGKYLGSVNYNSEGIAIGPPVFTDKVDI
 9 NiV-B1.4  100.0%  98.7%    ALSNGVLFANCISVTCQCQTTGRAISQSGEQTLLMIDNTTCPTAVLGNVIISLGKYLGSINYNSEGIAIGPPVFTDKVDI
10 NiV-B2.3  100.0%  98.5%    ALSNGVLFANCISVTCQCQTTGRAISQSGEQTLLMIDNTTCPTAVLGNVIISLGKYLGSVNYNSEGIAIGPPVFTDKVDI
11 NiV-B3.1  100.0%  98.2%    ALSNGVLFANCISVTCQCQTTGRAISQSGEQTLLMIDNTTCPTAVLGNVIISLGKYLGSVNYNSEGIAIGPPVFTDKVDI
12 HeV-α1.1   99.8%  88.1%    ALSGGVLFANCISVTCQCQTTGRAISQSGEQTLLMIDNTTCTTVVLGNIIISLGKYLGSINYNSESIAVGPPVYTDKVDI
13 HeV-α2     99.8%  88.3%    ALSGGVLFANCISVTCQCQTTGRAISQSGEQTLLMIDNTTCTTVVLGNIIISLGKYLGSINYNSESIAVGPPVYTDKVDI
14 HeV-β1    100.0%  87.9%    ALSGGVLFANCISVTCQCQTTGRAISQSGEQTLLMIDNTTCTTVVLGNIIISLGKYLGSTNYNSESIAVGPPVYTDRVDI
15 HeV-β2    100.0%  87.7%    ALSGGVLFANCISVTCQCQTTGRAISQSGEQTLLMIDNTTCTTVVLGNIIISLGKYLGSTNYNSESIAVGPPVYTDRVDI
16 CedV-1     99.6%  42.9%    ALTNGVIFANCINTICRCQDNGKTITQNINQFVSMIDNSTCNDVMVDKFTIKVGKYMGRKDINNINIQIGPQIIIDKVDL
17 GhV(+A)    99.6%  51.5%    AISGGVIYANCLSTTCQCYQTGKVIAQDGSQTLMMIDNQTCSIVRIEEILISTGKYLGSQEYNTMHVSVGNPVFTDKLDI
18 AngV       98.0%  39.3%    ALSEGVIYANCLATSCKCATTNKPIVQSTSTVITMIDNSKCPVVEVGKQMISVGSYLGQVSPNNLTIEIGPPVYTEPVDI
19 SHNV1      98.4%  37.7%    ALSQGVIFANCIATTCRCADDGRAISQSSSQTVLLLTSKDCKVYEVQSMMISTGEYLGESIFENTDIPLGPSIVIDKIDI
20 NinV       99.8%  38.0%    ALSGGVIYANCLNTQCACATNGRSISQSSRQTIMMLDDKDCKIYEVGGIFISVSTYMGIGKFENENISIGPPVVVDKIDV
21 SHNV2      99.1%  40.6%    ALSGGVIYANCLSTVCRCIDKDQPISQSLSQTLMMLDNQHCNVYQISNVLISTGRYLGDAEFRNEGIDLGPPIVVDKIDL
22 GakV-1     99.6%  39.9%    ALSGGVIYANCLSAVCRCAVDGVPIVQSLKATVMMLDNKSCRVYQIGELLISTGAYLGSIEFKNENIELGPPIVIDKIDL
23 GakV-2     99.6%  39.9%    ALSGGVIYANCLSAVCRCAVDGVPIVQSLKATVMMLDNKNCRVYQIGELLISTGAYLGSIEFKNENIELGPPIVIDKIDL
24 SHNV3      99.6%  39.6%    ALSGGVIYANCLSAVCRCAVDGVPIVQSLKTTIMMLDNKNCRVYQIGELLISTGAYLGSVEFKNENIELGPPIVVDKIDL
25 ShyV       99.8%  38.7%    ALSGGVIYANCLSAVCRCAVDGVPIVQSLKTTIMMLDNKNCRVYQIGELLISTGSYLGSVEFKNENIELGPPIVVDKIDL
26 SHNV4      99.6%  40.6%    ALSGGVIYANCLSTVCRCAVDGVPIVQSLKATIMMLDNKNCRVYQIGELLISTGAYLGSVEFKNENIDLGPPIVIDKVDL
27 MelV       99.1%  39.7%    ALSDGVVYANCLSTVCRCMDNGVPISQSLKSTVMMLDDKKCTIYQIGDILISVGKYMGHIDYNPENVVLGPPIVIDKIDI
28 DewV       99.1%  40.1%    ALSGGVVYANCLSTVCRCMDNGTPISQGIRHTVALLDNKKCSVYQVGDILISVGKYLGHLDYNTEDIVLGPPIVIDKIDI
29 DarV-K     99.6%  40.6%    ALSGGVIYANCLSTVCRCMDNQKPISQSLRSTVVMLDNKMCKVYQIGDILISVGEYKGAVEYNPEDVHLGPPIVLDKIDI
30 DarV-C     99.6%  40.4%    ALSGGVIYANCLSTVCRCMDNQKPISQSLRSTVVMLDNKMCKVYQIGDILISVGEYKGAVEYNPEDVHLGPPIVLDKIDI
31 LayV-1     98.9%  40.8%    ALSDGLVYANCLNTICRCMDTDTPISQSLGTTVSLLDNKKCLVYQVGDILISVGSYLGEGEYSADNVELGPPVVIDKIDI
32 LayV-2     98.9%  40.8%    ALSDGLVYANCLNTICRCMDTDTPISQSLGTTVSLLDNKKCLVYQVGDILISVGSYLGEGEYSADNVELGPPVVIDKIDI
33 MojV       99.5%  41.2%    ALSDGLVYANCLNTICRCMDTDTPISQSLGATVSLLDNKRCSVYQVGDVLISVGSYLGDGEYNADNVELGPPIVIDKIDI
34 SHNV5-1    99.8%  40.8%    ALSDGVVYANCLNTICRCMDTDTPITQSLRSTVTLLDNKACLVYQIGDILISVGSYLGNTEYNTQNITLGPPIVIDKIDI
35 SHNV5-2    99.8%  40.8%    ALSDGVVYANCLNTICRCMDTDTPITQSLRSTVTLLDNKACLVYQIGDILISVGSYLGNTEYNTQNITLGPPIVIDKIDI

                cov    pid 481         .         5         .         .         .         .         :         . 560
 1 NiV-M1    100.0% 100.0%    SSQISSMNQSLQQSKDYIKEAQRLLDTVNPSLISMLSMIILYVLSIASLCIGLITFISFIIVEKKRNTYSRL--------
 2 NiV-M2    100.0%  99.8%    SSQISSMNQSLQQSKDYIKEAQRLLDTVNPSLISMLSMIILYVLSIASLCIGLITFISFIIVEKKRNTYSRL--------
 3 NiV-M3    100.0%  99.8%    SSQISSMNQSLQQSKDYIKEAQRLLDTVNPSLISMLSMIILYVLSIASLCIGLITFISFIIVEKKRNTYSRL--------
 4 NiV-M4    100.0%  99.5%    SSQKSSMNQSLQQSKDYIKEAQRLLDTVNPSLISMLSMIILYVLSIASLCIGLITFISFIIVEKKRNTYSRL--------
 5 NiV-C     100.0%  98.9%    SSQISSMNQSLQQSKDYIKEAQRLLDTVNPSLISMLSMIILYVLSIASLCIGLITFISFIIVEKKRNTYSRL--------
 6 NiV-B1.1  100.0%  98.5%    SSQISSMNQSLQQSKDYIKEAQRLLDTVNPSLISMLSMIILYVLSIASLCIGLITFISFIIVEKKRNTYSRL--------
 7 NiV-B1.2  100.0%  99.1%    SSQISSMNQSLQQSKDYIKEAQRLLDTVNPSLISMLSMIILYVLSIASLCIGLITFISFIIVEKKRNTYSRL--------
 8 NiV-B1.3  100.0%  98.9%    SSQISSMNQSLQQSKDYIKEAQRLLDTVNPSLISMLSMIILYVLSIASLCIGLITFISFIIVEKKRNTYSRL--------
 9 NiV-B1.4  100.0%  98.7%    SSQISSMNQSLQQSKDYIKEAQRLLDTVNPSLISMLSMIILYVLSIASLCIGLITFISFIIVEKKRNTYSRL--------
10 NiV-B2.3  100.0%  98.5%    SSQISSMNQSLQQSKDYIKEAQRLLDTVNPSLISMLSMIILYVLSIASLCIGLITFISFIIVEKKRNTYSRL--------
11 NiV-B3.1  100.0%  98.2%    SSQISSMNQSLQQSKDYIKEAQRLLDTVNPSLISMLSMIILYVLSIASLCIGLITFISFIIVEKKRNTYSRL--------
```

```
12 HeV-α1.1   99.8%  88.1%     SSQISSMNQSLQQSKDYIKEAQKILDTVNPSLISMLSMIILYVLSIAALCIGLITFISFVIVEKKRGNYSRL--------
13 HeV-α2     99.8%  88.3%     SSQISSMNQSLQQSKDYIKEAQKILDTVNPSLISMLSMIILYVLSIAALCIGLITFISFVIVEKKRGNYSRL--------
14 HeV-β1    100.0%  87.9%     SSQISSMNQSLQQSKDYIKEAQRILDTVNPSLISMLSMIVLYVLSTAALCIGLITLISFIIVEKKRGNYSRL--------
15 HeV-β2    100.0%  87.7%     SSQISSMNQSLQQSKDYIKEAQRILDTVNPSLISMLSMIVLYVLSTAALCIGLITLISFIIVEKKRGNYSRL--------
16 CedV-1      99.6%  42.9%     SNEINKMNQSLKDSIFYLREAKRILDSVNISLISPSVQLFLIIISVLSFIILLIIIVYLYCKSKHSYKYNKFIDDPDYYN
17 GhV(+A)     99.6%  51.5%     TSQISNINQSIEQSKFYLDKSKAILDKINLNLIGSVPISILFIIAILSLILSIITFVIVMIIVRRYNKYTPLIN------
18 AngV        98.0%  39.3%     TNQLGNINETLSKTLDLIKQSNSILDMITGGLNPQIGSMISLILAAIAILLSGITCLFSTKSYVKCNKLQMNC-------
19 SHNV1       98.4%  37.7%     SGQLAEINKTLDHVDNTIKDSNDILDKIDVSTVSAISMVILYVVIALIGFMSALSLLLSVRTFSRCTTLGSQF-------
20 NinV        99.8%  38.0%     SGQLAEVNQSLSKTEELLEESNEYLSQVHVSLVSLKSMIILYVFIGLIGLLAAAALLVSVRSLSMLKTMGPAT-------
21 SHNV2       99.1%  40.6%     GGQIADINQTISDAEEFIEESNKILSKINPKIISVKSMVVLYVVVALIAILAMVSLVLSIRLTMQVGTKINQF-------
22 GakV-1      99.6%  39.9%     GGQIAGINQTLQGVEDYIDKSNEILDQVNPSVTSLGAMIIVYIFIALAILLGLIALIMDVKLNSQVKILINQSMINQMRA
23 GakV-2      99.6%  39.9%     GGQIAGINQTLQGVEDYIDKSNDILDQVNPSVTSLGAMIIVYIFIALAILLGLIALIMDVKLNSQVKILINQSMINQMRA
24 SHNV3       99.6%  39.6%     GGQIAGINQTLQGVEDYIDKSNEILDQVNPSVTSLGAMIIVYIFIALAILIGLIALMIDVKLNSQIKILINQSIINQMRA
25 ShyV        99.8%  38.7%     GGQIAGINQTLQGVEDYIDKSNEILDQVNPSVTSLGAMIIVYIFIALAILIGLIALMIDVKLNSQIKILINQSIINQMRA
26 SHNV4       99.6%  40.6%     GGQIAGINQTLQGVEDYIDKSNEILDQVNPSVTSLGAMIIVYIFIALAILIGLIALMIDVKLNSQIKILINQSIVNQMRA
27 MelV        99.1%  39.7%     GNQLAGINQTLQEAGDFIEKSEEILNSINPSVLTLSSMITLYIFLIITVIIAVAALVLSIRLTIKARILTNQF-------
28 DewV        99.1%  40.1%     GNQLAGINSSLQQAENYIDKSNEILKSINPTIISTNSMITLYIFIIITVIIAITSLVLSLRLTMKAKILTNQF-------
29 DarV-K      99.6%  40.6%     GNQLAGINQTLSEAGDFISKSEEILKDINPAILSVSSMIVLYIFIIVITIISLTSLILSIRLTIKAKIFNNQF-------
30 DarV-C      99.6%  40.4%     GNQLAGINQTLSEAGDFISKSEEILKDINPAILSVSSMIVLYIFIIVITIISLTSLILSIRLTIKAKIFNNQF-------
31 LayV-1      98.9%  40.8%     GNQLAGINQTLQNAEDYIEKSEEFLKGINPSIITLGSMAVLYIFMIVIAVISIIALVLSIKLTVKGNVVRQQF-------
32 LayV-2      98.9%  40.8%     GNQLAGINQTLQNAEDYIEKSEEFLKGINPSIITLGSMAVLYIFMIVIAVISIIALVLSIKLTVKGNVVRQQF-------
33 MojV        99.5%  41.2%     GNQLAGINQTLQEAEDYIEKSEEFLKGVNPSIITLGSMVVLYIFMILIAIVSVIALVLSIKLTVKGNVVRQQF-------
34 SHNV5-1     99.8%  40.8%     GNQLAGINQSLQNAEDYIEKSDEFLKGINPSVITLGSMVVLYIFMILIAIISIIALVLSIRLTTKSNMQKAQF-------
35 SHNV5-2     99.8%  40.8%     GNQLAGINQSLQNAEDYIEKSEEFLKGINPSVITLGSMVVLYIFMILIAIISIIALVLSIRLTTKSNMQKAQF-------

                cov    pid 561          .         .         .         6         .         ] 620
 1 NiV-M1     100.0% 100.0%     -----EDRRVR----PTSSGDLYYIGT---------------------------------
 2 NiV-M2     100.0%  99.8%     -----EDRRVR----PTSSGDLYYIGT---------------------------------
 3 NiV-M3     100.0%  99.8%     -----EDRRVR----PTSSGDLYYIGT---------------------------------
 4 NiV-M4     100.0%  99.5%     -----EDRRVR----PTSSGDLYYIGT---------------------------------
 5 NiV-C      100.0%  98.9%     -----EDRRVR----PTSSGDLYYIGT---------------------------------
 6 NiV-B1.1   100.0%  98.5%     -----EDRRVR----PTSSGDLYYIGT---------------------------------
 7 NiV-B1.2   100.0%  99.1%     -----EDRRVR----PTSSGDLYYIGT---------------------------------
 8 NiV-B1.3   100.0%  98.9%     -----EDRRVR----PTSSGDLYYIGT---------------------------------
 9 NiV-B1.4   100.0%  98.7%     -----EDRRVR----PTSSGDLYYIGT---------------------------------
10 NiV-B2.3   100.0%  98.5%     -----EDRRVR----PTSSGDLYYIGT---------------------------------
11 NiV-B3.1   100.0%  98.2%     -----EDRRVR----PTSNGDLYYIGT---------------------------------
12 HeV-α1.1   99.8%  88.1%     -----DDRQVR----PVSNGDLYYIGT---------------------------------
13 HeV-α2     99.8%  88.3%     -----DDRQVR----PVSNGDLYYIGT---------------------------------
14 HeV-β1    100.0%  87.9%     -----DDRRVR----PVSNGDLYYIGT---------------------------------
15 HeV-β2    100.0%  87.7%     -----DDRRVR----PVSNGDLYYIGT---------------------------------
16 CedV-1      99.6%  42.9%     -----DYKRERINGKASKSNNIYYVGD---------------------------------
17 GhV(+A)     99.6%  51.5%     -----SDPSSR----RSTIQDVYIIPNPGEHSIRSAARSIDRDRD---------------
18 AngV        98.0%  39.3%     ---FKSYERMD----P-----IYHSQQN--------------------------------
19 SHNV1       98.4%  37.7%     -----AYQRQD----P-TLGDVHYAFTSNIPKNQRSKNSNNLSNDLGSTNGSFSSNE---
20 NinV        99.8%  38.0%     -----LMNNIQ----P-TMHNLQYSGHSTINSKSSLINTHQSSHNHKYGSVNGSYTSDES
21 SHNV2       99.1%  40.6%     -----AYTKHV----P-SMENVQYIGTR--------------------------------
22 GakV-1      99.6%  39.9%     GLENPGYSRSL----P-SFGSIASRGSSQELVNTN-------------------------
23 GakV-2      99.6%  39.9%     GLENPGYSRSL----P-SFGSVASRGSSQELVNTN-------------------------
24 SHNV3       99.6%  39.6%     GLENPGYSKSL----P-SFSSTVSKGSSQELINTH-------------------------
25 ShyV        99.8%  38.7%     GLENPGYSRSL----P-SFSSTVSKGSSQELINTH-------------------------
26 SHNV4       99.6%  40.6%     GLENPGYSRSL----P-SFSSIASKGSSQELVNTN-------------------------
27 MelV        99.1%  39.7%     -----AYGRHS----P-SMDNVSYVSK---------------------------------
28 DewV        99.1%  40.1%     -----AYGRHS----P-SMDNVSYVSR---------------------------------
29 DarV-K      99.6%  40.6%     -----AYGRHS----P-SMDNVSYVTH---------------------------------
30 DarV-C      99.6%  40.4%     -----AYGRHS----P-SMDNVSYVTH---------------------------------
31 LayV-1      98.9%  40.8%     -----AYTQHV----P-SMENVNYVSH---------------------------------
32 LayV-2      98.9%  40.8%     -----AYTQHV----P-SMENVNYVSH---------------------------------
33 MojV        99.5%  41.2%     -----TYTQHV----P-SMENINYVSH---------------------------------
34 SHNV5-1     99.8%  40.8%     -----SYTRQA----P-SMDNINYVSR---------------------------------
35 SHNV5-2     99.8%  40.8%     -----SYTRQA----P-SMDNINYVSR---------------------------------
```

MView 1.67, Copyright © 1997-2020 Nigel P. Brown

Supplemental Data 2: Sequence Alignment of HNV-G Proteins

Reference sequence (1): NiV-M3
Identities normalised by aligned length.
Colored by: identity

```
                 cov    pid   1 [        .         .         .         .         :         .         .         . 80
 1 NiV-M3     100.0% 100.0%     ------------MPAENKKVRFENT---------TSDKGKNPSKVIKSYY---GTMDIKK--------------------
 2 NiV-M5     100.0%  99.5%     ------------MPTENKKVRFENT---------TSDKGKIPSKVIKSYY---GTMDIKK--------------------
 3 NiV-B1.5   100.0%  95.7%     ------------MPTESKKVRFENT---------ASDKGKNPSKVIKSYY---GTMDIKK--------------------
 4 NiV-B1.6   100.0%  95.3%     ------------MPTESKKVRFENT---------ASDKGKNPSKVIKSYY---GTMDIKK--------------------
 5 NiV-B2.4   100.0%  95.3%     ------------MPTESKKVRFENT---------ASDKGKNPSKVIKSYY---GTMDIKK--------------------
 6 NiV-B2.5   100.0%  95.8%     ------------MPTESKKVRFENT---------ASDKGKNPSKVIKSYY---GTMDIKK--------------------
 7 NiV-B3.1   100.0%  95.5%     ------------MPTESKKVRFENT---------ASDKGKNPSKVIKSYY---GTMDIKK--------------------
 8 NIV-I1.3   100.0%  95.2%     ------------MPTESKKVRFENT---------ASDKGKNPSKVIKSYY---GTMDIKK--------------------
 9 HeV-α1.2 100.0%  78.1%     ------------MMADSKLVSLNNN---------LSGKIKDQGKVIKNYY---GTMDIKK--------------------
10 HeV-α3   100.0%  78.3%     ------------MMADSKLVSLNNN---------LSGKIKDQGKVIKNYY---GTMDIKK--------------------
11 HeV-α4   100.0%  78.0%     ------------MMADSKLVSLNNN---------LSGKIKDQGKVIKNYY---GTMDIKK--------------------
12 HeV-α5   100.0%  78.3%     ------------MMADSKLVSPNNN---------LSGKIKDQGKVIKNYY---GTMDIKK--------------------
13 HeV-α6   100.0%  78.3%     ------------MMADSKLVSLNNN---------LSGKIKDQGKVIKNYY---GTMDIKK--------------------
14 HeV-β1      99.8%  78.8%     -------------MAESKVVRPSSS---------LSSKIKDQGKIIKNYY---GTMDIKK--------------------
15 HeV-β3      99.8%  79.1%     -------------MAESKVVRPSSS---------LSSKIKDQGKIIKNYY---GTMDIKK--------------------
16 CedV-1      98.0%  28.9%     ------------MLSQLQKNYLDNS-------NQQGDKMNNPDKKLSVNF---NPLELDKGQKDLNKSYYVKNKNYNVSN
17 CedV-2      98.0%  29.1%     ------------MLSQLQKNYLDNS-------NQQGDKMNNPDKKLSVNF---NPLELDKGQKDLNKSYYVKNKNYNISN
18 GhV         98.3%  24.9%     ------------MPQKTVEFINMNSPLERGVSTLSDKKTLNQSKITKQGYFGLGSHSERN--------------------
19 AngV        98.0%  16.9%     -------------MSQKKSLIIKGH-----------DYSPKDKNQSSKYY---NSYESDQ--------------------
20 LayV-2      96.8%  19.3%     -------------MATNKKDVIKTT-------------ESTREDKVKKFY---GVETAEK--------------------
21 LayV-3      96.8%  19.2%     -------------MATNKRDVIKTT-------------ESTREDKVKKFY---GVETAEK--------------------
22 MojV        96.8%  19.6%     -------------MATNRDNTITSA-------------EVSQEDKVKKYY---GVETAEK--------------------
23 SHNV5-1     97.7%  18.9%     MQNRRTSSDPVNTQNSNNRPRIENGGPMNPTMGNQPRIQTARSDPARKYY---GVETAER--------------------
24 SHNV5-3     97.7%  18.7%     MQNRRTSSDLANTQNSNNRPRTENGGPMNSTMGNQPRIQTARSDPVRKYY---GVETAER--------------------
25 DarV-K      97.5%  19.0%     -------------MDKIMANKNNNN-----------NKVNTRETTVKKFY---GVDTAEK--------------------
26 DarV-C      97.5%  19.0%     -------------MDKIMANKNNNN-----------NKVNTRETTVKKFY---GVDTAEK--------------------
27 DewV        96.7%  18.2%     ------------------MANTNTS-----------NKVQTRESTVKKFY---GVDTAEK--------------------
28 MelV        97.0%  18.8%     ----------------MSGPAKQNN-----------PKINTRETNVKKFY---GVETAEK--------------------
29 NinV        97.5%  16.9%     -----------MTQISTGSERTMST------------EKQEFENAARRYY---GVEKAEN--------------------
30 SHNV2       96.3%  16.3%     ------------MTDDNKMVKVGTE-----------------TTTPHKYY---GVEKVDN--------------------
31 GakV-1      96.5%  17.2%     -----------------MSPKIDTN-------------KTAPISDSRKYY---GVDSVER--------------------
32 GakV-2      96.5%  17.1%     -----------------MSPKIDTN-------------KTAPISDSRKYY---GVDSVER--------------------
33 SHNV3       96.3%  17.4%     ------------------MSKVDTS-------------KTTSTTNSRKYY---GVESVEK--------------------
34 ShyV        96.3%  17.1%     ------------------MSKVDTS-------------KTTSTTNSRKYY---GVESVEK--------------------

                 cov    pid  81          .         1         .         .         .         .         :         . 160
 1 NiV-M3     100.0% 100.0%     -INEGLLDS--K-ILSAFNTVIALLGSIVIIVMNIMIIQNYTRSTDNQAVIKDALQGIQQ----QIKGLADKIGTEIGPK
 2 NiV-M5     100.0%  99.5%     -INEGLLDS--K-ILSAFNTVIALLGSIVIIVMNIMIIQNYTRSTDNQAVIKDALQGIQQ----QIKGLADKIGTEIGPK
 3 NiV-B1.5   100.0%  95.7%     -INEGLLDS--K-ILSAFNTVIALLGSIVIIVMNIMIIQNYTRSTDNQAMIKDALQSIQQ----QIKGLADKIGTEIGPK
 4 NiV-B1.6   100.0%  95.3%     -INEGLLDS--K-ILSAFNTVIALLGSIVIIVMNIMIIQNYTRSTDNQAMIKDALQSIQQ----QIKGLADKIGTEIGPK
 5 NiV-B2.4   100.0%  95.3%     -INEGLLDS--K-ILSAFNTVIALLGSIVIIVMNIMIIQNYTRSTDNQAMIKDALQSIQQ----QIKGLADKIGTEIGPK
 6 NiV-B2.5   100.0%  95.8%     -INEGLLDS--K-ILSAFNTVIALLGSIVIIVMNIMIIQNYTRSTDNQAMIKDALQSIQQ----QIKGLADKIGTEIGPK
 7 NiV-B3.1   100.0%  95.5%     -INEGLLDS--K-ILSAFNTVIALLGSIVIIVMNVMIIQNYTRSTDNQAMIKDALQSIQQ----QIKGLADKIGTEIGPK
 8 NIV-I1.3   100.0%  95.2%     -INEGLLDS--K-ILSAFNTVIALLGSIVIIVMNIMIIQNYTRSTDNQAMIKDALQSIQQ----QIKGLADKIGTEIGPK
 9 HeV-α1.2 100.0%  78.1%     -INDGLLDS--K-ILGAFNTVIALLGSIIIIVMNIMIIQNYTRTTDNQALIKESLQSVQQ----QIKALTDKIGTEIGPK
10 HeV-α3   100.0%  78.3%     -INDGLLDS--K-ILGAFNTVIALLGSIIIIVMNIMIIQNYTRTTDNQALIKESLQSVQQ----QIKALTDKIGTEIGPK
11 HeV-α4   100.0%  78.0%     -INDGLLDS--K-ILGAFNTVIALLGSIIIIVMNIMIIQNYTRTTDNQALIKESLQSVQQ----QIKALTDKIGTEIGPK
12 HeV-α5   100.0%  78.3%     -INDGLLDS--K-ILGAFNTVIALLGSIIIIVMNIMIIQNYTRTTDNQALIKESLQSVQQ----QIKALTDKIGTEIGPK
13 HeV-α6   100.0%  78.3%     -INDGLLDS--K-ILGAFNTVIALLGSIIIIVMNIMIIQNYTRTTDNQALIKESLQSVQQ----QIKALTDKIGTEIGPK
14 HeV-β1      99.8%  78.8%     -INDGLLDS--K-ILGAFNTVIALLGSIIIIVMNVMIIQNYARTTDNQALIKDSLQSVQQ----QIKALTDKIGTEIGPK
15 HeV-β3      99.8%  79.1%     -INDGLLDS--K-ILGAFNTVIALLGSIIIIVMNVMIIQNYTRTTDNQALIKDSLQSVQQ----QIKALTDKIGTEIGPK
16 CedV-1      98.0%  28.9%     LLNESLHDI--KFCIYCIFSLLIIITIINIITISI-VITRLKVHEENNGMESPNLQSIQD----SLSSLTNMINTEITPR
17 CedV-2      98.0%  29.1%     LLNESLHDI--KFCIYCIFSLLIIITIINIITISI-VITRLKVHEENNGMESPNLQSIQD----SLSSLTNMINTEITPR
18 GhV         98.3%  24.9%     -WKKQKNQN--D-HYMTVSTMILEILVVLGIMFNLIVLTMVYYQNDNINQRMAELTSNITVLNLNLNQLTNKIQREIIPR
19 AngV        98.0%  16.9%     ---DTINPNEFK-CMLTLIFLVLLLTIASVIVLGICNVVKLDHESKQISINTMSINKAIG----KINELYSLINNELKIR
20 LayV-2      96.8%  19.3%     -VADSISSN--K-VFILMNTLLILTGAIITITLNVTNLTTIKNQQAMLKIIQDEVNSKLE----MFVSLDQLVKGEIKPK
21 LayV-3      96.8%  19.2%     -VADSISSN--K-VFILMNTLLILTGAIITITLNVTNLTTIKNQQAMLKIIQDEVNSKLE----MFVSLDQLVKGEIKPK
22 MojV        96.8%  19.6%     -VADSISGN--K-VFILMNTLLILTGAIITITLNITNLTAAKSQQNMLKIIQDDVNAKLE----MFVNLDQLVKGEIKPK
23 SHNV5-1     97.7%  18.9%     -VADSISSN--K-VFILMNTLLILTGAIITITLNITNLNNAQNQQTMLKMIQDEVDLKLS----AFDNLEQLVKGDLKPK
24 SHNV5-3     97.7%  18.7%     -VADSISSN--K-VFILMNTLLILTGAIITITLNITNLNNAQNQQTMLKMIQDEVDLKLS----VFDNLEQLVKGDLKPK
25 DarV-K      97.5%  19.0%     -VADSIISN--K-IFILINTLLIITSSVITITLNITNLEGIKNQNAIIKTLQMEIMNKVE----TISKLEQIVKGDLKPK
26 DarV-C      97.5%  19.0%     -VADSIISN--K-IFILINTLLIITSSVITITLNITNLEGIKNQNAIIKTLQMEIMNKVE----TISKLEQIVKGDLKPK
27 DewV        96.7%  18.2%     -VADSISNN--K-LFILANSLLILTSTVIIITLNITNLEGIKHQNQVIKTLHDEIQAKID----VISQLETIIKGDLKPK
28 MelV        97.0%  18.8%     -VADSISNN--K-LFILANTLLILTSTVIIITLNITNLEGIKHQNEVIRNLHDEIKSKIE----TINNLEQIIKGDLKPK
29 NinV        97.5%  16.9%     -LAGTILNN--K-MFILMNTLLIITGSLITIGLNATNLTNISYQKTLVNNLDKEMRHKFD----VVSQLDEFLRGEIRPK
30 SHNV2       96.3%  16.3%     -FAGKVVSN--R-IFILTNMILIITSSIVAITLNVTNLESLYNQRETLAMVNQEVHNKLG----DVSTIGNTIKGDIKPK
31 GakV-1      96.5%  17.2%     -FADGVTNN--K-IFILANMLLIIMSSIVMISLNITNLENLNNQKTSLKILNEDVTNKLE----QIDTLDQIVKGEIKPK
32 GakV-2      96.5%  17.1%     -FADGVTNN--K-IFILANMLLIIMSSIVMISLNITNLENLNNQKTSLKILNEDVTNKLE----QIDTLDQIVKGEIKPK
33 SHNV3       96.3%  17.4%     -FADGVTNN--K-IFILANMLLIIMSSIVMISLNITNLENLNNQKTSLKILNDDVNNKLE----QIDTLDQIVKGEIKPK
34 ShyV        96.3%  17.1%     -FADGVTNN--K-IFILANMLLIIMSSIVMISLNITNLENLNNQKTSLKILNEDVNNKLE----QIDTLDQIVKGEIKPK

                 cov    pid 161          .         .         .         2         .         .         .         . 240
 1 NiV-M3     100.0% 100.0%     VSLIDTSSTITIPANIGLLGSKISQSTASINENVNEKCK------------------FTLPPLKIHECNISCPNPLPFRE
```

```
 2 NiV-M5     100.0%  99.5%     VSLIDTSSTITIPANIGLLGSKISQSTASINENVNEKCK-------------------FTLPPLKIHECNISCPNLPFRE
 3 NiV-B1.5   100.0%  95.7%     VSLIDTSSTITIPANIGLLGSKISQSTASINENVNEKCK-------------------FTLPPLKIHECNISCPNLPFRE
 4 NiV-B1.6   100.0%  95.3%     VSLIDTSSTITIPANIGLLGSKISQSTASINENVNEKCK-------------------FTLPPLKIHECNISCPNLPFRE
 5 NiV-B2.4   100.0%  95.3%     VSLIDTSSTITIPANIGLLGSKISQSTASINENVNEKCK-------------------FTLPPLKIHECNISCPNLPFRE
 6 NiV-B2.5   100.0%  95.8%     VSLIDTSSTITIPANIGLLGSKISQSTASINENVNEKCK-------------------FTLPPLKIHECNISCPNLPFRE
 7 NiV-B3.1   100.0%  95.5%     VSLIDTSSTITIPANIGLLGSKISQSTASINENVNEKCK-------------------FTLPPLKIHECNISCPNLPFRE
 8 NIV-I1.3   100.0%  95.2%     VSLIDTSSTITIPANIGLLGSKISQSAASINENVNEKCK-------------------FTLPPLKIHECNISCPNLPFRE
 9 HeV-α1.2 100.0%  78.1%     VSLIDTSSTITIPANIGLLGSKISQSTSSINENVNDKCK--------------------FTLPPLKIHECNISCPNLPFRE
10 HeV-α3   100.0%  78.3%     VSLIDTSSTITIPANIGLLGSKISQSTSSINENVNDKCK--------------------FTLPPLKIHECNISCPNLPFRE
11 HeV-α4   100.0%  78.0%     VSLIDTSSTITIPANIGLLGSKISQSTSSINENVNDKCK--------------------FTLPPLKIHECNISCPNLPFRE
12 HeV-α5   100.0%  78.3%     VSLIDTSSTITIPANIGLLGSKISQSTSSINENVNDKCK--------------------FTLPPLKIHECNISCPNLPFRE
13 HeV-α6   100.0%  78.3%     VSLIDTSSTITIPANIGLLGSKISQSTSSINENVNDKCK--------------------FTLPPLKIHECNISCPNLPFRE
14 HeV-β1     99.8%  78.8%     VSLIDTSSTITIPANIGLLGSKISQSTSSINENVNDKCK--------------------FTLPPLKIHECNISCPNLPFRE
15 HeV-β3     99.8%  79.1%     VSLIDTSSTITIPANIGLLGSKISQSTSSINENVNDKCK--------------------FTLPPLKIHECNISCPNLPFRE
16 CedV-1      98.0%  28.9%     IGILVTATSVTLSSSINYVGTKTNQLVNELKDYITKSCG-------------------FKVPELKLHECNISCADPKISKS
17 CedV-2      98.0%  29.1%     IGILVTATSVTLPSSINYVGTKTNQLVNELKDYITKSCG-------------------FKVPELKLHECNISCADPKISKS
18 GhV         98.3%  24.9%     ITLIDTATTITIPSAITYILATLTTRISELLPSINQKCE-------------------FKTPTLVLNDCRINCTPPLNPSD
19 AngV        98.0%  16.9%     MMVMDTAVNVDIPADISALSMKLTSAITSIEELIQSQTSSVPSNRPSMNQSGGSSGSSQLSNSSRPIPDQGNPNYCNYDK
20 LayV-2      96.8%  19.3%     VSLINTAVSVSIPGQISNLQTKFLQKYVYLEESITKQCTCNP--------LSGIFP--TTKPP-----PPPTDKPDDDTTD
21 LayV-3      96.8%  19.2%     VSLINTAVSVSIPGQISNLQTKFLQKYVYLEESITKQCTCNP--------LSGIFP--TTKPP-----PPPTDKPDDDTTD
22 MojV        96.8%  19.6%     VSLINTAVSVSIPGQISNLQTKFLQKYVYLEESITKQCTCNP--------LSGIFP--TSGPT-----YPPTDKPDDDTTD
23 SHNV5-1     97.7%  18.9%     VTLINSAVSVSIPSQISNLQNKLTQRISSLEDMVGRKCNFNP--------LATIFP--TVEPS-----KPPTTEEDEDTSD
24 SHNV5-3     97.7%  18.7%     VTLINSAVSVSIPSQISNLQNKLTQRISNLEDMVGRKCNFNP--------LATIFP--TVEPS-----KPPTTEEDEDTSD
25 DarV-K      97.5%  19.0%     VTLINSAVSVSIPGQISNLQTKMSQKLKFIEDSVIDQCTCNP--------LSGIFP--SKEPP---KEPHPGTEDDDDTSD
26 DarV-C      97.5%  19.0%     VTLINSAVSVSIPSQISNLQTKMSQKLKFIEDSVIDQCTCNP--------LSGIFP--SKEPP---KEPHPGTEDDDDTSD
27 DewV        96.7%  18.2%     ISLINSAVSVSLPGQITNLQTKLNQKIKLLEDQVQDQCTCNP--------LSGIFP--SRPPQ---PTPSPDDESDDDMSD
28 MelV        97.0%  18.8%     ISLINSAVSVSLPGQISNLQNKMLQKFKTLEDSVIDQCTCNP--------LSGIFP--SKPPH---TSKPDDEEDDNDFTD
29 NinV        97.5%  16.9%     INIISSATTVSLPTQLSQLSQRLMNQIAKLESMIVAQCSGAT---------STGAP--ANDPNKKPGEVDVDAPTLQPPED
30 SHNV2       96.3%  16.3%     VNLINSAVSVSLPAQMSSMLQKMLNKMNGIEDAIDQRCSCST--------LGSLLPGSSQRPP---TVSTPGAGDDDESTD
31 GakV-1      96.5%  17.2%     LTLVNSAVSVTIPSQISNLATKLNNQIKTLEVAVNKQCSGNI--------LGGLL---TPKPPTGSGTTSPDDGNPDDNVD
32 GakV-2      96.5%  17.1%     LTLVNSAVSVTIPSQISNLATKLNNQIKTLEVAVNKQCSGNI--------LGGLL---TPKPPTGSGTTSPDDGNSDDNVD
33 SHNV3       96.3%  17.4%     LTLVNSAVSVTIPSQLSNLATKLNNQIKTLEVAVNKQCSGNI--------LGGLL---TPKPPEGGDVINPDDNSTGDNTD
34 ShyV        96.3%  17.1%     LTLVNSAVSVTIPSQLSNLATKLNNQIKTLEVAVNKQCSGNI--------LGGLL---TPKPPEGGDVINPDDNSTGDNTD

                 cov    pid 241         :         .         .         .         .         3         .         . 320
 1 NiV-M3     100.0% 100.0%     YRPQTEGVSNLVGLPNNIC--LQKT----SNQILKPKLISYTLPVVGQ----SGTCITDPLLAMDEGYFAYSHLERIGSC
 2 NiV-M5     100.0%  99.5%     YRPQTEGVSNLVGLPNNIC--LQKT----SNQILKPKLISYTLPVVGQ----SGTCITDPLLAMDEGYFAYSHLERIGSC
 3 NiV-B1.5   100.0%  95.7%     YKPQTEGVSNLVGLPNNIC--LQKT----SNQILKPKLISYTLPVVGQ----SGTCITDPLLAMDEGYFAYSHLEKIGSC
 4 NiV-B1.6   100.0%  95.3%     YKPQTEGVSNLVGLPNNIC--LQKT----SNQILKPKLISYTLPVVGQ----SGTCITDPLLAMDEGYFAYSHLEKIGSC
 5 NiV-B2.4   100.0%  95.3%     YKPQTEGVSNLVGLPNNIC--LQKT----SNQILKPKLISYTLPIVGQ----SGTCITDPLLAMDEGYFAYSHLEKIGSC
 6 NiV-B2.5   100.0%  95.8%     YKPQTEGVSNLVGLPNNIC--LQKT----SNQILKPKLISYTLPVVGQ----SGTCITDPLLAMDEGYFAYSHLEKIGSC
 7 NiV-B3.1   100.0%  95.5%     YKPQTEGVSNLVGLPNNIC--LQKT----SNQILKPKLISYTLPVVGQ----SGTCITDPLLAMDEGYFAYSHLEKIGSC
 8 NIV-I1.3   100.0%  95.2%     YKPQTEGVSNLVGLPNNIC--LQKT----SNQILKPKLISYTLPVVGQ----SGTCITDPLLAMDEGYFAYSHLEKIGSC
 9 HeV-α1.2 100.0%  78.1%     YRPISQGVSDLVGLPNQIC--LQKT----TSTILKPRLISYTLPINTR----EGVCITDPLLAVDNGFFAYSHLEKIGSC
10 HeV-α3   100.0%  78.3%     YRPISQGVSDLVGLPNQIC--LQKT----TSTILKPRLISYTLPINTR----EGVCITDPLLAVDNGFFAYSHLEKIGSC
11 HeV-α4   100.0%  78.0%     YRPISQGVSDLVGLPNQIC--LQKT----TSTILKPRLISYTLPINTR----EGVCITDPLLAVDNGFFAYSHLEKIGSC
12 HeV-α5   100.0%  78.3%     YRPISQGVSDLVGLPNQIC--LQKT----TSTILKPRLISYTLPINTR----EGVCITDPLLAVDNGFFAYSHLEKIGSC
13 HeV-α6   100.0%  78.3%     YRPISQGVSDLVGLPNQIC--LQKT----TSTILKPRLISYTLPINTR----EGVCITDPLLAVDNGFFAYSHLEKIGSC
14 HeV-β1     99.8%  78.8%     YRPISQGVSDLVGLPNQIC--LQRT----TSTILKPKLISYTLPINTR----EGVCITDPLLTIDNGFFAYSHLEKIGSC
15 HeV-β3     99.8%  79.1%     YRPISQGVSDLVGLPNQIC--LQRT----TSTILKPKLISYTLPINTR----EGVCITDPLLTIDNGFFAYSHLEKIGSC
16 CedV-1      98.0%  28.9%     AMYSTNAYAELAGPPKIFC----------KSVSKDPDFRLKQIDYVIPVQQDRSICMNNPLLDISDGFFTYIHYEGINSC
17 CedV-2      98.0%  29.1%     AMYSTNAYAELAGPPKIFC----------KSVSKDPDFRLKQIDYVIPVQQDRSICMNNPLLDISDGFFTYIHYEGINSC
18 GhV         98.3%  24.9%     GVKMSSLATNLVAHGPSPC----------RNFSSVPTIYYYRIPGLYNRTALDERCILNPRLTISSTKFAYVHSEYDKNC
19 AngV        98.0%  16.9%     PNRPSMSFTSLNIITPTES--AYTEGCHTETVLTSEQFQLVPLNQLLAGDYGEDYCSRYPSLSLRGRRFTYTEERYRGSC
20 LayV-2      96.8%  19.3%     DDKVDTTIKPVEYYKPDGC--NKTN----DHFTMQPGVNFYTVPNLGPSSSSADECYTNPSFSIGSSIYMFSQEIRKTDC
21 LayV-3      96.8%  19.2%     DDKVDTTIKPVEYYKPDGC--NKTN----DHFTMQPGVNFYTVPNLGPSSSSADECYTNPSFSIGSSIYMFSQEIRKTDC
22 MojV        96.8%  19.6%     DDKVDTTIKPIEYPKPDGC--NRTG----DHFTMEPGANFYTVPNLGPASSNSDECYTNPSFSIGSSIYMFSQEIRKTDC
23 SHNV5-1     97.7%  18.9%     DDKVDISIKPIDYKSPINC--SKPN----DHPTMEPGPNFYAVPNLGPSSSNADECYTNPSFSIGSSIYMFSQEIRKTDC
24 SHNV5-3     97.7%  18.7%     DDKVDISIRPIDYKSPMNC--SKPN----DHPTMEPGPNFYAVPNLGPSSSNADECYTNPSFSIGSSIYMFSQEIRKTDC
25 DarV-K      97.5%  19.0%     DDKVDDSIQSFDYPSFTVC--NNSD----ETTSIIPGPNLYAVPNLSIKEDEEDECVTNPSFSVGTSIYMYSQEIRKTDC
26 DarV-C      97.5%  19.0%     DDKVDDSIQSFDYPGFTVC--NNSD----ETTSIIPGPNLYAVPNLSIKEDEEDECVTNPSFSVGTSIYMYSQEIRKTDC
27 DewV        96.7%  18.2%     DDKVDDSIQGIDYPNFMIC--NGTR----NELSITPGQNLYSVPSLTMREDHDDECVVNPSFSVGTSIYMYSQEIRKTDC
28 MelV        97.0%  18.8%     DDKVDDTIQGIDYPTFTTC--NKSD----HSMSITPGPNLYTVPSLTYREDNDDECVVNPSFSIGTSIYMYSQEIRKTDC
29 NinV        97.5%  16.9%     DD--QDSIQMINLSEYPMC--SNQQQYKEPHFNVTVGPIEYT--ELLGLNNSSDYCISSPSLAIEGSTYIYAQQVRKTNC
30 SHNV2       96.3%  16.3%     TD--SGSVTMIDLSAFTKCTLDRSK----PYAAYSLTGVAHNIPGLSVNIADNGDCVSNVAFAISEGIYVYTQEIRKTDC
31 GakV-1      96.5%  17.2%     D--LEGDMRPLNLAELNDC--SRYPQTPNPTFAISPDI--HPMPELTEPIQLKNHCAVFPTVALGESIYVYSHQIRKTPC
32 GakV-2      96.5%  17.1%     D--LEGDMRPLNLAELNDC--SRYPQTPNPTFAISPDI--HPMPELTEPIQLKNHCAVFPTVALGESIYVYSHQIRKTPC
33 SHNV3       96.3%  17.4%     D--LEGDMRPLNLAQLNDC--SNYPQAPQPTFAIDPDL--HPMPELTEPIQLKNHCAVFPTVALGESIYVYSHQIRKTPC
34 ShyV        96.3%  17.1%     D--LEGDMRPLNLAQLNDC--SNYPQAPQPTFAIDPDL--HPMPELTEPIQLKNHCAVFPTVALGESIYVYSHQIRKTPC

                 cov    pid 321         .         .         :         .         .         .         .         4 400
 1 NiV-M3     100.0% 100.0%     SRGVSKQRIIGVGEVLDRGDEVPSLFMTNVWAPPNPNTVYHCSAVYNNEFYYVLCAVST---VGDPILNSTY-WSGSLMM
 2 NiV-M5     100.0%  99.5%     SRGVSKQRIIGVGEVLDRGDEVPSLFMTNVWTPPNPNTVYHCSAVYNNEFYYVLCAVST---VGDPILNSTY-WSGSLMM
 3 NiV-B1.5   100.0%  95.7%     SRGVSKQRIIGVGEVLDRGDEVPSLFMTNVWTPSNPNTVYHCSAVYNNEFYYVLCAVSV---VGDPILNSTY-WSGSLMM
 4 NiV-B1.6   100.0%  95.3%     SRGVSKQRIIGVGEVLDRGDEVPSLFMTNVWTPSNPNTVYHCSAVYNNEFYYVLCAVSV---VGDPILNSTY-WSGSLMM
 5 NiV-B2.4   100.0%  95.3%     SRGVSKQRIIGVGEVLDRGDEVPSLFMTNVWTPSNPNTVYHCSAVYNNEFYYVLCAVSV---VGDPILNSTY-WSGSLMM
 6 NiV-B2.5   100.0%  95.8%     SRGVSKQRIIGVGEVLDRGDEVPSLFMTNVWTPSNPNTVYHCSAVYNNEFYYVLCAVSV---VGDPILNSTY-WSGSLMM
 7 NiV-B3.1   100.0%  95.5%     SRGVSKQRIIGVGEVLDRGDEVPSLFMTNVWTPSNPNTVYHCSAVYNNEFYYVLCAVSV---VGDPILNSTY-WSGSLMM
 8 NIV-I1.3   100.0%  95.2%     SRGVSKQRIIGVGEVLDRGDEVPSLFMTNVWTPSNPNTVYHCSAVYNSEFYYVLCAVSV---VGDPILNSTY-WSGSLMM
 9 HeV-α1.2 100.0%  78.1%     TRGIAKQRIIGVGEVLDRGDKVPSMFMTNVWTPPNPSTIHHCSSTYHEDFYYTLCAVSH---VGDPILNSTS-WTESLSL
10 HeV-α3   100.0%  78.3%     TRGIAKQRIIGVGEVLDRGDKVPSMFMTNVWTPPNPSTIHHCSSTYHEDFYYTLCAVSH---VGDPILNSTS-WTESLSL
```

```
11 HeV-α4   100.0%  78.0%    TRGIAKQRIIGVGEVLDRGDKVPSMFMTNVWTPPNPSTIHHCSSTYHEDFYYTLCAVSH---VGDPILNSTS-WTESLSL
12 HeV-α5   100.0%  78.3%    TRGIAKQRIIGVGEVLDRGDKVPSMFMTNVWTPPNPSTIHHCSSTYHEDFYYTLCAVSH---VGDPILNSTS-WTESLSL
13 HeV-α6   100.0%  78.3%    TRGIAKQRIIGVGEVLDRGDKVPSMFMTNVWTPPNPSTIHHCSSTYHEDFYYTLCAVSH---VGDPILNSTS-WTESLSL
14 HeV-β1    99.8%  78.8%     TRGIAKQRIIGVGEVLDRGDKVPSMFMTNVWTPPNPSTVHHCSSTYHDDFYYTLCAVSH---VGDPILNGTS-WIESLFV
15 HeV-β3    99.8%  79.1%     TRGIAKQRIIGVGEVLDRGDKVPSMFMTNVWTPPNPSTVHHCSSTYHDDFYYTLCAVSH---VGDPILNGTS-WIESLFM
16 CedV-1     98.0%  28.9%     KKSDSFKVLLSHGEIVDRGDYRPSLYLLSSHYHPYSMQVINCVPVTCNQSSFVFCHISN---NTKTLDNSDY-SSDEYYI
17 CedV-2     98.0%  29.1%     KKSDSFKVLLSHGEIVDRGDYRPSLYLLSSHYHPYSMQVINCVPVTCNQSSFVFCHISN---NTKTLDNSDY-SSDEYYI
18 GhV        98.3%  24.9%     TRGFKYYELMTFGEILEGPEKEPRMFSRSFYSPTNAVNYHSCTPIVTVNEGYFLCLECT---SSDPLYKANL-SNSTFHL
19 AngV       98.0%  16.9%     STRTKEAQIIVYGYITDFGYRHPTLMILDTFNPPNYDMIYRCTSVTKLSTGLVICLYTN--MTEFELYESPK--NTDLII
20 LayV-2     96.8%  19.3%     TTGEILSIQIVLGRIVDKGQQGPQASPLLVWSVPNPKIINSCAVAAGDETGWVLCSVTLTAASGEPI---PH-MFDGFWL
21 LayV-3     96.8%  19.2%     TTGEILSIQIVLGRIVDKGQQGPQASPLLVWSVPNPKIINSCAVAAGDETGWVLCSVTLTAASGEPI---PH-MFDGFWL
22 MojV       96.8%  19.6%     TAGEILSIQIVLGRIVDKGQQGPQASPLLVWAVPNPKIINSCAVAAGDEMGWVLCSVTLTAASGEPI---PH-MFDGFWL
23 SHNV5-1    97.7%  18.9%     IAGELQSVQIILGRIVDKGQQGPQASPLLVWSVPNPRTINSCAVAAGDEMGWVLCSVTSTAASGGPN---PH-TFDGFWL
24 SHNV5-3    97.7%  18.7%     IAGELQSVQIILGRIVDKGQQGPQASPLLVWSVPNPRTINSCAVAAGDEMGWVLCSVTLTAASGGPS---PH-TFDGFWL
25 DarV-K     97.5%  19.0%     KSGPLTGIKIILGRIVDKGQFGPQVSPLLVWDVPKPETINSCAVVAEDEVGWALCSITSIANSGEPI---PY-LLSGFKL
26 DarV-C     97.5%  19.0%     KSGPLTGIKIILGRIVDKGQFGPQVSPLLVWDVPKPETINSCAVVAEDEVGWALCSITSIANSGEPI---PY-LLSGFKL
27 DewV       96.7%  18.2%     KQGPLIGIKISLGRVVDKGQSGPQVSPLLVWDVPKPETISSCAVVAGDEIGWALCSTIMTAKSGEPI---PY-MFEGFKL
28 MelV       97.0%  18.8%     KQGPLVGIKITLGRIVDKGLSGPQVSPLLVWEVPKPELTGSCAVAAGNEVGWVLCSTTLAASSGEPI---PN-MFEGFKL
29 NinV       97.5%  16.9%     QFGEIVGIQVVMGRLVDKGRTVPSSSPLLRWEVPAPESDQSCAAAASRGTGWLFCVSGN-TVSGEFLSDGRL---LGVRL
30 SHNV2      96.3%  16.3%     LTGQKISQITTLGRIVDKGQSGPRVSPLSTYLEPDPSSKISCSVAASSGSGWVFCVLTT----GEGVNYSKFGPFIGFTL
31 GakV-1     96.5%  17.2%     KSEDTIHQRVSLGRIVDRGFSGPRASPLSTWDLQETEFLSSCSVAASGEIGWVLCVTTD-KFTRDTVYAGPY---TGLKL
32 GakV-2     96.5%  17.1%     KSEDTTHQRVSLGRIVDRGFSGPRASPLSTWDLQETEFLSSCSVAASGEIGWVLCVTTD-KFTRDTVYAGPY---TGLKL
33 SHNV3      96.3%  17.4%     KSEDTIHQRVSIGRIVDRGFSGPRASPLSTWDLQETGYLSSCSVAASGETGWVLCVTTD-KFTGATVYLGPY---TGLKL
34 ShyV       96.3%  17.1%     KSEDTIHQRVSIGRIVDRGFSGPRASPLSTWDLQETGYLSSCSVAASGETGWVLCVTTD-KFTGTTVYLGPY---TGLKL

                  cov    pid 401         .         .         .         .         :         .         .         . 480
 1 NiV-M3     100.0% 100.0%     TRLAVKPKSNGGGYNQHQLALRSIEKGRYDKVMPYGPSGIKQGDTLYFPAVGFLVRTEFKYNDSNCPITKCQ----YSKP
 2 NiV-M5     100.0%  99.5%     TRLAVKPKSNGGGYNQHQLALRSIEKGRYDKVMPYGPSGIKQGDTLYFPAVGFLVRTEFKYNDSNCPITKCQ----YSKP
 3 NiV-B1.5   100.0%  95.7%     TRLAVKPKNNGESYNQHQFALRNIEKGKYDKVMPYGPSGIKQGDTLYFPAVGFLVRTEFKYNDSNCPIAECQ----YSKP
 4 NiV-B1.6   100.0%  95.3%     TRLAVKPKNNGESYNQHQFALRNIEKGKYDKVMPYGPSGIKQGDTLYFPAVGFLVRTEFTYNDSNCPIAECQ----YSKP
 5 NiV-B2.4   100.0%  95.3%     TRLAVKPKNNGESYNQHQFALRNIEKGKYDKVMPYGPSGIKQGDTLYFPAVGFLVRTEFKYNDSNCPIAECQ----YSRP
 6 NiV-B2.5   100.0%  95.8%     TRLAVKPKSNGESYNQHQFALRNIEKGKYDKVMPYGPSGIKQGDTLYFPAVGFLVRTEFKYNDSNCPIAECQ----YSKP
 7 NiV-B3.1   100.0%  95.5%     TRLAVKPKNNGESYNQHQFALRNIEKGKYDKVMPYGPSGIKQGDTLYFPAVGFLVRTEFKYNDSNCPIAECQ----YSKP
 8 NIV-I1.3   100.0%  95.2%     TRLAVKPKNNGESYNQHQFALRNIEKGMYDKVMPYGPSGIKQGDTLYFPAVGFLVRTEFKYNDSNCPVAKCQ----YSKP
 9 HeV-α1.2 100.0%  78.1%    IRLAVRPKSDSGNYNQKYIAITKVERGKYDKVMPYGPSGIKQGDTLYFPAVGFLPRTEFQYNDSNCPIIHCK----YSKA
10 HeV-α3   100.0%  78.3%    IRLAVRPKSDNGDYNQKYIAITKVERGKYDKVMPYGPSGIKQGDTLYFPAVGFLPRTEFQYNDSNCPIIHCK----YSKA
11 HeV-α4   100.0%  78.0%    IRLAVRPKSDSGDYNQKYITITKVERGKYDKVMPYGPSGIKQGNTLYFPAVGFLPRTEFQYNDSNCPIIHCK----YSKA
12 HeV-α5   100.0%  78.3%    IRLAVRPKSDSGDYNQKYIAINKVERGKYDKVMPYGPSGIKQGDTLYFPAVGFLPRTEFQYNDSNCPIIHCK----YSKA
13 HeV-α6   100.0%  78.3%    IRLAVRPKSDSGDYNQKYIAITKVERGKYDKVMPYGPSGIKQGDTLYFPAVGFLPRTEFQYNDSNCPIIHCK----YSKA
14 HeV-β1    99.8%  78.8%     IRLAVRPKTDGGGYNQKYITMTKIERGKYDKVMPYGPSGIKQGDTLYFPAVGFLPRTEFQYNDSNCPIINCK----YSKA
15 HeV-β3    99.8%  79.1%     IRLAVRPKTDGGGYNQKYITMTKIERGKYDKVMPYGPSGIKQGDTLYFPAVGFLPRTEFQYNDSNCPIINCK----YSKA
16 CedV-1     98.0%  28.9%     TYFN--GIDRPKTKKIPINNMTADNR--YIHFTFSGGGGVCLGEEFIIPVTTVI--NTDVFTHDYCESFNCSVQTGKSLK
17 CedV-2     98.0%  29.1%     TYFN--GIDRPKTKKIPINNMTADNR--YIHFTFSGGGGVCLGEEFIIPVTTVI--NTDVFTHDYCESFNCSVQTGKSLK
18 GhV        98.3%  24.9%     VILR--HNKDEKIVSMPSFNLSTDQE--YVQIIPAEGGGTAESGNLYFPCIGRL--LHKRVTHPLCKKSNCS----RTDD
19 AngV       98.0%  16.9%     FEAF--DGSQHKGTILNSSKFIYLND--IDMFIPGTGGGIYLNKTIYLPGIVFT--TNPPDGNAGCPNSACT----NKDP
20 LayV-2     96.8%  19.3%     YKFE--PDTEVVAYRITGFAYLLDKV--YDSVFIGKGGGIQRGNDLYFQMFGLS--RNRQSIKALCEHGSCLGTGGGGYQ
21 LayV-3     96.8%  19.2%     YKFE--PDTEVVAYRITGFAYLLDKV--YDSVFIGKGGGIQRGNDLYFQMFGLS--RNRQGIKALCEHGSCLGTGGGGYQ
22 MojV       96.8%  19.6%     YKLE--PDTEVVSYRITGYAYLLDKQ--YDSVFIGKGGGIQKGNDLYFQMYGLS--RNRQSFKALCEHGSCLGTGGGGYQ
23 SHNV5-1    97.7%  18.9%     YRLT--PDTDVVSYRISGFAYLLDKV--YDSVYIGKGGGVQKGSDLYFQMYGIS--RNRASFTALCDHGSCPGVGGGGYQ
24 SHNV5-3    97.7%  18.7%     YRLT--PDTDVVSYRISGFAYLLDKV--YDSVYIGKGGGVQKGSDLYFQMYGIS--RNRASFTALCDHGSCPGVGGGGYQ
25 DarV-K     97.5%  19.0%     FKFE--PDSEVVQYVLGSKSFRLDRV--YHTLYVGKGGGAARYDGLYFEGFGVI--TNIGKDQPLCNHGKCSGS--GSYI
26 DarV-C     97.5%  19.0%     FKFE--PDSEVVQYVLGSKSFRLDRV--YHTLYVGKGGGAVRYDGLYFEGFGVI--TNIGKDQPLCNHEKCSGS--GSYT
27 DewV       96.7%  18.2%     FKFE--PDKDVVQYTLAPRAFRLDEK--YHTLNIGKGGGIIRGEELYFTGFGII--TNYKKVDPLCNHGICPGS--GSYL
28 MelV       97.0%  18.8%     YKFE--PEKEVVQYIITKRAFRLDNP--YHTMNIGKGGGIIKEDGLYFQGFGVI--YNSGKVDPLCDHGKCPGV--GSYM
29 NinV       97.5%  16.9%     FTFK--GGSLYHAVYVPQTSIRFSLP--IKTFMVGTGGGVYVDGKLYFVGYGTV--TAPHTGGYLCSFDDCPGI--TDAG
30 SHNV2      96.3%  16.3%     SHIT--EDGDVTTYNISLNVVNSNYA--FKSIFIGPGNGIVRGNNMYFVGYGAL--RDNTNYPSWCIYDGCPAD--PSYN
31 GakV-1     96.5%  17.2%     YKLS--IRGQKEEYSITANNITTDAV--IIALTLTRGSGVPKNNKLIFLGLAAV--RDVDTTGVLCPTWKCENI--NNNV
32 GakV-2     96.5%  17.1%     YKLS--IRGQKEEYSITANNITTDAV--IIALTLTRGSGVPKNNKLIFLGLAAV--RDVDTTGVLCPTWKCDNI--NNNV
33 SHNV3      96.3%  17.4%     YKLS--VRGQKEEYAITANDITSDAT--LIALTLTRGSGVSKNNKLIFLGIAAV--RDADRTGVLCPDWKCDNI--NNNI
34 ShyV       96.3%  17.1%     YKLS--VRGQKEEYAITASDITADTT--VIALTLTRGSGVSKNNKLIFLGLAAV--RDADRTGVLCPDWKCDNI--NNNI

                  cov    pid 481         .         5         .         .         .         .         :         . 560
 1 NiV-M3     100.0% 100.0%     ENCRLSM--GIRPNSHYILRSGLLKYNLSDGENPKVVFIEISDQRLSIGSPSKIYDSLGQPVFYQASFSWDTMIKFGDVL
 2 NiV-M5     100.0%  99.5%     ENCRLSM--GIRPNSHYILRSGLLKYNLSDGENPKVVFIEISDQRLSIGSPSKIYDSLGQPVFYQASFSWDTMIKFGDVL
 3 NiV-B1.5   100.0%  95.7%     ENCRLSM--GIRPNSHYILRSGLLKYNLSDEENSKIVFIEISDQRLSIGSPSKIYDSLGQPVFYQASFSWDTMIKFGDVQ
 4 NiV-B1.6   100.0%  95.3%     ENCRLSM--GIRPSSHYILRSGLLKYNLSDEENSKIVFIEISDQRLSIGSPSKIYDSLGQPVFYQASFSWDTMIKFGDVQ
 5 NiV-B2.4   100.0%  95.3%     ENCRLSM--GIRPNSHYILRSGLLKYNLSDEENSKIVFIEISDQRLSIGSPSKIYDSLGQPVFYQASFSWDTMIKFGDVQ
 6 NiV-B2.5   100.0%  95.8%     ENCRLSM--GIRPNSHYILRSGLLKYNLSDEENSKIVFIEISDQRLSIGSPSKIYDSLGQPVFYQASFSWDTMIKFGDVQ
 7 NiV-B3.1   100.0%  95.5%     ENCRLSM--GIRPNSHYILRSGLLKYNLSDEENSKIVFIEISDQRLSIGSPSKIYDSLGQPVFYQASFSWDTMIKFGDVQ
 8 NIV-I1.3   100.0%  95.2%     ENCRLSM--GIRPNSHYILRSGLLKYNLSDEEDSKIIFIEISDQRLSIGSPSKIYDSLGQPVFYQASFSWDTMIKFGDVQ
 9 HeV-α1.2 100.0%  78.1%    ENCRLSM--GVNSKSHYILRSGLLKYNLSLGGDIILQFIEIADNRLTIGSPSKIYNSLGQPVFYQASYSWDTMIKLGDID
10 HeV-α3   100.0%  78.3%    ENCRLSM--GVNSKSHYILRSGLLKYNLSLGGDIILQFIEIADNRLTIGSPSKIYNSLGQPVFYQASYSWDTMIKLGDVD
11 HeV-α4   100.0%  78.0%    ENCRLSM--GVNSKSHYILRSGLLKYNLSLGGDIILQFIEIADNRLTIGSPSKIYNSLGQPVFYQASYSWDTMIKLGDVD
12 HeV-α5   100.0%  78.3%    ENCRLSM--GVNSKSHYILRSGLLKYNLSLGGDIILQFIEIADNRLTIGSPSKIYNSLGQPVFYQASYSWDTMIKLGDVD
13 HeV-α6   100.0%  78.3%    ENCRLSM--GVNSKSHYILRSGLLKYNLSLGGDITLQFIEIADNRLTIGSPSKIYNSLGQPVFYQASYSWDTMIKLGDVD
14 HeV-β1    99.8%  78.8%     ENCRLSM--GINPRSHYVLRSGLLKYNLSLGEDTRLQFIEIADNRLTIGSPSKIYNSLGQPVFYQASYSWDTMIKLGDVD
15 HeV-β3    99.8%  79.1%     ENCRLSM--GINPRSHYVLRSGLLKYNLSLGEDTRLQFIEIADNRLTIGSPSKIYNSLGQPVFYQASYSWDTMIKLGDVD
16 CedV-1     98.0%  28.9%     EICSESL--RSPTNSSRYNLNGIMIISQNNMTDFKIQLNGITYNKLSFGSPGRLSKTLGQVLYYQSSMSWDTYLKAGFVE
17 CedV-2     98.0%  29.1%     EICSESL--RSPTNSSRYNLNGIMIISQNNMTDFKIQLNGITYNKLSFGSPGRLSKTLGQVLYYQSSMSWDTYLKAGFVE
18 GhV        98.3%  24.9%     ESCLKSY--YNQGSPQHQVVNCLIRIRNAQRDNPTWDVITVDLTNTYPGSRSRIFGSFSKPMLYQSSVSWHTLLQVAEIT
19 AngV       98.0%  16.9%     KLCRKGL--KYIFNNSYYMVNGFALISQKGRKEYEVTVTMIETDVYHFGSRSRVQYFNSRAKYYQAPNGWFSYPIYGNIY
```

```
20 LayV-2       96.8%  19.3%      VLCDRAV--MSFGSEESLISNAYLKVNDVASGKPTIISQTFPPSDSYKGSNGRIYTIGERYGIYLAPSSWNRYLRFGLTP
21 LayV-3       96.8%  19.2%      VLCDRAV--MSFGSEESLISNAYLKVNDVASGKPTIISQTFPPSDSYKGSNGRIYTIGERYGIYLAPSSWNRYLRFGLTP
22 MojV         96.8%  19.6%      VLCDRAV--MSFGSEESLITNAYLKVNDLASGKPVIIGQTFPPSDSYKGSNGRMYTIGDKYGLYLAPSSWNRYLRFGITP
23 SHNV5-1      97.7%  18.9%      TLCDKSM--TVLGSDESLVTSAYLRVGDIYSGKPVIVSQTFPPSDSYRGSNGRIYLIGDSYGIYLAPSSWNRYLRFGVTP
24 SHNV5-3      97.7%  18.7%      TLCDKSM--TVLGSDESLVTSAYLRVGDIYSGKPVIVSQTFPPSDSYRGSNGRIYLIGDSYGIYLAPSSWNRYLRFGVTP
25 DarV-K       97.5%  19.0%      PICLSAM--SMMGDTDFLVVSNIIKVVDANIGKPVITVQTFKIQDTYKGSHGRIYQMDNNYGIYLASSSWNRYLKFGTTN
26 DarV-C       97.5%  19.0%      PICLSAM--SMMGDTDFLVVSNIIKVVDTNIGKPVITVQTFKIQDTYKGSHGRIYQMDNNYGVYLASSSWNRYLKFGTTN
27 DewV         96.7%  18.2%      PVCKSAI--SFMGDEDLIVVDNIIKVVNVDIGTPVIDLKTFKASDTYRGSNGRLYAMGDNFGVYFSPSSWNRYLRFGTSS
28 MelV         97.0%  18.8%      PTCLSAM--SMMGDVDFVVVDNIIKVVDYDIGKPVIDLKTFKISETYRGSNGRIYDMGSKFGLYLASSSWNRFLKFGTSN
29 NinV         97.5%  16.9%      TKCGDALFPPISYDGKAMLVNCLVKVSLDNPEVPSIEVDFFHPGDTYAGSNGRVYKMDRGIGIYISPASWNPHPRFGITR
30 SHNV2        96.3%  16.3%      KYCEWSMRPDYFGRLPGL--NCIITITNYKESNRGILIRAIPNTNSYPAAGGRLYDLGDRVGLYFTTASWQGKLQFALFE
31 GakV-1       96.5%  17.2%      GSCVHSY--RLTADMNNYFMNVVVAVDVTPTGKMTASVSLLPMSESYIGSEGGVIDKPGGYGLMISNKGWFARIRYGQTD
32 GakV-2       96.5%  17.1%      GSCVHSY--RLTADMNNYFMNVVVAVDVTPTGKMTASVSLLPMSESYIGSEGGVIDKPGGYGLMISNKGWFARIRYGQTD
33 SHNV3        96.3%  17.4%      NSCVHSY--RLTSDNNNYFMNVVVTVDVSPAGKMIASVSLLPMSESYIGSEGSVIDKPGGYGLMISNKGWFARIRYGQTE
34 ShyV         96.3%  17.1%      NSCVHSY--RLTSDNNNYFMNVVVTVDVSPAGKMIASISLLPMSESYIGSEGSVIDKPGGYGLMISNKGWFARIRYGQTE

                    cov    pid 561         .         .         .         6         .         .         .         . 640
 1 NiV-M3      100.0% 100.0%      TVNPLVVNWRN--NTVISRPGQSQCPRFNTCPEICWEGVYNDAFLIDRINWISAGVFLDSNQTAENP--VFTVFKDNEIL
 2 NiV-M5      100.0%  99.5%      TVNPLVVNWRN--NTVISRPGQSQCPRFNTCPEICWEGVYNDAFLIDRINWISAGVFLDSNQTAENP--VFTVFKDNEIL
 3 NiV-B1.5    100.0%  95.7%      TVNPLVVNWRD--NTVISRPGQSQCPRFNKCPEICWEGVYNDAFLIDRINWISAGVFLDSNQTAENP--VFTVFKDNEVL
 4 NiV-B1.6    100.0%  95.3%      TVNPLVVNWRD--NTVISRPGQSQCPRFNKCPEVCWEGVYNDAFLIDRINWISAGVFLDSNQTAENP--VFTVFKDNEVL
 5 NiV-B2.4    100.0%  95.3%      TVNPLVVNWRD--NTVISRPGQSQCPRFNKCPEVCWEGVYNDAFLIDRINWISAGVFLDSNQTAENP--VFTVFKDNEVL
 6 NiV-B2.5    100.0%  95.8%      TVNPLVVNWRD--NTVISRPGQSQCPRFNKCPEVCWEGVYNDAFLIDRINWISAGVFLDSNQTAENP--VFTVFKDNEVL
 7 NiV-B3.1    100.0%  95.5%      TVNPLVVNWRD--NTVISRPGQSQCPRFNKCPEVCWEGVYNDAFLIDRINWISAGVFLDSNQTAENP--VFTVFKDNEVL
 8 NIV-I1.3    100.0%  95.2%      TVNPLVVNWRD--NTVISRPGQSQCPRFNTCPEVCWEGVYNDAFLIDRINWISAGVFLDSNQTAENP--VFTVFKDNEVL
 9 HeV-α1.2 100.0%  78.1%      TVDPLRVQWRN--NSVISRPGQSQCPRFNVCPEVCWEGTYNDAFLIDRLNWVSAGVYLNSNQTAENP--VFAVFKDNEIL
10 HeV-α3   100.0%  78.3%      TVDPLRVQWRN--NSVISRPGQSQCPRFNVCPEVCWEGTYNDAFLIDRLNWVSAGVYLNSNQTAENP--VFAVFKDNEIL
11 HeV-α4   100.0%  78.0%      TVDPLRVQWRN--NSVISRPGQSQCPRFNVCPEVCWEGTYNDAFLIDRLNWVSAGVYLNSNQTAENP--VFAVFKDNEIL
12 HeV-α5   100.0%  78.3%      TVDPLRVQWRN--NSVISRPGQSQCPRFNVCPEVCWEGTYNDAFLIDRLNWVSAGVYLNSNQTAENP--VFAVFKDNEIL
13 HeV-α6   100.0%  78.3%      TVDPLRVQWRN--NSVISRPGQSQCPRFNVCPEVCWEGTYNDAFLIDRLNWVSAGVYLNSNQTAENP--VFAVFKDNEIL
14 HeV-β1      99.8%  78.8%      TVDPLKVQWRN--NSVISRPGQSQCPRFNVCPEVCWEGTYNDAFLIDRLNWISAGVYLNSNQTAENP--VFAVFKDNEIL
15 HeV-β3      99.8%  79.1%      TVDPLKVQWRN--NSVISRPGQSQCPRFNVCPEVCWEGTYNDAFLIDRLNWISAGVYLNSNQTAENP--VFAVFKDNEIL
16 CedV-1       98.0%  28.9%      KWKPFTPNWMN--NTVISRPNQGNCPRYHKCPEICYGGTYNDIAPLDLGKDMYVSVILDSDQLAENP--EITVFNSTTIL
17 CedV-2       98.0%  29.1%      KWKPFTPNWMN--NTVISRPNQGNCPRYHKCPEICYGGTYNDIAPLDLGKDMYVSVILDSDQLAENP--EITVFNSTTIL
18 GhV          98.3%  24.9%      DLDKYQLDWLD--TPYISRPGGSECPFGNYCPTVCWEGTYNDVYSLTPNNDLFVTVYLKSEQVAENP--YFAIFSRDQIL
19 AngV         98.0%  16.9%      FEKNQKITFDEANYTVYERYTEGSCPINSSCPAFCHSSGYNDIWANDIDSNITFGIYNSDSHSYGRP--VFFMANQSGIM
20 LayV-2       96.8%  19.3%      DISVRSTTWLKEKDPIMKVLTTCTNTDKDMCPEICNTRGYQDIFPLSEDSSFYTYIGITPSNEGTKS--FVAVKDDAGHV
21 LayV-3       96.8%  19.2%      DISVRSTTWLKEKDPIMKVLTTCTNTDKDMCPEICNTRGYQDIFPLSEDSSFYTYIGITPSNEGTKS--FVAVKDDAGHV
22 MojV         96.8%  19.6%      DISVRSTTWLKSQDPIMKILSTCTNTDRDMCPEICNTRGYQDIFPLSEDSEYYTYIGITPNNGGTKN--FVAVRDSDGHI
23 SHNV5-1      97.7%  18.9%      DISVKSTRWLDAKYPVMYIRSTCTNKVSNMCPEICSTRGYQDIFPLSADSESYTYIGISPKGEGTSS--FVAVRDRDGHH
24 SHNV5-3      97.7%  18.7%      DISVKSTRWLDAKYPVMYIRSTCTNKVSNMCPEICSTRGYQDIFPLSADSESYTYIGISPKGEGTSS--FVAVRDRDGHH
25 DarV-K       97.5%  19.0%      TLIPTEVEWSKLKDPIMDIQNNCHNTNQDMCPAVCSSYGYEDIFPLNIEGNAMTYMSMSRNGEGTSN--FIVARSQDNYV
26 DarV-C       97.5%  19.0%      TLIPTEVEWSKLKDPIMDIQNNCHNTNQDMCPAVCSSYGYEDIFPLNIEGNAMTYMSMSRNGEGTSN--FIVARSQDNYV
27 DewV         96.7%  18.2%      SPVPGTFKWSPLKSPVLAVGTTCHNDNLSMCPQVCHSYGYEDVFPLDVDGTSMTYMSISTDGQRSKA--YVQARSKDGFV
28 MelV         97.0%  18.8%      DMNPDSFKWANFKTPIMNVQTSCRNTNENMCPQVCYGTGYEDIFPLNDDGSSRTYTTITTNGSRTQV--YIRARVNGNNV
29 NinV         97.5%  16.9%      RKSISDISWIG--YASQSRVSTTNCPSNVACPAVCYYPNYFDIYPLDEEGELMTITNYNNPDQADRGDLYTCISTDQEDL
30 SHNV2        96.3%  16.3%      DFPSYKPIYVN--SDIITVRAHGSCRDKNNCPQDCYSPLYADIYPLNLEATLFTSTPIFMGGDNADY--RTVIINSNKIE
31 GakV-1       96.5%  17.2%      RASPQRYEWTD--YMSFETPYYLYCSGGRICPVSCKTWNFTVPTILNPSGSIIIGVKAKSKTGNSAS--MITINTPDEVI
32 GakV-2       96.5%  17.1%      RASPQRYEWTD--YMSFETPYYLYCSGGRICPVSCKTWNFTVPTILNPSGSIIIGVKAKSKTGNSAS--MITINTPDEVI
33 SHNV3        96.3%  17.4%      RTSPQRYDWTD--YMSFETPYYLYCSGGRICPSSCRTWNFTVPTILNPSGEIIIGVKAKSRTGNSMS--MISINTPNEVI
34 ShyV         96.3%  17.1%      RTSPQRYEWTD--YMSFETPYYLYCSGGRMCPSSCRTWNFTVPTILNPSGEIIIGVKAKSKTGNSMS--MISINTPNEVI

                    cov    pid 641         :         .         .         .         .         7         .         . 720
 1 NiV-M3      100.0% 100.0%      YRAQLASEDTNAQKTITNCFLLKNKIWCISLVEIYDTGDNVIRPKLFAVKIPEQC---T---------------------
 2 NiV-M5      100.0%  99.5%      YRAQLASEDTNAQKTITNCFLLKNKIWCISLVEIYDTGDNVIRPKLFAVKIPEQC---T---------------------
 3 NiV-B1.5    100.0%  95.7%      YRAQLASEDTNAQKTITNCFLLRNKIWCISLVEIYDTGDNVIRPKLFAVKIPEQC---T---------------------
 4 NiV-B1.6    100.0%  95.3%      YRAQLASEDTNAQKTITNCFLLKNKIWCISLVEIYDTGDNVIRPKLFAVKIPEQC---T---------------------
 5 NiV-B2.4    100.0%  95.3%      YRAQLASEDTNAQKTITNCFLLKNKIWCISLVEIYDTGDNVIRPKLFAVKIPEQC---T---------------------
 6 NiV-B2.5    100.0%  95.8%      YRAQLASEDTNAQKTITNCFLLKNKIWCISLVEIYDTGDNVIRPKLFAVKIPEQC---T---------------------
 7 NiV-B3.1    100.0%  95.5%      YRAQLASEDTNAQKTITNCFLLKNKIWCISLVEIYDTGDNVIRPKLFAVKIPEQC---T---------------------
 8 NIV-I1.3    100.0%  95.2%      YRAQLASEDTNAQKTITNCFLLKNKIWCISLVEIYDTGDNVIRPKLFAVKIPEQC---T---------------------
 9 HeV-α1.2 100.0%  78.1%      YQVPLAEDDTNAQKTITDCFLLENVIWCISLVEIYDTGDSVIRPKLFAVKIPAQC---SES-------------------
10 HeV-α3   100.0%  78.3%      YQVPLAEDDTNAQKTITDCFLLENVIWCISLVEIYDTGDSVIRPKLFAVKIPAQC---SES-------------------
11 HeV-α4   100.0%  78.0%      YQVPLAEDDTNAQKTITDCFLLENVIWCISLVEIYDTGDSVIRPKLFAVKIPAQC---SES-------------------
12 HeV-α5   100.0%  78.3%      YQVPLAEDDTNAQKTITDCFLLENVIWCISLVEIYDTGDSVIRPKLFAVKIPAQC---SES-------------------
13 HeV-α6   100.0%  78.3%      YQVPLAEDDTNAQKTITDCFLLENVIWCISLVEIYDTGDSVIRPKLFAVKIPAQC---SES-------------------
14 HeV-β1      99.8%  78.8%      YQVQLADEDTNAQRTITDCFLLDNVIWCISLVEIYDTGDSVIRPKLFAVKIPAQC---SGN-------------------
15 HeV-β3      99.8%  79.1%      YQVQLADEDTNAQRTITDCFLLDNVIWCISLVEIYDTGDSVIRPKLFAVKIPAQC---SGN-------------------
16 CedV-1       98.0%  28.9%      YKERVSKDELNTRSTTTSCFLFLDEPWCISVLETNRFNGKSIRPEIYSYKIPKYC-------------------------
17 CedV-2       98.0%  29.1%      YKERVSKDELNTRSTTTSCFLFLDEPWCISVLETNRFNGKSIRPEIYSYKIPKYC-------------------------
18 GhV          98.3%  24.9%      KEFPLDAWISSARTTTISCFMFNNEIWCIAALEITRLNDDIIRPIYYSFWLPTDC---RTPYPHTGKM----TRVPLRST
19 AngV         98.0%  16.9%      YEFVIPGSIIAAGQSTTSCLLFYQEVWCMNVIEIRKTLNSSQEIYGFWTKTPLVC---PTKDQYDIAH----AFPLPTPS
20 LayV-2       96.8%  19.3%      ASITILPNYYSITSATISCFMYKEEIWCIAVTEGRKQKENPQRIYAHSYRVQKMC---FNIKPASVVT----SLPSNVTI
21 LayV-3       96.8%  19.2%      ASITILPNYYSITSATISCFMYKEEIWCIAVTEGRKQKENPQRIYAHSYRVQKMC---FNIKPASVVT----SLPSNVTI
22 MojV         96.8%  19.6%      ASIDILQNYYSITSATISCFMYKDEIWCIAITEGKKQKDNPQRIYAHSYKIRQMC---YNMKSATVTV----GNAKNITI
23 SHNV5-1      97.7%  18.9%      ASKEILSSYFSILSSTISCFTYKEEIWCIAVSEGKRTKGSAQRIFAHSYRVIRGC---GHNLVYPNMT----DIVPSFSK
24 SHNV5-3      97.7%  18.7%      ASKEILSSYFSILSSTISCFTYKEEIWCIAVSEGKRTKGSAQRIFAHSYRVIRGC---GHNLVYPNMT----DIVSSFSK
25 DarV-K       97.5%  19.0%      NTMKALGEYFKIITATTSCFTFKDEAWCIVVNEGQHKKGDKQRIFAHSYKLKKSC---IKRSKITDLA----TTSITFNN
26 DarV-C       97.5%  19.0%      NTMKALGEYFKIITATTSCFTFKDEAWCIVVNEGQHKKGDKQRIFAHSYKLKKSC---IKRSRITDLA----TTSITINN
27 DewV         96.7%  18.2%      NSIPALTDYYNIYTSTISCFMYRNDVWCIVINEASLKKNDKQRIYAHSYKLKKEC---VKSDMLMTDM----VTLMQNNK
28 MelV         97.0%  18.8%      GSIQVLNDYYFVYSATTSCFMFKNEGWCIVINEAKLNKNDKQRIYAHSYRLKRLCMSGMRSINSTTGL----TTVVLPSN
```

```
29 NinV        97.5%  16.9%    NRVLDSNVKREVTYTTTSCFVYRRDVWCIVIIVEKSEVREYTEIKPYIYKIKKSC---SLHQGHDAGLRDEEVKVVPLPT
30 SHNV2       96.3%  16.3%    SLVQMFVNYKSIMGTTTTCFMYKDEPWCIVTVRGVRKGYSGVEAISYSYKIKKTC---QNTQPKIGNI----SIPDFPSI
31 GakV-1      96.5%  17.2%    DQYEVFNDYQSIGSTITKCFSYKRQPWCLVLLEGVLKSTGVTETSIQTFKIFRSC---VKHRTYLDSLGTRFYYTVSDNG
32 GakV-2      96.5%  17.1%    DQYEVFNDYQSIGSTITKCFSYKRQPWCLVLLEGVLKSTGVTETSIQTFKIFRSC---VKHRTYLDSLGTRFYYTVSDNG
33 SHNV3       96.3%  17.4%    DQYEVFTDYLSIGSTITKCFLYKRQPWCLVLLEGVPKSTGLTETSIQTFRIYKSC---VKSRTYLDSLGSRYYYVSDQGG
34 ShyV        96.3%  17.1%    DQYEVFKDYLSIGSTITKCFLYKRQPWCLVLLEGVPKSTGQTETSIQTFRIYKSC---VKSRTYLDSLGARYFYVPDQES

                 cov    pid 721         .         .         :         .         ] 770
 1 NiV-M3     100.0% 100.0%    --------------------------------------------------
 2 NiV-M5     100.0%  99.5%    --------------------------------------------------
 3 NiV-B1.5   100.0%  95.7%    --------------------------------------------------
 4 NiV-B1.6   100.0%  95.3%    --------------------------------------------------
 5 NiV-B2.4   100.0%  95.3%    --------------------------------------------------
 6 NiV-B2.5   100.0%  95.8%    --------------------------------------------------
 7 NiV-B3.1   100.0%  95.5%    --------------------------------------------------
 8 NIV-I1.3   100.0%  95.2%    --------------------------------------------------
 9 HeV-α1.2 100.0%  78.1%    --------------------------------------------------
10 HeV-α3   100.0%  78.3%    --------------------------------------------------
11 HeV-α4   100.0%  78.0%    --------------------------------------------------
12 HeV-α5   100.0%  78.3%    --------------------------------------------------
13 HeV-α6   100.0%  78.3%    --------------------------------------------------
14 HeV-β1     99.8%  78.8%    --------------------------------------------------
15 HeV-β3     99.8%  79.1%    --------------------------------------------------
16 CedV-1      98.0%  28.9%    --------------------------------------------------
17 CedV-2      98.0%  29.1%    --------------------------------------------------
18 GhV         98.3%  24.9%    YNY-----------------------------------------------
19 AngV        98.0%  16.9%    PAPAPSQSVPVTEASTITLIQSVEPQGTPTDIPSTTNRDDIQTITNATSG
20 LayV-2      96.8%  19.3%    RS------------------------------------------------
21 LayV-3      96.8%  19.2%    RS------------------------------------------------
22 MojV        96.8%  19.6%    RRY-----------------------------------------------
23 SHNV5-1     97.7%  18.9%    --------------------------------------------------
24 SHNV5-3     97.7%  18.7%    --------------------------------------------------
25 DarV-K      97.5%  19.0%    RSIR----------------------------------------------
26 DarV-C      97.5%  19.0%    RSIR----------------------------------------------
27 DewV        96.7%  18.2%    SRTR----------------------------------------------
28 MelV        97.0%  18.8%    RRTR----------------------------------------------
29 NinV        97.5%  16.9%    EKPKPEARSSYSFWF-----------------------------------
30 SHNV2       96.3%  16.3%    KPVR----------------------------------------------
31 GakV-1      96.5%  17.2%    NKTKQTYIPGSDT-------------------------------------
32 GakV-2      96.5%  17.1%    NKTKQTYIPGSDT-------------------------------------
33 SHNV3       96.3%  17.4%    NRTRQAYKLTSDT-------------------------------------
34 ShyV        96.3%  17.1%    NRTRQAYKLTSDT-------------------------------------
```

MView 1.67, Copyright © 1997-2020 Nigel P. Brown
