# Supplementary material for: Structural and antigenic characterization of novel and diverse Henipavirus glycoproteins
Source: bioRxiv. 2024 Dec 11:2024.12.11.627382. Preprint. [Version 1] doi: 10.1101/2024.12.11.627382 (PMC11661166; doi:10.1101/2024.12.11.627382)
Supplement: Supplementary file 1 [file media-1.pdf]

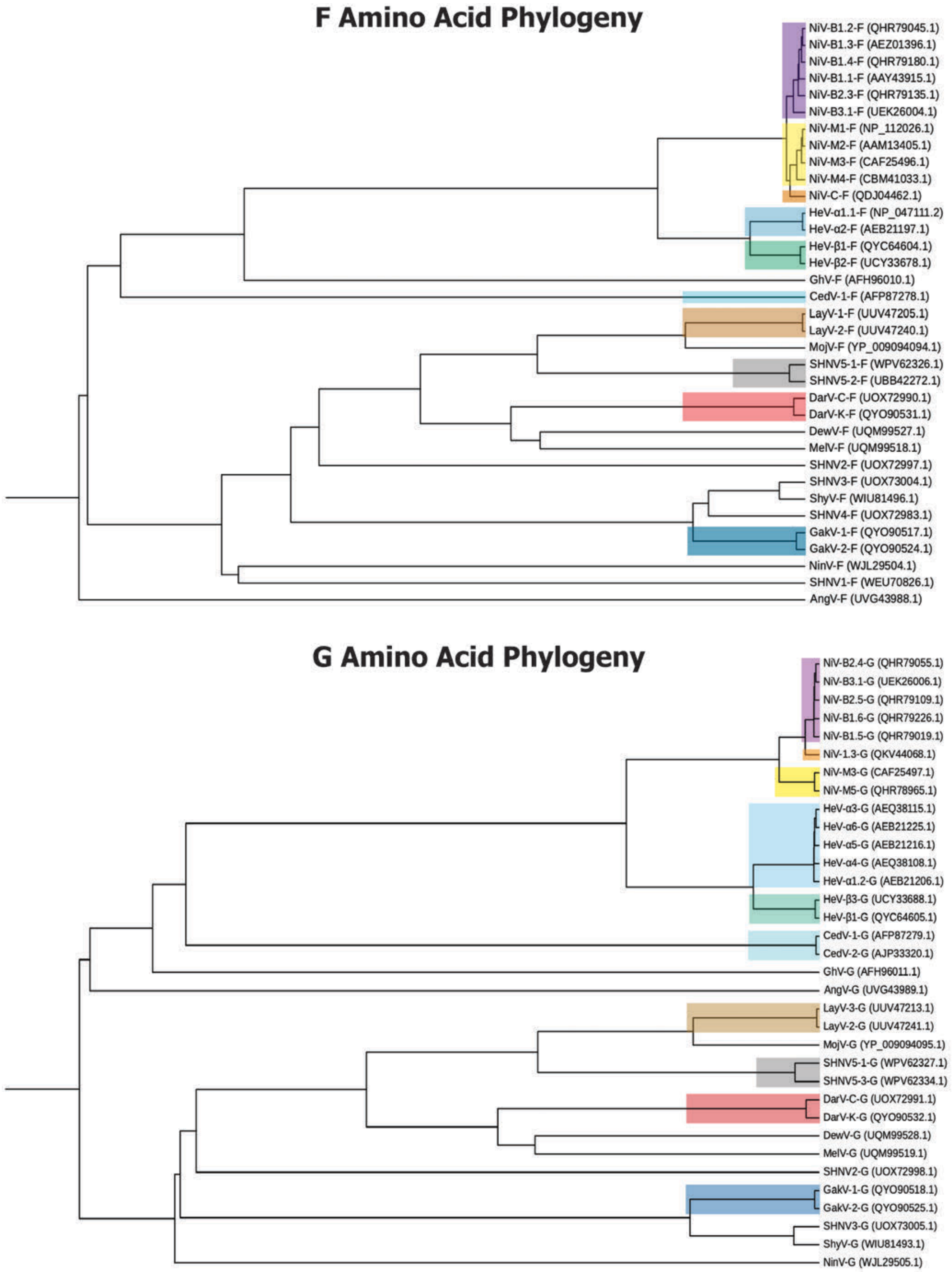

Supplemental Figure 1: Amino Acid-based Phylogeny of HNV Species. Phylogenetic trees constructed using either F or G amino acid sequences. Genbank amino acid accession codes listed next to each tip. Color scheme is continued from Figure 1.

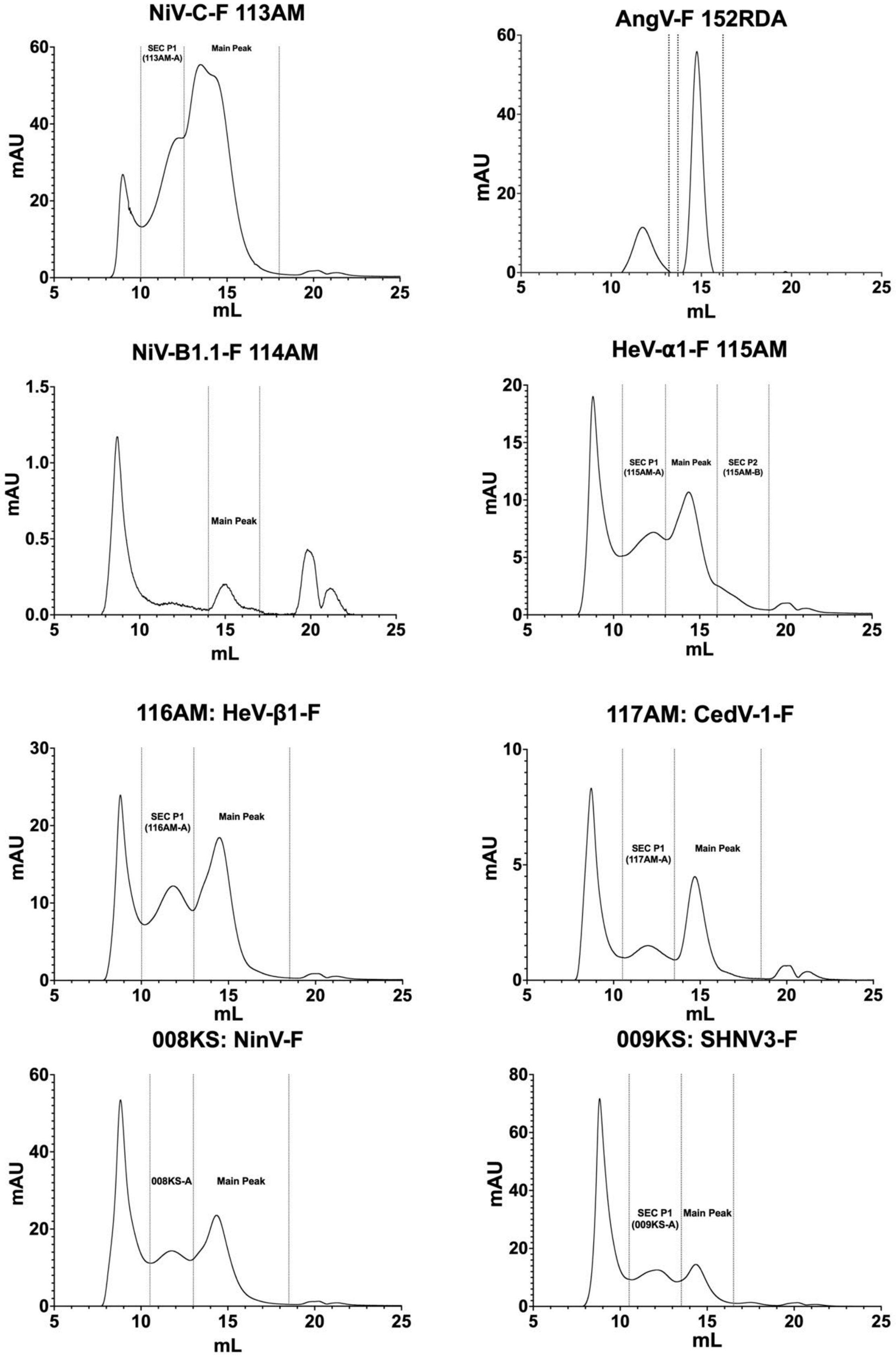

Supplemental Figure 2: Size Exclusion Chromatography (SEC) profiles of HNV F ectodomains.

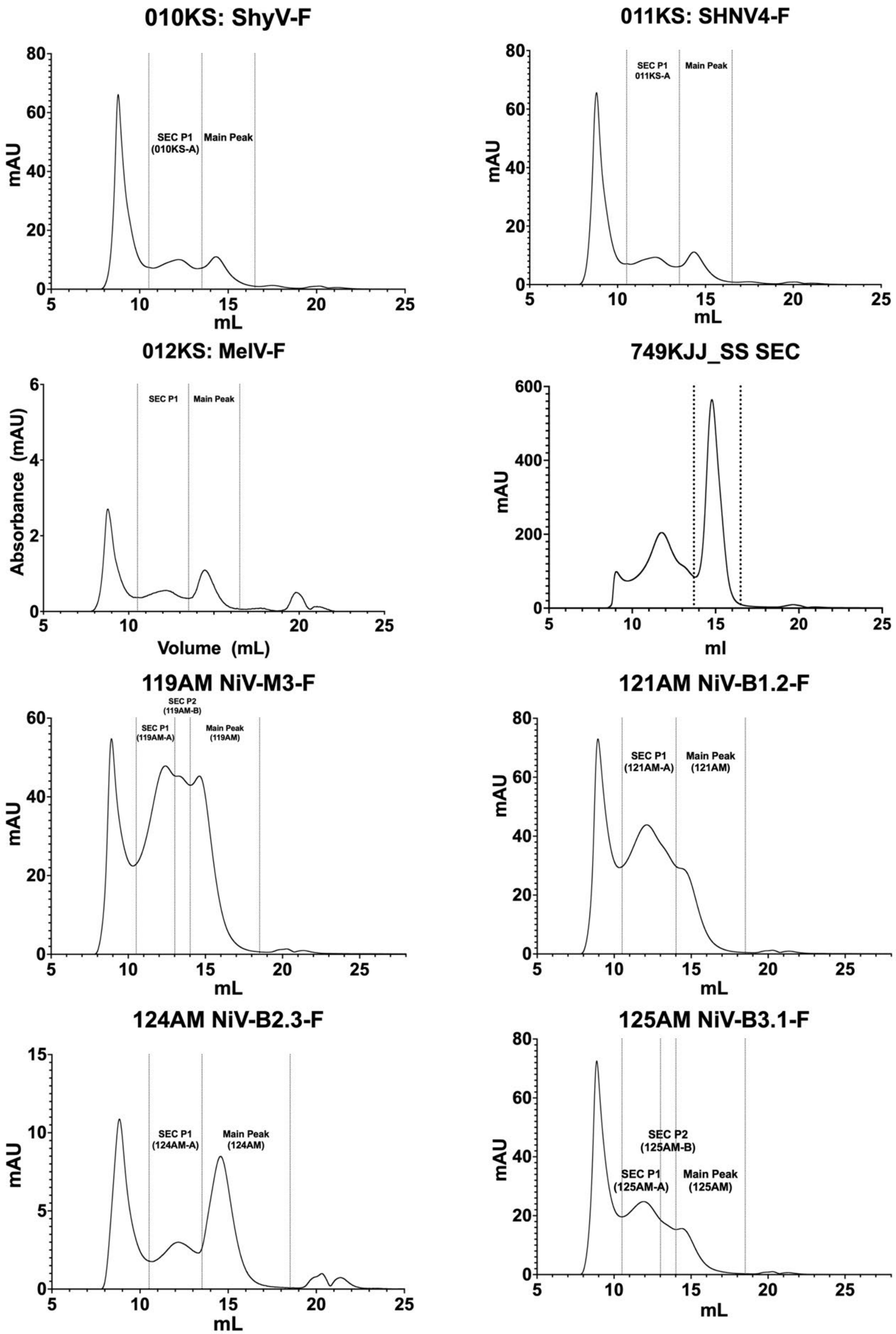

Supplemental Figure 2 (continued): Size Exclusion Chromatography (SEC) profiles of HNV F ectodomains.

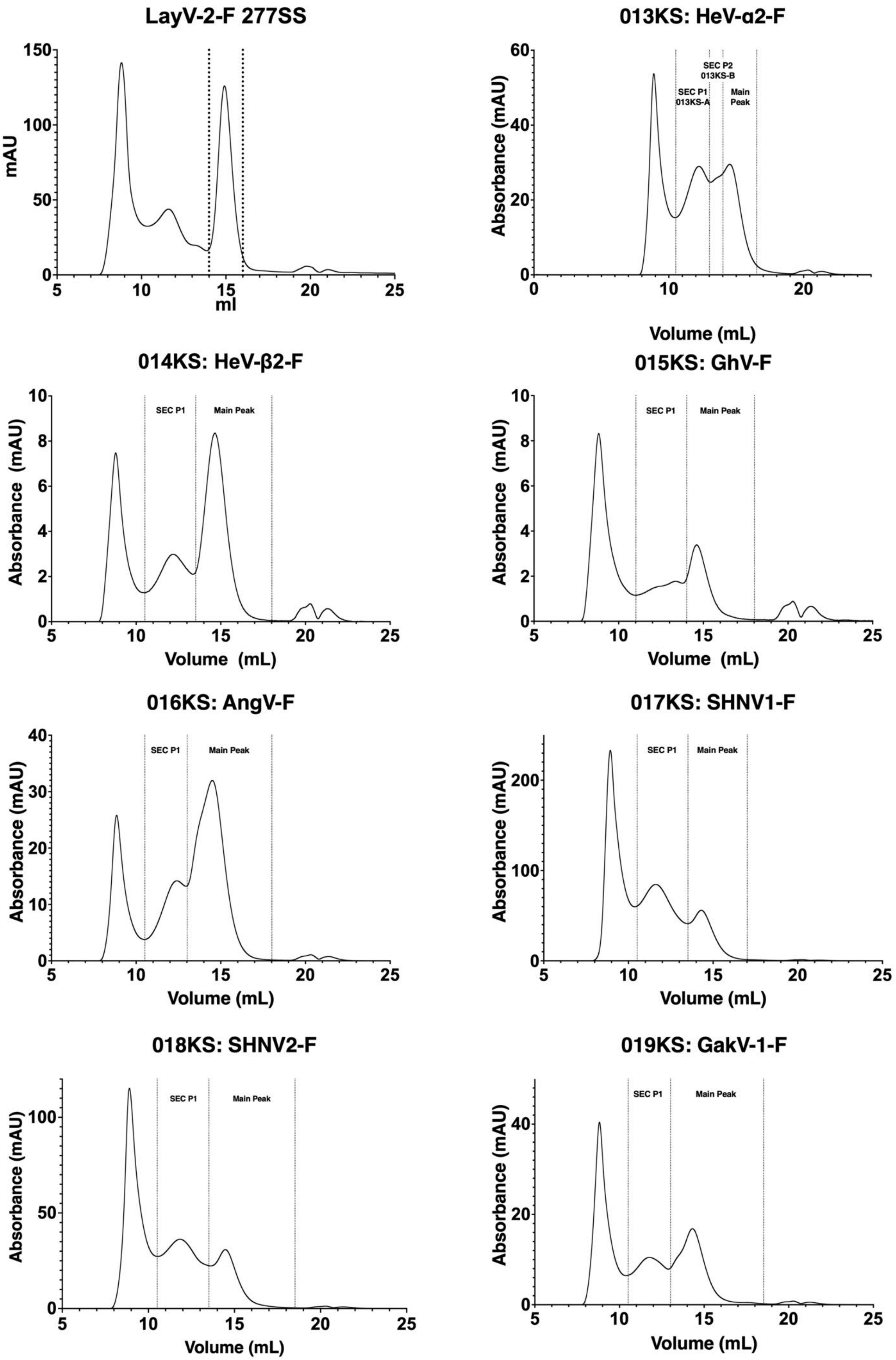

Supplemental Figure 2 (continued): Size Exclusion Chromatography (SEC) profiles of HNV F ectodomains.

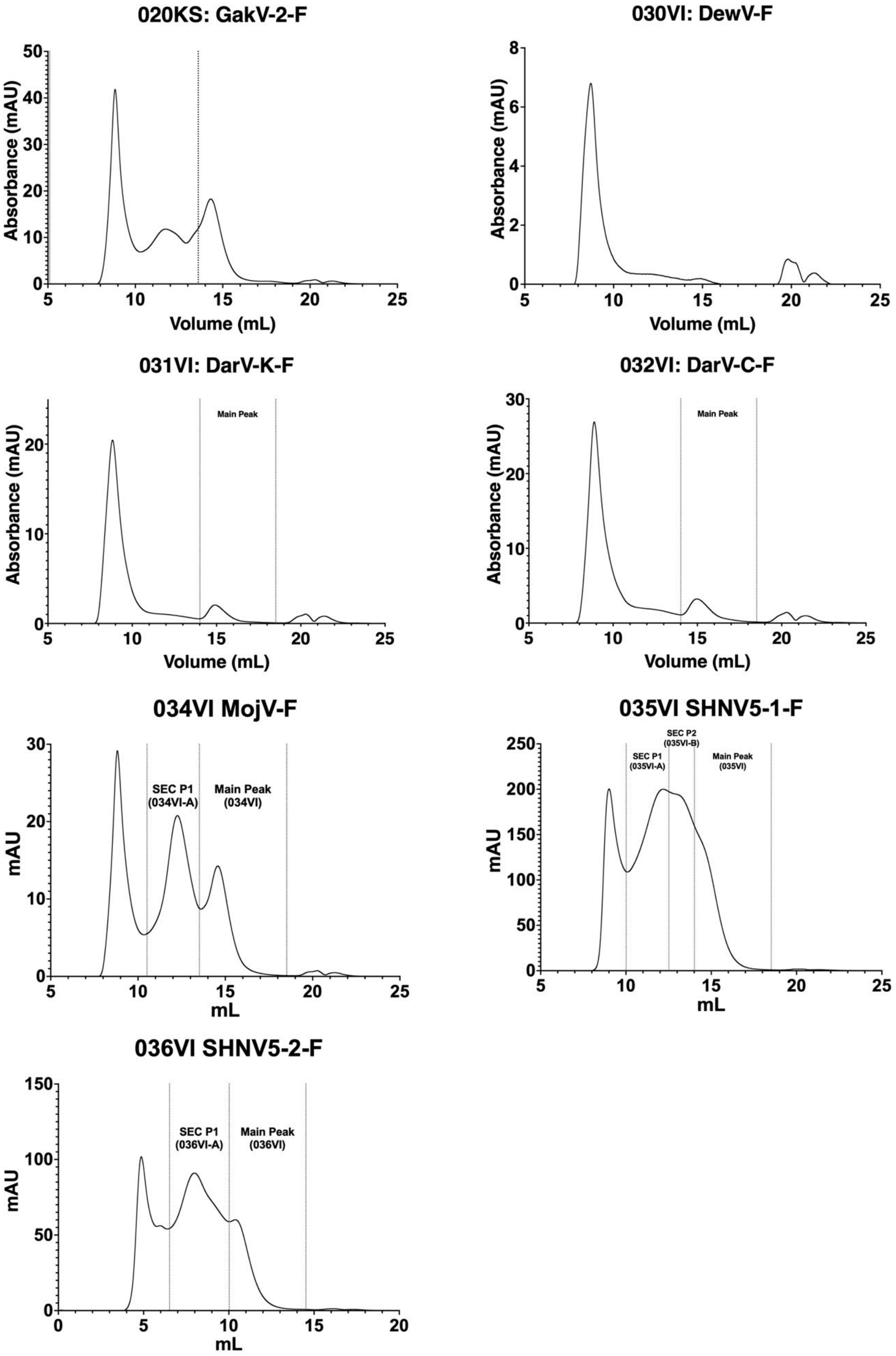

Supplemental Figure 2 (continued): Size Exclusion Chromatography (SEC) profiles of HNV F ectodomains.

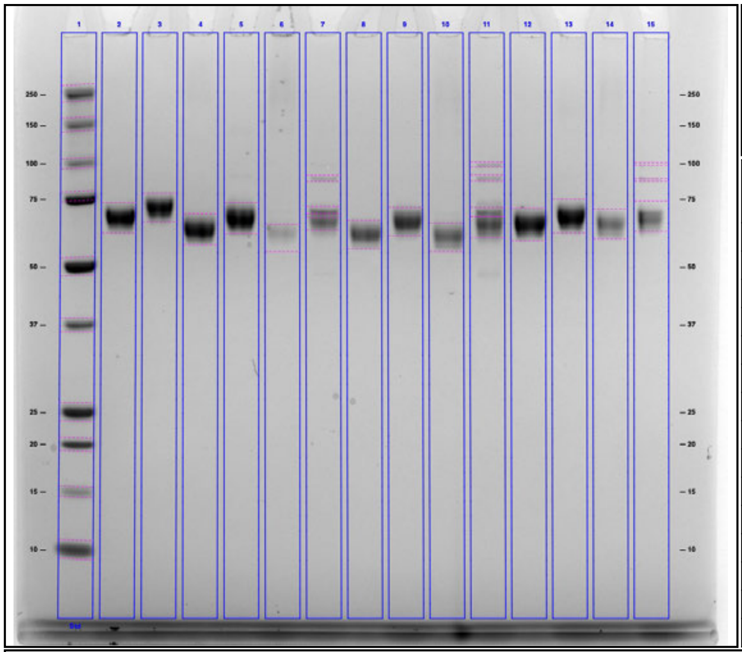

Protocol: 15 µL of each sample was loaded both reduced and non-reduced. The sample was boiled @ 95˚C for 3 min.
DTT: 1M DTT
Protein Marker: Precision Plus Protein Unstained Standards
Sample Buffer: 4X Laemmli Sample buffer
Gel: NuPAGE 4-12% BT 1.0, 15-well combs

Lane	Sample Name	Amount Loaded (µg)	Volume Loaded (µL)	Reduced (R/NR)	Molecular Weight (kDa)
1	Ladder	N/A	4	N/A	N/A
2	117AM: CedV-1-F	1	15	NR	67.3
3	117AM: CedV-1-F	1	15	R	71.5
4	123AM: NiV-B1.4-F	0.68	15	NR	62.4
5	123AM: NiV-B1.4-F	0.68	15	R	66.8
6	123AM-A: NiV-B1.4-F SEC P1	1	15	NR	61.8
7	123AM-A: NiV-B1.4-F SEC P1	1	15	R	88.9, 69.5, 65.8
8	124AM: NiV-B2.3-F	1	15	NR	60.8
9	124AM: NiV-B2.3-F	1	15	R	65.5
10	124AM-A: NiV-B2.3-F SEC P1	0.81	15	NR	60.3
11	124AM-A: NiV-B2.3-F SEC P1	0.81	15	R	99.2, 88.9, 68.9, 64.7
12	014KS: HeV-β2-F	1	15	NR	64.7
13	014KS: HeV-β2-F	1	15	R	67.5
14	014KS-A: HeV-β2-F SEC P1	1	15	NR	64.9
15	014KS-A: HeV-β2-F SEC P1	1	15	R	98.9, 88.3, 67.1

Notes: NR: 3.75 µL Dye, Protein, PBS
R: 3.75 µL dye, 4.5 µL DTT , Protein, PBS

Supplemental Figure 3: SDS-PAGE profiles of HNV F ectodomains. Representative example of an SDS-PAGE profile shown for HNV F ectodomains. Lane 2 and 3: CedV-1-F (NR= Non-reduced; R= Reduced). Lanes 4 and 5: Main (trimer) peak of NiV-B1.4-F. Lanes 6 and 7: Aggregate of trimers (Middle peak; P1) of NiV-B1.4-F. Lanes 8-11: NiV-B2.3-F, same order of samples as for NiV-B1.4-F. Lanes 12-15: Hev-ß2-F, same order of samples as for NiV-B1.4-F.

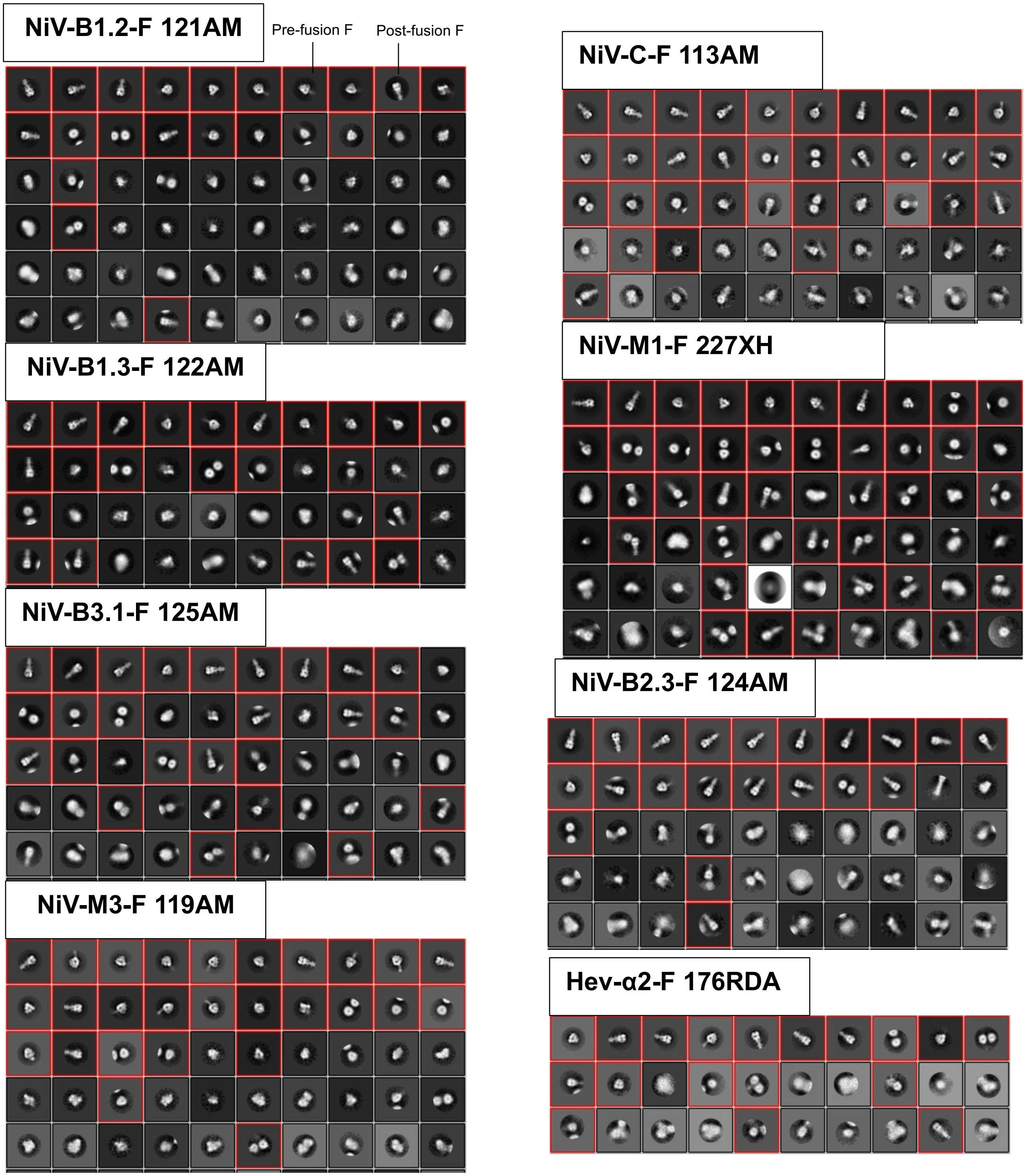

Supplemental Figure 4: NSEM analysis of HNV F ectodomains. Representative 2D classes are shown. Classes containing the pre-fusion or post-fusion F protein can be identified by their shapes (as shown for NiV-B1.2-F as representative examples). The classes that correspond to the F proteins are identified by their typical shape and distinguishable from junk classes.

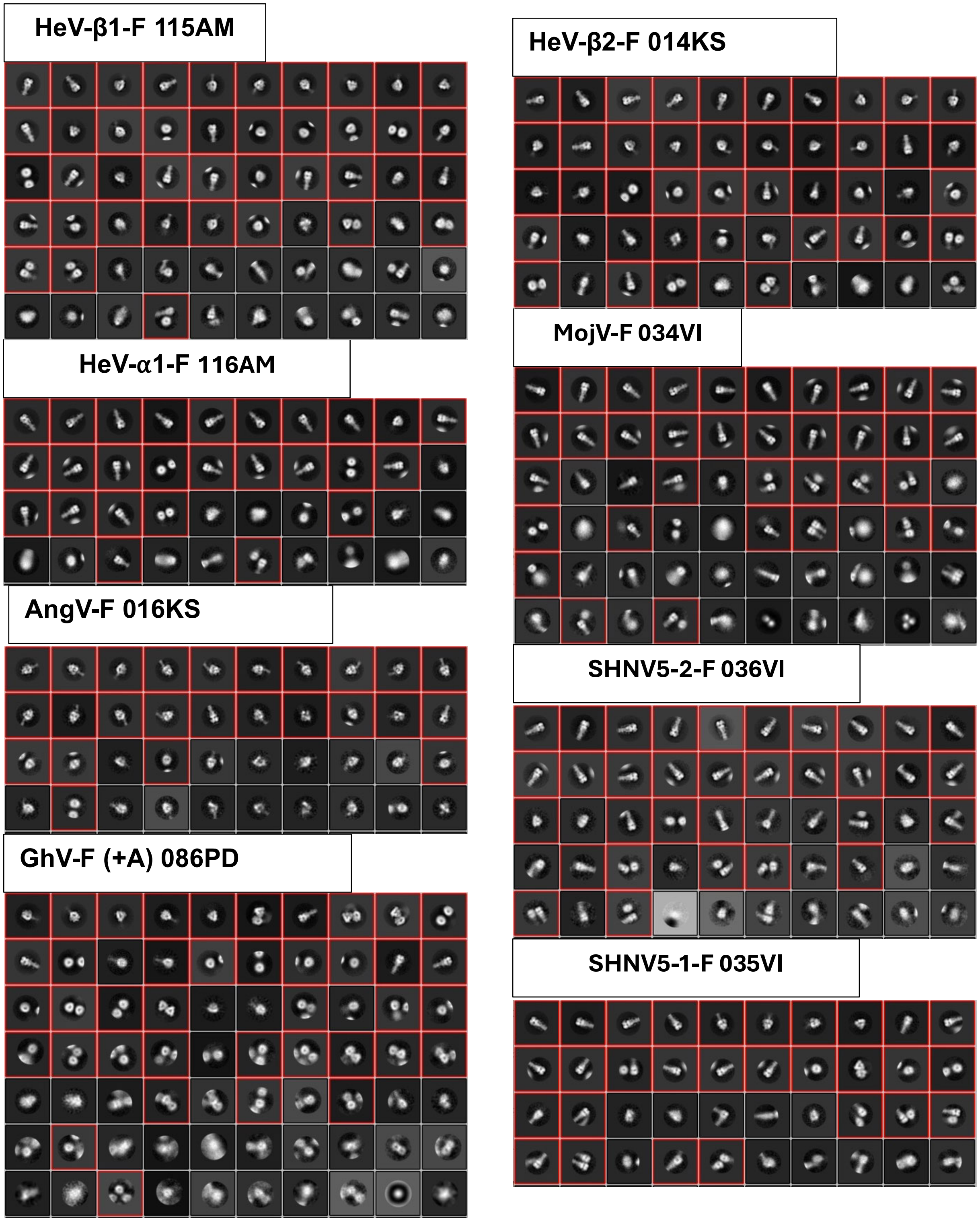

Supplemental Figure 4 (continued): NSEM analysis of HNV F ectodomains. Representative 2D classes are shown.

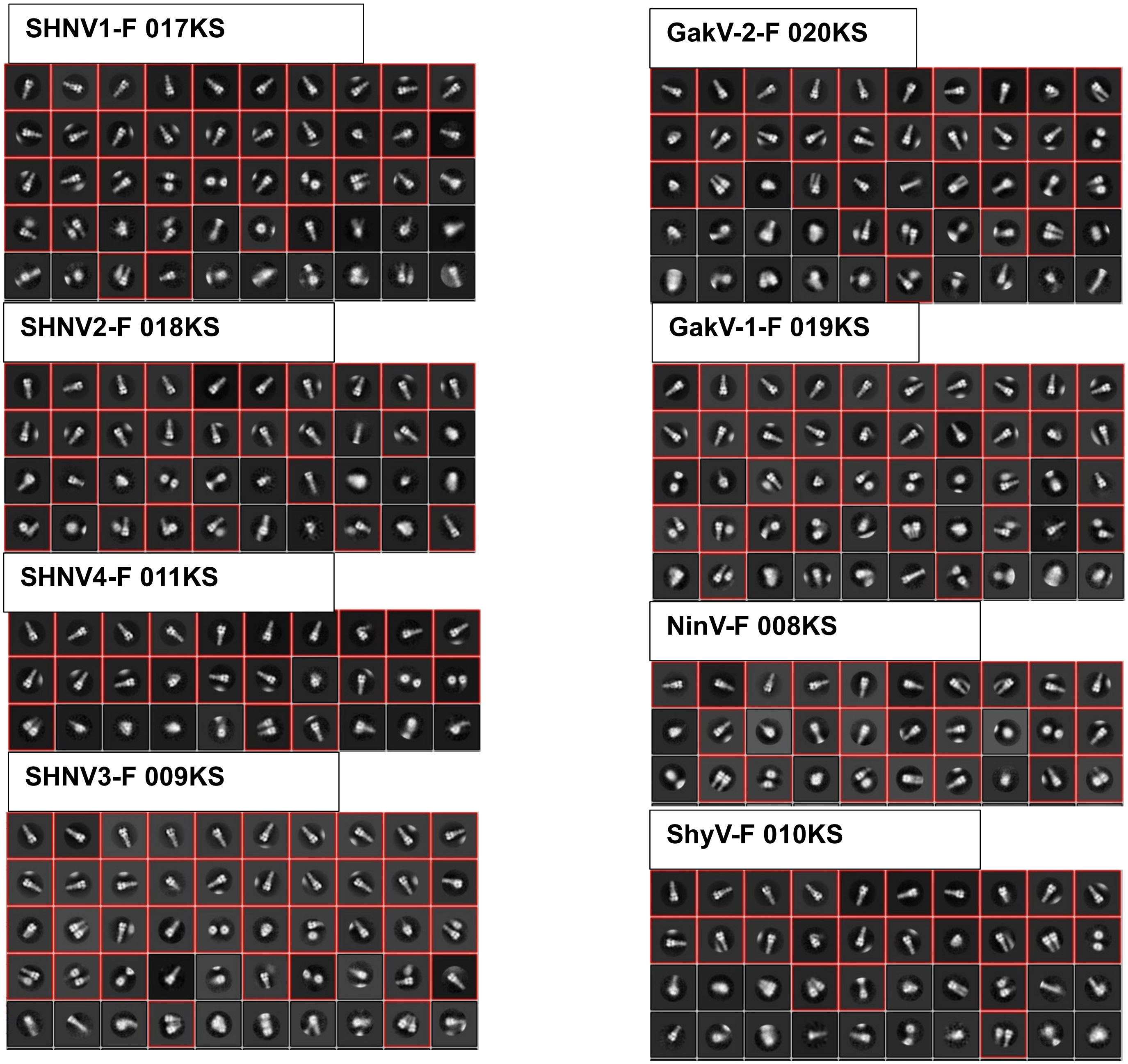

Supplemental Figure 4 (continued): NSEM analysis of HNV F ectodomains. Representative 2D classes are shown.

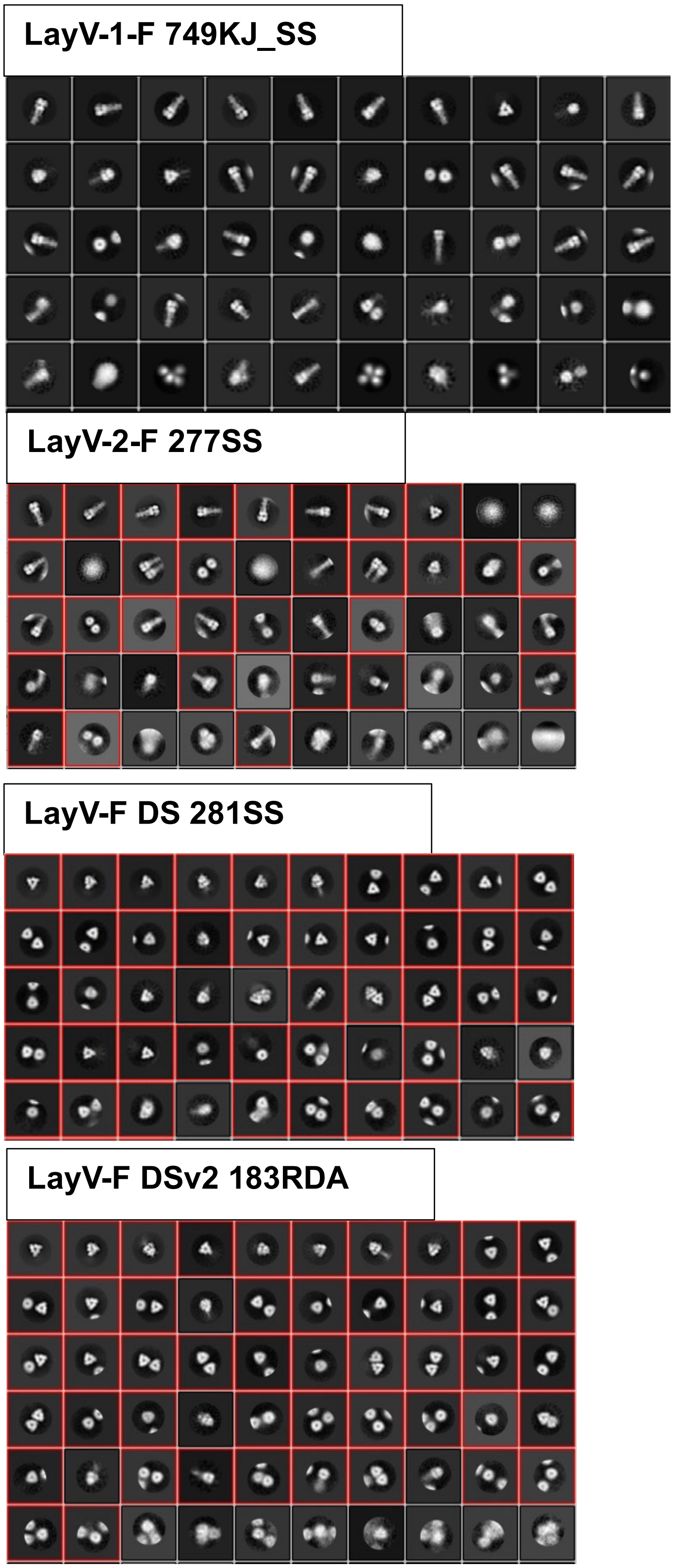

Supplemental Figure 4 (continued): NSEM analysis of HNV F ectodomains. Representative 2D classes are shown.

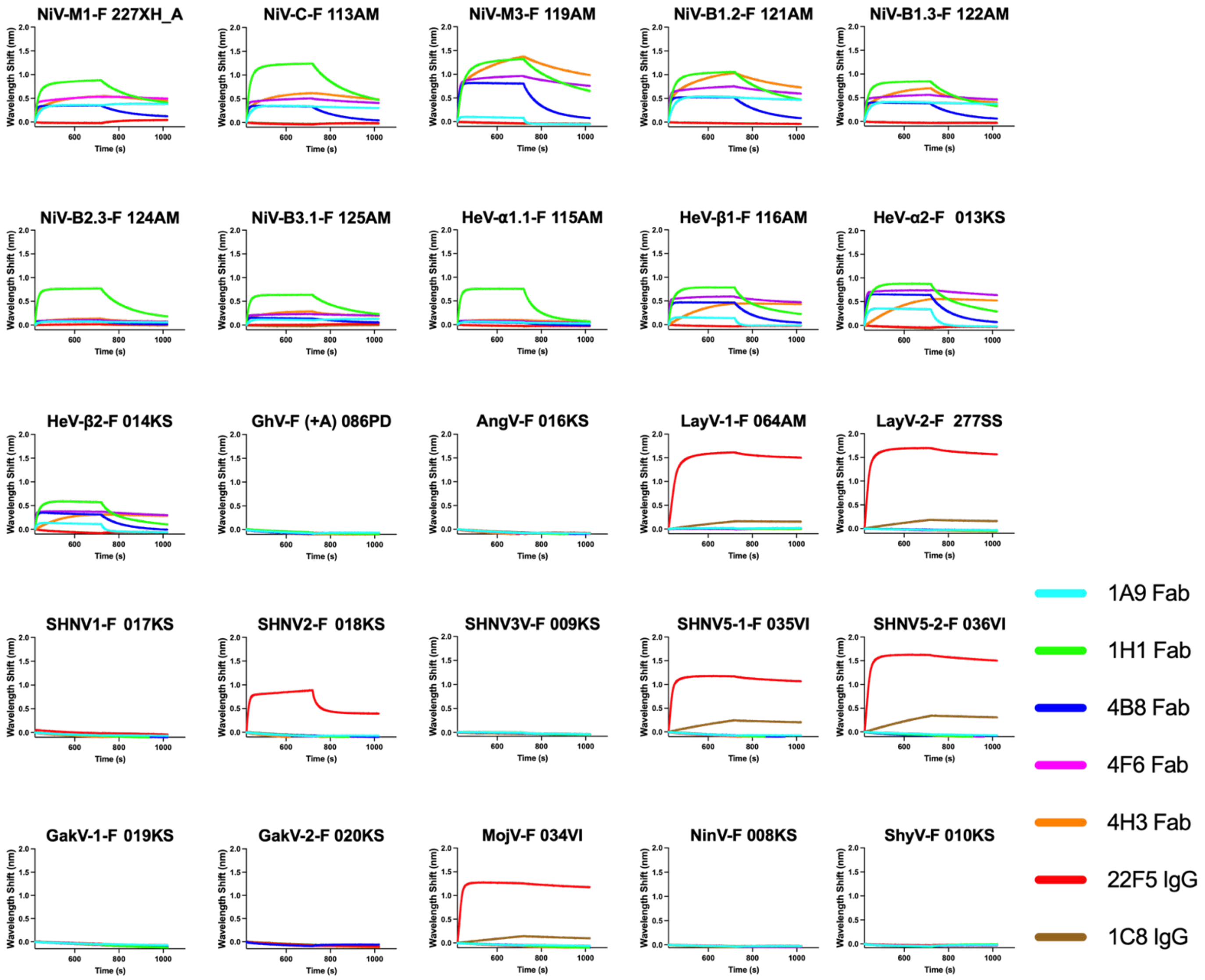

Supplemental Figure 5: Measurement of antibody binding to HNV F proteins using Biolayer Interferometry (BLI). The F proteins were captured on a BLI sensor tips via their C terminal TwinStrep tags. Binding to Fabs (1A9, 1H1, 4B8, 4F6, 4H3) or the vaccine elicited antibodies (22F5, 1C8) were measured. For each graph the virus from which the F ectodomain is derived is indicated at the top along with the lot number of the F ectodomain preparation.

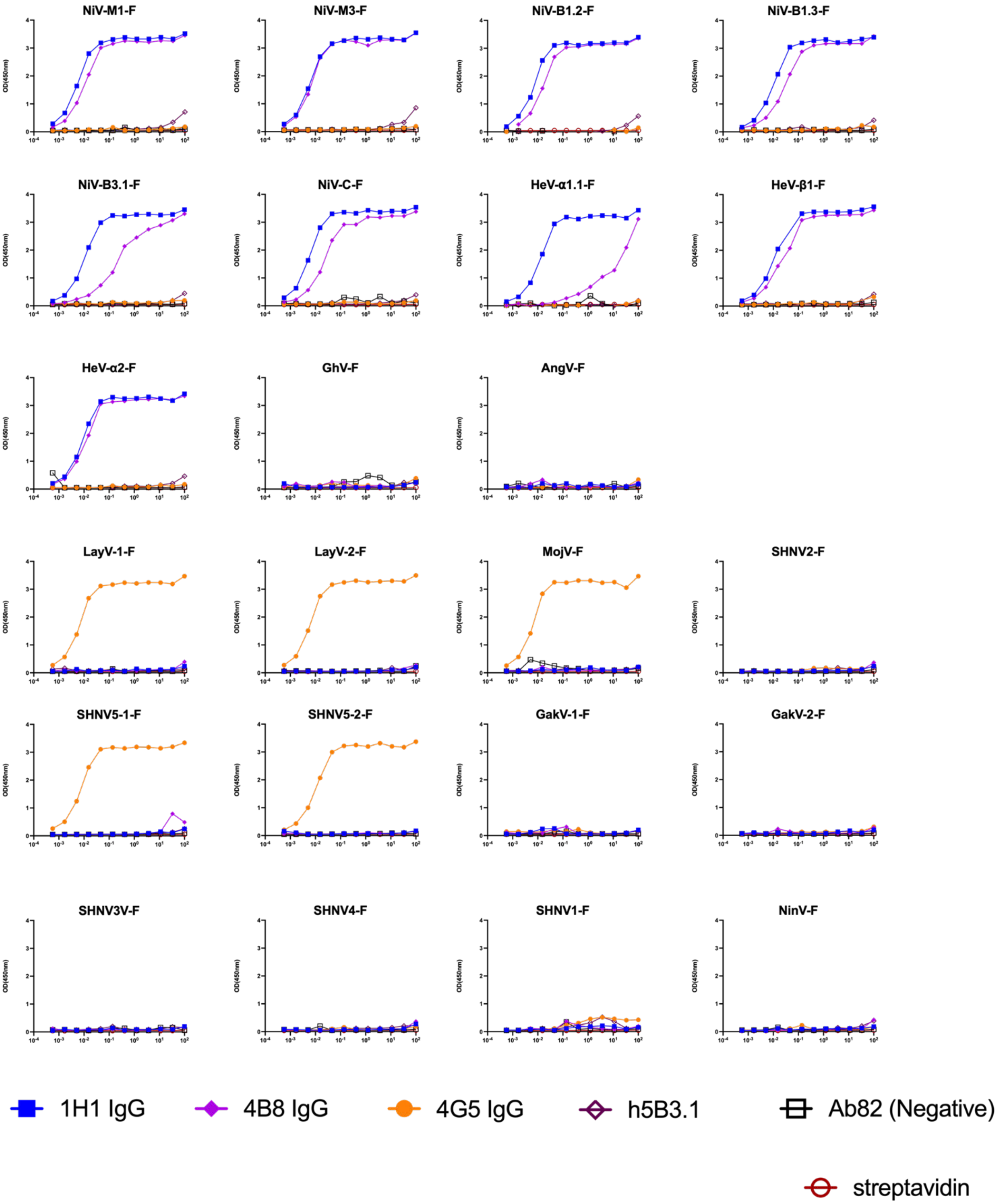

Supplemental Figure 6: Measurement of antibody binding to HNV F proteins using ELISA.

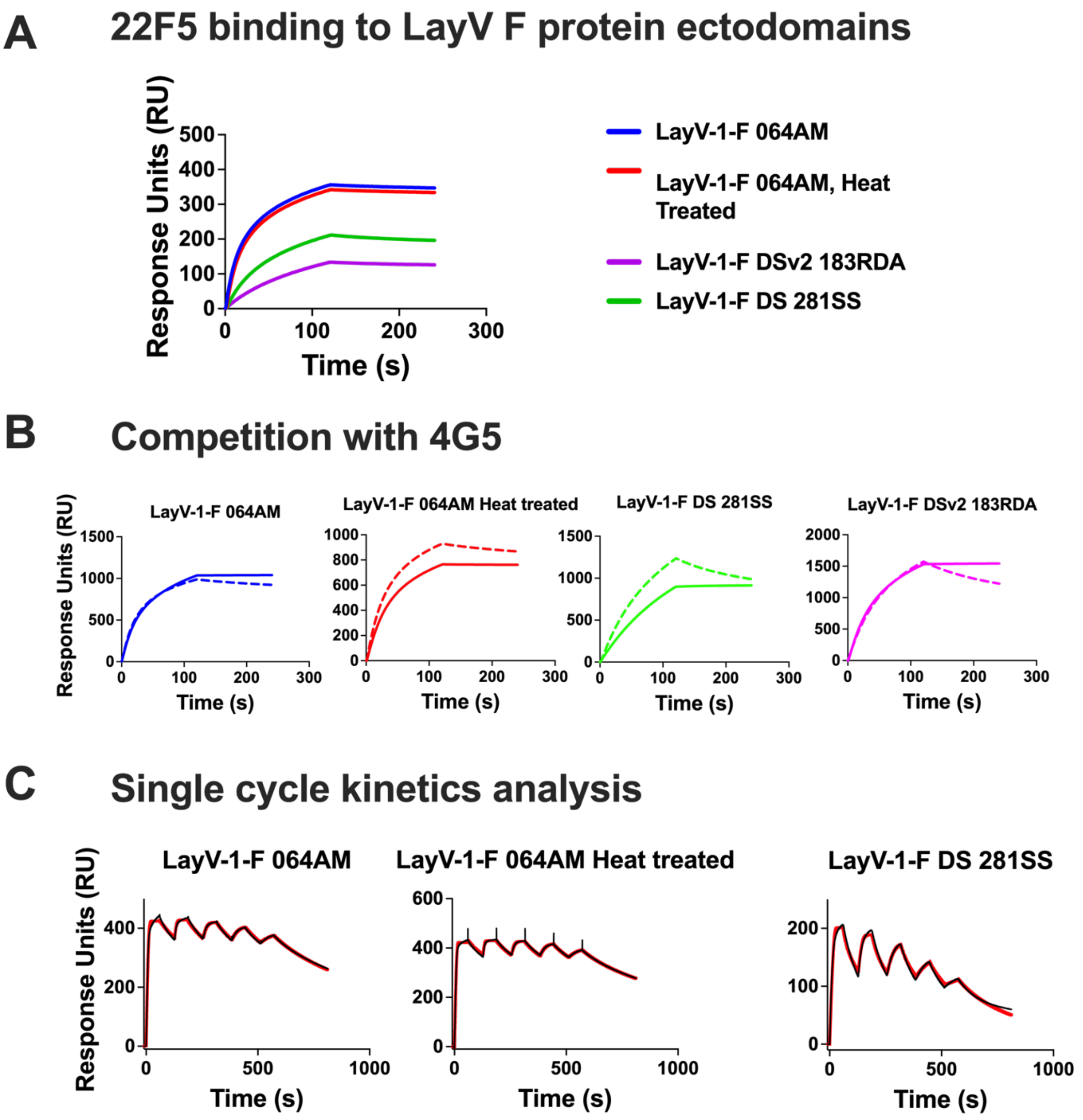

	LayV-1-F 064AM	LayV-1-F 064AM, Heat Treated	LayV-1-F DS 281SS
ka (1/Ms)	7.90E+06	1.10E+07	7.74E+06
kd (1/s)	0.002806	0.002879	0.01923
KD (M)	3.55E-10	2.62E-10	2.49E-09

Supplemental Figure 7: SPR analysis of 22F5 binding to LayV-1-F ectodomain. A. Binding of LayV F ectodomains to 22F5 IgG captured on a SPR sensor chip. The F ectodomains tested were LayV-1-F (064AM), heat treated LayV-1-F, and pre-fusion stabilized versions DS (281SS) and DSv2 (183RDA). **B.** Competition assay with 4G5 IgG captured on sensor chip and LayV F ectodomains injected as analyte either with (dashed lines) or without (solid lines) 4G5 Fab. **C.** Measurement of affinity and kinetics of binding of 22F5 Fab to LayV-1-F ectodomains. The black lines in the graphs are the blank subtracted sensorgrams and the red lines are the fit of the data to a 1:1 Langmuir binding model. The table below show the affinity and kinetic parameters obtained from the measurements.

F Protein W and Y Variability

NiV Residue	30	44	46	54	79	96	97	100	104	107	108	135	170	178	206	207	213	239	248	254	268	274	275	281
NiV-M1-F	Y	R	Y	T	Y	I	Y	N	L	D	V	Y	Y	Y	Y	L	V	Y	Y	D	Y	Y	Y	Y
NiV-M2-F	Y	R	Y	T	Y	I	Y	N	L	D	V	Y	Y	Y	Y	L	V	Y	Y	D	Y	Y	Y	Y
NiV-M3-F	Y	R	Y	T	Y	I	Y	N	L	D	V	Y	Y	Y	Y	L	V	Y	Y	D	Y	Y	Y	Y
NiV-M4-F	Y	R	Y	T	Y	I	Y	N	L	D	V	Y	Y	Y	Y	L	V	Y	Y	D	Y	Y	Y	Y
NiV-C-F	Y	R	Y	T	Y	I	Y	N	L	D	V	Y	Y	Y	Y	L	V	Y	Y	D	Y	Y	Y	Y
NiV-B1.1-F	Y	R	Y	T	Y	I	Y	N	L	D	V	Y	Y	Y	Y	S	V	Y	Y	D	Y	Y	Y	Y
NiV-B1.2-F	Y	R	Y	T	Y	I	Y	N	L	D	V	Y	Y	Y	Y	L	V	Y	Y	D	Y	Y	Y	Y
NiV-B1.3-F	Y	R	Y	T	Y	I	Y	N	L	D	V	Y	Y	Y	Y	L	V	Y	Y	D	Y	Y	Y	Y
NiV-B1.4-F	Y	R	Y	T	Y	I	Y	N	L	D	V	Y	Y	Y	Y	L	V	Y	Y	D	Y	Y	Y	Y
NiV-B2.3-F	Y	R	Y	T	Y	I	Y	N	L	D	V	Y	Y	Y	Y	L	V	Y	Y	D	Y	Y	Y	Y
NiV-B3.1-F	Y	R	Y	T	Y	I	Y	N	L	D	V	Y	Y	Y	Y	L	V	Y	Y	D	Y	Y	Y	Y
HeV-α1.1-F	Y	R	Y	T	Y	L	Y	N	L	D	V	Y	Y	Y	Y	L	V	Y	Y	D	Y	Y	Y	Y
HeV-α2-F	Y	R	Y	T	Y	L	Y	N	L	D	V	Y	Y	Y	Y	L	V	Y	Y	D	Y	Y	Y	Y
HeV-β1-F	Y	R	Y	T	Y	L	Y	N	L	D	V	Y	Y	Y	Y	L	V	Y	Y	D	Y	Y	Y	Y
HeV-β2-F	Y	R	Y	T	Y	L	Y	N	L	D	V	Y	Y	Y	Y	L	V	Y	Y	D	Y	Y	Y	Y
CedV-1-F	F	L	Y	T	Y	L	Y	N	K	G	L	Y	L	Y	Y	L	V	Y	Y	L	Y	L	Y	Y
GhV(+A)-F	Y	R	Y	S	Y	L	Y	S	A	N	A	H	N	Y	Y	Y	V	I	Y	L	Y	Y	F	Y
AngV-F	F	K	Y	T	Y	L	M	N	G	N	E	H	S	Q	Y	Y	V	L	I	N	W	H	Y	N
SHNV1-F	Y	Y	Y	T	H	T	M	R	Y	N	Y	H	L	Q	Y	Y	Y	F	Y	Y	S	G	F	R
NinV-F	F	L	Y	Y	H	S	M	K	Y	N	S	H	T	Q	Y	Y	V	F	Y	K	D	G	Y	E
SHNV2-F	Y	Y	Y	T	Y	Q	M	N	Y	Y	V	H	T	E	Y	Y	F	F	Y	Q	G	G	Y	R
GakV-1-F	Y	Y	Y	T	Y	T	M	Y	Q	G	V	H	G	Q	Y	Y	A	F	Y	H	G	G	Y	N
GakV-2-F	Y	Y	Y	T	Y	T	M	Y	Q	G	V	H	G	Q	Y	Y	A	F	Y	H	G	G	Y	N
SHNV3-F	Y	Y	Y	T	Y	T	M	Y	Q	G	V	H	G	Q	Y	Y	A	F	Y	H	G	G	Y	H
ShyV-F	Y	Y	Y	T	Y	T	M	Y	Q	G	V	H	G	Q	Y	Y	A	F	Y	H	G	G	Y	H
SHNV4-F	Y	Y	Y	T	Y	T	M	Y	Q	G	V	H	G	Q	Y	Y	A	F	Y	H	G	G	Y	H
MelV-F	F	Y	Y	T	Y	Y	Y	N	Q	R	V	H	A	E	Y	Y	A	F	Y	Y	S	G	Y	E
DewV-F	F	Y	Y	T	Y	N	M	N	Q	R	V	H	A	E	Y	Y	A	F	Y	Y	S	G	Y	E
DarV-K-F	F	Y	Y	T	Y	T	M	N	E	R	Y	H	A	E	Y	Y	V	F	Y	Y	G	G	Y	E
DarV-C-F	F	Y	Y	T	Y	T	M	N	E	R	Y	H	A	E	Y	Y	V	F	Y	Y	G	G	Y	E
LayV-1-F	Y	Y	Y	T	Y	A	M	N	G	K	Y	H	A	E	Y	Y	A	F	Y	Y	D	G	Y	E
LayV-2-F	Y	Y	Y	T	Y	A	M	N	G	K	Y	H	A	E	Y	Y	A	F	Y	Y	D	G	Y	E
MojV-F	Y	Y	Y	T	Y	T	M	N	G	K	Y	H	A	E	Y	Y	A	F	Y	Y	D	G	Y	E
SHNV5-1-F	Y	Y	Y	T	Y	A	M	K	G	K	Y	H	A	E	Y	Y	A	F	Y	Y	D	G	Y	E
SHNV5-2-F	Y	Y	Y	T	Y	A	M	K	G	K	Y	H	A	E	Y	Y	A	F	Y	Y	D	G	Y	E

NiV Residue	282	292	301	303	308	315	321	322	330	332	338	341	344	350	356	362	372	376	384	420	432	438	450	473	480
NiV-M1-F	F	Y	F	N	W	F	T	L	F	F	V	N	Y	N	N	K	H	F	F	A	Y	Y	F	Y	L
NiV-M2-F	F	Y	F	N	W	F	T	L	F	F	V	N	Y	N	N	K	H	F	F	A	Y	Y	F	Y	L
NiV-M3-F	F	Y	F	N	W	F	T	L	F	F	V	N	Y	N	N	K	H	F	F	A	Y	Y	F	Y	L
NiV-M4-F	F	Y	F	N	W	F	T	L	F	F	V	N	Y	N	N	K	H	F	F	A	Y	Y	F	Y	L
NiV-C-F	F	Y	F	N	W	F	T	L	F	F	V	N	Y	N	N	K	H	F	F	A	Y	Y	F	Y	L
NiV-B1.1-F	F	Y	F	N	W	F	T	L	F	F	V	N	Y	N	N	K	H	F	F	A	Y	Y	F	Y	L
NiV-B1.2-F	F	Y	F	N	W	F	T	L	F	F	V	N	Y	N	N	K	H	F	F	A	Y	Y	F	Y	L
NiV-B1.3-F	F	Y	F	N	W	F	T	L	F	F	V	N	Y	N	N	K	H	F	F	A	Y	Y	F	Y	L
NiV-B1.4-F	F	Y	F	N	W	F	T	L	F	F	V	N	Y	N	N	K	H	F	F	A	Y	Y	F	Y	L
NiV-B2.3-F	F	Y	F	N	W	F	T	L	F	F	V	N	Y	N	N	K	H	F	F	A	Y	Y	F	Y	L
NiV-B3.1-F	F	Y	F	N	W	F	T	L	F	F	V	N	Y	N	N	K	H	F	F	A	Y	Y	F	Y	L
HeV-α1.1-F	F	Y	F	N	W	F	T	L	Y	Y	V	N	Y	A	A	K	H	F	F	V	Y	Y	Y	Y	I
HeV-α2-F	F	Y	F	N	W	F	T	L	Y	Y	V	N	Y	A	A	K	H	F	F	V	Y	Y	Y	Y	I
HeV-β1-F	F	Y	F	N	W	F	T	L	Y	Y	V	N	Y	S	S	K	H	F	F	V	Y	Y	Y	Y	I
HeV-β2-F	F	Y	F	N	W	F	T	L	Y	Y	V	N	Y	S	S	K	H	F	F	V	Y	Y	Y	Y	I
CedV-1-F	L	Q	M	S	Y	F	N	Y	T	T	V	N	Y	Q	Q	Y	H	F	F	V	Y	I	I	Y	I
GhV(+A)-F	Y	Y	F	V	W	Y	S	Y	E	E	V	R	F	Y	Y	K	Y	F	Y	V	Y	Y	F	Y	I
AngV-F	I	V	Y	F	W	K	Y	H	N	N	Y	D	Y	P	P	S	Y	Y	Y	V	Y	P	Y	L	I
SHNV1-F	F	I	F	D	W	Y	L	Y	L	L	V	D	Y	H	H	Y	Y	F	F	Y	Y	F	V	T	I
NinV-F	F	H	F	Y	W	F	T	Y	H	H	V	D	L	T	T	K	Y	F	Y	Y	Y	F	V	L	Y
SHNV2-F	I	Y	F	I	W	Y	T	Y	R	R	V	G	Y	N	N	H	Y	F	Y	Y	Y	F	V	F	I
GakV-1-F	F	V	F	D	W	Y	T	Y	K	K	V	Y	Y	F	F	Y	H	F	Y	Y	Y	F	V	Y	I
GakV-2-F	F	V	F	D	W	Y	T	Y	K	K	V	Y	Y	F	F	Y	H	F	Y	Y	Y	F	V	Y	I
SHNV3-F	F	V	F	D	W	Y	T	Y	K	K	V	Y	Y	F	F	F	H	F	Y	Y	Y	F	V	Y	I
ShyV-F	F	V	F	D	W	Y	T	Y	K	K	V	Y	Y	F	F	F	H	F	Y	Y	Y	F	V	Y	I
SHNV4-F	F	V	F	D	W	Y	T	Y	K	K	V	Y	Y	F	F	Y	Y	F	Y	Y	Y	F	V	Y	I
MelV-F	F	Y	F	V	W	H	T	L	T	T	I	N	Y	N	N	L	Y	F	Y	Y	Y	Y	V	F	I
DewV-F	F	Y	F	V	W	Y	T	F	L	L	I	N	Y	S	S	V	Y	F	Y	Y	Y	Y	V	Y	I
DarV-K-F	F	Y	F	V	W	F	T	L	M	M	V	N	Y	T	T	S	Y	F	Y	Y	Y	Y	V	F	I
DarV-C-F	F	Y	F	V	W	F	T	L	M	M	V	N	Y	T	T	S	Y	F	Y	Y	Y	Y	V	F	I
LayV-1-F	F	V	Y	V	W	F	T	L	R	R	V	D	Y	Y	Y	K	Y	F	Y	Y	Y	Y	V	Y	F
LayV-2-F	F	V	Y	V	W	F	T	L	R	R	V	D	Y	Y	Y	K	Y	F	Y	Y	Y	Y	V	Y	F
MojV-F	F	V	Y	I	W	F	T	L	R	R	V	D	Y	H	H	K	Y	F	Y	Y	Y	Y	V	Y	F
SHNV5-1-F	F	I	F	S	W	F	T	L	K	K	V	D	Y	N	N	K	Y	F	Y	Y	Y	Y	V	Y	F
SHNV5-2-F	F	I	F	S	W	F	T	L	K	K	V	D	Y	N	N	K	Y	F	Y	Y	Y	Y	V	Y	F

G Head Domain W and Y Variability

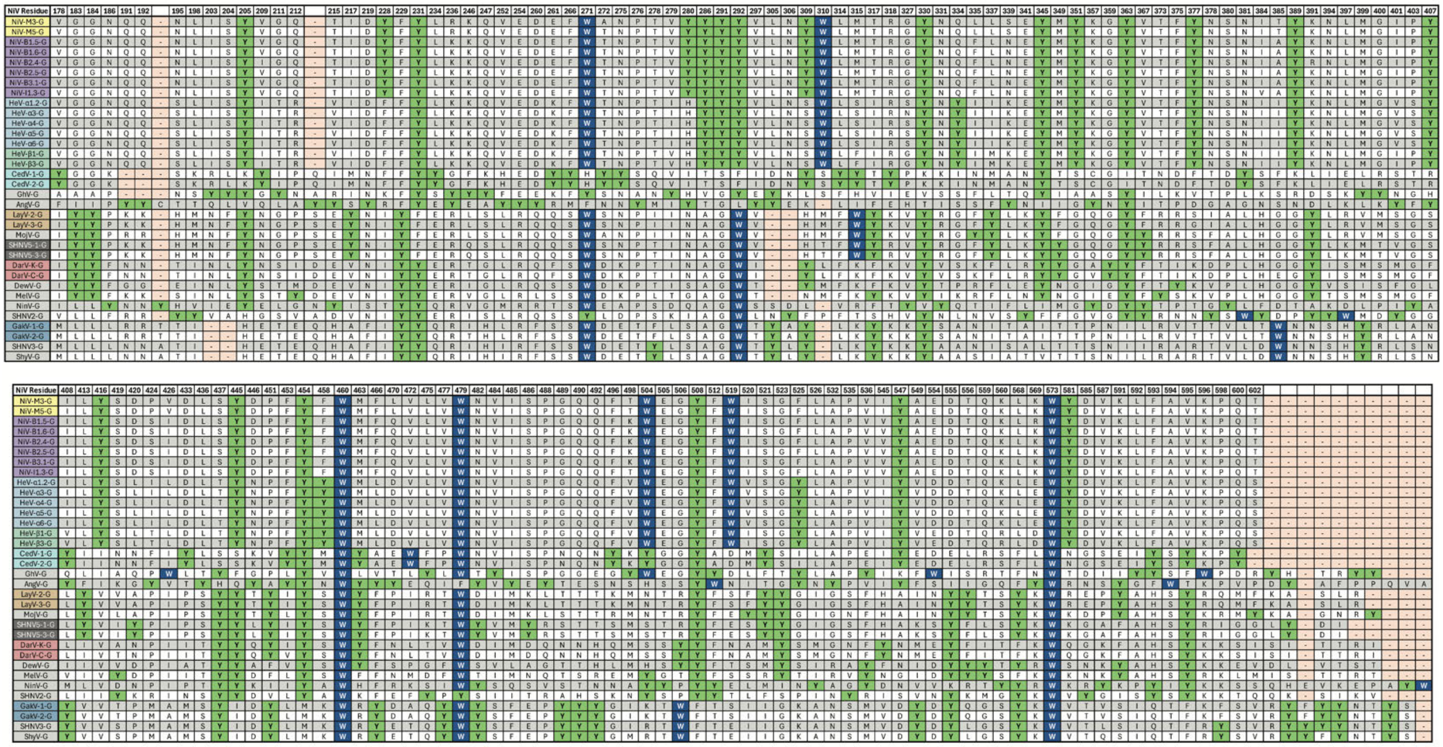

Supplemental Figure 8: DSF-relevant Residue Variability. Sequence alignment and highlighting of DSF-relevant tryptophan, blue, and tyrosine, green, residues. Gaps in the alignment are colored salmon. Species color scheme continued from Figure 1.

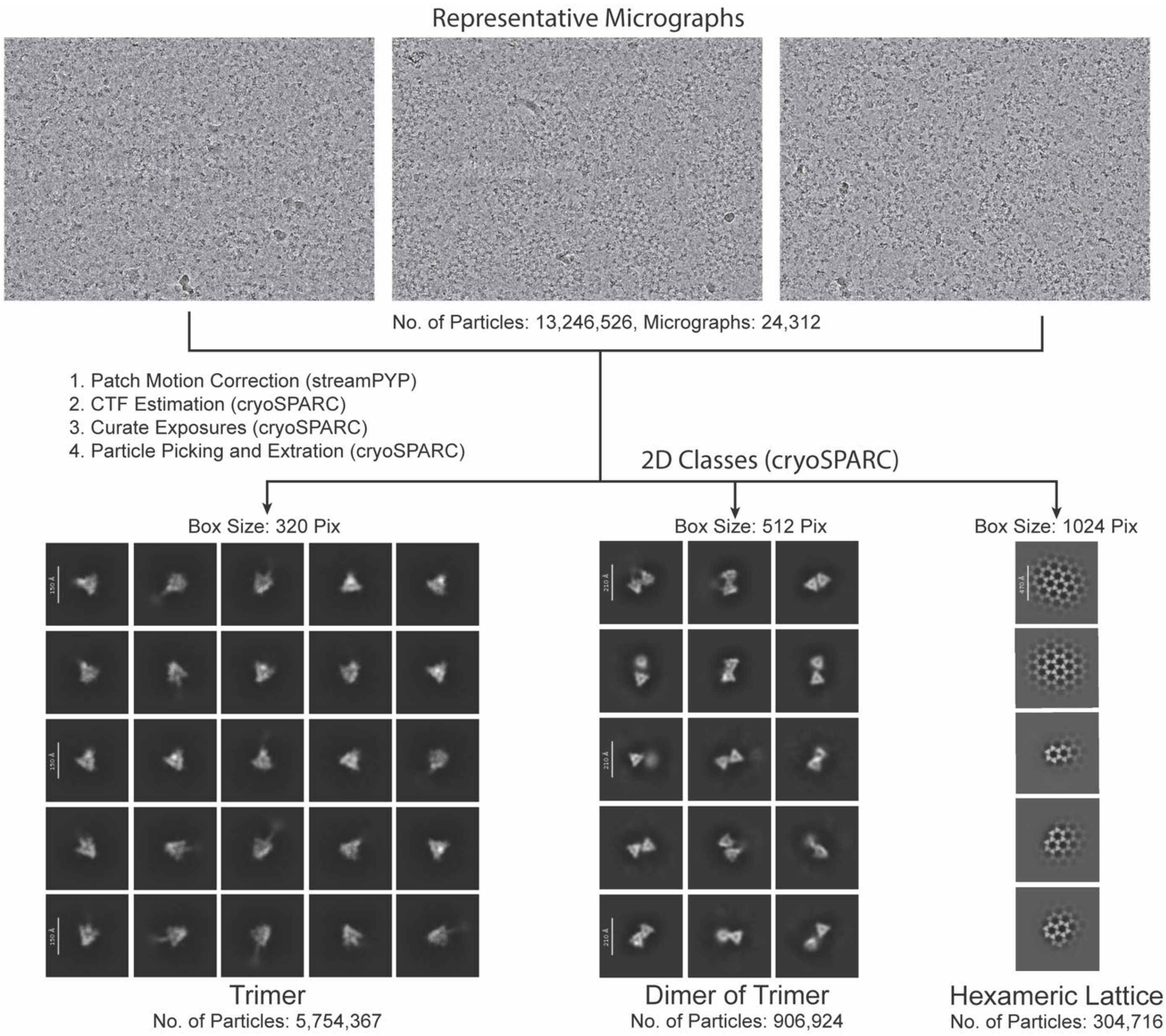

Supplemental Figure 9: Workflow of particle extraction and 2D classification. Representative cryo-EM micrographs of the AngV-F protein after motion correction by nextPYP (1). Particles were picked and extracted after curating micrographs through CTF estimation. Different box sizes are utilized to accommodate the varying dimensions of specific molecular assemblies. Trimer particles are extracted using a box size of 320 pixels, while dimer of trimer particles require a larger box size of 512 pixels. For even larger molecular assemblies, such as hexameric lattices, a box size of 1024 pixels is employed. These extracted particles are then subjected to 2D classification to group and refine them based on their structural features, ensuring accurate downstream analysis (Supplemental Figures 8-9).

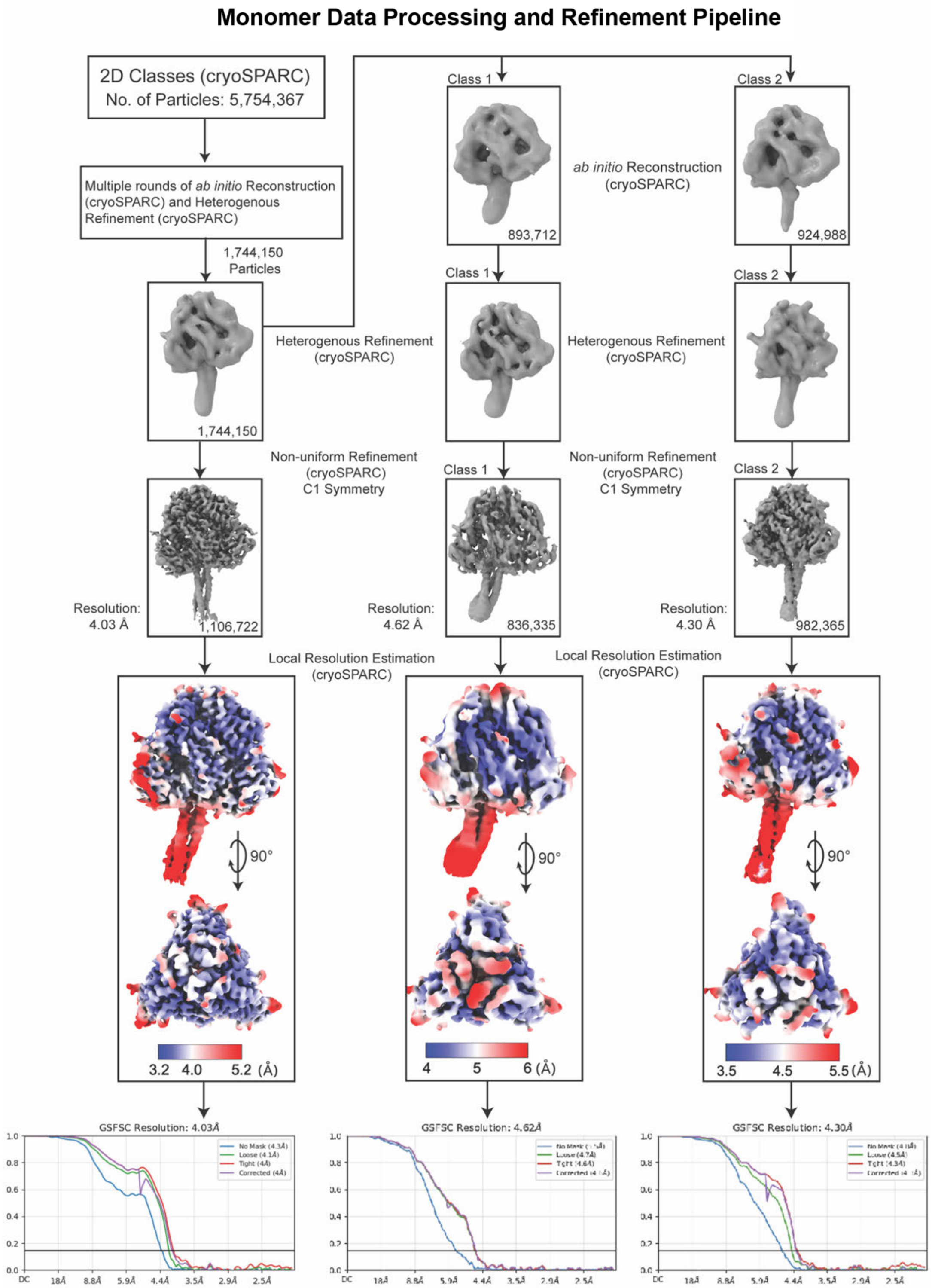

Supplemental Figure 10: Cryo-EM Data Processing and Refinement Outline of Monomer pre-fusion AngV-F protein. Representative 2D classes (Supplemental Figure 7) were selected for the *ab-intio* 3D reconstruction, with number of particles and software programs used in each step documented. Poor-quality particles were discarded after through heterogenous refinement and resulting volumes were processed using non-uniform refinement to build a high-resolution 3D reconstruction. Local resolutions were estimated, and side and top views of the AngV-F protein were plotted. Resolution values are shown as a blue to red spectrum for high-resolution regions (~3-4 Å) blue, and low-resolution regions (~5–6 Å) red. Structures were refined using C1 symmetry, with gold-standard Fourier shell correlation (GSFSC) resolutions displayed at the bottom of each step in the workflow.

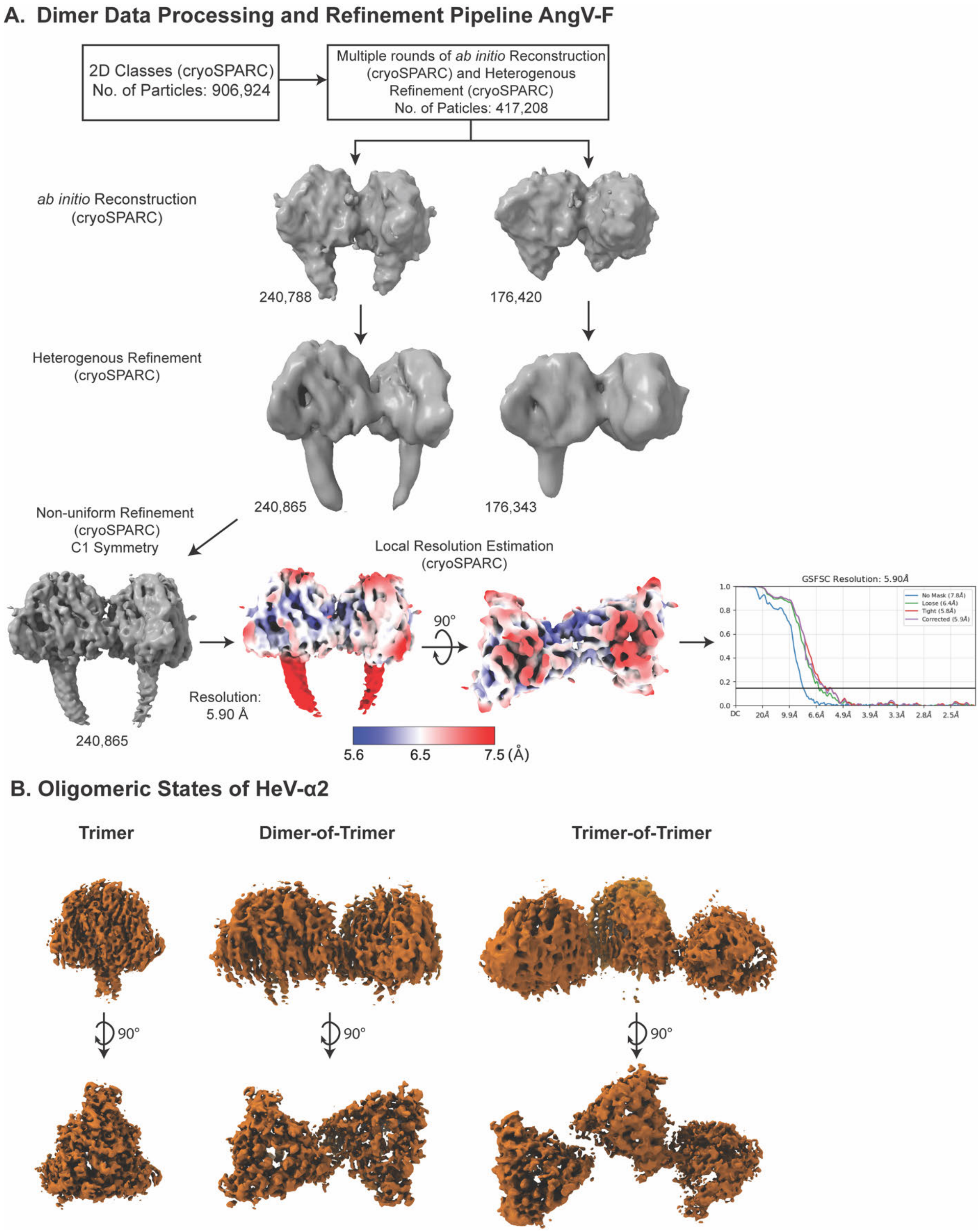

Supplemental Figure 11: Oligomeric states of AngV-F and HeV-α2-F. Panel A. Cryo-EM Data Processing and Refinement Outline of Dimer pre-fusion AngV-F protein. Representative 2D classes (Supplemental Figure 7) were selected for the *ab-intio* 3D reconstruction, with number of particles and software programs used in each step documented. Poor-quality particles were discarded after through heterogenous refinement and resulting volume was processed with non-uniform refinement to build a high-resolution 3D reconstruction. Local resolutions were estimated, and side and top views of the AngV-F Dimer protein were plotted. Resolution values are shown as a a blue to red spectrum for high-resolution regions (~5-6 Å) blue, and low-resolution regions (~7.5 Å) red. Structures were refines using C1 symmetry, and gold-standard Fourier shell correlation (GSFSC) resolutions displayed at the bottom of the workflow. **Panel B.** Cryo-EM HeV-α2-F ectodomain. The non-uniform refinement reconstruction (cryosparc) of trimer (left), dimer-of-trimers (middle) and trimer-of-trimers (right) are shown here.

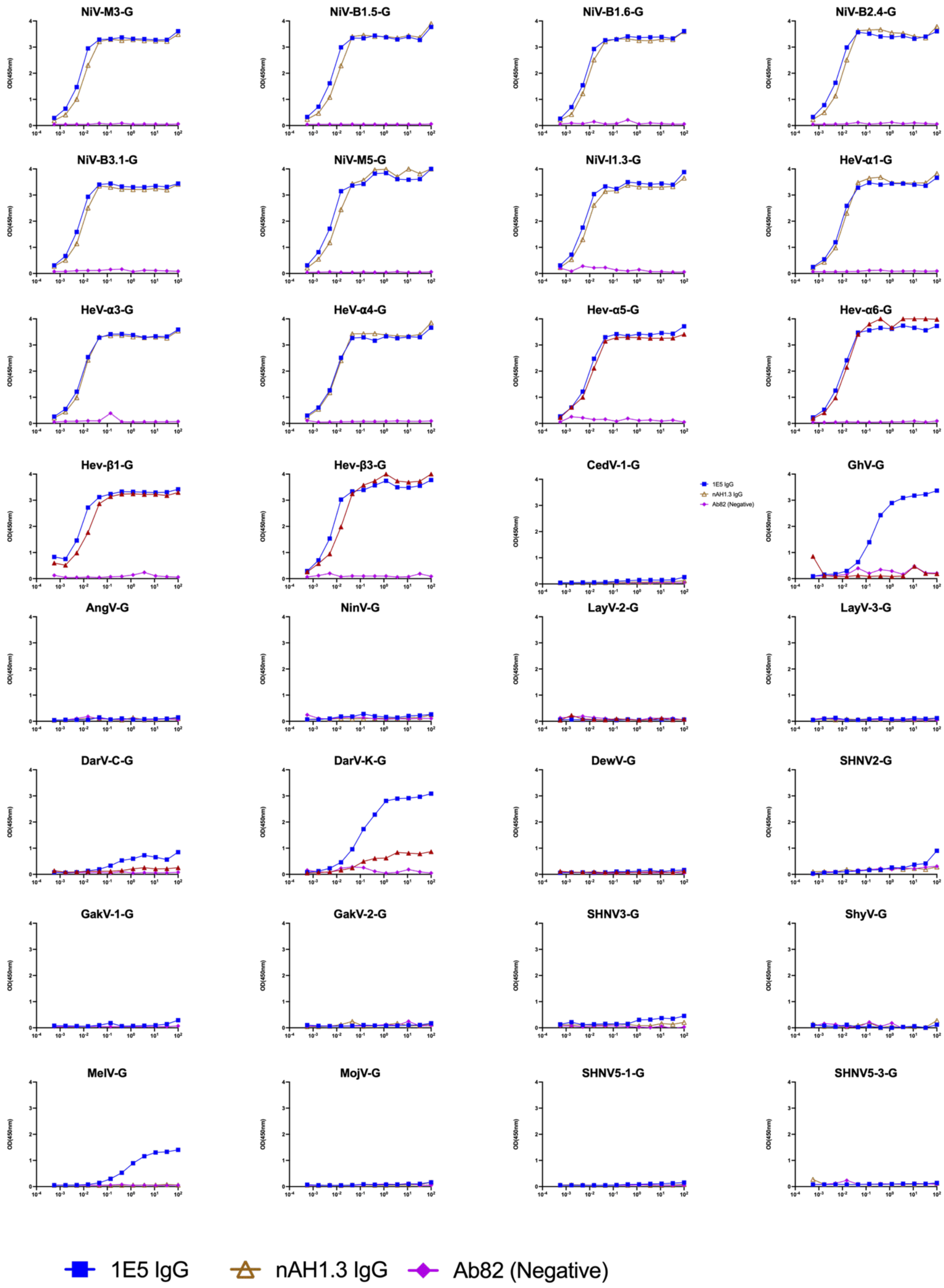

Supplemental Figure 12: Measurement of antibody binding to HNV G head domains using ELISA.

1. Liu H-F, Zhou Y, Huang Q, Piland J, Jin W, Mandel J, Du X, Martin J, Bartesaghi A. 2023. nextPYP: a comprehensive and scalable platform for characterizing protein variability in situ using single-particle cryo-electron tomography. Nature Methods 20:1909-1919.
