# Supplementary material for: Structural and antigenic characterization of novel and diverse Henipavirus glycoproteins
Source: bioRxiv. 2024 Dec 11:2024.12.11.627382. Preprint. [Version 1] doi: 10.1101/2024.12.11.627382 (PMC11661166; doi:10.1101/2024.12.11.627382)
Supplement: Supplementary file 2 [file media-2.pdf]

```
Reference sequence (1): NiV-M1
Identities normalised by aligned length.
Colored by: identity

                cov    pid     1 [         .         .         .         .         :         .         .         . 80
 1 NiV-M1    100.0% 100.0%     ------------MVVI-----LDKRCYCNL-LILILMISECSVGILHYEKLSKIGLVKGVTRKYKIKSNPLTKDIVIKMI
 2 NiV-M2    100.0%  99.8%     ------------MVVI-----LDKRCYCNL-LILILMISECSVGILHYEKLSKIGLVKGVTRKYKIKSNPLTKDIVIKMI
 3 NiV-M3    100.0%  99.8%     ------------MVVI-----LDKRCYCNL-LILILMISECSVGILHYEKLSKIGLVKGVTRKYKIKSNPLTKDIVIKMI
 4 NiV-M4    100.0%  99.5%     ------------MVVI-----LDKRCYSNL-LILILMISECSVGILHYEKLSKIGLVKGVTRKYKIKSNPLTKDIVIKMI
 5 NiV-C     100.0%  98.9%     ------------MVVV-----LDKRCYSNL-LMLILMISECSVGILHYEKLSKIGLVKGITRKYKIKSNPLTKDIVIKMI
 6 NiV-B1.1  100.0%  98.5%     ------------MAVI-----LNKRYYSNL-LILILMISECSVGILHYEKLSKIGLVKGITRKYKIKSNPLTKDIVIKMI
 7 NiV-B1.2  100.0%  99.1%     ------------MAVI-----LNKRYYSNL-LILILMISECSVGILHYEKLSKIGLVKGITRKYKIKSNPLTKDIVIKMI
 8 NiV-B1.3  100.0%  98.9%     ------------MAVI-----LNKRYYSNL-LILILMISECSVGILHYEKLSKIGLVKGITRKYKIKSNPLTKDIVIKMI
 9 NiV-B1.4  100.0%  98.7%     ------------MAVI-----LNKRYYSNL-LILILMISECSVGILHYEKLSKIGLVKGITRKYKIKSNPLTKDIVIKMI
10 NiV-B2.3  100.0%  98.5%     ------------MAVI-----LNKRYYSNL-LILILMISECSVGILHYEKLSKVGLVKGITRKYKIKSNPLTKDIVIKMI
11 NiV-B3.1  100.0%  98.2%     ------------MAVI-----LNMRYYSNL-LILILMISECSVGILHYEKLSKIGLVKGITRKYKIKSNPLTKDIVIKMI
12 HeV-α1.1   99.8%  88.1%    ------------MAT------QEVRLKCLLCGIIVLVLSLEGLGILHYEKLSKIGLVKGITRKYKIKSNPLTKDIVIKMI
13 HeV-α2     99.8%  88.3%    ------------MAT------QEVRLKCLLCGIIVLVLSLEGLGILHYEKLSKIGLVKGITRKYKIKSNPLTKDIVIKMI
14 HeV-β1    100.0%  87.9%    ------------MATQ-----RVKVSYLIC-GIVISALSLEGLGILHYEKLSKIGLVKGVTRKYKIKSNPLTKDIVIKMI
15 HeV-β2    100.0%  87.7%    ------------MATQ-----GVKVSYLIC-GIVISALSLEGLGILHYEKLSKIGLVKGVTRKYKIKSNPLTKDIVIKMI
16 CedV-1     99.6%  42.9%     ------------MS-------NKRTTVLIIISYTLFYLNNAAIVGFDFDKLNKIGVVQGRVLNYKIKGDPMTKDLVLKFI
17 GhV(+A)    99.6%  51.5%     ------------MV-------INGNIITIILLLILILKTQMSEGAIHYETLSKIGLIKGITREYKVKGTPSSKDIVIKLI
18 AngV       98.0%  39.3%     ------------MGFR-----KNLRLLGIF-WLIILS------DTVDFERLASIGGIKGHSKLYKIKGHPTTKDIVIKLV
19 SHNV1      98.4%  37.7%     ---------------------MNYTIVGIM-IITFVHESQC----INYEQLASIGVIKGHTYNYKIRGPPNTKLMVVKLI
20 NinV       99.8%  38.0%     --MSENSHWYNIIYII-----MKSRCSRLIGLIIVLTALMSAVVGIDFEGLSSIGVVKGRTLNYKIRGNSAYKILVIKLT
21 SHNV2      99.1%  40.6%     ------------MSLKFRQSTNNRTNIRLILIIVLIKHMLC----IHYENLSSVGIIKGNTYNYKIKGDPSTKMLVVKLI
22 GakV-1     99.6%  39.9%     -------------MEL-----IKLFNILYL-VGYSGLFFTDVNAGLDYEGLSSIGVIKGPSYNYKIRGTPSTKLLVIKLI
23 GakV-2     99.6%  39.9%     -------------MEL-----IKLFYILYL-VGYSGLFFTDVNAGLDYEGLSSIGVIKGPSYNYKIRGTPSTKLLVIKLI
24 SHNV3      99.6%  39.6%     -------------MEL-----IKNYCLIYL-IFYSSTLIRPLQAGLDYEALASIGVIKGPSYNYKIRGTPSTKLLVIKLI
25 ShyV       99.8%  38.7%     MKLNNTRRDNKINKQHTTMACIKNCCLVYL-ILYSSTLIIPLEAGLDYESLASIGVIKGPSYNYKIRGTPSTKLLVIKLI
26 SHNV4      99.6%  40.6%     -------------MGL-----TKSTILVYL-LVYYHAHIIAINAGLDYEGLASIGVVKGPSYNYKIRGTPTTKLLVIKLI
27 MelV       99.1%  39.7%     ----------MAKFVF-----LKTMCIGLL-----IIISDRVDSSMDFHSLSKIGIIKGKTYNYKIRGEPNTKLMVIKLI
28 DewV       99.1%  40.1%     ---------------------MARLQVVIL-YLYLLVASDVVKASLDFDNMSKIGIIKGNTYNYKIRGEPTTKLMVVKLI
29 DarV-K     99.6%  40.6%     ------------MSS------SNKIRITVI-IINILISNYLIHCSMDFVELSRVGIIKGSTYNYKIRGEPSTKLMVVKLI
30 DarV-C     99.6%  40.4%     ------------MSS------SNKIRITVI-IINILISNYLINCSMDFVELSRVGIIKGSTYNYKIRGEPSTKLMVVKLV
31 LayV-1     98.9%  40.8%     -------------MAF-----LKSAIICYL-LFYPHI----VKSSLHYDSLSKVGIIKGLTYNYKIKGSPSTKLMVVKLI
32 LayV-2     98.9%  40.8%     -------------MAF-----LKSAIICYL-LFYPHI----VKSSLHYDSLSKVGIIKGLTYNYKIKGSPSTKLMVVKLI
33 MojV       99.5%  41.2%     ------------MA-------LNKNMFSSLFLGYLLVYATTVQSSIHYDSLSKVGVIKGLTYNYKIKGSPSTKLMVVKLI
34 SHNV5-1    99.8%  40.8%     ------------MINMD----SKRLKVKVVFPIYLIIYIQMSRASLDYDQLSKIGIVKGPSYNYKIKGSPSTKLMVVKLV
35 SHNV5-2    99.8%  40.8%     ------------MINME----SKRLKVKVVFSIYFIIYIQMSRASLDYDQLSKIGIVKGPSYNYKIKGSPSTKLMVVKLV

                cov    pid    81         .         1         .         .         .         .         :         . 160
 1 NiV-M1    100.0% 100.0%     PNV------SNMSQCTGSVMENYKTRLNGILTPIKGALEIYKNNTHDLVGDVRLAGVIMAGVAIGIATAAQITAGVALYE
 2 NiV-M2    100.0%  99.8%     PNV------SNMSQCTGSVMENYKTRLNGILTPIKGALEIYKNNTHDLVGDVRLAGVIMAGVAIGIATAAQITAGVALYE
 3 NiV-M3    100.0%  99.8%     PNV------SNMSQCTGSVMENYKTRLNGILTPIKGALEIYKNNTHDLVGDVRLAGVIMAGVAIGIATAAQITAGVALYE
 4 NiV-M4    100.0%  99.5%     ANV------SNMSQCTGSVMENYKTRLNGILTPIKGALEIYKNNTHDLVGDVRLAGVIMAGVAIGIATAAQITAGVALYE
 5 NiV-C     100.0%  98.9%     PNV------SNMSQCTGSVMENYKTRLNGILTPIKGALEIYKNNTHDLVGDVRLAGVIMAGVAIGIATAAQITAGVALYE
 6 NiV-B1.1  100.0%  98.5%     PNV------SNMSQCTGSVMENYKTRLNGILTPIKGALEIYKNNTHDLVGDVRLAGVIMAGVAIGIATAAQITAGVALYE
 7 NiV-B1.2  100.0%  99.1%     PNV------SNMSQCTGSVMENYKTRLNGILTPIKGALEIYKNNTHDLVGDVRLAGVIMAGVAIGIATAAQITAGVALYE
 8 NiV-B1.3  100.0%  98.9%     PNV------SNMSQCTGSVMENYKTRLNGILTPIKGALEIYKNNTHDLVGDVRLAGVIMAGVAIGIATAAQITAGVALYE
 9 NiV-B1.4  100.0%  98.7%     PNV------SNMSQCTGSVMENYKTRLNGILTPIKGALEIYKNNTHDLVGDVRLAGVIMAGVAIGIATAAQITAGVALYE
10 NiV-B2.3  100.0%  98.5%     PNV------SNMSQCTGSVMENYKTRLNGILTPIKGALEIYKNNTHDLVGDVRLAGVIMAGVAIGIATAAQITAGVALYE
11 NiV-B3.1  100.0%  98.2%     PNV------SNMSQCTGSVMENYKTRLNGILMPIKGALEIYKNNTHDLVGDVRLAGVIMAGVAIGIATAAQITAGVALYE
12 HeV-α1.1   99.8%  88.1%    PNV------SNVSKCTGTVMENYKSRLTGILSPIKGAIELYNNNTHDLVGDVKLAGVVMAGIAIGIATAAQITAGVALYE
13 HeV-α2     99.8%  88.3%    PNV------SNVSKCTGTVMENYKSRLTGILSPIKGAIELYNNNTHDLVGDVKLAGVVMAGIAIGIATAAQITAGVALYE
14 HeV-β1    100.0%  87.9%    PNV------SNVSKCTGTVMENYKNRLTGILSPIKGAIELYNNNTHDLIGDVKLAGVVMAGIAIGIATAAQITAGVALYE
15 HeV-β2    100.0%  87.7%    PNV------SNVSKCTGTVMENYKNRLTGILSPIRGAIELYNNNTHDLIGDVKLAGVVMAGIAIGIATAAQITAGVALYE
16 CedV-1     99.6%  42.9%     PNI------VNITECVREPLSRYNETVRRLLPIHNMLGLYLNNTNAKMTGLMIAGVIMGGIAIGIATAAQITAGFALYE
17 GhV(+A)    99.6%  51.5%     PNV------TGLNKCTNISMENYKEQLDKILIPINNIIELYANSTKSAPGNARFAGVIIAGVALGVAAAAQITAGIALHE
18 AngV       98.0%  39.3%     PNL------NNLTVCSEMSIDGYLNLINAVIIPISQSLELMRNNVKDGTPNENIFGAIVAGAALGIATAAQVTSVVALHK
19 SHNV1      98.4%  37.7%     PNINIDNLGGGLSNCSSKQMESHKELVEKVLSPVAQALETMRNRVTDYSGNYRFVGAVMAGAALGVATAATVTAGIALHQ
20 NinV       99.8%  38.0%     PTIRSTSGAVNFENCTKDQISAHKVLVRNVLTPVKDALTSMKSKVTDYTPNSRLFGAIVAGAALVTATAATITAGVALHQ
21 SHNV2      99.1%  40.6%     PNI------DGLGNCTDKQMKDYKTLVKSTLQPVKDSLAQMLNNVETYNGYVKFFGAVMAGAALGVATAATVTAGIALHQ
22 GakV-1     99.6%  39.9%     PNV------ESIDNCTQKQMSDYRALVKNVLTPVSESLSTMLNYIEQQSNGVRLIGAVLAGAALGVATGAAITAGIALHK
23 GakV-2     99.6%  39.9%     PNV------ESIDNCTQKQMSDYRALVKNVLTPVSESLSTMLNYIEQQSNGVRLIGAVLAGAALGVATGAAITAGIALHK
24 SHNV3      99.6%  39.6%     PNV------GSLDNCTQKQMSDYRALVKNVLTPVSESLKTMLNYIEQQSNGVRLIGAVLAGAALGVATGAAITAGIALHK
25 ShyV       99.8%  38.7%     PNV------GSLDNCTQKQMADYKALVKNVLTPVSESLKTMLNYIEQQSNGVRLIGAVLAGAALGVATGAAITAGIALHK
26 SHNV4      99.6%  40.6%     PNV------GSLDNCTQKQMADYKSLVKNVLTPVSDALSTMLNYIEQQSNGVRLIGAVLAGAALGVATGAAITAGIALHK
27 MelV       99.1%  39.7%     PNI------DVVENCSTTQVNNYKKLVRNVLTPVRIISRYYVKNVIEQNNRVRLFGAIMAGAALGVATAATVTAGIALHR
28 DewV       99.1%  40.1%     PNI------DVVENCSATQLANYKKLVTNVLTPVKLSLDNMLKNVQDQNNRVRLFGAIMAGAALGVATAATVTAGIALHR
29 DarV-K     99.6%  40.6%     PNV------GDVANCSATQVSNYKKLVKNVLSPVSNALNTMLENVQTENNRYRLFGAIMAGAALGVATAATVTAGIALHR
30 DarV-C     99.6%  40.4%     PNV------GDVANCSTTQVSNYKKLVKNVLSPVSNALNTMLENVQTENNRYRLFGAIMAGAALGVATAATVTAGIALHR
31 LayV-1     98.9%  40.8%     PNI------DGVRNCTQKQFDEYKNLVKNVLEPVKLALNAMLDNVKSGNNKYRFAGAIMAGVALGVATAATVTAGIALHR
32 LayV-2     98.9%  40.8%     PNI------DRVRNCTQKQFDEYKNLVKNVLEPVKLALNAMLDNVKSGNNKYRFAGAIMAGVALGVATAATVTAGIALHR
33 MojV       99.5%  41.2%     PNI------DSVKNCTQKQYDEYKNLVRKALEPVKMAIDTMLNNVKSGNNKYRFAGAIMAGVALGVATAATVTAGIALHR
34 SHNV5-1    99.8%  40.8%     PNI------KEVANCTHKQVENYKTLVRNVLDPVKMSLQAMLEKVKSGNNKYRFAGAIMAGVALGVATAATVTAGIALHR
35 SHNV5-2    99.8%  40.8%     PNI------KEVANCTHKQIENYKTLVRNVLDPVKMSLQAMLEKVKSGNNKYRFAGAIMAGVALGVATAATVTAGIALHR
```

```
             cov     pid 161      .         .         .         2         .         .         .         . 240
 1 NiV-M1    100.0% 100.0%   AMKNADNINKLKSSIESTNEAVVKLQETAEKTVYVLTALQDYINTNLVPTIDKISCKQTELSLDLALSKYLSDLLFVFGP
 2 NiV-M2    100.0%  99.8%   AMKNADNINKLKSSIESTNEAVVKLQETAEKTVYVLTALQDYINTNLVPTIDKISCKQTELSLDLALSKYLSDLLFVFGP
 3 NiV-M3    100.0%  99.8%   AMKNADNINKLKSSIESTNEAVVKLQETAEKTVYVLTALQDYINTNLVPTIDKISCKQTELSLDLALSKYLSDLLFVFGP
 4 NiV-M4    100.0%  99.5%   AMKNADNINKLKSSIESTNEAVVKLQETAEKTVYVLTALQDYINTNLVPTIDKISCKQTELSLDLALSKYLSDLLFVFGP
 5 NiV-C     100.0%  98.9%   AMKNADNINKLKSSIESTNEAVIKLQETAEKTVYVLTALQDYINTNLVPTIDKISCKQTELSLDLALSKYLSDLLFVFGP
 6 NiV-B1.1  100.0%  98.5%   AMKNADNINKLKSSIESTNEAVVKLQETAEKTVYVLTALQDYINTNLVPTIDKISCKQTELSLDLALSKYSSDLLFVFGP
 7 NiV-B1.2  100.0%  99.1%   AMKNADNINKLKSSIESTNEAVVKLQETAEKTVYVLTALQDYINTNLVPTIDKISCKQTELSLDLALSKYLSDLLFVFGP
 8 NiV-B1.3  100.0%  98.9%   AMKNADNINKLKSSIESTNEAVVKLQETAEKTVYVLTALQDYINTNLVPTIDKISCKQTELSLDLALSKYLSDLLFVFGP
 9 NiV-B1.4  100.0%  98.7%   AMKNADNINKLKSSIESTNEAVVKLQETAEKTVYVLTALQDYINTNLVPTIDKISCKQTELSLDLALSKYLSDLLFVFGP
10 NiV-B2.3  100.0%  98.5%   AMKNADNINKLKSSIESTNEAVVKLQETAEKTVYVLTALQDYINTNLVPTIDKISCKQTELSLDLALSKYLSDLLFVFGP
11 NiV-B3.1  100.0%  98.2%   AMKNADNINKLKSSIESTNEAVVKLQETAEKTVYVLTALQDYINTNLVPTIDKISCKQMELSLDLALSKYLSDLLFVFGP
12 HeV-α1.1   99.8%  88.1%  AMKNADNINKLKSSIESTNEAVVKLQETAEKTVYVLTALQDYINTNLVPTIDQISCKQTELALDLALSKYLSDLLFVFGP
13 HeV-α2     99.8%  88.3%  AMKNADNINKLKSSIESTNEAVVKLQETAEKTVYVLTALQDYINTNLVPTIDQISCKQTELALDLALSKYLSDLLFVFGP
14 HeV-β1    100.0%  87.9%  AMKNADNINKLKSSIESTNEAVVKLQETAEKTVYVLTALQDYINTNLVPTIDQISCKQTELALDLALSKYLSDLLFVFGP
15 HeV-β2    100.0%  87.7%  AMKNADNINKLKSSIESTNEAVVKLQETAEKTVYVLTALQDYINTNLVPTIDQISCKQTELALDLALSKYLSDLLFVFGP
16 CedV-1     99.6%  42.9%   AKKNTENIQKLTDSIMKTQDSIDKLTDSVGTSILILNKLQTYINNQLVPNLELLSCRQNKIEFDLMLTKYLVDLMTVIGP
17 GhV(+A)    99.6%  51.5%   ARQNAERINLLKDSISATNNAVAELQEATGGIVNVITGMQDYINTNLVPQIDKLQCSQIKTALDISLSQYYSEILTVFGP
18 AngV       98.0%  39.3%   SNQNAAKINSLRDSITKTNQAVEQLSLGVQETVSVLMGLQDQINTNLVPKINILSCKQLSNTLNIMLLQYYSQILTVFGP
19 SHNV1      98.4%  37.7%   SNQNAKAIDQMKEAIRTTNKAVQELTLSTRQTLLVIDSLQNQINTQIVPAMNRLSCEVLGLTVGIQLTQYYSEILTYFGP
20 NinV       99.8%  38.0%   SNQNAQAIANMKDAISKTNQAVSELKQGTQKLATVVDSLQNQINSQIIPVLNQLGCQTTGLTIGLYLTRYYSEILTVFGP
21 SHNV2      99.1%  40.6%   SNQNAKDIANMKDAIMKTNQAITTLSSASQKMLTVIDSIRGEINQQIIPVLNQLSCETVGLNLGIKLTQYYSQISTFFGP
22 GakV-1     99.6%  39.9%   SNQNAQAIAQLKDAIKNTNQAVQTLKMANQELLGVVDSLRGQINTQIIPVLNKLSCDTVGLTLGIKLTQYYSEILTAFGP
23 GakV-2     99.6%  39.9%   SNQNAQAIAQLKDAIKNTNQAVQTLKMANQELLGVVDSLRGQINTQIIPVLNKLSCDTVGLTLGIKLTQYYSEILTAFGP
24 SHNV3      99.6%  39.6%   SNQNAQAIAQLKDSIKNTNMAVQTLKLANQEILGVVDSLRGQINTQIIPVLNQLSCDTVGLTLGIKLTQYYSEILTAFGP
25 ShyV       99.8%  38.7%   SNQNAQAIAQLKDSIKNTNMAVQTLKLANQEILGVVDSLRGQINTQIIPVLNQLSCDTVGLTLGIKLTQYYSEILTAFGP
26 SHNV4      99.6%  40.6%   SNQNAQAIAQLKDAIKNTNQAVQTLKLANQEILGVVDSLRGQINTQIIPVINQLSCDTIGLTLGIKLTQYYSEILTAFGP
27 MelV       99.1%  39.7%   SNENARNIALMKEAIKNTNQAVTKLQLAGQQTLAVIDNIRGEINNQIIPVINKLTCENIGLNVGIKLTQYYSEVLTAFGP
28 DewV       99.1%  40.1%   SNENAKNIAKLKNAIQNTNLAITKLQMAGQQTLAVIDNIRGEINNQIIPVLNQLSCETVGLNVGIKLTQYYSEVLTAFGP
29 DarV-K     99.6%  40.6%   SNENARNIAAMKNAIQNTNQAITKLQMAGQQTLAVIDNIRGEINNQIIPLLNKLSCETVGLNVGIKLTQYYSEILTVFGP
30 DarV-C     99.6%  40.4%   SNENARNIAAMKNAIQNTNQAITKLQMAGQQTLAVIDNIRGEINNQIIPLLNKLSCETVGLNVGIKLTQYYSEILTVFGP
31 LayV-1     98.9%  40.8%   SNENAQAIANMKNAIQNTNEAVKQLQLANKQTLAVIDTIRGEINNNIIPVINQLSCDTIGLSVGIKLTQYYSEILTAFGP
32 LayV-2     98.9%  40.8%   SNENAQAIANMKNAIQNTNEAVKQLQLANKQTLAVIDTIRGEINNNIIPVINQLSCDTIGLSVGIKLTQYYSEILTAFGP
33 MojV       99.5%  41.2%   SNENAQAIANMKSAIQNTNEAVKQLQLANKQTLAVIDTIRGEINNNIIPVINQLSCDTIGLSVGIRLTQYYSEIITAFGP
34 SHNV5-1    99.8%  40.8%   SSENAQAIARIKGAIQNTNEAVKQLQLANKQMLAVIDTIRGEINENIIPIMNELSCETIGLNVGIKLTQYYSEIITAFGP
35 SHNV5-2    99.8%  40.8%   SSENAQAIARIKGAIQNTNEAVKQLQLGNKQMLAVIDTIRGEINENIIPIMNELSCETIGLNVGIKLTQYYSEIITAFGP

             cov     pid 241      :         .         .         .         .         3         .         . 320
 1 NiV-M1    100.0% 100.0%   NLQDPVSNSMTIQAISQAFGGNYETLLRTLGYATEDFDDLLESDSITGQIIYVDLSSYYIIVRVYFPILTEIQQAYIQEL
 2 NiV-M2    100.0%  99.8%   NLQDPVSNSMTIQAISQAFGGNYETLLRTLGYATEDFDDLLESDSITGQIIYVDLSSYYIIVRVYFPILTEIQQAYIQEL
 3 NiV-M3    100.0%  99.8%   NLQDPVSNSMTIQAISQAFGGNYETLLRTLGYAIEDFDDLLESDSITGQIIYVDLSSYYIIVRVYFPILTEIQQAYIQEL
 4 NiV-M4    100.0%  99.5%   NLQDPVSNSMTIQAISQAFGGNYETLLRTLGYATEDFDDLLESDSITGQIIYVDLSSYYIIVRVYFPILTEIQQAYIQEL
 5 NiV-C     100.0%  98.9%   NLQDPVSNSMTIQAISQAFGGNYETLLRTLGYATEDFDDLLESDSITGQIIYVDLSSYYIIVRVYFPILTEIQQAYIQEL
 6 NiV-B1.1  100.0%  98.5%   NLQDPVSNSMTIQAISQAFGGNYETLLRTLGYATEGFDDLLESDSITGQIIYVDLSGYYIIVRVYFPILTEIQQAYIQEL
 7 NiV-B1.2  100.0%  99.1%   NLQDPVSNSMTIQAISQAFGGNYETLLRTLGYATEDFDDLLESDSITGQIIYVDLSSYYIIVRVYFPILTEIQQAYIQEL
 8 NiV-B1.3  100.0%  98.9%   NLQDPVSNSMTIQAISQAFGGNYETLLRTLGYATEDFDDLLESDSITGQIIYVDLSGYYIIVRVYFPILTEIQQAYIQEL
 9 NiV-B1.4  100.0%  98.7%   NLQDPVSNSMTIQAISQAFGGNYETLLRTLGYATEDFDDLLESDSITGQIIYVDLSGYYIIVRVYFPILTEIQQAYIQEL
10 NiV-B2.3  100.0%  98.5%   NLQDPVSNSMTIQAISQAFGGNYETLLRTLGYTTEDFDDLLESDSITGQIIYVDLSGYYIIVRVYFPILTEIQQAYIQEL
11 NiV-B3.1  100.0%  98.2%   NLQDPVSNSMTIQAISQAFGGNYETLLRTLGYATEDFDDLLESDSITGQIIYVDLSGYYIIVRVYFPILTEIQQAYIQEL
12 HeV-α1.1   99.8%  88.1%  NLQDPVSNSMTIQAISQAFGGNYETLLRTLGYATEDFDDLLESDSIAGQIVYVDLSSYYIIVRVYFPILTEIQQAYVQEL
13 HeV-α2     99.8%  88.3%  NLQDPVSNSMTIQAISQAFGGNYETLLRTLGYATEDFDDLLESDSITGQIVYVDLSSYYIIVRVYFPILTEIQQAYVQEL
14 HeV-β1    100.0%  87.9%  NLQDPVSNSMTIQAISQAFGGNYETLLRTLGYATEDFDDLLESDSITGQIVYVDLSSYYIIVRVYFPILTEIQQAYVQEL
15 HeV-β2    100.0%  87.7%  NLQDPVSNSMTIQAISQAFGGNYETLLRTLGYATEDFDDLLESDSITGQIVYVDLSSYYIIVRVYFPILTEIQQAYVQEL
16 CedV-1     99.6%  42.9%   NINNPVNKDMTIQSLSLLFDGNYDIMMSELGYTPQDFLDLIESKSITGQIIYVDMENLYVVIRTYLPTLIEVPDAQIYEF
17 GhV(+A)    99.6%  51.5%   NLQNPVTTSMSIQAISQSFGGNIDLLLNLLGYTANDLLDLLESKSITGQITYINLEHYFMVIRVYYPIMTTISNAYVQEL
18 AngV       98.0%  39.3%   NLRDPATVPVSIQALSQLFEGNLELLTSSLGISSTDFNDLLTGKLITGSIIWADTQGHYLILRVNIPDLIEVPGAVIQEF
19 SHNV1      98.4%  37.7%   ALQDPIDSTLTIQAISHAFGGNFDILMKTMGYTVGDLYDVLKGDLITGKIISVNPKEGFIALEVRFPTLTQVNNAIVQEL
20 NinV       99.8%  38.0%   AIQDPVNSALTIQAISKAFEGNFDKLMSAMGYSVSDLKDVLESDLVRGKIIDVDPDVGYMALQIEFPQITPVTGAHIQEL
21 SHNV2      99.1%  40.6%   ALQNPVQSILTIQAISHAFGGNFNELMTVMGYTGSDFQDIIQGNLITGSVIGVDPEVGYIALEIRIPSLTVVPNAYVQEL
22 GakV-1     99.6%  39.9%   AIQDPVNSKLTIQAISGAFSGNFDEMMKVMGYSGSDLHDILQGDLITGNIIGVDPDVGYIALEINFPTLTEIPNAVIQEL
23 GakV-2     99.6%  39.9%   AIQDPVNSKLTIQAISGAFSGNFDEMMKVMGYSGSDLHDILQGDLITGNIIGVDPDVGYIALEINFPTLTEIPNAVIQEL
24 SHNV3      99.6%  39.6%   AIQDPVNSKLTIQAISGAFSGNFDEMMKVMGYTGSDLHDILQGDLITGNIIGVDPDVGYIALEIHFPTLTEIPNAVIQEL
25 ShyV       99.8%  38.7%   AIQDPVNSKLTIQAISGAFSGNFDEMMKVMGYTGSDLHDILQGDLITGNIIGVDPDVGYIALEIHFPTLTEIPNAVIQEL
26 SHNV4      99.6%  40.6%   AIQDPVNSKLTIQAISGAFSGNFDEMMKVMGYTGSDLHDILQGDLITGNIIGVDPDIGYIALEIHFPTLTEIPNAVIQEL
27 MelV       99.1%  39.7%   AIQDPVNARITIQAISKVFNNNFDELLNVMGYSTQDLYEVLHGGLIRGNVISADPEVGYLALEIEFPNLSVVPNAYIQEI
28 DewV       99.1%  40.1%   AIQDPVNSKITIQAISKAFNNNFDELLKVMGYTSQDLYEILHGDLITGNVISADPEAGYIALEIEFPNLSTVPNAYVQEL
29 DarV-K     99.6%  40.6%   AIQDPINSKITIQAISKAFGGNFDELLKVMGYTSQDLYEILHGGLITGNIIGVDPDTGYIALEIEFPNLSIVQNAYIQEL
30 DarV-C     99.6%  40.4%   AIQDPINSKITIQAISKAFGGNFDELLNVMGYTSQDLYEILHGGLITGNIIGVDPDTGYIALEIEFPNLSIVQNAYIQEL
31 LayV-1     98.9%  40.8%   ALQNPVNTRITIQAISSVFNRNFDELLKIMGYTSGDLYEILHSGLIRGNIIDVDVEAGYIALEIEFPNLTLVPNAVVQEL
32 LayV-2     98.9%  40.8%   ALQNPVNTRITIQAISSVFNRNFDELLKIMGYTSGDLYEILHSGLIRGNIIDVDVEAGYIALEIEFPNLTLVPNAVVQEL
33 MojV       99.5%  41.2%   ALQNPVNTRITIQAISSVFNGNFDELLKIMGYTSGDLYEILHSELIRGNIIDVDVDAGYIALEIEFPNLTLVPNAVVQEL
34 SHNV5-1    99.8%  40.8%   ALQNPIGSKITIQAISSAFNGNFDELLKSMGYTSNDLYEVLQSGLIRGNIIDVDPEVGYIALEIEFPNLTLVPNAIIQEL
35 SHNV5-2    99.8%  40.8%   ALQNPIGSKITIQAISSAFNGNFDELLKSMGYTSNDLYEVLQSGLIRGNIIDVDPEAGYIALEIEFPNLTLVPNAIIQEL

             cov     pid 321      .         .         :         .         .         .         .         4 400
 1 NiV-M1    100.0% 100.0%   LPVSFNNDNSEWISIVPNFILVRNTLISNIEIGFCLITKRSVICNQDYATPMTNNMRECLTGSTEKCPRELVVSSHVPRF
 2 NiV-M2    100.0%  99.8%   LPVSFNNDNSEWISIVPNFILVRNTLISNIEIGFCLITKRSVICNQDYATPTTNNMRECLTGSTEKCPRELVVSSHVPRF
 3 NiV-M3    100.0%  99.8%   LPVSFNNDNSEWISIVPNFILVRNTLISNIEIGFCLITKRSVICNQDYATPMTNNMRECLTGSTEKCPRELVVSSHVPRF
 4 NiV-M4    100.0%  99.5%   LPVSFNNDNSEWISIVPNFILVRNTLISNIEIGFCLITKRSVICNQDYATPMTNNMRECLTGSTEKCPRELVVSSHVPRF
 5 NiV-C     100.0%  98.9%   LPVSFNNDNSEWISIVPNFILVRNTLISNIEIGFCLITKRSVICNQDYATPMTNNMRECLTGSTEKCPRELVVSSHVPRF
```

```
 6 NiV-B1.1  100.0%  98.5%     LPVSFNNDNSEWISIVPNFILVRNTLISNIEIGFCLITKRSVICNQDYATPMTNNMRECLTGSTEKCPRELVVSSHVPRF
 7 NiV-B1.2  100.0%  99.1%     LPVSFNNDNSEWISIVPNFILVRNTLISNIEIGFCLITKRSVICNQDYATPMTNNMRECLTGSTEKCPRELVVSSHVPRF
 8 NiV-B1.3  100.0%  98.9%     LPVSFNNDNSEWISIVPNFILVRNTLISNIEIGFCLITKRSVICNQDYATPMTNNMRECLTGSTEKCPRELVVSSHVPRF
 9 NiV-B1.4  100.0%  98.7%     LPVSFNNDNSEWISIVPNFILVRNTLISNIEIGFCLITKRSVICNQDYATPMTNNMRECLTGSTEKCPRELVVSSHVPRF
10 NiV-B2.3  100.0%  98.5%     LPVSFNNDNSEWISIVPNFILVRNTLISNIEIGFCLITKRSVICNQDYATPMTNNMRECLTGSTEKCPRELVVSSHVPRF
11 NiV-B3.1  100.0%  98.2%     LPVSFNNDNSEWISIVPNFILVRNTLISNIEIGFCLITKRSVICNQDYATPMTNNMRECLTGSTEKCPRELVVSSHVPRF
12 HeV-α1.1   99.8%  88.1%     LPVSFNNDNSEWISIVPNFVLIRNTLISNIEVKYCLITKKSVICNQDYATPMTASVRECLTGSTDKCPRELVVSSHVPRF
13 HeV-α2     99.8%  88.3%     LPVSFNNDNSEWISIVPNFVLIRNTLISNIEVKYCLITKKSVICNQDYATPMTASVRECLTGSTDKCPRELVVSSHVPRF
14 HeV-β1    100.0%  87.9%     LPVSFNNDNSEWISIVPNFVLIRNTLISNIEVKYCLTTKKSVICNQDYATPMTSTVRECLTGSTDKCPRELVVSSHVPRF
15 HeV-β2    100.0%  87.7%     LPVSFNNDNSEWISIVPNFVLIRNTLISNIEVKYCLTTKKSVICNQDYATPMTSTVRECLTGSTDKCPRELVVSSHVPRF
16 CedV-1     99.6%  42.9%     NKITMSSNGGEYLSTIPNFILIRGNYMSNIDVATCYMTKASVICNQDYSLPMSQNLRSCYQGETEYCPVEAVIASHSPRF
17 GhV(+A)    99.6%  51.5%     IKISFNVDGSEWVSLVPSYILIRNSYLSNIDISECLITKNSVICRHDFAMPMSYTLKECLTGDTEKCPREAVVTSYVPRF
18 AngV       98.0%  39.3%     IKIGYNFGGSLWMPTIPNKIIIRGYHLSLIDSTNCIITDNSYVCDRDYSLPMNPILRECFEGNTSSCGREMVLNSYIPLY
19 SHNV1      98.4%  37.7%     MPISFNDKGDEWISTVPRYVLERVLYLSNIDISLCSVGETSVVCDNDYASPMSHQLRECLQTNTSYCPRERVLASYVPKF
20 NinV       99.8%  38.0%     LPISFNYKGDEWITILPSFVLNRMTYLSNIDIAHCLVTETSVICDNDLAMPMSTQMRDCLLGNTSKCSREQVITSYVPRF
21 SHNV2      99.1%  40.6%     LPVSFNIDGDEWMTIVPNYVLTRTTYLSNIDINRCLITDKSVICGNDYATPMSNQLINCLNGNTQHCAREAVVTSYVPKF
22 GakV-1     99.6%  39.9%     MPISFNDKGDEWMALVPRYVLLRTTYISNIDISKCLLTERSVICYNDYATPMSFDIIRCLTGNLTYCPREQIMASHVPKF
23 GakV-2     99.6%  39.9%     MPISFNDKGDEWMALVPRYVLLRTTYISNIDISKCLLTERSVICYNDYATPMSFDIIRCLTGNLTYCPREQIMASHVPKF
24 SHNV3      99.6%  39.6%     MPISFNDKGDEWMALVPRYVLLRTTYVSNIDISKCLLTERSVICYNDYATPMSFDVLRCLTGNLTFCPREQIIASHVPKF
25 ShyV       99.8%  38.7%     MPISFNDKGDEWMALVPRYVLLRTTYVSNIDISKCLLTERSVICYNDYATPMSFDVLRCLTGNLTFCPREQIIASHVPKF
26 SHNV4      99.6%  40.6%     MPISFNDKGDEWMTLVPRYVLLRTTYVSNIDISKCLITERSVICYNDYATPMSFDVIRCLTGNLTYCPREQIIASYVPRF
27 MelV       99.1%  39.7%     MPISFNVDGDEWVTVVPRHTLIRTTLLSNIDITTCSIIESSIICNNDYALPMSNELINCLQGSTDLCAREKVISNYVPKF
28 DewV       99.1%  40.1%     MPISFNVDGDEWVTLVPRYVLIRTTFLSNIDISLCSIMETSIVCNNDYALPMSSELINCLQGDTGVCAREKVISSYVPKF
29 DarV-K     99.6%  40.6%     MPISFNVDGDEWVTLAPRFVLIRTTLLSNIDTSMCTIIDSSVICNNDYALPMSTELINCLQGMTESCAREKVISSYVPKF
30 DarV-C     99.6%  40.4%     MPISFNVDGDEWVTLAPRFVLTRTTLLSNIDTSMCTIIDSSVICNNDYALPMSTELINCLQGMTESCAREKVISSYVPKF
31 LayV-1     98.9%  40.8%     MPISYNVDGDEWVTLVPRFVLTRTTLLSNIDTSRCTVTESSVICDNDYALPMSYELIGCLQGDTSKCAREKVVSSYVPRF
32 LayV-2     98.9%  40.8%     MPISYNVDGDEWVTLVPRFVLTRTTLLSNIDTSRCTVTESSVICDNDYALPMSYELIGCLQGDTSKCAREKVVSSYVPRF
33 MojV       99.5%  41.2%     MPISYNIDGDEWVTLVPRFVLTRTTLLSNIDTSRCTITDSSVICDNDYALPMSHELIGCLQGDTSKCAREKVVSSYVPKF
34 SHNV5-1    99.8%  40.8%     MPISFNSEGDEWVTLMPRFILTRTTLLSNIDITKCTVTDRSVICDNDYALPMSNQLIECLRGDTMKCTREKVMSSYIPKF
35 SHNV5-2    99.8%  40.8%     MPISFNSEGDEWVTLMPRFILTRTTLLSNIDITKCTVTDRSVICDNDYALPMSNQLIECLRGDTMKCTREKVMSSYIPKF

               cov    pid 401        .         .         .         .         :         .         .         . 480
 1 NiV-M1    100.0% 100.0%     ALSNGVLFANCISVTCQCQTTGRAISQSGEQTLLMIDNTTCPTAVLGNVIISLGKYLGSVNYNSEGIAIGPPVFTDKVDI
 2 NiV-M2    100.0%  99.8%     ALSNGVLFANCISVTCQCQTTGRAISQSGEQTLLMIDNTTCPTAVLGNVIISLGKYLGSVNYNSEGIAIGPPVFTDKVDI
 3 NiV-M3    100.0%  99.8%     ALSNGVLFANCISVTCQCQTTGRAISQSGEQTLLMIDNTTCPTAVLGNVIISLGKYLGSVNYNSEGIAIGPPVFTDKVDI
 4 NiV-M4    100.0%  99.5%     ALSNGVLFANCISVTCQCQTTGRAISQSGEQTLLMIDNTTCPTAVLGNVIISLGKYLGSVNYNSEGIAIGPPVFTDKVDI
 5 NiV-C     100.0%  98.9%     ALSNGVLFANCISVTCQCQTTGRAISQSGEQTLLMIDNTTCPTAVLGNVIISLGKYLGSVNYNSEGIAVGPPVFTDKVDI
 6 NiV-B1.1  100.0%  98.5%     ALSNGVLFANCISVTCQCQTTGRAISQSGEQTLLMIDNTTCPTAVLGNVIISLGKYLGSVNYNSEGIAIGPPVFTDKVDI
 7 NiV-B1.2  100.0%  99.1%     ALSNGVLFANCISVTCQCQTTGRAISQSGEQTLLMIDNTTCPTAVLGNVIISLGKYLGSVNYNSEGIAIGPPVFTDKVDI
 8 NiV-B1.3  100.0%  98.9%     ALSNGVLFANCISVTCQCQTTGRAISQSGEQTLLMIDNTTCPTAVLGNVIISLGKYLGSVNYNSEGIAIGPPVFTDKVDI
 9 NiV-B1.4  100.0%  98.7%     ALSNGVLFANCISVTCQCQTTGRAISQSGEQTLLMIDNTTCPTAVLGNVIISLGKYLGSINYNSEGIAIGPPVFTDKVDI
10 NiV-B2.3  100.0%  98.5%     ALSNGVLFANCISVTCQCQTTGRAISQSGEQTLLMIDNTTCPTAVLGNVIISLGKYLGSVNYNSEGIAIGPPVFTDKVDI
11 NiV-B3.1  100.0%  98.2%     ALSNGVLFANCISVTCQCQTTGRAISQSGEQTLLMIDNTTCPTAVLGNVIISLGKYLGSVNYNSEGIAIGPPVFTDKVDI
12 HeV-α1.1   99.8%  88.1%     ALSGGVLFANCISVTCQCQTTGRAISQSGEQTLLMIDNTTCTTVVLGNIIISLGKYLGSINYNSESIAVGPPVYTDKVDI
13 HeV-α2     99.8%  88.3%     ALSGGVLFANCISVTCQCQTTGRAISQSGEQTLLMIDNTTCTTVVLGNIIISLGKYLGSINYNSESIAVGPPVYTDKVDI
14 HeV-β1    100.0%  87.9%     ALSGGVLFANCISVTCQCQTTGRAISQSGEQTLLMIDNTTCTTVVLGNIIISLGKYLGSTNYNSESIAVGPPVYTDRVDI
15 HeV-β2    100.0%  87.7%     ALSGGVLFANCISVTCQCQTTGRAISQSGEQTLLMIDNTTCTTVVLGNIIISLGKYLGSTNYNSESIAVGPPVYTDRVDI
16 CedV-1     99.6%  42.9%     ALTNGVIFANCINTICRCQDNGKTITQNINQFVSMIDNSTCNDVMVDKFTIKVGKYMGRKDINNINIQIGPQIIIDKVDL
17 GhV(+A)    99.6%  51.5%     AISGGVIYANCLSTTCQCYQTGKVIAQDGSQTLMMIDNQTCSIVRIEEILISTGKYLGSQEYNTMHVSVGNPVFTDKLDI
18 AngV       98.0%  39.3%     ALSEGVIYANCLATSCKCATTNKPIVQSTSTVITMIDNSKCPVVEVGKQMISVGSYLGQVSPNNLTIEIGPPVYTEPVDI
19 SHNV1      98.4%  37.7%     ALSQGVIFANCIATTCRCADDGRAISQSSSQTVLLLTSKDCKVYEVQSMMISTGEYLGESIFENTDIPLGPSIVIDKIDI
20 NinV       99.8%  38.0%     ALSGGVIYANCLNTQCACATNGRSISQSSRQTIMMLDDKDCKIYEVGGIFISVSTYMGIGKFENENISIGPPVVVDKIDV
21 SHNV2      99.1%  40.6%     ALSGGVIYANCLSTVCRCIDKDQPISQSLSQTLMMLDNQHCNVYQISNVLISTGRYLGDAEFRNEGIDLGPPIVVDKIDL
22 GakV-1     99.6%  39.9%     ALSGGVIYANCLSAVCRCAVDGVPIVQSLKATVMMLDNKSCRVYQIGELLISTGAYLGSIEFKNENIELGPPIVIDKIDL
23 GakV-2     99.6%  39.9%     ALSGGVIYANCLSAVCRCAVDGVPIVQSLKATVMMLDNKNCRVYQIGELLISTGAYLGSIEFKNENIELGPPIVIDKIDL
24 SHNV3      99.6%  39.6%     ALSGGVIYANCLSAVCRCAVDGVPIVQSLKTTIMMLDNKNCRVYQIGELLISTGAYLGSVEFKNENIELGPPIVVDKIDL
25 ShyV       99.8%  38.7%     ALSGGVIYANCLSAVCRCAVDGVPIVQSLKTTIMMLDNKNCRVYQIGELLISTGSYLGSVEFKNENIELGPPIVVDKIDL
26 SHNV4      99.6%  40.6%     ALSGGVIYANCLSTVCRCAVDGVPIVQSLKATIMMLDNKNCRVYQIGELLISTGAYLGSVEFKNENIDLGPPIVIDKVDL
27 MelV       99.1%  39.7%     ALSDGVVYANCLSTVCRCMDNGVPISQSLKSTVMMLDDKKCTIYQIGDILISVGKYMGHIDYNPENVVLGPPIVIDKIDI
28 DewV       99.1%  40.1%     ALSGGVVYANCLSTVCRCMDNGTPISQGIRHTVALLDNKKCSVYQVGDILISVGKYLGHLDYNTEDIVLGPPIVIDKIDI
29 DarV-K     99.6%  40.6%     ALSGGVIYANCLSTVCRCMDNQKPISQSLRSTVVMLDNKMCKVYQIGDILISVGEYKGAVEYNPEDVHLGPPIVLDKIDI
30 DarV-C     99.6%  40.4%     ALSGGVIYANCLSTVCRCMDNQKPISQSLRSTVVMLDNKMCKVYQIGDILISVGEYKGAVEYNPEDVHLGPPIVLDKIDI
31 LayV-1     98.9%  40.8%     ALSDGLVYANCLNTICRCMDTDTPISQSLGTTVSLLDNKKCLVYQVGDILISVGSYLGEGEYSADNVELGPPVVIDKIDI
32 LayV-2     98.9%  40.8%     ALSDGLVYANCLNTICRCMDTDTPISQSLGTTVSLLDNKKCLVYQVGDILISVGSYLGEGEYSADNVELGPPVVIDKIDI
33 MojV       99.5%  41.2%     ALSDGLVYANCLNTICRCMDTDTPISQSLGATVSLLDNKRCSVYQVGDVLISVGSYLGDGEYNADNVELGPPIVIDKIDI
34 SHNV5-1    99.8%  40.8%     ALSDGVVYANCLNTICRCMDTDTPITQSLRSTVTLLDNKACLVYQIGDILISVGSYLGNTEYNTQNITLGPPIVIDKIDI
35 SHNV5-2    99.8%  40.8%     ALSDGVVYANCLNTICRCMDTDTPITQSLRSTVTLLDNKACLVYQIGDILISVGSYLGNTEYNTQNITLGPPIVIDKIDI

               cov    pid 481        .         5         .         .         .         .         :         . 560
 1 NiV-M1    100.0% 100.0%     SSQISSMNQSLQQSKDYIKEAQRLLDTVNPSLISMLSMIILYVLSIASLCIGLITFISFIIVEKKRNTYSRL--------
 2 NiV-M2    100.0%  99.8%     SSQISSMNQSLQQSKDYIKEAQRLLDTVNPSLISMLSMIILYVLSIASLCIGLITFISFIIVEKKRNTYSRL--------
 3 NiV-M3    100.0%  99.8%     SSQISSMNQSLQQSKDYIKEAQRLLDTVNPSLISMLSMIILYVLSIASLCIGLITFISFIIVEKKRNTYSRL--------
 4 NiV-M4    100.0%  99.5%     SSQKSSMNQSLQQSKDYIKEAQRLLDTVNPSLISMLSMIILYVLSIASLCIGLITFISFIIVEKKRNTYSRL--------
 5 NiV-C     100.0%  98.9%     SSQISSMNQSLQQSKDYIKEAQRLLDTVNPSLISMLSMIILYVLSIASLCIGLITFISFIIVEKKRNTYSRL--------
 6 NiV-B1.1  100.0%  98.5%     SSQISSMNQSLQQSKDYIKEAQRLLDTVNPSLISMLSMIILYVLSIASLCIGLITFISFIIVEKKRNTYSRL--------
 7 NiV-B1.2  100.0%  99.1%     SSQISSMNQSLQQSKDYIKEAQRLLDTVNPSLISMLSMIILYVLSIASLCIGLITFISFIIVEKKRNTYSRL--------
 8 NiV-B1.3  100.0%  98.9%     SSQISSMNQSLQQSKDYIKEAQRLLDTVNPSLISMLSMIILYVLSIASLCIGLITFISFIIVEKKRNTYSRL--------
 9 NiV-B1.4  100.0%  98.7%     SSQISSMNQSLQQSKDYIKEAQRLLDTVNPSLISMLSMIILYVLSIASLCIGLITFISFIIVEKKRNTYSRL--------
10 NiV-B2.3  100.0%  98.5%     SSQISSMNQSLQQSKDYIKEAQRLLDTVNPSLISMLSMIILYVLSIASLCIGLITFISFIIVEKKRNTYSRL--------
11 NiV-B3.1  100.0%  98.2%     SSQISSMNQSLQQSKDYIKEAQRLLDTVNPSLISMLSMIILYVLSIASLCIGLITFISFIIVEKKRNTYSRL--------
```

```
12 HeV-α1.1   99.8%  88.1%    SSQISSMNQSLQQSKDYIKEAQKILDTVNPSLISMLSMIILYVLSIAALCIGLITFISFVIVEKKRGNYSRL--------
13 HeV-α2     99.8%  88.3%    SSQISSMNQSLQQSKDYIKEAQKILDTVNPSLISMLSMIILYVLSIAALCIGLITFISFVIVEKKRGNYSRL--------
14 HeV-β1    100.0%  87.9%    SSQISSMNQSLQQSKDYIKEAQRILDTVNPSLISMLSMIVLYVLSTAALCIGLITLISFIIVEKKRGNYSRL--------
15 HeV-β2    100.0%  87.7%    SSQISSMNQSLQQSKDYIKEAQRILDTVNPSLISMLSMIVLYVLSTAALCIGLITLISFIIVEKKRGNYSRL--------
16 CedV-1      99.6%  42.9%    SNEINKMNQSLKDSIFYLREAKRILDSVNISLISPSVQLFLIIISVLSFIILLIIIVYLYCKSKHSYKYNKFIDDPDYYN
17 GhV(+A)     99.6%  51.5%    TSQISNINQSIEQSKFYLDKSKAILDKINLNLIGSVPISILFIIAILSLILSIITFVIVMIIVRRYNKYTPLIN------
18 AngV        98.0%  39.3%    TNQLGNINETLSKTLDLIKQSNSILDMITGGLNPQIGSMISLILAAIAILLSGITCLFSTKSYVKCNKLQMNC-------
19 SHNV1       98.4%  37.7%    SGQLAEINKTLDHVDNTIKDSNDILDKIDVSTVSAISMVILYVVIALIGFMSALSLLLSVRTFSRCTTLGSQF-------
20 NinV        99.8%  38.0%    SGQLAEVNQSLSKTEELLEESNEYLSQVHVSLVSLKSMIILYVFIGLIGLLAAAALLVSVRSLSMLKTMGPAT-------
21 SHNV2       99.1%  40.6%    GGQIADINQTISDAEEFIEESNKILSKINPKIISVKSMVVLYVVVALIAILAMVSLVLSIRLTMQVGTKINQF-------
22 GakV-1      99.6%  39.9%    GGQIAGINQTLQGVEDYIDKSNEILDQVNPSVTSLGAMIIVYIFIALAILLGLIALIMDVKLNSQVKILINQSMINQMRA
23 GakV-2      99.6%  39.9%    GGQIAGINQTLQGVEDYIDKSNDILDQVNPSVTSLGAMIIVYIFIALAILLGLIALIMDVKLNSQVKILINQSMINQMRA
24 SHNV3       99.6%  39.6%    GGQIAGINQTLQGVEDYIDKSNEILDQVNPSVTSLGAMIIVYIFIALAILIGLIALMIDVKLNSQIKILINQSIINQMRA
25 ShyV        99.8%  38.7%    GGQIAGINQTLQGVEDYIDKSNEILDQVNPSVTSLGAMIIVYIFIALAILIGLIALMIDVKLNSQIKILINQSIINQMRA
26 SHNV4       99.6%  40.6%    GGQIAGINQTLQGVEDYIDKSNEILDQVNPSVTSLGAMIIVYIFIALAILIGLIALMIDVKLNSQIKILINQSIVNQMRA
27 MelV        99.1%  39.7%    GNQLAGINQTLQEAGDFIEKSEEILNSINPSVLTLSSMITLYIFLIITVIIAVAALVLSIRLTIKARILTNQF-------
28 DewV        99.1%  40.1%    GNQLAGINSSLQQAENYIDKSNEILKSINPTIISTNSMITLYIFIIITVIIAITSLVLSLRLTMKAKILTNQF-------
29 DarV-K      99.6%  40.6%    GNQLAGINQTLSEAGDFISKSEEILKDINPAILSVSSMIVLYIFIIVITIISLTSLILSIRLTIKAKIFNNQF-------
30 DarV-C      99.6%  40.4%    GNQLAGINQTLSEAGDFISKSEEILKDINPAILSVSSMIVLYIFIIVITIISLTSLILSIRLTIKAKIFNNQF-------
31 LayV-1      98.9%  40.8%    GNQLAGINQTLQNAEDYIEKSEEFLKGINPSIITLGSMAVLYIFMIVIAVISIIALVLSIKLTVKGNVVRQQF-------
32 LayV-2      98.9%  40.8%    GNQLAGINQTLQNAEDYIEKSEEFLKGINPSIITLGSMAVLYIFMIVIAVISIIALVLSIKLTVKGNVVRQQF-------
33 MojV        99.5%  41.2%    GNQLAGINQTLQEAEDYIEKSEEFLKGVNPSIITLGSMVVLYIFMILIAIVSVIALVLSIKLTVKGNVVRQQF-------
34 SHNV5-1     99.8%  40.8%    GNQLAGINQSLQNAEDYIEKSDEFLKGINPSVITLGSMVVLYIFMILIAIISIIALVLSIRLTTKSNMQKAQF-------
35 SHNV5-2     99.8%  40.8%    GNQLAGINQSLQNAEDYIEKSEEFLKGINPSVITLGSMVVLYIFMILIAIISIIALVLSIRLTTKSNMQKAQF-------

                cov    pid 561        .         .         .         6         .         ] 620
 1 NiV-M1     100.0% 100.0%    -----EDRRVR----PTSSGDLYYIGT---------------------------------
 2 NiV-M2     100.0%  99.8%    -----EDRRVR----PTSSGDLYYIGT---------------------------------
 3 NiV-M3     100.0%  99.8%    -----EDRRVR----PTSSGDLYYIGT---------------------------------
 4 NiV-M4     100.0%  99.5%    -----EDRRVR----PTSSGDLYYIGT---------------------------------
 5 NiV-C      100.0%  98.9%    -----EDRRVR----PTSSGDLYYIGT---------------------------------
 6 NiV-B1.1   100.0%  98.5%    -----EDRRVR----PTSSGDLYYIGT---------------------------------
 7 NiV-B1.2   100.0%  99.1%    -----EDRRVR----PTSSGDLYYIGT---------------------------------
 8 NiV-B1.3   100.0%  98.9%    -----EDRRVR----PTSSGDLYYIGT---------------------------------
 9 NiV-B1.4   100.0%  98.7%    -----EDRRVR----PTSSGDLYYIGT---------------------------------
10 NiV-B2.3   100.0%  98.5%    -----EDRRVR----PTSSGDLYYIGT---------------------------------
11 NiV-B3.1   100.0%  98.2%    -----EDRRVR----PTSNGDLYYIGT---------------------------------
12 HeV-α1.1   99.8%  88.1%    -----DDRQVR----PVSNGDLYYIGT---------------------------------
13 HeV-α2     99.8%  88.3%    -----DDRQVR----PVSNGDLYYIGT---------------------------------
14 HeV-β1    100.0%  87.9%    -----DDRRVR----PVSNGDLYYIGT---------------------------------
15 HeV-β2    100.0%  87.7%    -----DDRRVR----PVSNGDLYYIGT---------------------------------
16 CedV-1      99.6%  42.9%    -----DYKRERINGKASKSNNIYYVGD---------------------------------
17 GhV(+A)     99.6%  51.5%    -----SDPSSR----RSTIQDVYIIPNPGEHSIRSAARSIDRDRD---------------
18 AngV        98.0%  39.3%    ---FKSYERMD----P-----IYHSQQN--------------------------------
19 SHNV1       98.4%  37.7%    -----AYQRQD----P-TLGDVHYAFTSNIPKNQRSKNSNNLSNDLGSTNGSFSSNE---
20 NinV        99.8%  38.0%    -----LMNNIQ----P-TMHNLQYSGHSTINSKSSLINTHQSSHNHKYGSVNGSYTSDES
21 SHNV2       99.1%  40.6%    -----AYTKHV----P-SMENVQYIGTR--------------------------------
22 GakV-1      99.6%  39.9%    GLENPGYSRSL----P-SFGSIASRGSSQELVNTN-------------------------
23 GakV-2      99.6%  39.9%    GLENPGYSRSL----P-SFGSVASRGSSQELVNTN-------------------------
24 SHNV3       99.6%  39.6%    GLENPGYSKSL----P-SFSSTVSKGSSQELINTH-------------------------
25 ShyV        99.8%  38.7%    GLENPGYSRSL----P-SFSSTVSKGSSQELINTH-------------------------
26 SHNV4       99.6%  40.6%    GLENPGYSRSL----P-SFSSIASKGSSQELVNTN-------------------------
27 MelV        99.1%  39.7%    -----AYGRHS----P-SMDNVSYVSK---------------------------------
28 DewV        99.1%  40.1%    -----AYGRHS----P-SMDNVSYVSR---------------------------------
29 DarV-K      99.6%  40.6%    -----AYGRHS----P-SMDNVSYVTH---------------------------------
30 DarV-C      99.6%  40.4%    -----AYGRHS----P-SMDNVSYVTH---------------------------------
31 LayV-1      98.9%  40.8%    -----AYTQHV----P-SMENVNYVSH---------------------------------
32 LayV-2      98.9%  40.8%    -----AYTQHV----P-SMENVNYVSH---------------------------------
33 MojV        99.5%  41.2%    -----TYTQHV----P-SMENINYVSH---------------------------------
34 SHNV5-1     99.8%  40.8%    -----SYTRQA----P-SMDNINYVSR---------------------------------
35 SHNV5-2     99.8%  40.8%    -----SYTRQA----P-SMDNINYVSR---------------------------------
```

MView 1.67, Copyright © 1997-2020 Nigel P. Brown
