# Supplementary material for: Structural and antigenic characterization of novel and diverse Henipavirus glycoproteins
Source: bioRxiv. 2024 Dec 11:2024.12.11.627382. Preprint. [Version 1] doi: 10.1101/2024.12.11.627382 (PMC11661166; doi:10.1101/2024.12.11.627382)
Supplement: Supplementary file 3 [file media-3.pdf]

```
Reference sequence (1): NiV-M3
Identities normalised by aligned length.
Colored by: identity

                cov    pid   1 [       .       .       .       .       :       .       .       . 80
 1 NiV-M3     100.0% 100.0%    ------------MPAENKKVRFENT---------TSDKGKNPSKVIKSYY---GTMDIKK--------------------
 2 NiV-M5     100.0%  99.5%    ------------MPTENKKVRFENT---------TSDKGKIPSKVIKSYY---GTMDIKK--------------------
 3 NiV-B1.5   100.0%  95.7%    ------------MPTESKKVRFENT---------ASDKGKNPSKVIKSYY---GTMDIKK--------------------
 4 NiV-B1.6   100.0%  95.3%    ------------MPTESKKVRFENT---------ASDKGKNPSKVIKSYY---GTMDIKK--------------------
 5 NiV-B2.4   100.0%  95.3%    ------------MPTESKKVRFENT---------ASDKGKNPSKVIKSYY---GTMDIKK--------------------
 6 NiV-B2.5   100.0%  95.8%    ------------MPTESKKVRFENT---------ASDKGKNPSKVIKSYY---GTMDIKK--------------------
 7 NiV-B3.1   100.0%  95.5%    ------------MPTESKKVRFENT---------ASDKGKNPSKVIKSYY---GTMDIKK--------------------
 8 NIV-I1.3   100.0%  95.2%    ------------MPTESKKVRFENT---------ASDKGKNPSKVIKSYY---GTMDIKK--------------------
 9 HeV-α1.2 100.0%  78.1%    ------------MMADSKLVSLNNN---------LSGKIKDQGKVIKNYY---GTMDIKK--------------------
10 HeV-α3   100.0%  78.3%    ------------MMADSKLVSLNNN---------LSGKIKDQGKVIKNYY---GTMDIKK--------------------
11 HeV-α4   100.0%  78.0%    ------------MMADSKLVSLNNN---------LSGKIKDQGKVIKNYY---GTMDIKK--------------------
12 HeV-α5   100.0%  78.3%    ------------MMADSKLVSPNNN---------LSGKIKDQGKVIKNYY---GTMDIKK--------------------
13 HeV-α6   100.0%  78.3%    ------------MMADSKLVSLNNN---------LSGKIKDQGKVIKNYY---GTMDIKK--------------------
14 HeV-β1      99.8%  78.8%    -------------MAESKVVRPSSS---------LSSKIKDQGKIIKNYY---GTMDIKK--------------------
15 HeV-β3      99.8%  79.1%    -------------MAESKVVRPSSS---------LSSKIKDQGKIIKNYY---GTMDIKK--------------------
16 CedV-1       98.0%  28.9%    ------------MLSQLQKNYLDNS-------NQQGDKMNNPDKKLSVNF---NPLELDKGQKDLNKSYYVKNKNYNVSN
17 CedV-2       98.0%  29.1%    ------------MLSQLQKNYLDNS-------NQQGDKMNNPDKKLSVNF---NPLELDKGQKDLNKSYYVKNKNYNISN
18 GhV          98.3%  24.9%    ------------MPQKTVEFINMNSPLERGVSTLSDKKTLNQSKITKQGYFGLGSHSERN--------------------
19 AngV         98.0%  16.9%    -------------MSQKKSLIIKGH-----------DYSPKDKNQSSKYY---NSYESDQ--------------------
20 LayV-2       96.8%  19.3%    -------------MATNKKDVIKTT-------------ESTREDKVKKFY---GVETAEK--------------------
21 LayV-3       96.8%  19.2%    -------------MATNKRDVIKTT-------------ESTREDKVKKFY---GVETAEK--------------------
22 MojV         96.8%  19.6%    -------------MATNRDNTITSA-------------EVSQEDKVKKYY---GVETAEK--------------------
23 SHNV5-1      97.7%  18.9%    MQNRRTSSDPVNTQNSNNRPRIENGGPMNPTMGNQPRIQTARSDPARKYY---GVETAER--------------------
24 SHNV5-3      97.7%  18.7%    MQNRRTSSDLANTQNSNNRPRTENGGPMNSTMGNQPRIQTARSDPVRKYY---GVETAER--------------------
25 DarV-K       97.5%  19.0%    -------------MDKIMANKNNNN-----------NKVNTRETTVKKFY---GVDTAEK--------------------
26 DarV-C       97.5%  19.0%    -------------MDKIMANKNNNN-----------NKVNTRETTVKKFY---GVDTAEK--------------------
27 DewV         96.7%  18.2%    ------------------MANTNTS-----------NKVQTRESTVKKFY---GVDTAEK--------------------
28 MelV         97.0%  18.8%    ----------------MSGPAKQNN-----------PKINTRETNVKKFY---GVETAEK--------------------
29 NinV         97.5%  16.9%    -----------MTQISTGSERTMST------------EKQEFENAARRYY---GVEKAEN--------------------
30 SHNV2        96.3%  16.3%    ------------MTDDNKMVKVGTE-----------------TTTPHKYY---GVEKVDN--------------------
31 GakV-1       96.5%  17.2%    -----------------MSPKIDTN-------------KTAPISDSRKYY---GVDSVER--------------------
32 GakV-2       96.5%  17.1%    -----------------MSPKIDTN-------------KTAPISDSRKYY---GVDSVER--------------------
33 SHNV3        96.3%  17.4%    ------------------MSKVDTS-------------KTTSTTNSRKYY---GVESVEK--------------------
34 ShyV         96.3%  17.1%    ------------------MSKVDTS-------------KTTSTTNSRKYY---GVESVEK--------------------

                cov    pid  81         .       1       .       .       .       .       :       . 160
 1 NiV-M3     100.0% 100.0%    -INEGLLDS--K-ILSAFNTVIALLGSIVIIVMNIMIIQNYTRSTDNQAVIKDALQGIQQ----QIKGLADKIGTEIGPK
 2 NiV-M5     100.0%  99.5%    -INEGLLDS--K-ILSAFNTVIALLGSIVIIVMNIMIIQNYTRSTDNQAVIKDALQGIQQ----QIKGLADKIGTEIGPK
 3 NiV-B1.5   100.0%  95.7%    -INEGLLDS--K-ILSAFNTVIALLGSIVIIVMNIMIIQNYTRSTDNQAMIKDALQSIQQ----QIKGLADKIGTEIGPK
 4 NiV-B1.6   100.0%  95.3%    -INEGLLDS--K-ILSAFNTVIALLGSIVIIVMNIMIIQNYTRSTDNQAMIKDALQSIQQ----QIKGLADKIGTEIGPK
 5 NiV-B2.4   100.0%  95.3%    -INEGLLDS--K-ILSAFNTVIALLGSIVIIVMNIMIIQNYTRSTDNQAMIKDALQSIQQ----QIKGLADKIGTEIGPK
 6 NiV-B2.5   100.0%  95.8%    -INEGLLDS--K-ILSAFNTVIALLGSIVIIVMNIMIIQNYTRSTDNQAMIKDALQSIQQ----QIKGLADKIGTEIGPK
 7 NiV-B3.1   100.0%  95.5%    -INEGLLDS--K-ILSAFNTVIALLGSIVIIVMNVMIIQNYTRSTDNQAMIKDALQSIQQ----QIKGLADKIGTEIGPK
 8 NIV-I1.3   100.0%  95.2%    -INEGLLDS--K-ILSAFNTVIALLGSIVIIVMNIMIIQNYTRSTDNQAMIKDALQSIQQ----QIKGLADKIGTEIGPK
 9 HeV-α1.2 100.0%  78.1%    -INDGLLDS--K-ILGAFNTVIALLGSIIIIVMNIMIIQNYTRTTDNQALIKESLQSVQQ----QIKALTDKIGTEIGPK
10 HeV-α3   100.0%  78.3%    -INDGLLDS--K-ILGAFNTVIALLGSIIIIVMNIMIIQNYTRTTDNQALIKESLQSVQQ----QIKALTDKIGTEIGPK
11 HeV-α4   100.0%  78.0%    -INDGLLDS--K-ILGAFNTVIALLGSIIIIVMNIMIIQNYTRTTDNQALIKESLQSVQQ----QIKALTDKIGTEIGPK
12 HeV-α5   100.0%  78.3%    -INDGLLDS--K-ILGAFNTVIALLGSIIIIVMNIMIIQNYTRTTDNQALIKESLQSVQQ----QIKALTDKIGTEIGPK
13 HeV-α6   100.0%  78.3%    -INDGLLDS--K-ILGAFNTVIALLGSIIIIVMNIMIIQNYTRTTDNQALIKESLQSVQQ----QIKALTDKIGTEIGPK
14 HeV-β1      99.8%  78.8%    -INDGLLDS--K-ILGAFNTVIALLGSIIIIVMNVMIIQNYARTTDNQALIKDSLQSVQQ----QIKALTDKIGTEIGPK
15 HeV-β3      99.8%  79.1%    -INDGLLDS--K-ILGAFNTVIALLGSIIIIVMNVMIIQNYTRTTDNQALIKDSLQSVQQ----QIKALTDKIGTEIGPK
16 CedV-1       98.0%  28.9%    LLNESLHDI--KFCIYCIFSLLIIITIINIITISI-VITRLKVHEENNGMESPNLQSIQD----SLSSLTNMINTEITPR
17 CedV-2       98.0%  29.1%    LLNESLHDI--KFCIYCIFSLLIIITIINIITISI-VITRLKVHEENNGMESPNLQSIQD----SLSSLTNMINTEITPR
18 GhV          98.3%  24.9%    -WKKQKNQN--D-HYMTVSTMILEILVVLGIMFNLIVLTMVYYQNDNINQRMAELTSNITVLNLNLNQLTNKIQREIIPR
19 AngV         98.0%  16.9%    ---DTINPNEFK-CMLTLIFLVLLLTIASVIVLGICNVVKLDHESKQISINTMSINKAIG----KINELYSLINNELKIR
20 LayV-2       96.8%  19.3%    -VADSISSN--K-VFILMNTLLILTGAIITITLNVTNLTTIKNQQAMLKIIQDEVNSKLE----MFVSLDQLVKGEIKPK
21 LayV-3       96.8%  19.2%    -VADSISSN--K-VFILMNTLLILTGAIITITLNVTNLTTIKNQQAMLKIIQDEVNSKLE----MFVSLDQLVKGEIKPK
22 MojV         96.8%  19.6%    -VADSISGN--K-VFILMNTLLILTGAIITITLNITNLTAAKSQQNMLKIIQDDVNAKLE----MFVNLDQLVKGEIKPK
23 SHNV5-1      97.7%  18.9%    -VADSISSN--K-VFILMNTLLILTGAIITITLNITNLNNAQNQQTMLKMIQDEVDLKLS----AFDNLEQLVKGDLKPK
24 SHNV5-3      97.7%  18.7%    -VADSISSN--K-VFILMNTLLILTGAIITITLNITNLNNAQNQQTMLKMIQDEVDLKLS----VFDNLEQLVKGDLKPK
25 DarV-K       97.5%  19.0%    -VADSIISN--K-IFILINTLLIITSSVITITLNITNLEGIKNQNAIIKTLQMEIMNKVE----TISKLEQIVKGDLKPK
26 DarV-C       97.5%  19.0%    -VADSIISN--K-IFILINTLLIITSSVITITLNITNLEGIKNQNAIIKTLQMEIMNKVE----TISKLEQIVKGDLKPK
27 DewV         96.7%  18.2%    -VADSISNN--K-LFILANSLLILTSTVIIITLNITNLEGIKHQNQVIKTLHDEIQAKID----VISQLETIIKGDLKPK
28 MelV         97.0%  18.8%    -VADSISNN--K-LFILANTLLILTSTVIIITLNITNLEGIKHQNEVIRNLHDEIKSKIE----TINNLEQIIKGDLKPK
29 NinV         97.5%  16.9%    -LAGTILNN--K-MFILMNTLLIITGSLITIGLNATNLTNISYQKTLVNNLDKEMRHKFD----VVSQLDEFLRGEIRPK
30 SHNV2        96.3%  16.3%    -FAGKVVSN--R-IFILTNMILIITSSIVAITLNVTNLESLYNQRETLAMVNQEVHNKLG----DVSTIGNTIKGDIKPK
31 GakV-1       96.5%  17.2%    -FADGVTNN--K-IFILANMLLIIMSSIVMISLNITNLENLNNQKTSLKILNEDVTNKLE----QIDTLDQIVKGEIKPK
32 GakV-2       96.5%  17.1%    -FADGVTNN--K-IFILANMLLIIMSSIVMISLNITNLENLNNQKTSLKILNEDVTNKLE----QIDTLDQIVKGEIKPK
33 SHNV3        96.3%  17.4%    -FADGVTNN--K-IFILANMLLIIMSSIVMISLNITNLENLNNQKTSLKILNDDVNNKLE----QIDTLDQIVKGEIKPK
34 ShyV         96.3%  17.1%    -FADGVTNN--K-IFILANMLLIIMSSIVMISLNITNLENLNNQKTSLKILNEDVNNKLE----QIDTLDQIVKGEIKPK

                cov    pid 161         .       .       .       2       .       .       .       . 240
 1 NiV-M3     100.0% 100.0%    VSLIDTSSTITIPANIGLLGSKISQSTASINENVNEKCK------------------FTLPPLKIHECNISCPNPLPFRE
```

```
 2 NiV-M5      100.0%  99.5%    VSLIDTSSTITIPANIGLLGSKISQSTASINENVNEKCK------------------FTLPPLKIHECNISCPNPLPFRE
 3 NiV-B1.5    100.0%  95.7%    VSLIDTSSTITIPANIGLLGSKISQSTASINENVNEKCK------------------FTLPPLKIHECNISCPNPLPFRE
 4 NiV-B1.6    100.0%  95.3%    VSLIDTSSTITIPANIGLLGSKISQSTASINENVNEKCK------------------FTLPPLKIHECNISCPNPLPFRE
 5 NiV-B2.4    100.0%  95.3%    VSLIDTSSTITIPANIGLLGSKISQSTASINENVNEKCK------------------FTLPPLKIHECNISCPNPLPFRE
 6 NiV-B2.5    100.0%  95.8%    VSLIDTSSTITIPANIGLLGSKISQSTASINENVNEKCK------------------FTLPPLKIHECNISCPNPLPFRE
 7 NiV-B3.1    100.0%  95.5%    VSLIDTSSTITIPANIGLLGSKISQSTASINENVNEKCK------------------FTLPPLKIHECNISCPNPLPFRE
 8 NIV-I1.3    100.0%  95.2%    VSLIDTSSTITIPANIGLLGSKISQSAASINENVNEKCK------------------FTLPPLKIHECNISCPNPLPFRE
 9 HeV-α1.2 100.0%  78.1%    VSLIDTSSTITIPANIGLLGSKISQSTSSINENVNDKCK------------------FTLPPLKIHECNISCPNPLPFRE
10 HeV-α3   100.0%  78.3%    VSLIDTSSTITIPANIGLLGSKISQSTSSINENVNDKCK------------------FTLPPLKIHECNISCPNPLPFRE
11 HeV-α4   100.0%  78.0%    VSLIDTSSTITIPANIGLLGSKISQSTSSINENVNDKCK------------------FTLPPLKIHECNISCPNPLPFRE
12 HeV-α5   100.0%  78.3%    VSLIDTSSTITIPANIGLLGSKISQSTSSINENVNDKCK------------------FTLPPLKIHECNISCPNPLPFRE
13 HeV-α6   100.0%  78.3%    VSLIDTSSTITIPANIGLLGSKISQSTSSINENVNDKCK------------------FTLPPLKIHECNISCPNPLPFRE
14 HeV-β1      99.8%  78.8%    VSLIDTSSTITIPANIGLLGSKISQSTSSINENVNDKCK------------------FTLPPLKIHECNISCPNPLPFRE
15 HeV-β3      99.8%  79.1%    VSLIDTSSTITIPANIGLLGSKISQSTSSINENVNDKCK------------------FTLPPLKIHECNISCPNPLPFRE
16 CedV-1       98.0%  28.9%    IGILVTATSVTLSSSINYVGTKTNQLVNELKDYITKSCG------------------FKVPELKLHECNISCADPKISKS
17 CedV-2       98.0%  29.1%    IGILVTATSVTLPSSINYVGTKTNQLVNELKDYITKSCG------------------FKVPELKLHECNISCADPKISKS
18 GhV          98.3%  24.9%    ITLIDTATTITIPSAITYILATLTTRISELLPSINQKCE------------------FKTPTLVLNDCRINCTPPLNPSD
19 AngV         98.0%  16.9%    MMVMDTAVNVDIPADISALSMKLTSAITSIEELIQSQTSSVPSNRPSMNQSGGSSGSSQLSNSSRPIPDQGNPNYCNYDK
20 LayV-2       96.8%  19.3%    VSLINTAVSVSIPGQISNLQTKFLQKYVYLEESITKQCTCNP-------LSGIFP--TTKPP-----PPPTDKPDDDTTD
21 LayV-3       96.8%  19.2%    VSLINTAVSVSIPGQISNLQTKFLQKYVYLEESITKQCTCNP-------LSGIFP--TTKPP-----PPPTDKPDDDTTD
22 MojV         96.8%  19.6%    VSLINTAVSVSIPGQISNLQTKFLQKYVYLEESITKQCTCNP-------LSGIFP--TSGPT-----YPPTDKPDDDTTD
23 SHNV5-1      97.7%  18.9%    VTLINSAVSVSIPSQISNLQNKLTQRISSLEDMVGRKCNFNP-------LATIFP--TVEPS-----KPPTTEEDEDTSD
24 SHNV5-3      97.7%  18.7%    VTLINSAVSVSIPSQISNLQNKLTQRISNLEDMVGRKCNFNP-------LATIFP--TVEPS-----KPPTTEEDEDTSD
25 DarV-K       97.5%  19.0%    VTLINSAVSVSIPGQISNLQTKMSQKLKFIEDSVIDQCTCNP-------LSGIFP--SKEPP---KEPHPGTEDDDDTSD
26 DarV-C       97.5%  19.0%    VTLINSAVSVSIPSQISNLQTKMSQKLKFIEDSVIDQCTCNP-------LSGIFP--SKEPP---KEPHPGTEDDDDTSD
27 DewV         96.7%  18.2%    ISLINSAVSVSLPGQITNLQTKLNQKIKLLEDQVQDQCTCNP-------LSGIFP--SRPPQ---PTPSPDDESDDDMSD
28 MelV         97.0%  18.8%    ISLINSAVSVSLPGQISNLQNKMLQKFKTLEDSVIDQCTCNP-------LSGIFP--SKPPH---TSKPDDEEDDNDFTD
29 NinV         97.5%  16.9%    INIISSATTVSLPTQLSQLSQRLMNQIAKLESMIVAQCSGAT--------STGAP--ANDPNKKPGEVDVDAPTLQPPED
30 SHNV2        96.3%  16.3%    VNLINSAVSVSLPAQMSSMLQKMLNKMNGIEDAIDQRCSCST-------LGSLLPGSSQRPP---TVSTPGAGDDDESTD
31 GakV-1       96.5%  17.2%    LTLVNSAVSVTIPSQISNLATKLNNQIKTLEVAVNKQCSGNI-------LGGLL---TPKPPTGSGTTSPDDGNPDDNVD
32 GakV-2       96.5%  17.1%    LTLVNSAVSVTIPSQISNLATKLNNQIKTLEVAVNKQCSGNI-------LGGLL---TPKPPTGSGTTSPDDGNSDDNVD
33 SHNV3        96.3%  17.4%    LTLVNSAVSVTIPSQLSNLATKLNNQIKTLEVAVNKQCSGNI-------LGGLL---TPKPPEGGDVINPDDNSTGDNTD
34 ShyV         96.3%  17.1%    LTLVNSAVSVTIPSQLSNLATKLNNQIKTLEVAVNKQCSGNI-------LGGLL---TPKPPEGGDVINPDDNSTGDNTD

                  cov    pid 241         :         .         .         .         .         3         .         . 320
 1 NiV-M3      100.0% 100.0%    YRPQTEGVSNLVGLPNNIC--LQKT----SNQILKPKLISYTLPVVGQ----SGTCITDPLLAMDEGYFAYSHLERIGSC
 2 NiV-M5      100.0%  99.5%    YRPQTEGVSNLVGLPNNIC--LQKT----SNQILKPKLISYTLPVVGQ----SGTCITDPLLAMDEGYFAYSHLERIGSC
 3 NiV-B1.5    100.0%  95.7%    YKPQTEGVSNLVGLPNNIC--LQKT----SNQILKPKLISYTLPVVGQ----SGTCITDPLLAMDEGYFAYSHLEKIGSC
 4 NiV-B1.6    100.0%  95.3%    YKPQTEGVSNLVGLPNNIC--LQKT----SNQILKPKLISYTLPVVGQ----SGTCITDPLLAMDEGYFAYSHLEKIGSC
 5 NiV-B2.4    100.0%  95.3%    YKPQTEGVSNLVGLPNNIC--LQKT----SNQILKPKLISYTLPIVGQ----SGTCITDPLLAMDEGYFAYSHLEKIGSC
 6 NiV-B2.5    100.0%  95.8%    YKPQTEGVSNLVGLPNNIC--LQKT----SNQILKPKLISYTLPVVGQ----SGTCITDPLLAMDEGYFAYSHLEKIGSC
 7 NiV-B3.1    100.0%  95.5%    YKPQTEGVSNLVGLPNNIC--LQKT----SNQILKPKLISYTLPVVGQ----SGTCITDPLLAMDEGYFAYSHLEKIGSC
 8 NIV-I1.3    100.0%  95.2%    YKPQTEGVSNLVGLPNNIC--LQKT----SNQILKPKLISYTLPVVGQ----SGTCITDPLLAMDEGYFAYSHLEKIGSC
 9 HeV-α1.2 100.0%  78.1%    YRPISQGVSDLVGLPNQIC--LQKT----TSTILKPRLISYTLPINTR----EGVCITDPLLAVDNGFFAYSHLEKIGSC
10 HeV-α3   100.0%  78.3%    YRPISQGVSDLVGLPNQIC--LQKT----TSTILKPRLISYTLPINTR----EGVCITDPLLAVDNGFFAYSHLEKIGSC
11 HeV-α4   100.0%  78.0%    YRPISQGVSDLVGLPNQIC--LQKT----TSTILKPRLISYTLPINTR----EGVCITDPLLAVDNGFFAYSHLEKIGSC
12 HeV-α5   100.0%  78.3%    YRPISQGVSDLVGLPNQIC--LQKT----TSTILKPRLISYTLPINTR----EGVCITDPLLAVDNGFFAYSHLEKIGSC
13 HeV-α6   100.0%  78.3%    YRPISQGVSDLVGLPNQIC--LQKT----TSTILKPRLISYTLPINTR----EGVCITDPLLAVDNGFFAYSHLEKIGSC
14 HeV-β1      99.8%  78.8%    YRPISQGVSDLVGLPNQIC--LQRT----TSTILKPKLISYTLPINTR----EGVCITDPLLTIDNGFFAYSHLEKIGSC
15 HeV-β3      99.8%  79.1%    YRPISQGVSDLVGLPNQIC--LQRT----TSTILKPKLISYTLPINTR----EGVCITDPLLTIDNGFFAYSHLEKIGSC
16 CedV-1       98.0%  28.9%    AMYSTNAYAELAGPPKIFC----------KSVSKDPDFRLKQIDYVIPVQQDRSICMNNPLLDISDGFFTYIHYEGINSC
17 CedV-2       98.0%  29.1%    AMYSTNAYAELAGPPKIFC----------KSVSKDPDFRLKQIDYVIPVQQDRSICMNNPLLDISDGFFTYIHYEGINSC
18 GhV          98.3%  24.9%    GVKMSSLATNLVAHGPSPC----------RNFSSVPTIYYYRIPGLYNRTALDERCILNPRLTISSTKFAYVHSEYDKNC
19 AngV         98.0%  16.9%    PNRPSMSFTSLNIITPTES--AYTEGCHTETVLTSEQFQLVPLNQLLAGDYGEDYCSRYPSLSLRGRRFTYTEERYRGSC
20 LayV-2       96.8%  19.3%    DDKVDTTIKPVEYYKPDGC--NKTN----DHFTMQPGVNFYTVPNLGPSSSSADECYTNPSFSIGSSIYMFSQEIRKTDC
21 LayV-3       96.8%  19.2%    DDKVDTTIKPVEYYKPDGC--NKTN----DHFTMQPGVNFYTVPNLGPSSSSADECYTNPSFSIGSSIYMFSQEIRKTDC
22 MojV         96.8%  19.6%    DDKVDTTIKPIEYPKPDGC--NRTG----DHFTMEPGANFYTVPNLGPASSNSDECYTNPSFSIGSSIYMFSQEIRKTDC
23 SHNV5-1      97.7%  18.9%    DDKVDISIKPIDYKSPINC--SKPN----DHPTMEPGPNFYAVPNLGPSSSNADECYTNPSFSIGSSIYMFSQEIRKTDC
24 SHNV5-3      97.7%  18.7%    DDKVDISIRPIDYKSPMNC--SKPN----DHPTMEPGPNFYAVPNLGPSSSNADECYTNPSFSIGSSIYMFSQEIRKTDC
25 DarV-K       97.5%  19.0%    DDKVDDSIQSFDYPSFTVC--NNSD----ETTSIIPGPNLYAVPNLSIKEDEEDECVTNPSFSVGTSIYMYSQEIRKTDC
26 DarV-C       97.5%  19.0%    DDKVDDSIQSFDYPGFTVC--NNSD----ETTSIIPGPNLYAVPNLSIKEDEEDECVTNPSFSVGTSIYMYSQEIRKTDC
27 DewV         96.7%  18.2%    DDKVDDSIQGIDYPNFMIC--NGTR----NELSITPGQNLYSVPSLTMREDHDDECVVNPSFSVGTSIYMYSQEIRKTDC
28 MelV         97.0%  18.8%    DDKVDDTIQGIDYPTFTTC--NKSD----HSMSITPGPNLYTVPSLTYREDNDDECVVNPSFSIGTSIYMYSQEIRKTDC
29 NinV         97.5%  16.9%    DD--QDSIQMINLSEYPMC--SNQQQYKEPHFNVTVGPIEYT--ELLGLNNSSDYCISSPSLAIEGSTYIYAQQVRKTNC
30 SHNV2        96.3%  16.3%    TD--SGSVTMIDLSAFTKCTLDRSK----PYAAYSLTGVAHNIPGLSVNIADNGDCVSNVAFAISEGIYVYTQEIRKTDC
31 GakV-1       96.5%  17.2%    D--LEGDMRPLNLAELNDC--SRYPQTPNPTFAISPDI--HPMPELTEPIQLKNHCAVFPTVALGESIYVYSHQIRKTPC
32 GakV-2       96.5%  17.1%    D--LEGDMRPLNLAELNDC--SRYPQTPNPTFAISPDI--HPMPELTEPIQLKNHCAVFPTVALGESIYVYSHQIRKTPC
33 SHNV3        96.3%  17.4%    D--LEGDMRPLNLAQLNDC--SNYPQAPQPTFAIDPDL--HPMPELTEPIQLKNHCAVFPTVALGESIYVYSHQIRKTPC
34 ShyV         96.3%  17.1%    D--LEGDMRPLNLAQLNDC--SNYPQAPQPTFAIDPDL--HPMPELTEPIQLKNHCAVFPTVALGESIYVYSHQIRKTPC

                  cov    pid 321         .         .         :         .         .         .         .         4 400
 1 NiV-M3      100.0% 100.0%    SRGVSKQRIIGVGEVLDRGDEVPSLFMTNVWAPPNPNTVYHCSAVYNNEFYYVLCAVST---VGDPILNSTY-WSGSLMM
 2 NiV-M5      100.0%  99.5%    SRGVSKQRIIGVGEVLDRGDEVPSLFMTNVWTPPNPNTVYHCSAVYNNEFYYVLCAVST---VGDPILNSTY-WSGSLMM
 3 NiV-B1.5    100.0%  95.7%    SRGVSKQRIIGVGEVLDRGDEVPSLFMTNVWTPSNPNTVYHCSAVYNNEFYYVLCAVSV---VGDPILNSTY-WSGSLMM
 4 NiV-B1.6    100.0%  95.3%    SRGVSKQRIIGVGEVLDRGDEVPSLFMTNVWTPSNPNTVYHCSAVYNNEFYYVLCAVSV---VGDPILNSTY-WSGSLMM
 5 NiV-B2.4    100.0%  95.3%    SRGVSKQRIIGVGEVLDRGDEVPSLFMTNVWTPSNPNTVYHCSAVYNNEFYYVLCAVSV---VGDPILNSTY-WSGSLMM
 6 NiV-B2.5    100.0%  95.8%    SRGVSKQRIIGVGEVLDRGDEVPSLFMTNVWTPSNPNTVYHCSAVYNNEFYYVLCAVSV---VGDPILNSTY-WSGSLMM
 7 NiV-B3.1    100.0%  95.5%    SRGVSKQRIIGVGEVLDRGDEVPSLFMTNVWTPSNPNTVYHCSAVYNNEFYYVLCAVSV---VGDPILNSTY-WSGSLMM
 8 NIV-I1.3    100.0%  95.2%    SRGVSKQRIIGVGEVLDRGDEVPSLFMTNVWTPSNPNTVYHCSAVYNSEFYYVLCAVSV---VGDPILNSTY-WSGSLMM
 9 HeV-α1.2 100.0%  78.1%    TRGIAKQRIIGVGEVLDRGDKVPSMFMTNVWTPPNPSTIHHCSSTYHEDFYYTLCAVSH---VGDPILNSTS-WTESLSL
10 HeV-α3   100.0%  78.3%    TRGIAKQRIIGVGEVLDRGDKVPSMFMTNVWTPPNPSTIHHCSSTYHEDFYYTLCAVSH---VGDPILNSTS-WTESLSL
```

```
11 HeV-α4   100.0%  78.0%     TRGIAKQRIIGVGEVLDRGDKVPSMFMTNVWTPPNPSTIHHCSSTYHEDFYYTLCAVSH---VGDPILNSTS-WTESLSL
12 HeV-α5   100.0%  78.3%     TRGIAKQRIIGVGEVLDRGDKVPSMFMTNVWTPPNPSTIHHCSSTYHEDFYYTLCAVSH---VGDPILNSTS-WTESLSL
13 HeV-α6   100.0%  78.3%     TRGIAKQRIIGVGEVLDRGDKVPSMFMTNVWTPPNPSTIHHCSSTYHEDFYYTLCAVSH---VGDPILNSTS-WTESLSL
14 HeV-β1    99.8%  78.8%      TRGIAKQRIIGVGEVLDRGDKVPSMFMTNVWTPPNPSTVHHCSSTYHDDFYYTLCAVSH---VGDPILNGTS-WIESLFV
15 HeV-β3    99.8%  79.1%      TRGIAKQRIIGVGEVLDRGDKVPSMFMTNVWTPPNPSTVHHCSSTYHDDFYYTLCAVSH---VGDPILNGTS-WIESLFM
16 CedV-1     98.0%  28.9%      KKSDSFKVLLSHGEIVDRGDYRPSLYLLSSHYHPYSMQVINCVPVTCNQSSFVFCHISN---NTKTLDNSDY-SSDEYYI
17 CedV-2     98.0%  29.1%      KKSDSFKVLLSHGEIVDRGDYRPSLYLLSSHYHPYSMQVINCVPVTCNQSSFVFCHISN---NTKTLDNSDY-SSDEYYI
18 GhV        98.3%  24.9%      TRGFKYYELMTFGEILEGPEKEPRMFSRSFYSPTNAVNYHSCTPIVTVNEGYFLCLECT---SSDPLYKANL-SNSTFHL
19 AngV       98.0%  16.9%      STRTKEAQIIVYGYITDFGYRHPTLMILDTFNPPNYDMIYRCTSVTKLSTGLVICLYTN--MTEFELYESPK--NTDLII
20 LayV-2     96.8%  19.3%      TTGEILSIQIVLGRIVDKGQQGPQASPLLVWSVPNPKIINSCAVAAGDETGWVLCSVTLTAASGEPI---PH-MFDGFWL
21 LayV-3     96.8%  19.2%      TTGEILSIQIVLGRIVDKGQQGPQASPLLVWSVPNPKIINSCAVAAGDETGWVLCSVTLTAASGEPI---PH-MFDGFWL
22 MojV       96.8%  19.6%      TAGEILSIQIVLGRIVDKGQQGPQASPLLVWAVPNPKIINSCAVAAGDEMGWVLCSVTLTAASGEPI---PH-MFDGFWL
23 SHNV5-1    97.7%  18.9%      IAGELQSVQIILGRIVDKGQQGPQASPLLVWSVPNPRTINSCAVAAGDEMGWVLCSVTSTAASGGPN---PH-TFDGFWL
24 SHNV5-3    97.7%  18.7%      IAGELQSVQIILGRIVDKGQQGPQASPLLVWSVPNPRTINSCAVAAGDEMGWVLCSVTLTAASGGPS---PH-TFDGFWL
25 DarV-K     97.5%  19.0%      KSGPLTGIKIILGRIVDKGQFGPQVSPLLVWDVPKPETINSCAVVAEDEVGWALCSITSIANSGEPI---PY-LLSGFKL
26 DarV-C     97.5%  19.0%      KSGPLTGIKIILGRIVDKGQFGPQVSPLLVWDVPKPETINSCAVVAEDEVGWALCSITSIANSGEPI---PY-LLSGFKL
27 DewV       96.7%  18.2%      KQGPLIGIKISLGRVVDKGQSGPQVSPLLVWDVPKPETISSCAVVAGDEIGWALCSTIMTAKSGEPI---PY-MFEGFKL
28 MelV       97.0%  18.8%      KQGPLVGIKITLGRIVDKGLSGPQVSPLLVWEVPKPELTGSCAVAAGNEVGWVLCSTTLAASSGEPI---PN-MFEGFKL
29 NinV       97.5%  16.9%      QFGEIVGIQVVMGRLVDKGRTVPSSSPLLRWEVPAPESDQSCAAAASRGTGWLFCVSGN-TVSGEFLSDGRL---LGVRL
30 SHNV2      96.3%  16.3%      LTGQKISQITTLGRIVDKGQSGPRVSPLSTYLEPDPSSKISCSVAASSGSGWVFCVLTT----GEGVNYSKFGPFIGFTL
31 GakV-1     96.5%  17.2%      KSEDTIHQRVSLGRIVDRGFSGPRASPLSTWDLQETEFLSSCSVAASGEIGWVLCVTTD-KFTRDTVYAGPY---TGLKL
32 GakV-2     96.5%  17.1%      KSEDTTHQRVSLGRIVDRGFSGPRASPLSTWDLQETEFLSSCSVAASGEIGWVLCVTTD-KFTRDTVYAGPY---TGLKL
33 SHNV3      96.3%  17.4%      KSEDTIHQRVSIGRIVDRGFSGPRASPLSTWDLQETGYLSSCSVAASGETGWVLCVTTD-KFTGATVYLGPY---TGLKL
34 ShyV       96.3%  17.1%      KSEDTIHQRVSIGRIVDRGFSGPRASPLSTWDLQETGYLSSCSVAASGETGWVLCVTTD-KFTGGTVYLGPY---TGLKL

                  cov    pid 401         .         .         .         .         :         .         .         . 480
 1 NiV-M3     100.0% 100.0%      TRLAVKPKSNGGGYNQHQLALRSIEKGRYDKVMPYGPSGIKQGDTLYFPAVGFLVRTEFKYNDSNCPITKCQ----YSKP
 2 NiV-M5     100.0%  99.5%      TRLAVKPKSNGGGYNQHQLALRSIEKGRYDKVMPYGPSGIKQGDTLYFPAVGFLVRTEFKYNDSNCPITKCQ----YSKP
 3 NiV-B1.5   100.0%  95.7%      TRLAVKPKNNGESYNQHQFALRNIEKGKYDKVMPYGPSGIKQGDTLYFPAVGFLVRTEFKYNDSNCPIAECQ----YSKP
 4 NiV-B1.6   100.0%  95.3%      TRLAVKPKNNGESYNQHQFALRNIEKGKYDKVMPYGPSGIKQGDTLYFPAVGFLVRTEFTYNDSNCPIAECQ----YSKP
 5 NiV-B2.4   100.0%  95.3%      TRLAVKPKNNGESYNQHQFALRNIEKGKYDKVMPYGPSGIKQGDTLYFPAVGFLVRTEFKYNDSNCPIAECQ----YSRP
 6 NiV-B2.5   100.0%  95.8%      TRLAVKPKSNGESYNQHQFALRNIEKGKYDKVMPYGPSGIKQGDTLYFPAVGFLVRTEFKYNDSNCPIAECQ----YSKP
 7 NiV-B3.1   100.0%  95.5%      TRLAVKPKNNGESYNQHQFALRNIEKGKYDKVMPYGPSGIKQGDTLYFPAVGFLVRTEFKYNDSNCPIAECQ----YSKP
 8 NIV-I1.3   100.0%  95.2%      TRLAVKPKNNGESYNQHQFALRNIEKGMYDKVMPYGPSGIKQGDTLYFPAVGFLVRTEFKYNDSNCPVAKCQ----YSKP
 9 HeV-α1.2 100.0%  78.1%    IRLAVRPKSDSGNYNQKYIAITKVERGKYDKVMPYGPSGIKQGDTLYFPAVGFLPRTEFQYNDSNCPIIHCK----YSKA
10 HeV-α3   100.0%  78.3%    IRLAVRPKSDNGDYNQKYIAITKVERGKYDKVMPYGPSGIKQGDTLYFPAVGFLPRTEFQYNDSNCPIIHCK----YSKA
11 HeV-α4   100.0%  78.0%    IRLAVRPKSDSGDYNQKYITITKVERGKYDKVMPYGPSGIKQGNTLYFPAVGFLPRTEFQYNDSNCPIIHCK----YSKA
12 HeV-α5   100.0%  78.3%    IRLAVRPKSDSGDYNQKYIAINKVERGKYDKVMPYGPSGIKQGDTLYFPAVGFLPRTEFQYNDSNCPIIHCK----YSKA
13 HeV-α6   100.0%  78.3%    IRLAVRPKSDSGDYNQKYIAITKVERGKYDKVMPYGPSGIKQGDTLYFPAVGFLPRTEFQYNDSNCPIIHCK----YSKA
14 HeV-β1    99.8%  78.8%     IRLAVRPKTDGGGYNQKYITMTKIERGKYDKVMPYGPSGIKQGDTLYFPAVGFLPRTEFQYNDSNCPIINCK----YSKA
15 HeV-β3    99.8%  79.1%     IRLAVRPKTDGGGYNQKYITMTKIERGKYDKVMPYGPSGIKQGDTLYFPAVGFLPRTEFQYNDSNCPIINCK----YSKA
16 CedV-1     98.0%  28.9%      TYFN--GIDRPKTKKIPINNMTADNR--YIHFTFSGGGGVCLGEEFIIPVTTVI--NTDVFTHDYCESFNCSVQTGKSLK
17 CedV-2     98.0%  29.1%      TYFN--GIDRPKTKKIPINNMTADNR--YIHFTFSGGGGVCLGEEFIIPVTTVI--NTDVFTHDYCESFNCSVQTGKSLK
18 GhV        98.3%  24.9%      VILR--HNKDEKIVSMPSFNLSTDQE--YVQIIPAEGGGTAESGNLYFPCIGRL--LHKRVTHPLCKKSNCS----RTDD
19 AngV       98.0%  16.9%      FEAF--DGSQHKGTILNSSKFIYLND--IDMFIPGTGGGIYLNKTIYLPGIVFT--TNPPDGNAGCPNSACT----NKDP
20 LayV-2     96.8%  19.3%      YKFE--PDTEVVAYRITGFAYLLDKV--YDSVFIGKGGGIQRGNDLYFQMFGLS--RNRQSIKALCEHGSCLGTGGGGYQ
21 LayV-3     96.8%  19.2%      YKFE--PDTEVVAYRITGFAYLLDKV--YDSVFIGKGGGIQRGNDLYFQMFGLS--RNRQGIKALCEHGSCLGTGGGGYQ
22 MojV       96.8%  19.6%      YKLE--PDTEVVSYRITGYAYLLDKQ--YDSVFIGKGGGIQKGNDLYFQMYGLS--RNRQSFKALCEHGSCLGTGGGGYQ
23 SHNV5-1    97.7%  18.9%      YRLT--PDTDVVSYRISGFAYLLDKV--YDSVYIGKGGGVQKGSDLYFQMYGIS--RNRASFTALCDHGSCPGVGGGGYQ
24 SHNV5-3    97.7%  18.7%      YRLT--PDTDVVSYRISGFAYLLDKV--YDSVYIGKGGGVQKGSDLYFQMYGIS--RNRASFTALCDHGSCPGVGGGGYQ
25 DarV-K     97.5%  19.0%      FKFE--PDSEVVQYVLGSKSFRLDRV--YHTLYVGKGGGAARYDGLYFEGFGVI--TNIGKDQPLCNHGKCSGS--GSYI
26 DarV-C     97.5%  19.0%      FKFE--PDSEVVQYVLGSKSFRLDRV--YHTLYVGKGGGAVRYDGLYFEGFGVI--TNIGKDQPLCNHEKCSGS--GSYT
27 DewV       96.7%  18.2%      FKFE--PDKDVVQYTLAPRAFRLDEK--YHTLNIGKGGGIIRGEELYFTGFGII--TNYKKVDPLCNHGICPGS--GSYL
28 MelV       97.0%  18.8%      YKFE--PEKEVVQYIITKRAFRLDNP--YHTMNIGKGGGIIKEDGLYFQGFGVI--YNSGKVDPLCDHGKCPGV--GSYM
29 NinV       97.5%  16.9%      FTFK--GGSLYHAVYVPQTSIRFSLP--IKTFMVGTGGGVYVDGKLYFVGYGTV--TAPHTGGYLCSFDDCPGI--TDAG
30 SHNV2      96.3%  16.3%      SHIT--EDGDVTTYNISLNVVNSNYA--FKSIFIGPGNGIVRGNNMYFVGYGAL--RDNTNYPSWCIYDGCPAD--PSYN
31 GakV-1     96.5%  17.2%      YKLS--IRGQKEEYSITANNITTDAV--IIALTLTRGSGVPKNNKLIFLGLAAV--RDVDTTGVLCPTWKCENI--NNNV
32 GakV-2     96.5%  17.1%      YKLS--IRGQKEEYSITANNITTDAV--IIALTLTRGSGVPKNNKLIFLGLAAV--RDVDTTGVLCPTWKCDNI--NNNV
33 SHNV3      96.3%  17.4%      YKLS--VRGQKEEYAITANDITSDAT--LIALTLTRGSGVSKNNKLIFLGIAAV--RDADRTGVLCPDWKCDNI--NNNI
34 ShyV       96.3%  17.1%      YKLS--VRGQKEEYAITASDITADTT--VIALTLTRGSGVSKNNKLIFLGLAAV--RDADRTGVLCPDWKCDNI--NNNI

                  cov    pid 481         .         5         .         .         .         .         :         . 560
 1 NiV-M3     100.0% 100.0%      ENCRLSM--GIRPNSHYILRSGLLKYNLSDGENPKVVFIEISDQRLSIGSPSKIYDSLGQPVFYQASFSWDTMIKFGDVL
 2 NiV-M5     100.0%  99.5%      ENCRLSM--GIRPNSHYILRSGLLKYNLSDGENPKVVFIEISDQRLSIGSPSKIYDSLGQPVFYQASFSWDTMIKFGDVL
 3 NiV-B1.5   100.0%  95.7%      ENCRLSM--GIRPNSHYILRSGLLKYNLSDEENSKIVFIEISDQRLSIGSPSKIYDSLGQPVFYQASFSWDTMIKFGDVQ
 4 NiV-B1.6   100.0%  95.3%      ENCRLSM--GIRPSSHYILRSGLLKYNLSDEENSKIVFIEISDQRLSIGSPSKIYDSLGQPVFYQASFSWDTMIKFGDVQ
 5 NiV-B2.4   100.0%  95.3%      ENCRLSM--GIRPNSHYILRSGLLKYNLSDEENSKIVFIEISDQRLSIGSPSKIYDSLGQPVFYQASFSWDTMIKFGDVQ
 6 NiV-B2.5   100.0%  95.8%      ENCRLSM--GIRPNSHYILRSGLLKYNLSDEENSKIVFIEISDQRLSIGSPSKIYDSLGQPVFYQASFSWDTMIKFGDVQ
 7 NiV-B3.1   100.0%  95.5%      ENCRLSM--GIRPNSHYILRSGLLKYNLSDEENSKIVFIEISDQRLSIGSPSKIYDSLGQPVFYQASFSWDTMIKFGDVQ
 8 NIV-I1.3   100.0%  95.2%      ENCRLSM--GIRPNSHYILRSGLLKYNLSDEEDSKIIFIEISDQRLSIGSPSKIYDSLGQPVFYQASFSWDTMIKFGDVQ
 9 HeV-α1.2 100.0%  78.1%    ENCRLSM--GVNSKSHYILRSGLLKYNLSLGGDIILQFIEIADNRLTIGSPSKIYNSLGQPVFYQASYSWDTMIKLGDID
10 HeV-α3   100.0%  78.3%    ENCRLSM--GVNSKSHYILRSGLLKYNLSLGGDIILQFIEIADNRLTIGSPSKIYNSLGQPVFYQASYSWDTMIKLGDVD
11 HeV-α4   100.0%  78.0%    ENCRLSM--GVNSKSHYILRSGLLKYNLSLGGDIILQFIEIADNRLTIGSPSKIYNSLGQPVFYQASYSWDTMIKLGDVD
12 HeV-α5   100.0%  78.3%    ENCRLSM--GVNSKSHYILRSGLLKYNLSLGGDIILQFIEIADNRLTIGSPSKIYNSLGQPVFYQASYSWDTMIKLGDVD
13 HeV-α6   100.0%  78.3%    ENCRLSM--GVNSKSHYILRSGLLKYNLSLGGDITLQFIEIADNRLTIGSPSKIYNSLGQPVFYQASYSWDTMIKLGDVD
14 HeV-β1    99.8%  78.8%     ENCRLSM--GINPRSHYVLRSGLLKYNLSLGEDTRLQFIEIADNRLTIGSPSKIYNSLGQPVFYQASYSWDTMIKLGDVD
15 HeV-β3    99.8%  79.1%     ENCRLSM--GINPRSHYVLRSGLLKYNLSLGEDTRLQFIEIADNRLTIGSPSKIYNSLGQPVFYQASYSWDTMIKLGDVD
16 CedV-1     98.0%  28.9%      EICSESL--RSPTNSSRYNLNGIMIISQNNMTDFKIQLNGITYNKLSFGSPGRLSKTLGQVLYYQSSMSWDTYLKAGFVE
17 CedV-2     98.0%  29.1%      EICSESL--RSPTNSSRYNLNGIMIISQNNMTDFKIQLNGITYNKLSFGSPGRLSKTLGQVLYYQSSMSWDTYLKAGFVE
18 GhV        98.3%  24.9%      ESCLKSY--YNQGSPQHQVVNCLIRIRNAQRDNPTWDVITVDLTNTYPGSRSRIFGSFSKPMLYQSSVSWHTLLQVAEIT
19 AngV       98.0%  16.9%      KLCRKGL--KYIFNNSYYMVNGFALISQKGRKEYEVTVTMIETDVYHFGSRSRVQYFNSRAKYYQAPNGWFSYPIYGNIY
```

```
20 LayV-2      96.8%  19.3%     VLCDRAV--MSFGSEESLISNAYLKVNDVASGKPTIISQTFPPSDSYKGSNGRIYTIGERYGIYLAPSSWNRYLRFGLTP
21 LayV-3      96.8%  19.2%     VLCDRAV--MSFGSEESLISNAYLKVNDVASGKPTIISQTFPPSDSYKGSNGRIYTIGERYGIYLAPSSWNRYLRFGLTP
22 MojV        96.8%  19.6%     VLCDRAV--MSFGSEESLITNAYLKVNDLASGKPVIIGQTFPPSDSYKGSNGRMYTIGDKYGLYLAPSSWNRYLRFGITP
23 SHNV5-1     97.7%  18.9%     TLCDKSM--TVLGSDESLVTSAYLRVGDIYSGKPVIVSQTFPPSDSYRGSNGRIYLIGDSYGIYLAPSSWNRYLRFGVTP
24 SHNV5-3     97.7%  18.7%     TLCDKSM--TVLGSDESLVTSAYLRVGDIYSGKPVIVSQTFPPSDSYRGSNGRIYLIGDSYGIYLAPSSWNRYLRFGVTP
25 DarV-K      97.5%  19.0%     PICLSAM--SMMGDTDFLVVSNIIKVVDANIGKPVITVQTFKIQDTYKGSHGRIYQMDNNYGIYLASSSWNRYLKFGTTN
26 DarV-C      97.5%  19.0%     PICLSAM--SMMGDTDFLVVSNIIKVVDTNIGKPVITVQTFKIQDTYKGSHGRIYQMDNNYGVYLASSSWNRYLKFGTTN
27 DewV        96.7%  18.2%     PVCKSAI--SFMGDEDLIVVDNIIKVVNVDIGTPVIDLKTFKASDTYRGSNGRLYAMGDNFGVYFSPSSWNRYLRFGTSS
28 MelV        97.0%  18.8%     PTCLSAM--SMMGDVDFVVVDNIIKVVDYDIGKPVIDLKTFKISETYRGSNGRIYDMGSKFGLYLASSSWNRFLKFGTSN
29 NinV        97.5%  16.9%     TKCGDALFPPISYDGKAMLVNCLVKVSLDNPEVPSIEVDFFHPGDTYAGSNGRVYKMDRGIGIYISPASWNPHPRFGITR
30 SHNV2       96.3%  16.3%     KYCEWSMRPDYFGRLPGL--NCIITITNYKESNRGILIRAIPNTNSYPAAGGRLYDLGDRVGLYFTTASWQGKLQFALFE
31 GakV-1      96.5%  17.2%     GSCVHSY--RLTADMNNYFMNVVVAVDVTPTGKMTASVSLLPMSESYIGSEGGVIDKPGGYGLMISNKGWFARIRYGQTD
32 GakV-2      96.5%  17.1%     GSCVHSY--RLTADMNNYFMNVVVAVDVTPTGKMTASVSLLPMSESYIGSEGGVIDKPGGYGLMISNKGWFARIRYGQTD
33 SHNV3       96.3%  17.4%     NSCVHSY--RLTSDNNNYFMNVVVTVDVSPAGKMIASVSLLPMSESYIGSEGSVIDKPGGYGLMISNKGWFARIRYGQTE
34 ShyV        96.3%  17.1%     NSCVHSY--RLTSDNNNYFMNVVVTVDVSPAGKMIASISLLPMSESYIGSEGSVIDKPGGYGLMISNKGWFARIRYGQTE

                cov    pid 561        .         .         .         6         .         .         .         . 640
 1 NiV-M3     100.0% 100.0%     TVNPLVVNWRN--NTVISRPGQSQCPRFNTCPEICWEGVYNDAFLIDRINWISAGVFLDSNQTAENP--VFTVFKDNEIL
 2 NiV-M5     100.0%  99.5%     TVNPLVVNWRN--NTVISRPGQSQCPRFNTCPEICWEGVYNDAFLIDRINWISAGVFLDSNQTAENP--VFTVFKDNEIL
 3 NiV-B1.5   100.0%  95.7%     TVNPLVVNWRD--NTVISRPGQSQCPRFNKCPEICWEGVYNDAFLIDRINWISAGVFLDSNQTAENP--VFTVFKDNEVL
 4 NiV-B1.6   100.0%  95.3%     TVNPLVVNWRD--NTVISRPGQSQCPRFNKCPEVCWEGVYNDAFLIDRINWISAGVFLDSNQTAENP--VFTVFKDNEVL
 5 NiV-B2.4   100.0%  95.3%     TVNPLVVNWRD--NTVISRPGQSQCPRFNKCPEVCWEGVYNDAFLIDRINWISAGVFLDSNQTAENP--VFTVFKDNEVL
 6 NiV-B2.5   100.0%  95.8%     TVNPLVVNWRD--NTVISRPGQSQCPRFNKCPEVCWEGVYNDAFLIDRINWISAGVFLDSNQTAENP--VFTVFKDNEVL
 7 NiV-B3.1   100.0%  95.5%     TVNPLVVNWRD--NTVISRPGQSQCPRFNKCPEVCWEGVYNDAFLIDRINWISAGVFLDSNQTAENP--VFTVFKDNEVL
 8 NIV-I1.3   100.0%  95.2%     TVNPLVVNWRD--NTVISRPGQSQCPRFNTCPEVCWEGVYNDAFLIDRINWISAGVFLDSNQTAENP--VFTVFKDNEVL
 9 HeV-α1.2 100.0%  78.1%     TVDPLRVQWRN--NSVISRPGQSQCPRFNVCPEVCWEGTYNDAFLIDRLNWVSAGVYLNSNQTAENP--VFAVFKDNEIL
10 HeV-α3   100.0%  78.3%     TVDPLRVQWRN--NSVISRPGQSQCPRFNVCPEVCWEGTYNDAFLIDRLNWVSAGVYLNSNQTAENP--VFAVFKDNEIL
11 HeV-α4   100.0%  78.0%     TVDPLRVQWRN--NSVISRPGQSQCPRFNVCPEVCWEGTYNDAFLIDRLNWVSAGVYLNSNQTAENP--VFAVFKDNEIL
12 HeV-α5   100.0%  78.3%     TVDPLRVQWRN--NSVISRPGQSQCPRFNVCPEVCWEGTYNDAFLIDRLNWVSAGVYLNSNQTAENP--VFAVFKDNEIL
13 HeV-α6   100.0%  78.3%     TVDPLRVQWRN--NSVISRPGQSQCPRFNVCPEVCWEGTYNDAFLIDRLNWVSAGVYLNSNQTAENP--VFAVFKDNEIL
14 HeV-β1      99.8%  78.8%     TVDPLKVQWRN--NSVISRPGQSQCPRFNVCPEVCWEGTYNDAFLIDRLNWISAGVYLNSNQTAENP--VFAVFKDNEIL
15 HeV-β3      99.8%  79.1%     TVDPLKVQWRN--NSVISRPGQSQCPRFNVCPEVCWEGTYNDAFLIDRLNWISAGVYLNSNQTAENP--VFAVFKDNEIL
16 CedV-1      98.0%  28.9%     KWKPFTPNWMN--NTVISRPNQGNCPRYHKCPEICYGGTYNDIAPLDLGKDMYVSVILDSDQLAENP--EITVFNSTTIL
17 CedV-2      98.0%  29.1%     KWKPFTPNWMN--NTVISRPNQGNCPRYHKCPEICYGGTYNDIAPLDLGKDMYVSVILDSDQLAENP--EITVFNSTTIL
18 GhV         98.3%  24.9%     DLDKYQLDWLD--TPYISRPGGSECPFGNYCPTVCWEGTYNDVYSLTPNNDLFVTVYLKSEQVAENP--YFAIFSRDQIL
19 AngV        98.0%  16.9%     FEKNQKITFDEANYTVYERYTEGSCPINSSCPAFCHSSGYNDIWANDIDSNITFGIYNSDSHSYGRP--VFFMANQSGIM
20 LayV-2      96.8%  19.3%     DISVRSTTWLKEKDPIMKVLTTCTNTDKDMCPEICNTRGYQDIFPLSEDSSFYTYIGITPSNEGTKS--FVAVKDDAGHV
21 LayV-3      96.8%  19.2%     DISVRSTTWLKEKDPIMKVLTTCTNTDKDMCPEICNTRGYQDIFPLSEDSSFYTYIGITPSNEGTKS--FVAVKDDAGHV
22 MojV        96.8%  19.6%     DISVRSTTWLKSQDPIMKILSTCTNTDRDMCPEICNTRGYQDIFPLSEDSEYYTYIGITPNNGGTKN--FVAVRDSDGHI
23 SHNV5-1     97.7%  18.9%     DISVKSTRWLDAKYPVMYIRSTCTNKVSNMCPEICSTRGYQDIFPLSADSESYTYIGISPKGEGTSS--FVAVRDRDGHH
24 SHNV5-3     97.7%  18.7%     DISVKSTRWLDAKYPVMYIRSTCTNKVSNMCPEICSTRGYQDIFPLSADSESYTYIGISPKGEGTSS--FVAVRDRDGHH
25 DarV-K      97.5%  19.0%     TLIPTEVEWSKLKDPIMDIQNNCHNTNQDMCPAVCSSYGYEDIFPLNIEGNAMTYMSMSRNGEGTSN--FIVARSQDNYV
26 DarV-C      97.5%  19.0%     TLIPTEVEWSKLKDPIMDIQNNCHNTNQDMCPAVCSSYGYEDIFPLNIEGNAMTYMSMSRNGEGTSN--FIVARSQDNYV
27 DewV        96.7%  18.2%     SPVPGTFKWSPLKSPVLAVGTTCHNDNLSMCPQVCHSYGYEDVFPLDVDGTSMTYMSISTDGQRSKA--YVQARSKDGFV
28 MelV        97.0%  18.8%     DMNPDSFKWANFKTPIMNVQTSCRNTNENMCPQVCYGTGYEDIFPLNDDGSSRTYTTITTNGSRTQV--YIRARVNGNNV
29 NinV        97.5%  16.9%     RKSISDISWIG--YASQSRVSTTNCPSNVACPAVCYYPNYFDIYPLDEEGELMTITNYNNPDQADRGDLYTCISTDQEDL
30 SHNV2       96.3%  16.3%     DFPSYKPIYVN--SDIITVRAHGSCRDKNNCPQDCYSPLYADIYPLNLEATLFTSTPIFMGGDNADY--RTVIINSNKIE
31 GakV-1      96.5%  17.2%     RASPQRYEWTD--YMSFETPYYLYCSGGRICPVSCKTWNFTVPTILNPSGSIIIGVKAKSKTGNSAS--MITINTPDEVI
32 GakV-2      96.5%  17.1%     RASPQRYEWTD--YMSFETPYYLYCSGGRICPVSCKTWNFTVPTILNPSGSIIIGVKAKSKTGNSAS--MITINTPDEVI
33 SHNV3       96.3%  17.4%     RTSPQRYDWTD--YMSFETPYYLYCSGGRICPSSCRTWNFTVPTILNPSGEIIIGVKAKSRTGNSMS--MISINTPNEVI
34 ShyV        96.3%  17.1%     RTSPQRYEWTD--YMSFETPYYLYCSGGRMCPSSCRTWNFTVPTILNPSGEIIIGVKAKSKTGNSMS--MISINTPNEVI

                cov    pid 641        :         .         .         .         .         7         .         . 720
 1 NiV-M3     100.0% 100.0%     YRAQLASEDTNAQKTITNCFLLKNKIWCISLVEIYDTGDNVIRPKLFAVKIPEQC---T---------------------
 2 NiV-M5     100.0%  99.5%     YRAQLASEDTNAQKTITNCFLLKNKIWCISLVEIYDTGDNVIRPKLFAVKIPEQC---T---------------------
 3 NiV-B1.5   100.0%  95.7%     YRAQLASEDTNAQKTITNCFLLRNKIWCISLVEIYDTGDNVIRPKLFAVKIPEQC---T---------------------
 4 NiV-B1.6   100.0%  95.3%     YRAQLASEDTNAQKTITNCFLLKNKIWCISLVEIYDTGDNVIRPKLFAVKIPEQC---T---------------------
 5 NiV-B2.4   100.0%  95.3%     YRAQLASEDTNAQKTITNCFLLKNKIWCISLVEIYDTGDNVIRPKLFAVKIPEQC---T---------------------
 6 NiV-B2.5   100.0%  95.8%     YRAQLASEDTNAQKTITNCFLLKNKIWCISLVEIYDTGDNVIRPKLFAVKIPEQC---T---------------------
 7 NiV-B3.1   100.0%  95.5%     YRAQLASEDTNAQKTITNCFLLKNKIWCISLVEIYDTGDNVIRPKLFAVKIPEQC---T---------------------
 8 NIV-I1.3   100.0%  95.2%     YRAQLASEDTNAQKTITNCFLLKNKIWCISLVEIYDTGDNVIRPKLFAVKIPEQC---T---------------------
 9 HeV-α1.2 100.0%  78.1%     YQVPLAEDDTNAQKTITDCFLLENVIWCISLVEIYDTGDSVIRPKLFAVKIPAQC---SES-------------------
10 HeV-α3   100.0%  78.3%     YQVPLAEDDTNAQKTITDCFLLENVIWCISLVEIYDTGDSVIRPKLFAVKIPAQC---SES-------------------
11 HeV-α4   100.0%  78.0%     YQVPLAEDDTNAQKTITDCFLLENVIWCISLVEIYDTGDSVIRPKLFAVKIPAQC---SES-------------------
12 HeV-α5   100.0%  78.3%     YQVPLAEDDTNAQKTITDCFLLENVIWCISLVEIYDTGDSVIRPKLFAVKIPAQC---SES-------------------
13 HeV-α6   100.0%  78.3%     YQVPLAEDDTNAQKTITDCFLLENVIWCISLVEIYDTGDSVIRPKLFAVKIPAQC---SES-------------------
14 HeV-β1      99.8%  78.8%     YQVQLADEDTNAQRTITDCFLLDNVIWCISLVEIYDTGDSVIRPKLFAVKIPAQC---SGN-------------------
15 HeV-β3      99.8%  79.1%     YQVQLADEDTNAQRTITDCFLLDNVIWCISLVEIYDTGDSVIRPKLFAVKIPAQC---SGN-------------------
16 CedV-1      98.0%  28.9%     YKERVSKDELNTRSTTTSCFLFLDEPWCISVLETNRFNGKSIRPEIYSYKIPKYC-------------------------
17 CedV-2      98.0%  29.1%     YKERVSKDELNTRSTTTSCFLFLDEPWCISVLETNRFNGKSIRPEIYSYKIPKYC-------------------------
18 GhV         98.3%  24.9%     KEFPLDAWISSARTTTISCFMFNNEIWCIAALEITRLNDDIIRPIYYSFWLPTDC---RTPYPHTGKM----TRVPLRST
19 AngV        98.0%  16.9%     YEFVIPGSIIAAGQSTTSCLLFYQEVWCMNVIEIRKTLNSSQEIYGFWTKTPLVC---PTKDQYDIAH----AFPLPTPS
20 LayV-2      96.8%  19.3%     ASITILPNYYSITSATISCFMYKEEIWCIAVTEGRKQKENPQRIYAHSYRVQKMC---FNIKPASVVT----SLPSNVTI
21 LayV-3      96.8%  19.2%     ASITILPNYYSITSATISCFMYKEEIWCIAVTEGRKQKENPQRIYAHSYRVQKMC---FNIKPASVVT----SLPSNVTI
22 MojV        96.8%  19.6%     ASIDILQNYYSITSATISCFMYKDEIWCIAITEGKKQKDNPQRIYAHSYKIRQMC---YNMKSATVTV----GNAKNITI
23 SHNV5-1     97.7%  18.9%     ASKEILSSYFSILSSTISCFTYKEEIWCIAVSEGKRTKGSAQRIFAHSYRVIRGC---GHNLVYPNMT----DIVPSFSK
24 SHNV5-3     97.7%  18.7%     ASKEILSSYFSILSSTISCFTYKEEIWCIAVSEGKRTKGSAQRIFAHSYRVIRGC---GHNLVYPNMT----DIVSSFSK
25 DarV-K      97.5%  19.0%     NTMKALGEYFKIITATTSCFTFKDEAWCIVVNEGQHKKGDKQRIFAHSYKLKKSC---IKRSKITDLA----TTSITFNN
26 DarV-C      97.5%  19.0%     NTMKALGEYFKIITATTSCFTFKDEAWCIVVNEGQHKKGDKQRIFAHSYKLKKSC---IKRSRITDLA----TTSITINN
27 DewV        96.7%  18.2%     NSIPALTDYYNIYTSTISCFMYRNDVWCIVINEASLKKNDKQRIYAHSYKLKKEC---VKSDMLMTDM----VTLMQNNK
28 MelV        97.0%  18.8%     GSIQVLNDYYFVYSATTSCFMFKNEGWCIVINEAKLNKNDKQRIYAHSYRLKRLCMSGMRSINSTTGL----TTVVLPSN
```

```
29 NinV        97.5%  16.9%     NRVLDSNVKREVTYTTTSCFVYRRDVWCIVIIVEKSEVREYTEIKPYIYKIKKSC---SLHQGHDAGLRDEEVKVVPLPT
30 SHNV2       96.3%  16.3%     SLVQMFVNYKSIMGTTTTCFMYKDEPWCIVTVRGVRKGYSGVEAISYSYKIKKTC---QNTQPKIGNI----SIPDFPSI
31 GakV-1      96.5%  17.2%     DQYEVFNDYQSIGSTITKCFSYKRQPWCLVLLEGVLKSTGVTETSIQTFKIFRSC---VKHRTYLDSLGTRFYYTVSDNG
32 GakV-2      96.5%  17.1%     DQYEVFNDYQSIGSTITKCFSYKRQPWCLVLLEGVLKSTGVTETSIQTFKIFRSC---VKHRTYLDSLGTRFYYTVSDNG
33 SHNV3       96.3%  17.4%     DQYEVFTDYLSIGSTITKCFLYKRQPWCLVLLEGVPKSTGLTETSIQTFRIYKSC---VKSRTYLDSLGSRYYYVSDQGG
34 ShyV        96.3%  17.1%     DQYEVFKDYLSIGSTITKCFLYKRQPWCLVLLEGVPKSTGQTETSIQTFRIYKSC---VKSRTYLDSLGARYFYVPDQES

                 cov    pid 721          .         .         :         .         ] 770
 1 NiV-M3     100.0% 100.0%     --------------------------------------------------
 2 NiV-M5     100.0%  99.5%     --------------------------------------------------
 3 NiV-B1.5   100.0%  95.7%     --------------------------------------------------
 4 NiV-B1.6   100.0%  95.3%     --------------------------------------------------
 5 NiV-B2.4   100.0%  95.3%     --------------------------------------------------
 6 NiV-B2.5   100.0%  95.8%     --------------------------------------------------
 7 NiV-B3.1   100.0%  95.5%     --------------------------------------------------
 8 NIV-I1.3   100.0%  95.2%     --------------------------------------------------
 9 HeV-α1.2   100.0%  78.1%     --------------------------------------------------
10 HeV-α3     100.0%  78.3%     --------------------------------------------------
11 HeV-α4     100.0%  78.0%     --------------------------------------------------
12 HeV-α5     100.0%  78.3%     --------------------------------------------------
13 HeV-α6     100.0%  78.3%     --------------------------------------------------
14 HeV-β1      99.8%  78.8%     --------------------------------------------------
15 HeV-β3      99.8%  79.1%     --------------------------------------------------
16 CedV-1      98.0%  28.9%     --------------------------------------------------
17 CedV-2      98.0%  29.1%     --------------------------------------------------
18 GhV         98.3%  24.9%     YNY-----------------------------------------------
19 AngV        98.0%  16.9%     PAPAPSQSVPVTEASTITLIQSVEPQGTPTDIPSTTNRDDIQTITNATSG
20 LayV-2      96.8%  19.3%     RS------------------------------------------------
21 LayV-3      96.8%  19.2%     RS------------------------------------------------
22 MojV        96.8%  19.6%     RRY-----------------------------------------------
23 SHNV5-1     97.7%  18.9%     --------------------------------------------------
24 SHNV5-3     97.7%  18.7%     --------------------------------------------------
25 DarV-K      97.5%  19.0%     RSIR----------------------------------------------
26 DarV-C      97.5%  19.0%     RSIR----------------------------------------------
27 DewV        96.7%  18.2%     SRTR----------------------------------------------
28 MelV        97.0%  18.8%     RRTR----------------------------------------------
29 NinV        97.5%  16.9%     EKPKPEARSSYSFWF-----------------------------------
30 SHNV2       96.3%  16.3%     KPVR----------------------------------------------
31 GakV-1      96.5%  17.2%     NKTKQTYIPGSDT-------------------------------------
32 GakV-2      96.5%  17.1%     NKTKQTYIPGSDT-------------------------------------
33 SHNV3       96.3%  17.4%     NRTRQAYKLTSDT-------------------------------------
34 ShyV        96.3%  17.1%     NRTRQAYKLTSDT-------------------------------------
```

MView 1.67, Copyright © 1997-2020 Nigel P. Brown
